# Regional differences in the profile of disabled community-dwelling older adults: A European population-based cross-sectional study

**DOI:** 10.1371/journal.pone.0208946

**Published:** 2018-12-11

**Authors:** Javier Jerez-Roig, Marina Bosque-Prous, Maria Giné-Garriga, Caritat Bagur-Calafat, Dyego L. Bezerra de Souza, Ester Teixidó-Compañó, Albert Espelt

**Affiliations:** 1 Research group on Methodology, Methods, Models and Outcomes of Health and Social Sciences (M3O), Faculty of Health Science and Welfare, Centre for Health and Social Care Research (CESS), University of Vic-Central University of Catalonia (UVIC-UCC), Vic, Spain; 2 Blanquerna Faculty of Psychology, Education and Sport Sciences, Ramon Llull University, Barcelona, Spain; 3 Postgraduate Program in Collective Health, Odontology Department, Federal University of Rio Grande do Norte, Natal, Brazil; 4 Faculty of Health Sciences, Universitat Oberta de Catalunya, Barcelona, Spain; 5 School of Health and Life Sciences, Glasgow Caledonian University, Glasgow, United Kingdom; 6 Department of Physiotherapy, Universitat Internacional de Catalunya, Sant Cugat del Vallès, Spain; 7 Graduate Program in Collective Health, Department of Collective Health, Federal University of Rio Grande do Norte, Natal, Brazil; 8 Faculty of Health Sciences of Manresa, University of Vic–Central University of Catalonia, Manresa, Spain; Nord University, NORWAY

## Abstract

The main objective of this work was to estimate the prevalence of disability in European community-dwelling older adults, as well as to investigate differences in the profile of disabled older adults between European regions (Northern, Central, Eastern and Southern). A cross-sectional study based on wave 6 (2015) of the Survey of Health, Ageing and Retirement in Europe (SHARE) was conducted. Community-dwelling participants aged 65–84 were selected (n = 33,369). Disability was defined as presenting at least one functional limitation in basic activities of daily living (BADL). Sociodemographic, health services, lifestyle and health-related variables were analyzed. Statistical analysis was carried out through the Chi-square and ANOVA tests for bivariate analysis, and Poisson regression for multivariate analysis. Overall prevalence of disability was 13.8%: 9.4% in the Northern region, 13.1% in the Southern region, 13.6% in the Central region, and 16.6% in the Eastern region. Portugal, Poland, Estonia and Belgium showed the highest prevalence of BADL limitations, while Sweden, Denmark, Greece and Switzerland showed the lowest prevalence. Besides, disabled older adults from East Europe presented the most disadvantaged health profile, followed by the Southern region. On the other hand, disabled older adults living in the Northern region showed the most advantaged characteristics of most variables, except for smoking and polypharmacy.

## Introduction

Population ageing process is a common phenomenon in most countries, caused by a decrease in the fecundity and mortality rates [1]. As a result, life expectancy has progressively raised as well as the risk of developing chronic illnesses, accompanied by a decline of the capacity to perform activities of daily living (ADL), losing autonomy and becoming dependent on other’s care [1]. This situation leads to a worsening quality of life of the older adults, and this deterioration had shown to be unequally distributed among the population [2].

The globalization phenomenon and the ageing population process are important challenges that produce strong pressure on the European welfare state and the need for reforms, especially on pensions and health systems [3]. Tackling and reducing disability should be the cornerstone to achieve the so-called ‘successful ageing’ [4]. Thus, in response to this major public health issue, the ‘European Disability Strategy 2010–2020’ framework aims at implementing the United Nations Convention on the rights of persons with disabilities through the promotion of accessibility, participation, equality, social protection, health, education and training, among other areas of action.

According to the recommendations of the World Report on Disability of the World Health Organization (WHO) and the World Bank Group, research on disability must be strengthened and supported [5]. Identifying the main characteristics of the disabled population is fundamental in order to address adequate preventive and health promotion policies [2]. Patterns of limitations in function can vary substantially within countries and also between geographical areas over time and, in this sense, cross-sectional comparative studies provide useful information about function and disability onset [6,7].

Social models are diverse among European states, and the public expenditure on long-term care varies across regions. The literature illustrates different social and welfare systems across Europe that can be classified in five provisional models: Scandinavian (Northern), Bismarckian (Central), Anglo-Saxon, Eastern and Southern [8].

As far as we know, few studies have analyzed disability in the European regions [3,5,6] and there is still no published works on disability of the last released wave of the Survey of Health, Ageing and Retirement in Europe (SHARE). Furthermore, research on the differences in the sociodemographic and health-related profile among disabled adults is lacking. This information can help indicate not only the quality of life among the older population, but also the social expenditure that is needed regarding health and social care in this population, with an emphasis on the programs aimed at enhancing healthy ageing and disability assistance [2].

Therefore, the present study sought to estimate the prevalence of disability among European community-dwelling aged 65–84 years, as well as to analyze the differences in the profile of disabled older adults among the European regions.

## Methods

We present a cross-sectional and population-based study using data from wave 6 of the SHARE project (www.share-project.org). The aforementioned study is the first multidisciplinary, cross-country, longitudinal research project conducted in Europe. The SHARE Project respects Helsinki Declaration, in terms of anonymity of the participants and obtaining of written consents. Wave 4 and the posterior waves have been reviewed and approved by the Ethics Committee of the Max Planck Society. Furthermore, the country implementations of SHARE were reviewed and approved by the respective ethics committees or institutional review boards [9]. Further details about data collection, sampling procedures and other methodology aspects are available in the project’s website (www.share-project.org) [9], and wave 6 questionnaire is available in S1 File.

Data from the following 17 European countries that participated in wave 6 (2015) were considered: Austria, Belgium, Croatia, Czech Republic, Denmark, Estonia, France, Germany, Greece, Italy, Luxembourg, Poland, Portugal, Spain, Sweden, Slovenia and Switzerland. Although sampling differed slightly, all countries obtained probabilistic samples [10]. The inclusion criterion was being 65–84 years old. We considered the aforementioned age range because we wanted to study disability in the older population. According to the WHO, "older people" from developed countries are those aged 65 and over. Individuals aged 85 and over were not included in the study due to lack of comparability across countries’ samplings [11]. Institutionalized individuals and cases with incomplete data on age and limitations on basic activities of daily living (BADL) were excluded from this study.

The dependent variable was *disability*, defined as presenting limitation in one or more BADL. As asked in the questionnaire, any difficulty because of a physical, mental, emotional or memory problem during at least 3 months was considered. The BADL included were dressing, walking across a room, bathing or showering, eating, getting in or out of bed and using the toilet (including getting up or down) [11]. From these items, a 0–6 index was created, in order to compare the European regions regarding disability level [6].

The main independent variable was the European region classified as the literature suggested: Northern (Denmark and Sweden), Central (Austria, Germany, France, Switzerland, Belgium and, Luxembourg), Eastern (Czech Republic, Poland, Slovenia, Estonia and, Croatia) and Southern (Spain, Italy, Greece and, Portugal) [8].

Other independent variables that we used to compare the characteristics of disabled individuals across countries were: sociodemographic variables (age, gender, country, region, rural area and educational level), health services-related variables (satisfaction with public insurance, area location (urban/rural), hospitalizations, ‘could not visit the doctor because of cost’, and ‘could not visit the doctor because of long waiting times’), lifestyle-related variables (smoking, physical inactivity and obesity) and health-related variables (self-rated health, polypharmacy, ≥ 2 chronic diseases, memory, numeracy, depressive symptoms, loneliness, and quality of life). The educational level categories from the International Standard Classification of Educational Degrees (ISCED) of 1997 and 2011 were dicothomized (lower secundary level or less; upper secondary level or more) [12].

The variables ‘hospitalizations’ (yes/no), ‘could not visit the doctor because of long waiting times’ and ‘could not visit the doctor because of cost’ (yes/no) referred to the previous 12-month period. Physical inactivity was defined as never or hardly ever engaging in moderate-intensity activities such as gardening, cleaning the car or doing a walk, or vigorous physical activities such as sports, heavy housework or a job that involves physical labour [6]. For smoking, the categories smoker or former smoker versus non-smoker were considered. Obesity was identified when body mass index (BMI) was ≥ 30.0 kg/m^2^; BMI was estimated from self-reported weight and height [13].

Polypharmacy was considered when the individual reported taking at least 5 of the following 13 drugs per day: for high blood cholesterol, high blood pressure, coronary or cerebrovascular diseases, other heart diseases, diabetes, joint pain or joint inflammation, other pain (e.g. headache, back pain, etc.), sleep problems, anxiety or depression, osteoporosis, stomach burns, chronic bronchitis, and for suppressing inflammation (only glucocorticoids or steroids).

Regarding illnesses, the following conditions were considered: heart attack, hypertension, high blood cholesterol, stroke or cerebral vascular disease, diabetes, chronic lung disease, cancer, Parkinson disease, cataracts, hip fracture, other fractures, dementia, affective/emotional disorders (including anxiety, nervous or psychiatric problems) and rheumatoid arthritis/osteoarthritis [11]. Memory was evaluated by an immediate and a delayed 10-word recall test (1 point for each correctly recalled noun, maximum: 20); and numeracy, assessed by a mathematical (substraction) performance test (1 point for each correct answer, maximum: 5) [4]. Both variables were dicothomized by the median.

As a quality of life measure, a modified and validated SHARE version of the CASP-12 was used, a shortened version of CASP-19. This scale includes 12 items reflecting four dimensions: control, autonomy, self-realization and pleasure (the first letter of each form the acronym CASP). Every question is answered on a 4-point Likert scale (often, sometimes, rarely and never); higher scores indicate a higher level of quality of life [14]. The lower tertile was considered to identify low quality of life. Depressive symptoms were assessed through the Euro-Depression scale (EURO-D), an instrument originally developed to harmonize data on late-life depression from population-based studies in different European countries. This unidimensional, easy administered and validated scale contains items about depression, pessimism, suicidality, guilt, sleep, interest, irritability, appetite, fatigue, concentration, enjoyment and tearfulness in the last month. Total score ranges from 0 to 12, with higher scores indicating more depressive symptoms, and the cutt off point is 3/4 [15–17].

Finally, loneliness was assessed by a 3-item short version of the loneliness scale devoloped by Hughes et al. (2004). The total score ranges from 3 (not lonely) to 9 (very lonely). This variable was dicothomized by the highest decile [18].

Prevalence of disability by country, age group and gender were classified by quartiles and depicted in maps. To compare differences between European regions, a bivariate analysis was carried out through the Chi-square test for dichotomous variables and one-way ANOVA for quantitative variables. To estimate the relationship between the dependent variable and regions, multivariate Poisson regression models with robust variance were used for multivariate analysis, obtaining prevalence ratios and their confidence intervals at 95% [19]. Statistical analysis was conducted using STATA 14.0, considering a significance level of 95%.

## Results

Among 33,471 individuals, 12 and 65 were not included due to missing data on the age and BADL limitations variables, respectively. After excluding 25 subjects permanently living in nursing homes, the final sample resulted on 33,369, from which 4,617 presented limitations in one or more BADL. Therefore, the prevalence of disability was 13.8% (95% CI: 13.5–14.2%) distributed as follows: 9.4% (95%CI: 8.6–10.3%) in the Northern region, 13.1% (95%CI: 12.4–13.8%) in the Southern region, 13.6% (95%CI: 13.0–14.3%) in the Central region, and 16.6% (95%CI: 15.8–17.3%) in the Eastern region.

Table 1 presents the descriptive analysis of the overall sample by age group and gender. Lower educational level, physical inactivity, poor self-rated health, low quality of life, depressive symptoms, loneliness, polypharmacy, comorbidity, low memory and numeracy score were found among older women. The smoking habit was more frequent among younger men (65–74) and hospitalizations among older men (75–84). Obesity was more common among women. The proportion of individuals living in a rural area was fairly consistent among genders and age groups.

**Table 1:** Weighted descriptive analysis (absolute and relative frequencies) of the independent variables in the sample of community-dwelling European older adults, by gender and age group in 2015.

	Men				Women			
	65–74		75–84		65–74		75–84	
	n	%	n	%	N	%	n	%
	9657	100.0	5596	100.0	11087	100.0	7029	100.0
**Country**								
Austria	538	5.6	299	5.3	705	6.4	396	5.6
Belgium	737	7.6	417	7.5	824	7.4	555	7.9
Croatia	360	3.7	149	2.7	360	3.2	207	2.9
Czech Republic	784	8.1	369	6.6	1096	9.9	533	7.6
Denmark	550	5.7	247	4.4	563	5.1	284	4.0
Estonia	648	6.7	467	8.3	941	8.5	883	12.6
France	490	5.1	314	5.6	599	5.4	435	6.2
Germany	678	7.0	412	7.4	648	5.8	352	5.0
Greece	680	7.0	431	7.7	702	6.3	544	7.7
Italy	816	8.4	516	9.2	906	8.2	505	7.2
Luxembourg	237	2.5	93	1.7	217	1.9	103	1.5
Poland	237	2.5	130	2.3	295	2.7	159	2.3
Portugal	297	3.1	141	2.5	329	3.0	141	2.0
Slovenia	607	6.3	328	5.9	695	6.3	475	6.8
Spain	802	8.3	598	10.7	877	7.9	713	10.1
Sweden	726	7.5	426	7.6	862	7.8	442	6.3
Switzerland	470	4.9	259	4.6	468	4.2	302	4.3
**Lower educational level**	3545	37.5	2678	48.5	4941	45.2	4210	60.7
**Rural area**(%)	**3141**	**33.9**	**1790**	**33.5**	**3517**	**33.3**	**2186**	**33.1**
**Physically inactive**	860	8.9	959	17.1	1077	9.7	1651	23.5
**Current/former smoker**	5727	60.3	3008	54.3	3616	33.0	1318	18.9
**Obesity**	2249	23.5	977	17.8	2668	24.6	1492	22.3
**Fair/poor self-rated health**	3638	37.7	2862	51.1	4622	41.7	4202	59.8
**Low quality of life**	2527	27.7	1933	37.9	3420	32.2	2973	46.5
**Depressive symptoms**	1546	16.8	1262	24.3	3387	31.4	2713	41.2
**Loneliness**	364	3.9	345	6.6	727	6.7	775	11.7
**≥ 5 drug types**	2169	27.9	1854	37.1	2585	27.9	2672	41.0
**≥ 2 chronic diseases**	4972	51.5	3479	52.2	6262	56.5	4927	70.1
**Low memory score** (≤8)	4153	45.1	3361	65.0	4075	37.8	4154	63.1
**Low numeracy score** (≤4)	3019	32.5	2261	43.3	4377	40.4	3672	55.3
**Hospitalizations**	1665	17.3	1219	21.8	1635	14.8	1442	20.5
**Bad satisfaction with health system**	1494	15.6	902	16.3	1890	17.2	1166	16.8
**Could not visit doctor because of cost**	311	3.2	204	3.6	566	5.1	358	5.1
**Could not visit doctor because of long waiting times**	763	7.9	515	9.2	1112	10.0	736	10.5

**Fig 1:**
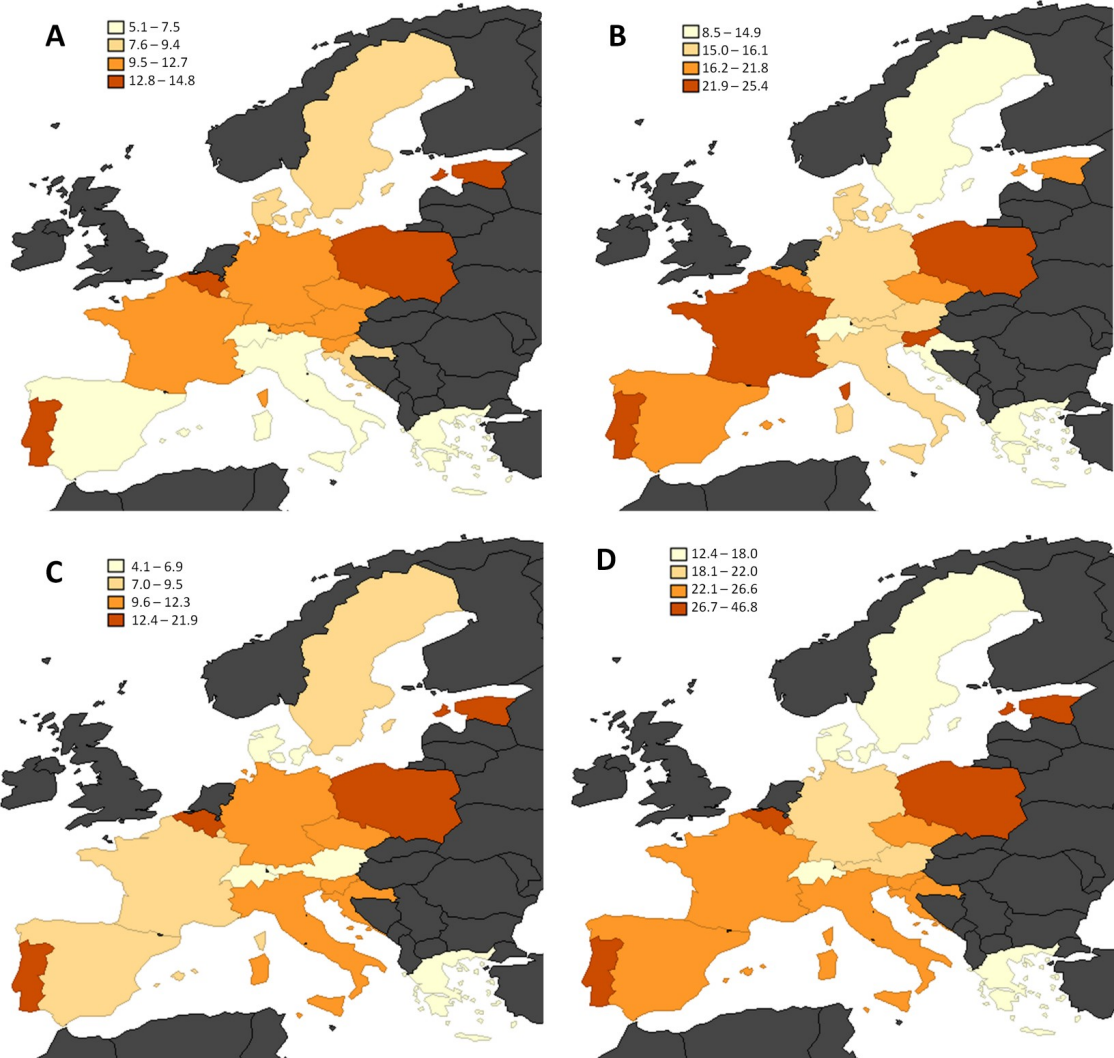
Prevalence of disability (1 or more BADL limitations), classified by quartiles, in 17 European countries in men aged 65–74 (A), men aged 75–84 (B), women aged 65–74 (C) and women aged 75–84 (D) in 2015.

Fig 1 contains the maps which show the prevalence of disability, divided into quartiles, by gender and age group in 17 European countries. Sweden, Denmark, Switzerland, Austria and Greece were always in the first or second quartile in both genders and age groups while Poland, Estonia, Czech Republic, Belgium and Portugal were always in the third or fourth quartile.

Table 2 shows the descriptive and bivariate analysis of the subsample of disabled European adults aged 65–74, comparing the four European regions. It is worth noting that the Southern region presents the highest disability index as well as a higher proportion of women, low educational level, physical inactivity, low quality of life, depressive symptoms, loneliness, polypharmacy, low memory and numeracy score, low satisfaction with public insurance and a higher proportion of disabled individuals who could not visit the doctor because of cost and long waiting lists. The highest frequency of disabled adults who live in a rural area, that had to be hospitalized, with obesity, poor/fair self-rated health, and two or more chronic diseases, was identified in the Eastern region. On the other hand, the Northern region presented the highest proportion of smokers or former smokers.

**Table 2:** Descriptive (frequencies or mean, and standard deviation) and bivariate (Chi-square and ANOVA tests) analysis of disabled older adults in Europe aged 65–74 (n = 2,023), according to European regions and independent variables in 2015.

	Total	Northern	Central	Eastern	Southern	*p*
Mean disability index (0–6 BADL limitations)	2.0 (1.4)	1.8 (1.4)	1.8 (1.3)	2.1(1.5)	2.3 (1.6)	<0.001*
Women (%)	54.3	49.5	47.5	57.0	61.4	<0.001*
Lower educational level (%)	50.2	36.2	37.7	45.5	81.7	<0.001*
Rural area (%)	36.0	35.3	37.5	41.1	25.6	<0.001*
Physically inactive (%)	37.7	20.5	32.5	39.1	50.3	<0.001*
Current/former smoker (%)	47.3	66.0	56.6	43.6	32.1	<0.001*
Obesity (%)	40.7	26.7	41.0	46.4	37.4	<0.001*
Fair/poor self-rated health (%)	80.4	63.5	71.1	88.8	87.6	<0.001*
Low quality of life (CASP, %)	57.6	29.1	45.5	64.1	79.0	<0.001*
Depressive symptoms (%)	55.9	36.2	49.3	59.1	70.7	<0.001*
Loneliness (%)	15.5	4.3	13.2	16.5	23.3	<0.001*
≥ 5drug types (%)	56.8	53.3	56.1	54.4	62.8	0.027*
≥2 chronic diseases (%)	81.0	71.9	79.1	84.9	81.5	<0.001*
Low memory score (%)	51.9	38.0	43.2	52.6	71.5	<0.001*
Low numeracy score (%)	47.3	41.0	38.5	43.1	71.4	<0.001*
Hospitalizations (%)	33.0	26.3	34.1	34.9	31.4	0.109
Low satisfaction with public insurance (%)	22.6	15.6	10.3	23.8	40.7	<0.001*
Could not visit doctor because of cost (%)	8.2	1.0	3.4	8.0	18.1	<0.001*
Could not visit doctor because of waiting times (%)	15.9	12.6	6.4	19.4	24.9	<0.001*

* Statistically significant (*p* <0.05)

Table 3 contains the descriptive and bivariate analysis of the subsample of disabled European adults aged 75–84. Similarly, the Southern region presented the highest disability index as well as a higher proportion of low educational level, physical inactivity, low quality of life, depressive symptoms, loneliness, polypharmacy, low memory and numeracy score, low satisfaction with public insurance and a higher proportion of disabled individuals who could not visit the doctor because of cost. The higher frequency of older women with obesity, poor/fair self-rated health, two or more chronic diseases and those who could not visit the doctor because of long waiting lists were identified in the Eastern region. On the other hand, the Central region had the highest proportion of disabled adults living in a rural area, and the Northern region the highest frequency of smokers or former smokers and individuals who had been hospitalized during the previous year.

**Table 3:** Descriptive (frequencies or mean, and standard deviation) and bivariate (Chi-square and ANOVA tests) analysis of disabled older adults in Europe aged 75–84 (n = 2,594), according to European regions and independent variables in 2015.

	Total	Northern	Central	Eastern	Southern	*p*
Mean disability index (0–6 BADL limitations)	2.3 (1.7)	2.0 (1.6)	2.1 (1.5)	2.4 (1.6)	2.6 (1.8)	<0.001*
Women (%)	62.0	50.0	61.3	65.1	62.1	0.002*
Lower educational level (%)	63.2	46.2	53.2	54.7	89.6	<0.001*
Rural area (%)	34.8	29.8	40.6	38.9	24.0	<0.001*
Physically inactive (%)	51.9	36.0	47.2	50.4	62.9	<0.001*
Current/former smoker (%)	31.7	57.1	35.7	27.1	26.5	<0.001*
Obesity (%)	29.1	22.8	27.2	35.2	25.3	<0.001*
Fair/poor self-rated health (%)	85.2	72.6	75.2	93.6	89.0	<0.001*
Low quality of life (CASP, %)	65.9	47.1	45.9	75.6	85.1	<0.001*
Depressive symptoms (%)	59.2	44.0	51.2	64.1	68.1	<0.001*
Loneliness (%)	18.4	12.9	11.0	19.9	28.2	<0.001*
≥ 5 drug types (%)	63.9	65.8	60.6	61.2	70.3	<0.001*
≥ 2 chronic diseases (%)	84.8	81.7	82.1	87.4	85.4	0.014*
Low memory score (%)	75.4	70.6	66.2	75.9	89.0	<0.001*
Low numeracy score (%)	60.4	46.2	50.4	58.0	82.4	<0.001*
Hospitalizations (%)	34.4	39.5	36.7	34.8	30.0	0.017*
Low satisfaction with public insurance (%)	21.0	10.2	9.5	23.9	33.0	<0.001*
Could not visit doctor because of cost (%)	5.9	0	1.9	4.9	13.2	<0.001*
Could not visit doctor because of waiting times (%)	12.4	7.6	3.5	17.6	17.1	<0.001*

* Statistically significant (*p* <0.05)

Finally, the results of the multivariate analysis are shown in Table 4. After adjusting for the other independent variables and considering the Northern region as the reference, some differences between regions were statistically significant. The Eastern region presented a higher proportion of disabled older adults living in a rural area, physically inactive, obese, with a negative self-rated health, low quality of life, depressive symptoms, comorbidity (≥ two chronic diseases), low satisfaction with public insurance and individuals who could not visit the doctor because of cost and long waiting lists. The Southern region presented a higher proportion of disabled people physically inactive, individuals who could not visit the doctor because of cost, low quality of life, low numeracy score, and not satisfied with public insurance. On the other hand, the rate of hospitalizations and people living in a rural area were significantly lower in the Southern region.

**Table 4:** Multivariate Poisson analysis (indicating prevalence ratios) of disabled older adults in Europe aged 65–84 (n = 4,617), according to European regions and independent variables in 2015.

	Northern	Central	Eastern	Southern
Age (75–84)	1	1.09 (0.98–1.22)	0.99 (0.89–1.12)	1.03 (0.91–1.17)
Women	1	0.97 (0.86–1.08)	0.99 (0.88–1.11)	0.89 (0.78–1.00)
Lower educational level	1	1.01 (0.92–1.12)	1.02 (0.92–1.14)	3.44 (2.70–4.34)*
Rural area	1	1.23 (1.02–1.47)*	1.25 (1.04–1.51)*	0.66 (0.53–0.83)*
Physically inactive	1	1.30 (1.10–1.53)*	1.23 (1.04–1.45)*	1.33 (1.11–1.59)*
Current/former smoker	1	0.77 (0.69–0.87)*	0.60 (0.53–0.68)*	0.51 (0.44–0.60) *
Obesity	1	1.25 (1.03–1.53)*	1.45 (1.19–1.78)*	1.14 (0.90–1.44)
Fair/poor self-rated health	1	1.00 (0.94–1.08)	1.16 (1.08–1.24)*	1.07 (0.99–1.16)
Low quality of life	1	1.06 (0.92–1.22)	1.36 (1.19–1.56)*	1.43 (1.24–1.64)*
Depressive symptoms	1	1.21 (1.06–1.39)*	1.20 (1.05–1.38)*	1.15 (0.99–1.34)
Loneliness	1	1.12 (0.77–1.63)	1.19 (0.82–1.72)	1.39 (0.94–2.05)
≥ 5 drug types	1	0.88 (0.80–0.97)*	0.78 (0.71–0.87)*	0.85 (0.76–0.95)*
≥2 chronic diseases	1	1.04 (0.98–1.11)	1.07 (1.00–1.13)*	1.03 (0.96–1.11)
Low memory score	1	0.97 (0.87–1.08)	1.08 (0.97–1.21)	1.09 (0.98–1.23)
Low numeracy score	1	1.02 (0.88–1.17)	1.06 (0.92–1.23)	1.29 (1.11–1.49)*
Hospitalizations	1	1.02 (0.86–1.21)	0.92 (0.77–1.09)	0.76 (0.63–0.92)*
Low satisfaction with public insurance	1	0.80 (0.58–1.10)	1.40 (1.04–1.89)*	2.08 (1.52–2.85)*
Could not visit doctor because of cost	1	5.52 (1.30–23.47)*	5.05 (1.21–21.04)*	9.26 (2.24–38.17)*
Could not visit doctor because of waiting times	1	0.51 (0.34–0.76)*	1.45 (1.03–2.04)*	1.21 (0.85–1.74)

*Statistically significant (*p* <0.05)

In the Central region, the prevalence ratio of older adults living in a rural area, physical inactivity, obesity, depressive symptoms, and inaccessibility of visiting a doctor because of cost were significantly higher. However, inaccessibility to visiting a doctor because of long waiting lists was lower than in the Northern region. The latter, however, presented the highest rate of tobacco use and polypharmacy. Differences between the regions regarding the prevalence ratios of the other independent variables (age, gender, loneliness, and low memory score) were not statistically significant.

## Discussion

The aim of the present study was twofold: to estimate the prevalence of disability among European community-dwelling older adults, as well as to analyze the differences in the profile of disabled older adults between the European regions. According to the results of this study, disability (one or more limitations in BADL) affected approximately 14% of community-dwelling older adults of 17 European countries. Comparison with other studies is limited due to the different definitions of disability, such as mean BADL limitations, mean instrumental ADL limitations, mobility limitations or any difficulty undergoing at least one BADL, as considered in our work [6,7]. Crimmins et al. (2011) used the same definition as in the present study, and data from 11 countries that participated in the first wave of SHARE (2004–2005) were analyzed. They showed a lower prevalence (9.7% in males and 11.6% in females) than the one identified in our study, but their work also included younger people aged 50–64 [20]. A cross-sectional study conducted in older people from Barcelona in 2006 found a prevalence of 30% in men and 53% in women, but it analyzed older adults aged 85 and over [2].

As shown in previous epidemiological studies, functioning is a dynamic health issue that varies not only between countries but also longitudinally within the same country [21,22]. Developed countries have shown a reduction of the total lifetime with disability in the last two decades [21]. This is the so-called disability transition model, characterized by the compression of morbidity and the postponement of severe disability to more advanced ages [21]. In Southern Europe, the latter process is related to the sociopolitical changes that occurred during the second half of the last century, particularly since the advent of the democracy regimes [21]. However, this trend is uncertain in the near future, due to the impact of current issues such as the increase in the prevalence of chronic diseases, risk factor exposures or inequalities [7].

In spite of the observed differences between countries, they seem to cluster strongly within each welfare regime, regarding most measures of longstanding illness [8]. In line with Wahrendorf et al. (2013), who analyzed the 2006’s SHARE wave, our study also found higher prevalence of disability in Eastern and Southern Europe than in the Central and Northern regions [6]. Besides, Chatterji et al. identified much larger proportions of individuals who were disabled across all ages in Southern Europe (Italy, Spain and Greece) [7]. These epidemiological studies support the hypothesis that the less developed social policies and more pronounced socioeconomic inequalities are related to higher disability levels and earlier beginning of the disability process [6,7].

These differences may be partially related to the different welfare systems between social democratic, Bismarckian, southern and Eastern European regions [8,23]. The work of Eikemo et al. (2008) pointed out larger health inequalities in South European welfare regimes, compared with Central European countries, characteristic from Central Europe. East European welfare regimes showed the highest prevalence of limiting long standing illness and poor self-rated health [8]. Eastern and Southern Europe have faced major social, economic and political challenges during an extended period of time, in contrast to economically and politically more consolidated and stable countries. Poorer working conditions, stronger health-adverse effects, poverty or social exclusion are important issues among older adults living in these regions [6].

Regarding prevalence differences between countries, the results of our work showed lower prevalence of disability in Sweden, Denmark, Switzerland, Austria and Greece, whereas Poland, Estonia, Czech Republic, Belgium and Portugal presented high proportion in both genders and age groups. Similar results have been reported in other analysis of the SHARE project in which, among 14 European countries, Poland was the one with less active agers, followed by Spain. Nordic countries such as Denmark and Sweden were the countries with most active older adults [4].

In this line, the Courage study conducted among people aged 18 and over found higher disability scores (defined as limitations in basic and instrumental ADL) in Poland than Finland or Spain [24]. Furthermore, Finnish people reported better well-being and achieved better scores of active ageing [23,24]. Poland also presented the highest proportion of current smokers and sedentary lifestyle, and the worst indicators of cognition, social participation, social contacts, control, coping, and environmental safety. Finland, on the other hand, had the best indicators in most components and, Spain showed average results in these active ageing components, but low education and occupation roles were outstanding [23].

Unlike what was observed by Chatterji et al. (2015) in a longitudinal analysis with SHARE data from the first waves (2004 to 2008), our study did not find the largest proportions of disabled adults in Italy, Spain and Greece [7]. In fact, Greece presented one of the lowest proportions for both age groups and genders, along with some Central and Northern countries such as Denmark, Sweden, Switzerland or Austria. Italy and Spain presented low prevalence in men aged 65–74 but the proportion of disabled adults increased in the oldest age group and especially in women.

The second main objective of this study was to investigate the differences in the profile of disabled older adults between four European regions (Northern, Central, Eastern and Southern). In general, the Eastern region presented the less advantaged profile, followed by the Southern and Central regions. In line with other studies, indicators were more favorable in Northern Europe, except for smoking and polypharmacy [23,25].

It is worth noting that all Southern countries worsen the results in women, indicating the feminization of the disabling process that has been commented in the literature. A cross-sectional study analyzing Portuguese, Italian and Spanish data from the wave 4 (2011) of the SHARE project found that functional limitations were unequally distributed: women and the worse-off tended to be affected more severely and earlier [26]. In this line, the Courage study found gender inequalities only in Spain regarding active ageing (men scored higher), but not in Poland and Finland. On the other hand, gender differences tended to be reduced in Northern Europe.

Regarding lifestyle-related variables, the highest prevalence of tobacco use, physical inactivity and obesity was found in the Northern, Southern and Easter regions, respectively. Nowadays, the so-called ‘obesity epidemic’ has arisen because of low levels of physical activity and increase of caloric intake in Europe. Among high-income countries, older women of low social class present the highest prevalence of obesity [25].

According to the results, disabled older adults living in Southern Europe suffer more commonly from psychological and cognitive issues, such as low cognitive performance, isolation or low quality of life. However, southern disabled older people showed the lowest rate of hospitalizations. It is well known that family support is greater in the Mediterranean countries and, therefore, individuals are more likely to be cared at home [27].

Some variables related with access to health system were analyzed in this work, such as living in a rural area or the limitation to access the doctor due to cost or waiting lists. These type of issues can undoubtedly impact on the provision of health and community care services and create inequities that affect the maintenance of physical and mental health [28]. Overall, the Eastern and Southern regions showed the worst indicators.

Some limitations of this work must be recognized, firstly regarding the sampling process. Although SHARE follows a probabilistic method, it could have added some errors in the representativeness of some subsamples, such as the institutionalized older adults, which can lead to underestimation of the prevalence of disability among women and the oldest old. However, this problem has been minimized in this work, because institutionalized adults and those aged 85 and over were excluded.

Moreover, difficulties in conducting a large study with heterogeneous populations and countries should be recognized. However, these difficulties were offset by the harmonization and standardization of questionnaires and procedures applied, secured thanks to the meticulous process of cultural adaptation as well as the high professionalism of pollsters and highly trained interviewers. Since SHARE is an epidemiological study, some information was rated by the individual, as in the case of activity limitations, disease, weight or height.

Concerning data loss, the variables ‘numeracy score’, ‘memory score’, ‘loneliness’, ‘depressive symptoms’ and ‘quality of life’ contained a proportion of missing information between 11% and 22% in the subsample of disabled older adults. However, it is noteworthy that the remaining variables had more than 95% of complete data.

On the other hand, this study stands out for the sample size, current data and novelty. As far as we know, it is the first work comparing the profile of disabled older adults from European regions.

## Conclusions

According to the results, it can be concluded that the overall prevalence of disability in community-dwelling older adults from 17 European countries was almost 14%; this proportion is higher in the Eastern region, followed by the Central, Southern and Northern regions. By countries, Sweden, Denmark, Switzerland, Austria and Greece were contained in the first and second quartile in both genders and age groups, while Poland, Estonia, Czech Republic, Belgium and Portugal were located in the third and fourth quartile.

Regarding the profile of the subsample of European disabled older adults, the Eastern region showed the most disadvantaged profile for the majority of the health-related variables, followed by the Southern region. On the other hand, Northern Europe presents the most advantaged profile, except for the deleterious habit of smoking and polypharmacy. This work may help understand the current picture of disability in the European older population and, especially arouse the interest of policy makers and stakeholders to design and apply health policies to prevent and manage this important public health issue.

## Supporting information

S1 File Questionnaire of the wave 6 of the survey on health, ageing and retirement in Europe project. (PDF)
