# Supplementary material for: Regional differences in the profile of disabled community-dwelling older adults: A European population-based cross-sectional study
Source: PLoS One. 2018 Dec 11;13(12):e0208946. doi: 10.1371/journal.pone.0208946 (PMC6289507; doi:10.1371/journal.pone.0208946)
Supplement: S1 File — (PDF) [file pone.0208946.s001.pdf]

System preset value SampID
System preset value RespID
System preset value index
System preset value MN101_Longitudinal
BLOCK

System preset value InterviewMonth_Last
System preset value InterviewYear_Last
System preset value InterviewMonth_Regular
System preset value InterviewYear_Regular
System preset value SNInterviewMonth
System preset value SNInterviewYear
System preset value PRELOAD_WAVE1_COMPLETED
System preset value PRELOAD_WAVE2_COMPLETED
System preset value PRELOAD_WAVE3_COMPLETED
System preset value PRELOAD_WAVE4_COMPLETED
System preset value PRELOAD_WAVE5_COMPLETED
System preset value PRELOAD_DN007_CitizenshipCountry
LOOP := 1 TO 2

System preset value PRELOAD_DN026_NaturalParentAlive

ENDLOOP
System preset value PRELOAD_DN036_HowManyBrothersAlive
System preset value PRELOAD_DN037_HowManySistersAlive
LOOP := 1 TO 14

System preset value PRELOAD_PH006_DocCon

ENDLOOP
LOOP := 1 TO 14

System preset value PRELOAD_PH067_HadCondition

ENDLOOP
System preset value PRELOAD_BR002_StillSmoking
LOOP := 1 TO 20

BLOCK

System preset value Kidcom
System preset value Name
System preset value Name_UTF8
System preset value Gender
System preset value Year
System preset value Relation
System preset value Distance
System preset value SN_id

ENDBLOCK

ENDLOOP
LOOP := 1 TO 7

BLOCK

System preset value Name
System preset value Name_UTF8
System preset value Gender
System preset value Relation

ENDBLOCK

ENDLOOP
LOOP := 1 TO 20

BLOCK

System preset value Kidcom
System preset value Name
System preset value Name_UTF8
System preset value Gender
System preset value Year
System preset value Relation
System preset value Distance
System preset value SN_id

ENDBLOCK

ENDLOOP
System preset value PreloadDefineNr
System preset value RosterDefineNr
System preset value PartnerChildrenDefineNr

ENDBLOCK
System preset value MN021_Version
IF MN021_Version = EMPTY
ENDIF
System preset value MN001_Country
IF MN001_Country = EMPTY
ENDIF
System preset value MN004_EuroCountry
System preset value MN005_ModeQues
System preset value MN006_NumFamR
System preset value MN007_NumFinR
System preset value MN008_NumHHR
System preset value MN010_TestMode
System preset value MN011_StartTime
System preset value MN012_StartDate
System preset value MN013_HHSize
System preset value MN014_NumberEligibles
System preset value MN015_Eligibles
System preset value FLEligibles
System preset value MN016_MotherinHH
System preset value MN017_FatherinHH
System preset value MN018_MotherinLawinHH
System preset value MN019_FatherinLawinHH
System preset value MN020_RandomEX023
IF MN011_StartTime = EMPTY
ENDIF
IF MN020_RandomEX023 = EMPTY
ENDIF
System preset value MN022_SampleType
System preset value MN023_PersonsOver17
System preset value MN024_NursingHome
System preset value MN025_RandomCF102
System preset value MN026_FirstResp
System preset value MN027_CVResp
System preset value MN028_bio
System preset value MN029_linkage
System preset value MN030_socnet
System preset value MN031_michi
System preset value MN032_socex
System preset value MN033_language
System preset value MN034_hasmoved
System preset value MN035_Random_PH004

System preset value index
LOOP cnt:= 1 TO 20

System preset value FLChild

ENDLOOP
LOOP cnt:= 1 TO 20

System preset value PreloadChild

ENDLOOP
LOOP cnt:= 1 TO 7

System preset value FLRoster

ENDLOOP
LOOP cnt:= 1 TO 7

System preset value FLSocialNetwork

ENDLOOP
BLOCK

System preset value Name
System preset value Name_UTF8
System preset value Age
System preset value Gender
System preset value MaritalStatus
System preset value Month
System preset value Year
System preset value RespId

ENDBLOCK
BLOCK

System preset value Name
System preset value Name_UTF8
System preset value Age
System preset value Gender
System preset value MaritalStatus
System preset value Month
System preset value Year
System preset value RespId

*ENDBLOCK*TxT_Defaults1
TxT_Errors
Txt_FLDayMonthYear
Txt_FLInitCountry
Txt_FL200_5000
Txt_MonthYear
InitEuro

IF (MN010_TestMode = a1) AND (MN001_Country = a1)

Test
Which sections do you want to test?

TO BE FILLED IN ONLY FOR TESTING

2. DN
3. SN
4. CH
5. PH
6. BR

7. CF
8. MH
9. HC
10. LI
11. EP
12. IT
13. GS
14. BS
15. PF
16. SP
17. FT
18. HO
19. HH
20. CO
21. AS
22. AC
24. EX
25. IV
26. All

Intro2
Now the actual interview starts.

TO BE FILLED IN ONLY FOR TESTING

1. Continue

ELSE
ENDIF
BLOCK

IF ((((MN001_Country = a11) OR (MN001_Country = a14)) OR (MN001_Country = a19)) OR (MN001_Country = a21)) OR (MN001_Country = a23)

CM601_Language

IWER:
Please choose the language you want to use during the interview. If unsure ask the respondent.
CHOOSE LANGUAGE
English (Generic)
MEDIA

ENDIF
IF piMode = a1
ELSE

IF piMode = a2

CM003_RespFin
Later in this interview, we will be asking questions about household and family finances, for example about your savings for old-age and financial support to children and other relatives. We only need to ask these questions of one of you. Which of you would be the one most able to answer questions about your finances?

IWER:
Code one only financial respondent
CHOICE RESPONDENT FINANCE
1. ^MN002_Person[1].Name
2. ^MN002_Person[2].Name

IF piLongitudinal = 0
ELSE
ENDIF

ELSE

IF piMode = a3
ENDIF

ENDIF

ENDIF

ENDBLOCK
IF (DN IN Test) OR (ALL IN Test)

BLOCK

IF (MN027_CVResp = 1) OR (MN101_Longitudinal = 0)
Txt_FL_DN001

DN001_Intro
Let me just repeat that this interview is confidential. Your answers will be used only for research purposes. If we should come to any question you don't want to answer, just let me know and I will go on to the next question. Now I would like to begin by asking some questions about your background.
INTRO DEMOGRAPHICS
1. Continue

ELSE
Txt_FL_DN001a

DN001a_Intro
Let me just repeat that this interview is confidential. Your answers will be used only for research purposes. If we should come to any question you don't want to answer, just let me know and I will go on to the next question. During our previous interview we asked you about your life. To shorten our interview today, I would like to refer to your previous answers instead of asking everything again. Would that be ok?
INTRO DEMOGRAPHICS A
1. Yes
5. No

IF DN001a_Intro = a5
*ENDIF*Txt_FL_DN001b

DN001b_Intro
I would like to begin by asking some questions about your background.
INTRO DEMOGRAPHICS B
1. Continue

ENDIF
System preset value DN901_TimeStampStart
IF DN901_TimeStampStart = EMPTY AND (DN001_Intro <> EMPTY OR DN001a_Intro <> EMPTY)
ENDIF
BLOCK

DN042_Gender
OBSERVATION

IWER:
Note sex of respondent from observation (ask if unsure)
MALE OR FEMALE
1. Male

2. Female

Txt_Month
Txt_Year
Txt_FL_DN043

DN043_BirthConf
Can I just confirm? You were born in [FLDefault[10]/FLDefault[11]/FLDefault[12]/FLDefault[13]/FLDefault[14]/FLDefault[15]/FLDefault[16]/FLDefault[17]/FLDefault[18]/FLDefault[19]/FLDefault[20]/FLDefault[21]] [STR (piYear)]?
CONFIRM MONTH/YEAR BIRTH
1. Yes
5. No

IF DN043_BirthConf = a1
ELSE

IF DN043_BirthConf = a5
Txt_FL_DN002

DN002_MoBirth
In which @bmonth@b and year were you born?

@bMONTH@b:
YEAR:
MONTH OF BIRTH
1. January
2. February
3. March
4. April
5. May
6. June
7. July
8. August
9. September
10. October
11. November
12. December

Txt_FL_DN003

DN003_YearBirth
In which month and @byear@b were you born?

MONTH: {DN002_MoBirth}
@bYEAR@b:
YEAR OF BIRTH
1900..2015

IF DN003_YearBirth <> NONRESPONSE AND DN002_MoBirth <> NONRESPONSE
ENDIF

ELSE
ENDIF

ENDIF
IF MN101_Longitudinal = 0
Txt_FL_DN004

DN004_CountryOfBirth
Were you born in the United Kingdom?
COUNTRY OF BIRTH
1. Yes

5. No

IF [DN004_CountryOfBirth](#) = a5

Txt_FL_DN005

DN005_OtherCountry
In which country were you born? Please name the country that your birthplace belonged to at the time of your birth.
OTHER COUNTRY OF BIRTH
STRING

Txt_FL_DN006

DN006_YearToCountry
In which year did you come to live in the United Kingdom?
YEAR CAME TO LIVE IN COUNTRY
1875..2015

*ENDIF*Txt_FL_DN007

DN007_Citizenship
Do you have British citizenship?
CITIZENSHIP COUNTRY
1. Yes
5. No

IF [DN007_Citizenship](#) = a1

Txt_FL_DN503

DN503_NationalitySinceBirth
Were you born a citizen of Britain?
NATIONALTIY SINCE BIRTH
1. Yes
5. No

IF [DN503_NationalitySinceBirth](#) = a5

Txt_FL_DN502

DN502_WhenBecomeCitizen
In what year did you become a citizen of Britain?
WHEN CITIZEN
1900..2015

ENDIF

ELSE

IF [DN007_Citizenship](#) = a5

Txt_FL_DN008

DN008_OtherCitizenship
What is your citizenship?
OTHER CITIZENSHIP
STRING

ENDIF

ENDIF
IF ((((MN001_Country = a1) OR (MN001_Country = a3)) OR (MN001_Country = a8)) OR (MN001_Country = a19)) OR (MN001_Country = a22)

DN009_WhereLived

Where did you live on November 1st 1989, that is before the Berlin wall came down - Did you live in the GDR, in the FRG, or elsewhere?
WHERE LIVED SINCE 1989
1. GDR
2. FRG
3. Elsewhere

ENDIF

ENDIF
IF MN101_Longitudinal = 0
Txt_FL_DN504

DN504_CountryOfBirthMother
In which country was your mother born?
COUNTRY BIRTH MOTHER
STRING
Txt_FL_DN505

DN505_CountryOfBirthFather
In which country was your father born?
COUNTRY BIRTH FATHER
STRING
Txt_FL_DN010

DN010_HighestEdu
Please look at card 2.
What is the highest school leaving certificate or school degree that you have obtained?

IWER:
If respondent mentions foreign degree/certificate, please ask if he/she can fit their degree into the given categories, if they cannot, please use the other option and type it in (next screen).
HIGHEST EDUCATIONAL DEGREE OBTAINED
1. No schooling/education at all
2. Some education, but less than [instead of put respective country specific degr.]
3. Country specific category
4. Country specific category
5. Country specific category
6. Country specific category
7. Country specific category
8. Country specific category
9. Country specific category
10. Country specific category
11. Country specific category
12. Country specific category
13. Country specific category
14. Country specific category
15. Country specific category
16. Country specific category
17. Country specific category
18. Country specific category
19. Country specific category
20. Country specific category
95. No degree yet/still in school
97. Other

IF DN010_HighestEdu = a97
Txt_FL_DN011

DN011_OtherHighestEdu
What other school leaving certificate or school degree have you obtained?
OTHER HIGHEST EDUCATION

STRING

*ENDIF*Txt_FL_DN012

DN012_FurtherEdu
Please look at card 3.
Which degrees of higher education or vocational training do you have?

IWER:
{CodeAll}
If respondent answers 'still in education/vocational training' please ask if he/she already holds one of the other degrees on the showcard.
FURTHER EDUCATION
1. No higher education/vocational training
2. Some education, but less than [ISCED 1] (instead of [ISCED 1] put respective country specific degr.)
3. Country specific category
4. Country specific category
5. Country specific category
6. Country specific category
7. Country specific category
8. Country specific category
9. Country specific category
10. Country specific category
11. Country specific category
12. Country specific category
13. Country specific category
14. Country specific category
15. Country specific category
16. Country specific category
17. Country specific category
18. Country specific category
19. Country specific category
20. Country specific category
95. Still in education/vocational training
97. Other

IF 97.00000000000001 IN DN012_FurtherEdu
Txt_FL_DN013

DN013_WhichOtherEdu
Which other degree of higher education or vocational training do you have?
OTHER EDUCATION
STRING

*ENDIF*Txt_FL_DN041

DN041_YearsEdu
How many years have you been in full-time education?

IWER:
full-time education
** includes: receiving tuition, engaging in practical work or supervised study or taking examinations*
** excludes: full-time working, home schooling, distance learning, special on-the-job training, evening classes, part-time private vocational training, flexible or part-time higher education studies, etc*
YEARS EDUCATION
0..25

ELSE

IF MN101_Longitudinal = 1
Txt_FL_DN044

DN044_MaritalStatus

Since our last interview, has your marital status changed?
MARITAL STATUS CHANGED
1. Yes, marital status has changed
5. No, marital status has not changed

ENDIF

ENDIF
IF ((MN101_Longitudinal = 1) AND (DN044_MaritalStatus = a1)) OR (MN101_Longitudinal = 0)
Txt_FL_DN014

DN014_MaritalStatus
Please look at card 4.
What is your marital status?

IWER:
If marriage persists but partner does not live in household for any reason, such as being in a nursing home, hospital, prison etc., then code 3.
MARITAL STATUS
1. Married and living together with spouse
2. Registered partnership
3. Married, living separated from spouse
4. Never married
5. Divorced
6. Widowed

IF DN014_MaritalStatus = a1

IF MN026_FirstResp = 1
Txt_FL_DN015

DN015_YearOfMarriage
In which year did you get married?
YEAR OF MARRIAGE
1905..2015
CHECK (CURRENTDATE.YEAR - DN015_YearOfMarriage) < (MN808_AgeRespondent - 12) L1 " [Year marriage should be at least 12 years after year of birth of respondent! If year is correct, please press "suppress" and enter a remark to explain]"

ENDIF

ELSE

IF DN014_MaritalStatus = a2
Txt_FL_DN016

DN016_YearOfPartnership
In which year did you register your partnership?
YEAR of REGISTERED PARTNERSHIP
1905..2015

ELSE

IF DN014_MaritalStatus = a3
Txt_FL_DN017

DN017_YearOfMarriage
In which year did you get married?
YEAR OF MARRIAGE
1905..2015

ELSE

IF DN014_MaritalStatus = a5

Txt_FL_DN018

DN018_DivorcedSinceWhen

In which year did you get divorced?

IWER:
If more than one divorce enter year of last divorce
SINCE WHEN DIVORCED
1905..2015

ELSE

IF DN014_MaritalStatus = a6

Txt_FL_DN019

DN019_WidowedSinceWhen

In which year did you become a [widow/ widower]?

IWER:
Enter year of death of partner
SINCE WHEN WIDOWED
1914..2015

ENDIF

ENDIF

ENDIF

ENDIF

ENDIF
IF (MN101_Longitudinal = 0) AND (((DN014_MaritalStatus = a3) OR (DN014_MaritalStatus = a5)) OR (DN014_MaritalStatus = a6))

Txt_FL_DN020

DN020_AgePart

In which year was [your/ your/ your/ your/ your/ your] [ex-/ ex-/ late/ late] [husband/ wife/ husband/ wife/ husband/ wife] born?

IWER:
Record birthyear of most recent spouse
AGE OF PARTNER
1895..1999

Txt_FL_DN021

DN021_HighestEduPart

Please look at card 2.
What is the highest school certificate or degree that [your/ your/ your/ your/ your/ your] [ex-/ vex-/ late/ late] [husband/ wife/ husband/ wife/ husband/ wife] has obtained?

IWER:
If respondent mentions foreign degree/certificate, please ask if he/she can fit their degree into the given categories, if they cannot, please use the other option and type it in (next screen).
HIGHEST EDUCATIONAL DEGREE OF PARTNER
1. No schooling/education at all
2. Some education, but less than [instead of put respective country specific degr.]
3. Country specific category
4. Country specific category
5. Country specific category
6. Country specific category
7. Country specific category

8. Country specific category
9. Country specific category
10. Country specific category
11. Country specific category
12. Country specific category
13. Country specific category
14. Country specific category
15. Country specific category
16. Country specific category
17. Country specific category
18. Country specific category
19. Country specific category
20. Country specific category
95. No degree yet/still in school
97. Other

IF DN021_HighestEduPart = a97

Txt_FL_DN022

DN022_OtherHighestEduPart

Which other school certificate or degree has [your/ your/ your/ your/ your/ your] [ex-/ ex-/ late/ late] [husband/ wife/ husband/ wife/ husband/ wife] obtained?

OTHER HIGHEST EDUCATIONAL DEGREE PARTNER OBTAINED

STRING

*ENDIF*Txt_FL_DN023

DN023_FurtherEduPart

Please look at card 3.

Which degrees of higher education or vocational training does [your/ your/ your/ your/ your/ your] [ex-/ ex-/ late/ late] [husband/ wife/ husband/ wife/ husband/ wife] have?

IWER:

{CodeAll}

FURTHER EDUCATION OR VOCATIONAL TRAINING OBTAINED OF PARTNER

1. No higher education/vocational training
2. Some education, but less than [ISCED 1] (instead of [ISCED 1] put respective country specific degr.)
3. Country specific category
4. Country specific category
5. Country specific category
6. Country specific category
7. Country specific category
8. Country specific category
9. Country specific category
10. Country specific category
11. Country specific category
12. Country specific category
13. Country specific category
14. Country specific category
15. Country specific category
16. Country specific category
17. Country specific category
18. Country specific category
19. Country specific category
20. Country specific category
95. Still in education/vocational training
97. Other

IF 97.00000000000001 IN DN023_FurtherEduPart

Txt_FL_DN024

DN024_WhichOtherEduPart

Which other higher education or vocational training does [your/ your/ your/ your/ your/ your] [ex-/ ex-/ late/ late] [husband/ wife/ husband/ wife/ husband/ wife] have?
OTHER EDUCATION PARTNER
STRING

ENDIF

ENDIF

ELSE
ENDIF
IF MN005_ModeQues = a1
Txt_FL_DN040

DN040_PartnerOutsideHH
Do you have a partner who lives outside this household?
PARTNER OUTSIDE HOUSEHOLD
1. Yes
5. No

ENDIF

ENDBLOCK

DN038_IntCheck

IWER:
CHECK: Who answered the questions in this section?
INTERVIEWER CHECK DN
1. Respondent only
2. Respondent and proxy
3. Proxy only

System preset value DN902_TimeStampEnd
IF DN902_TimeStampEnd = EMPTY AND DN038_IntCheck <> EMPTY
ENDIF

ENDBLOCK

ENDIF
IF (SN IN Test) OR (ALL IN Test)

IF MN030_socnet = 1

BLOCK

SN014_Privacy

IWER:
The following set of questions should be answered by the respondent in private. If there are any other people in the room at this point, please remind them that parts of the interview are of a private nature and should be answered by each respondent on his or her own.
Start of a @BNon-proxy section@B. If the respondent is not capable of answering any of these questions on her/his own, please select '5. Proxy-interview'.
INTRODUCTION PRIVACY SN
1. No need to explain, respondent is interviewed in private (and will answer this section).
2. Explained private nature of the interview to third people, left the room (respondent will answer this section).
3. Explained private nature of the interview to third people, did not leave the room (respondent will answer this section).
5. Proxy-interview

System preset value SN901_TimeStampStart
IF SN901_TimeStampStart = EMPTY AND SN014_Privacy <> EMPTY
ENDIF
IF NOT (SN014_Privacy = a5) AND (SN014_Privacy = RESPONSE)

Txt_FL_SN001

SN001_Introduction
Now I am going to ask some questions about your relationships with other people. Most people discuss with others the good or bad things that happen to them, problems they are having, or important concerns they may have. Looking back over the last 12 months, who are the people with whom you most often discussed important things? These people may include your family members, friends, neighbors, or other acquaintances. Please refer to these people by their first names.
INTRODUCTION SN
1. Continue

IF SN001_Introduction = REFUSAL
ELSE

LOOP cnt:= 1 TO 6

IF (cnt > 1) AND (SN_Roster[cnt - 1].SN002a_NoMore = a5)
ELSE

BLOCK

IF piIndex = 7
ELSE

IF piIndex = 1
ELSE

Txt_FL_SN002a

SN002a_NoMore
Are there any other people (with whom you often discuss things that are important to you)?

IWER:
Click '1. Yes' immediately when it is obvious there are others
Any more
1. Yes
5. No

ENDIF

ENDIF
IF SN002a_NoMore = a1

IF piIndex = 7
ELSE

Txt_FL_SN002

SN002_Roster
Please give me the first name of the person with whom you [MOST OFTEN/ often] discuss things that are important to you:

IWER:
[if R cannot name any network member, type 991]
FIRST NAME OF ROSTER N
STRING

ENDIF

IF ((SN002_Roster = REFUSAL) OR (SN002_Roster = DONTKNOW)) OR (SN002_Roster = '991')
ELSE

IF SN002_Roster = RESPONSE
ENDIFTxt_FL_SN005

SN005_NetworkRelationship
What is {SN002_Roster}'s relationship to you?

IWER:
Prompt if needed: so this person is your...
NETWORK RELATIONSHIP
1. Spouse/Partner
2. Mother
3. Father
4. Mother-in-law
5. Father-in-law
6. Stepmother
7. Stepfather
8. Brother
9. Sister
10. Child
11. Step-child/your current partner's child
12. Son-in-law
13. Daughter-in-law
14. Grandchild
15. Grandparent
16. Aunt
17. Uncle
18. Niece
19. Nephew
20. Other relative
21. Friend
22. (Ex-)colleague/co-worker
23. Neighbour
24. Ex-spouse/partner
25. Minister, priest, or other clergy
26. Therapist or other professional helper
27. Housekeeper/Home health care provider
96. None of these

IF SN005_NetworkRelationship = RESPONSE

IF SN005_NetworkRelationship <> a96
getRelationShip

IF NOT (RelationshipString = '') AND SN002_Roster <> EMPTY
ENDIF
IF SN005_NetworkRelationship = a2
ELSE

IF SN005_NetworkRelationship = a3
ELSE

IF (SN005_NetworkRelationship = a8) OR (SN005_NetworkRelationship = a9)
ELSE

IF (SN005_NetworkRelationship = a10) OR (SN005_NetworkRelationship = a11)
ENDIF

ENDIF

ENDIF

ENDIF

ENDIF

ENDIF

ENDIF

ENDIF

ENDBLOCK

ENDIF
IF SN_Roster[cnt].SN002a_NoMore = a1
ENDIF

*ENDLOOP*Txt_FL_SN003a

SN003a_AnyoneElse
Is there anyone (else) who is very important to you for some other reason?
ANY MORE
1. Yes
5. No

IF SN003a_AnyoneElse = a1
Txt_FL_SN003

SN003_AnyoneElse
Please give me the first name of a person who is important to you for some other reason.
FIRST NAME OF ROSTER 7
STRING

IF SN003_AnyoneElse = RESPONSE

BLOCK

IF piIndex = 7
ELSE

IF piIndex = 1
ELSE
Txt_FL_SN002a

SN002a_NoMore
Are there any other people (with whom you often discuss things that are important to you)?

IWER:
Click '1. Yes' immediately when it is obvious there are others
Any more
1. Yes
5. No

ENDIF

ENDIF
IF SN002a_NoMore = a1

IF piIndex = 7
ELSE
Txt_FL_SN002

SN002_Roster
Please give me the first name of the person with whom you [MOST OFTEN/ often] discuss things that are important to you:

IWER:
[if R cannot name any network member, type 991]
FIRST NAME OF ROSTER N
STRING

ENDIF
IF ((SN002_Roster = REFUSAL) OR (SN002_Roster = DONTKNOW)) OR (SN002_Roster = '991')
ELSE

IF SN002_Roster = RESPONSE
*ENDIF*Txt_FL_SN005

SN005_NetworkRelationship
What is {SN002_Roster}'s relationship to you?

IWER:
Prompt if needed: so this person is your...
NETWORK RELATIONSHIP
1. Spouse/Partner
2. Mother
3. Father
4. Mother-in-law
5. Father-in-law
6. Stepmother
7. Stepfather
8. Brother
9. Sister
10. Child
11. Step-child/your current partner's child
12. Son-in-law
13. Daughter-in-law
14. Grandchild
15. Grandparent
16. Aunt
17. Uncle
18. Niece
19. Nephew
20. Other relative
21. Friend
22. (Ex-)colleague/co-worker
23. Neighbour
24. Ex-spouse/partner
25. Minister, priest, or other clergy
26. Therapist or other professional helper
27. Housekeeper/Home health care provider
96. None of these

IF SN005_NetworkRelationship = RESPONSE

IF SN005_NetworkRelationship <> a96
getRelationShip

IF NOT (RelationshipString = '') AND SN002_Roster <> EMPTY
ENDIF
IF SN005_NetworkRelationship = a2
ELSE

IF SN005_NetworkRelationship = a3
ELSE

IF (SN005_NetworkRelationship = a8) OR (SN005_NetworkRelationship = a9)
ELSE

IF (SN005_NetworkRelationship = a10) OR (SN005_NetworkRelationship = a11)
ENDIF

ENDIF

ENDIF

ENDIF

ENDIF

ENDIF

ENDIF

ENDIF

ENDBLOCK
IF SN_Roster[7].SN002a_NoMore = a1
ENDIF

ENDIF

ENDIF
System preset value Sizeofsocialnetwork
LOOP cnt:= 1 TO 7

IF NOT ((SN_Roster[cnt].SN002_Roster = '') OR (SN_Roster[cnt].SN002_Roster = '991'))

IF (SN_Roster[cnt].SN005_NetworkRelationship = a10) OR (SN_Roster[cnt].SN005_NetworkRelationship = a11)
*ENDIF*getRelationShip

ENDIF

ENDLOOP
IF Sizeofsocialnetwork > 0
Txt_FL_SN008

SN008_Intro_closeness
Now I would like to ask a few more questions about the people who are close to you
INTRODUCTION CLOSENESS
1. Continue

LOOP cnt:= 1 TO 7

BLOCK
getRelationShip

IF FLRosterName <> ''

IF (FLRosterRelation = a10) OR (FLRosterRelation = a11)

IF MN006_NumFamR <> 1

LOOP i:= 1 TO 20

IF NOT ((Preload.PartnerChildren[i].Name = '') OR Preload.PartnerChildren[i].Name = EMPTY)

IF Preload.PartnerChildren[i].Gender = a1
ELSE

IF Preload.PartnerChildren[i].Gender = a2
ENDIF

ENDIFTxt_SET_STRING

ENDIF

ENDLOOP
IF num_of_preloadchildren > 0
Txt_FL_SN018

SN018_PreloadMatch
You just mentioned your child {FLRosterName}. I would like to confirm if this child was mentioned by your partner or in a previous interview.

IWER:
Tick the child if available in the list
LINK TO PRELOAD CHILD
^PreloadChild[1]
^PreloadChild[2]
^PreloadChild[3]
^PreloadChild[4]
^PreloadChild[5]
^PreloadChild[6]
^PreloadChild[7]
^PreloadChild[8]
^PreloadChild[9]
^PreloadChild[10]
^PreloadChild[11]
^PreloadChild[12]
^PreloadChild[13]
^PreloadChild[14]
^PreloadChild[15]
^PreloadChild[16]
^PreloadChild[17]
^PreloadChild[18]
^PreloadChild[19]
^PreloadChild[20]
96. ^FLDefault[79]

IF (SN018_PreloadMatch = RESPONSE) AND (SN018_PreloadMatch <> a96)
ELSE
Txt_FL_SN005a

SN005a_Gender

IWER:
Code sex of {FLRosterName} {localRelationText}
NETWORK PERSON GENDER
1. Male
2. Female

ENDIF

ELSE
Txt_FL_SN005a

SN005a_Gender

IWER:
Code sex of {FLRosterName} {localRelationText}
NETWORK PERSON GENDER
1. Male
2. Female

ENDIF

ELSE
Txt_FL_SN006

SN006_NetworkProximity
Please look at card 5
Where does {FLRosterName} {localRelationText} live?
NETWORK Proximity
1. In the same household
2. In the same building
3. Less than 1 kilometre away
4. Between 1 and 5 kilometres away
5. Between 5 and 25 kilometres away
6. Between 25 and 100 kilometres away
7. Between 100 and 500 kilometres away
8. More than 500 kilometres away

ENDIF

ELSE

IF (FLRosterRelation = a1) AND ((MN002_Person[1].MaritalStatus = a1) OR (MN002_Person[1].MaritalStatus = a2))
ELSE

IF ((((((FLRosterRelation = a3) OR (FLRosterRelation = a5)) OR (FLRosterRelation = a7)) OR (FLRosterRelation = a8)) OR (FLRosterRelation = a12)) OR (FLRosterRelation = a17)) OR (FLRosterRelation = a19)
ELSE

IF ((((((FLRosterRelation = a2) OR (FLRosterRelation = a4)) OR (FLRosterRelation = a6)) OR (FLRosterRelation = a9)) OR (FLRosterRelation = a13)) OR (FLRosterRelation = a16)) OR (FLRosterRelation = a18)
ELSE
Txt_FL_SN005a

SN005a_Gender

IWER:
Code sex of {FLRosterName} {localRelationText}
NETWORK PERSON GENDER
1. Male
2. Female

ENDIF

ENDIF
IF NOT ((FLRosterRelation = a2) OR (FLRosterRelation = a3))
Txt_FL_SN006

SN006_NetworkProximity

Please look at card 5
Where does {FLRosterName} {localRelationText} live?
NETWORK Proximity
1. In the same household
2. In the same building
3. Less than 1 kilometre away
4. Between 1 and 5 kilometres away
5. Between 5 and 25 kilometres away
6. Between 25 and 100 kilometres away
7. Between 100 and 500 kilometres away
8. More than 500 kilometres away

ENDIF

ENDIF

ENDIF
IF NOT ((FLRosterRelation = a2) OR (FLRosterRelation = a3))

IF NOT (SN006_NetworkProximity = a1)
Txt_FL_SN007

SN007_NetworkContact
During the past twelve months, how often did you have contact with {FLRosterName} {localRelationText} either in person, by phone or mail, email or any other electronic means?
NETWORK CONTACT
1. Daily
2. Several times a week
3. About once a week
4. About every two weeks
5. About once a month
6. Less than once a month
7. Never

ENDIF

*ENDIF*Txt_FL_SN009

SN009_Network_Closeness
How close do you feel to {FLRosterName} {localRelationText}?

IWER:
{ReadOut}
Network Closeness
1. Not very close
2. Somewhat close
3. Very close
4. Extremely close

IF NOT (((FLRosterRelation = a10) OR (FLRosterRelation = a11)) OR (FLRosterRelation = a1))

SN027_YearOfBirthSNMember
In which year was {FLRosterName} {localRelationText} born?

IWER:
If respondent does not know the exact year of birth, ask for an estimate
YEAR OF BIRTH SN MEMBER
1875..2015

SN028_SNOcc
Please look at card 6.

What is {FLRosterName}'s employment status?
SN MEMBER OCCUPATION
1. Full-time employed
2. Part-time employed
3. Self-employed or working for own family business
4. Unemployed
5. In vocational training/retraining/education
6. Parental leave
7. In retirement or early retirement
8. Permanently sick or disabled
9. Looking after home or family
97. Other

SN029_RelationshipStatus
What is {FLRosterName}'s relationship status?

IWER:
{ReadOut}
RELATIONSHIP STATUS
1. No partner
2. Living with a partner
3. Has a partner but not living with him/her

ELSE

IF ((FLRosterRelation = a10) OR (FLRosterRelation = a11)) AND (MN006_NumFamR <> 1)

IF (SN018_PreloadMatch = RESPONSE) AND (SN018_PreloadMatch <> a96)
ELSE

SN027_YearOfBirthSNMember
In which year was {FLRosterName} {localRelationText} born?

IWER:
If respondent does not know the exact year of birth, ask for an estimate
YEAR OF BIRTH SN MEMBER
1875..2015

ENDIF

ENDIF

ENDIF

ENDIF

ENDBLOCK

ENDLOOP

ENDIF
IF Sec_SN.SN906_ChildInSocialNetwork > 0

LOOP cnt:= 1 TO 7

IF (SocialNetwork[cnt].SN005_NetworkRelationship = a10) OR (SocialNetwork[cnt].SN005_NetworkRelationship = a11)
ELSE
ENDIF

ENDLOOP

ENDIF
IF Sizeofsocialnetwork = 0
Txt_FL_SN017

SN017_Network_Satisfaction
You indicated that there is no one with whom you discuss important matters, and no one who is important to you for some other reason. On a scale from 0-10, where 0 means completely dissatisfied and 10 means completely satisfied, how satisfied are you with this (situation)?
EMPTY NETWORK SATISFACTION
0..10

ELSE
Txt_FL_SN012

SN012_Network_Satisfaction
Overall, on a scale from 0 to 10, where 0 means completely dissatisfied and 10 means completely satisfied, how satisfied are you with the [relationship that you have with the person/ relationships that you have with all the people] we have just talked about?
NETWORK SATISFACTION
0..10

ENDIF

ENDIF
LOOP cnt:= 1 TO 7

IF NOT (Preload.PreloadedSocialNetwork[cnt].Name = '')
getRelationShip

IF TempRelationshipString = ''
ELSE
ENDIF

ENDIF

ENDLOOP

LinkOldRoster

:*TABLE Section_SN.BLinking*Txt_MonthYear

LOOP X:= 1 TO 7

IF pName[X] <> EMPTY AND (pName[X] <> '')

BLOCK

LOOP Y:= 1 TO 7

IF FL_Unmatched_NEW_SN[Y] <> ''
ELSE
ENDIF

*ENDLOOP*Txt_MonthYear

IF NOT (((((((FL_Unmatched_NEW_SN_ANSWER[1] = '') AND (FL_Unmatched_NEW_SN_ANSWER[2] = '')) AND (FL_Unmatched_NEW_SN_ANSWER[3] = '')) AND (FL_Unmatched_NEW_SN_ANSWER[4] = '')) AND (FL_Unmatched_NEW_SN_ANSWER[5] = '')) AND (FL_Unmatched_NEW_SN_ANSWER[6] = '')) AND (FL_Unmatched_NEW_SN_ANSWER[7] = ''))
TXT_THIS_INTERVIEW

THIS_INTERVIEW

[(' As you may remember, when we interviewed you in ' + piIDate) + ', you also mentioned some people that were important to you at that time.'/(' As you may remember, when we interviewed you in ' + piIDate) + ', you also mentioned some people that were important to you at that time.'] [Now we would like to compare those persons to the ones you just mentioned today to find out who you mentioned again and who not./ Now we would like to compare those persons to the ones you just mentioned today to find out who you mentioned again and who not.]

Last time you mentioned {piName} {piRelation}. Did you mention him/her again today?

IWER:
If respondent confirms that {piName} was mentioned today, check FIRST list below for {piName} and enter the corresponding number.
If {piName} was not mentioned today, enter 96 (Person not mentioned again this time).

Persons mentioned this time:
Link to
^FL_Unmatched_NEW_SN_ANSWER[1]
^FL_Unmatched_NEW_SN_ANSWER[2]
^FL_Unmatched_NEW_SN_ANSWER[3]
^FL_Unmatched_NEW_SN_ANSWER[4]
^FL_Unmatched_NEW_SN_ANSWER[5]
^FL_Unmatched_NEW_SN_ANSWER[6]
^FL_Unmatched_NEW_SN_ANSWER[7]
96. ^FLDefault[81]

IF (THIS_INTERVIEW = RESPONSE) AND (THIS_INTERVIEW <> a96)
ELSE

IF THIS_INTERVIEW = a96

Txt_FL_SN023

SN023_whathappnd
[(' As you may remember, when we interviewed you in ' + piDate) + ', you mentioned some people that were important to you at that time.@/'] [(((' Last time you mentioned ' + piName) + ' (') + piRelation) + ').@/'] What is the main reason you didn't mention {piName} this time?

1. I forgot, ^piName should have been included
2. I moved
3. ^piName moved
4. ^piName died
5. I became ill or had a health problem
6. ^piName became ill or had a health problem
7. Respondent does not recognize the named person
8. We are no longer close
9. Wrong, ^piName WAS mentioned this time
97. Other reason (specify)

CHECK NOT ((SN023_whathappnd = a9) AND (THIS_INTERVIEW = a96)) L1 " {FLError[44]}"

IF SN023_whathappnd = a97

SN024_Other
What other reason?
OTHER REASON FOR NOT MENTIONING IN SOCIAL NETWORK
STRING

ENDIF

ENDIF

ENDIF

ELSE

Txt_FL_SN023

SN023_whathappnd
[(' As you may remember, when we interviewed you in ' + piDate) + ', you mentioned some people that were important to you at that time.@/'] [(((' Last time you mentioned ' + piName) + ' (') + piRelation) + ').@/'] What is the main reason you didn't mention {piName} this time?

1. I forgot, ^piName should have been included
2. I moved
3. ^piName moved
4. ^piName died
5. I became ill or had a health problem
6. ^piName became ill or had a health problem
7. Respondent does not recognize the named person
8. We are no longer close
9. Wrong, ^piName WAS mentioned this time
97. Other reason (specify)

IF SN023_whathappnd = a97

SN024_Other
What other reason?
OTHER REASON FOR NOT MENTIONING IN SOCIAL NETWORK
STRING

ENDIF

ENDIF

ENDBLOCK

ENDIF

*ENDLOOP*ENDTABLE

SN015_Who_present

IWER:
Check who was present during this section.
{CodeAll}
WHO WAS PRESENT
1. Respondent alone
2. Partner present
3. Child(ren) present
4. Other(s)

CHECK NOT ((SN015_Who_present.CARDINAL > 1) AND (a1 IN SN015_Who_present)) L1 " [Cannot select -respondent alone- with any other category]"

ENDIF
System preset value SN902_TimeStampEnd
IF SN902_TimeStampEnd = EMPTY
ENDIF

ENDBLOCK

ENDIF

ENDIF
IF (DN IN Test) OR (ALL IN Test)

BLOCK

System preset value DN901_TimeStampStart
IF DN901_TimeStampStart = EMPTY
ENDIF
BLOCK

IF (Preload.PRELOAD_DN026_NaturalParentAlive[1] <> a5) OR (Sec_SN.SN903_FatherInSocialNetwork = 1)

BLOCK

IF piParentAlive = 1

IF ((piIndex = 1) AND (Sec_SN.SN904_MotherInSocialNetwork = 1)) OR ((piIndex = 2) AND (Sec_SN.SN903_FatherInSocialNetwork = 1))
ELSE

IF (piIndex = 1) OR (piIndex = 2)
Txt_FL_DN026

DN026_NaturalParentAlive
Is [your/ your] [natural/ natural] [mother/ father] still alive?
IS NATURAL PARENT STILL ALIVE
1. Yes
5. No

IF DN026_NaturalParentAlive = a5
Txt_FL_DN127

DN127_YearOfDeathParent
[You told us in a previous interview that your mother is not alive anymore./ You told us in a previous interview that your father is not alive anymore.] In what year did [your/ your] [mother/ father] die?
AGE OF DEATH OF PARENT
1800..2015

ENDIF
IF DN026_NaturalParentAlive = a5
Txt_FL_DN027

DN027_AgeOfDeathParent
How old was [your/ your] [mother/ father] when [she/ he] died?
AGE OF DEATH OF PARENT
10..120

ELSE

IF (DN026_NaturalParentAlive = a1) AND (MN101_Longitudinal = 0)
Txt_FL_DN028

DN028_AgeOfNaturalParent
How old is [your/ your] [mother/ father] now?
AGE OF NATURAL PARENT
40..120
CHECK DN028_AgeOfNaturalParent >= (MN808_AgeRespondent + 10) L1 " [Age should be at least ten years above respondent""s age. If age is correct, please press "suppress" and enter a remark to explain]"

ENDIF

ENDIF

ELSE

IF MN101_Longitudinal = 0

Txt_FL_DN028

DN028_AgeOfNaturalParent
How old is [your/ your] [mother/ father] now?
AGE OF NATURAL PARENT
40..120
CHECK DN028_AgeOfNaturalParent >= (MN808_AgeRespondent + 10) L1 " [Age should be at least ten years above respondent""s age. If age is correct, please press "suppress" and enter a remark to explain]"

ENDIF

ENDIF

ENDIF

ELSE
Txt_FL_DN127

DN127_YearOfDeathParent
[You told us in a previous interview that your mother is not alive anymore./ You told us in a previous interview that your father is not alive anymore.] In what year did [your/ your] [mother/ father] die?
AGE OF DEATH OF PARENT
1800..2015

ENDIF
IF MN101_Longitudinal = 0
Txt_FL_DN629

DN629_JobSitParent10
Please look at card 7. In general, which of the following best describes [your/ your] [mother/ father] employment situation had when you were about 10 years old?
NAME OR TITLE OF JOB OF PARENT
1. Retired
2. Employed or self-employed (including working for family business)
3. Unemployed
4. Permanently sick or disabled
5. Homemaker
97. Other

IF DN629_JobSitParent10 = a2
Txt_FL_DN029

DN029_JobOfParent10
What was the job [your/ your] [mother/ father] had when you were about 10 years old? Please give the exact name or title.
NAME OR TITLE OF JOB OF PARENT
STRING

IF DN029_JobOfParent10 = RESPONSE

BLOCK

JobCode

STRING

ENDBLOCK

ENDIF

*ENDIF*Txt_FL_DN051

DN051_HighestEduParent
Please look at card 2. What is the highest school certificate or degree that [your/ your] [mother/ father] has obtained?

IWER:
If respondent mentions foreign degree/certificate, please ask if he/she can fit their degree into the given categories, if they cannot, please use the other option and type it in (next screen).
HIGHEST EDUCATIONAL DEGREE OF PARENT
1. No schooling/education at all
2. Some education, but less than [instead of put respective country specific degr.]
3. Country specific category
4. Country specific category
5. Country specific category
6. Country specific category
7. Country specific category
8. Country specific category
9. Country specific category
10. Country specific category
11. Country specific category
12. Country specific category
13. Country specific category
14. Country specific category
15. Country specific category
16. Country specific category
17. Country specific category
18. Country specific category
19. Country specific category
20. Country specific category
95. No degree yet/still in school
97. Other

IF DN051_HighestEduParent = a97
Txt_FL_DN052

DN052_OtherHighestEduParent
Which other school certificate or degree has [your/ your] [mother/ father] obtained?
OTHER HIGHEST EDUCATION PARENT
STRING

*ENDIF*Txt_FL_DN053

DN053_FurtherEduParent
Please look at card 3. Which degrees of higher education or vocational training does [your/ your] [mother/ father] have?

IWER:
{CodeAll}
FURTHER EDUCATION OR VOCATIONAL TRAINING PARENT
1. No higher education/vocational training
2. Some education, but less than [ISCED 1] (instead of [ISCED 1] put respective country specific degr.)
3. Country specific category
4. Country specific category
5. Country specific category
6. Country specific category
7. Country specific category
8. Country specific category
9. Country specific category
10. Country specific category
11. Country specific category
12. Country specific category
13. Country specific category
14. Country specific category
15. Country specific category

16. Country specific category
17. Country specific category
18. Country specific category
19. Country specific category
20. Country specific category
95. Still in education/vocational training
97. Other

IF a97 IN DN053_FurtherEduParent

Txt_FL_DN054

DN054_WhichOtherEduParent

Which other degree of higher education or vocational training has [your/ your] [mother/ father] obtained?

OTHER HIGHEST PARENT

STRING

ENDIF

ENDIF

IF piParentAlive = 1

IF (piIndex = 2) OR (piIndex = 1)

IF DN026_NaturalParentAlive = a1

Txt_FL_DN030

DN030_LivingPlaceParent

Please look at card 5.

Where does [your/ your] [mother/ father] live?

WHERE DOES PARENT LIVE

1. In the same household
2. In the same building
3. Less than 1 kilometre away
4. Between 1 and 5 kilometres away
5. Between 5 and 25 kilometres away
6. Between 25 and 100 kilometres away
7. Between 100 and 500 kilometres away
8. More than 500 kilometres away

IF DN030_LivingPlaceParent > a1

Txt_FL_DN032

DN032_ContactDuringPast12Months

During the past twelve months, how often did you have contact with [your/ your] [mother/ father], either in person, by phone, mail, email or any other electronic means?

PERSONAL CONTACT WITH PARENT DURING PAST 12 MONTHS

1. Daily
2. Several times a week
3. About once a week
4. About every two weeks
5. About once a month
6. Less than once a month
7. Never

*ENDIF*Txt_FL_DN033

DN033_HealthParent

How would you describe the health of [your/ your] [mother/ father]? Would you say it is

IWER:

{ReadOut}

HEALTH OF PARENT
1. Excellent
2. Very good
3. Good
4. Fair
5. Poor

ENDIF

ELSE
Txt_FL_DN033

DN033_HealthParent
How would you describe the health of [your/ your] [mother/ father]? Would you say it is

IWER:
{ReadOut}
HEALTH OF PARENT
1. Excellent
2. Very good
3. Good
4. Fair
5. Poor

ENDIF

ENDIF

ENDBLOCK

ELSE

BLOCK

IF piParentAlive = 1

IF ((piIndex = 1) AND (Sec_SN.SN904_MotherInSocialNetwork = 1)) OR ((piIndex = 2) AND (Sec_SN.SN903_FatherInSocialNetwork = 1))
ELSE

IF (piIndex = 1) OR (piIndex = 2)
Txt_FL_DN026

DN026_NaturalParentAlive
Is [your/ your] [natural/ natural] [mother/ father] still alive?
IS NATURAL PARENT STILL ALIVE
1. Yes
5. No

IF DN026_NaturalParentAlive = a5
Txt_FL_DN127

DN127_YearOfDeathParent
[You told us in a previous interview that your mother is not alive anymore./ You told us in a previous interview that your father is not alive anymore.] In what year did [your/ your] [mother/ father] die?
AGE OF DEATH OF PARENT
1800..2015

ENDIF
IF DN026_NaturalParentAlive = a5
Txt_FL_DN027

DN027_AgeOfDeathParent
How old was [your/ your] [mother/ father] when [she/ he] died?
AGE OF DEATH OF PARENT
10..120

ELSE

IF (DN026_NaturalParentAlive = a1) AND (MN101_Longitudinal = 0)
Txt_FL_DN028

DN028_AgeOfNaturalParent
How old is [your/ your] [mother/ father] now?
AGE OF NATURAL PARENT
40..120
CHECK DN028_AgeOfNaturalParent >= (MN808_AgeRespondent + 10) L1 " [Age should be at least ten years above respondent""s age. If age is correct, please press "suppress" and enter a remark to explain]"

ENDIF

ENDIF

ELSE

IF MN101_Longitudinal = 0
Txt_FL_DN028

DN028_AgeOfNaturalParent
How old is [your/ your] [mother/ father] now?
AGE OF NATURAL PARENT
40..120
CHECK DN028_AgeOfNaturalParent >= (MN808_AgeRespondent + 10) L1 " [Age should be at least ten years above respondent""s age. If age is correct, please press "suppress" and enter a remark to explain]"

ENDIF

ENDIF

ENDIF

ELSE
Txt_FL_DN127

DN127_YearOfDeathParent
[You told us in a previous interview that your mother is not alive anymore./ You told us in a previous interview that your father is not alive anymore.] In what year did [your/ your] [mother/ father] die?
AGE OF DEATH OF PARENT
1800..2015

ENDIF
IF MN101_Longitudinal = 0
Txt_FL_DN629

DN629_JobSitParent10
Please look at card 7. In general, which of the following best describes [your/ your] [mother/ father] employment situation had when you were about 10 years old?
NAME OR TITLE OF JOB OF PARENT
1. Retired
2. Employed or self-employed (including working for family business)
3. Unemployed
4. Permanently sick or disabled
5. Homemaker
97. Other

IF DN629_JobSitParent10 = a2

Txt_FL_DN029

DN029_JobOfParent10

What was the job [your/ your] [mother/ father] had when you were about 10 years old? Please give the exact name or title.

NAME OR TITLE OF JOB OF PARENT

STRING

IF DN029_JobOfParent10 = RESPONSE

BLOCK

JobCode

STRING

ENDBLOCK

ENDIF

*ENDIF*Txt_FL_DN051

DN051_HighestEduParent

Please look at card 2. What is the highest school certificate or degree that [your/ your] [mother/ father] has obtained?

IWER:

If respondent mentions foreign degree/certificate, please ask if he/she can fit their degree into the given categories, if they cannot, please use the other option and type it in (next screen).

HIGHEST EDUCATIONAL DEGREE OF PARENT

1. No schooling/education at all
2. Some education, but less than [instead of put respective country specific degr.]
3. Country specific category
4. Country specific category
5. Country specific category
6. Country specific category
7. Country specific category
8. Country specific category
9. Country specific category
10. Country specific category
11. Country specific category
12. Country specific category
13. Country specific category
14. Country specific category
15. Country specific category
16. Country specific category
17. Country specific category
18. Country specific category
19. Country specific category
20. Country specific category
95. No degree yet/still in school
97. Other

IF DN051_HighestEduParent = a97

Txt_FL_DN052

DN052_OtherHighestEduParent

Which other school certificate or degree has [your/ your] [mother/ father] obtained?

OTHER HIGHEST EDUCATION PARENT

STRING

ENDIF Txt_FL_DN053

DN053_FurtherEduParent
Please look at card 3. Which degrees of higher education or vocational training does [your/ your] [mother/ father] have?

IWER:
{CodeAll}
FURTHER EDUCATION OR VOCATIONAL TRAINING PARENT
1. No higher education/vocational training
2. Some education, but less than [ISCED 1] (instead of [ISCED 1] put respective country specific degr.)
3. Country specific category
4. Country specific category
5. Country specific category
6. Country specific category
7. Country specific category
8. Country specific category
9. Country specific category
10. Country specific category
11. Country specific category
12. Country specific category
13. Country specific category
14. Country specific category
15. Country specific category
16. Country specific category
17. Country specific category
18. Country specific category
19. Country specific category
20. Country specific category
95. Still in education/vocational training
97. Other

IF a97 IN DN053_FurtherEduParent
Txt_FL_DN054

DN054_WhichOtherEduParent
Which other degree of higher education or vocational training has [your/ your] [mother/ father] obtained?
OTHER HIGHEST PARENT
STRING

ENDIF

ENDIF
IF piParentAlive = 1

IF (piIndex = 2) OR (piIndex = 1)

IF DN026_NaturalParentAlive = a1
Txt_FL_DN030

DN030_LivingPlaceParent
Please look at card 5.
Where does [your/ your] [mother/ father] live?
WHERE DOES PARENT LIVE
1. In the same household
2. In the same building
3. Less than 1 kilometre away
4. Between 1 and 5 kilometres away
5. Between 5 and 25 kilometres away
6. Between 25 and 100 kilometres away
7. Between 100 and 500 kilometres away

8. More than 500 kilometres away

IF DN030_LivingPlaceParent > a1

Txt_FL_DN032

DN032_ContactDuringPast12Months

During the past twelve months, how often did you have contact with [your/ your] [mother/ father], either in person, by phone, mail, email or any other electronic means?

PERSONAL CONTACT WITH PARENT DURING PAST 12 MONTHS

1. Daily
2. Several times a week
3. About once a week
4. About every two weeks
5. About once a month
6. Less than once a month
7. Never

*ENDIF*Txt_FL_DN033

DN033_HealthParent

How would you describe the health of [your/ your] [mother/ father]? Would you say it is

IWER:

{ReadOut}

HEALTH OF PARENT

1. Excellent
2. Very good
3. Good
4. Fair
5. Poor

ENDIF

ELSE

Txt_FL_DN033

DN033_HealthParent

How would you describe the health of [your/ your] [mother/ father]? Would you say it is

IWER:

{ReadOut}

HEALTH OF PARENT

1. Excellent
2. Very good
3. Good
4. Fair
5. Poor

ENDIF

ENDIF

ENDBLOCK

ENDIF

IF (Preload.PRELOAD_DN026_NaturalParentAlive[2] <> a5) OR (Sec_SN.SN904_MotherInSocialNetwork = 1)

BLOCK

IF piParentAlive = 1

IF ((piIndex = 1) AND (Sec_SN.SN904_MotherInSocialNetwork = 1)) OR ((piIndex = 2) AND (Sec_SN.SN903_FatherInSocialNetwork = 1))
ELSE

IF (piIndex = 1) OR (piIndex = 2)

Txt_FL_DN026

DN026_NaturalParentAlive
Is [your/ your] [natural/ natural] [mother/ father] still alive?
IS NATURAL PARENT STILL ALIVE
1. Yes
5. No

IF DN026_NaturalParentAlive = a5

Txt_FL_DN127

DN127_YearOfDeathParent
[You told us in a previous interview that your mother is not alive anymore./ You told us in a previous interview that your father is not alive anymore.] In what year did [your/ your] [mother/ father] die?
AGE OF DEATH OF PARENT
1800..2015

ENDIF
IF DN026_NaturalParentAlive = a5

Txt_FL_DN027

DN027_AgeOfDeathParent
How old was [your/ your] [mother/ father] when [she/ he] died?
AGE OF DEATH OF PARENT
10..120

ELSE

IF (DN026_NaturalParentAlive = a1) AND (MN101_Longitudinal = 0)

Txt_FL_DN028

DN028_AgeOfNaturalParent
How old is [your/ your] [mother/ father] now?
AGE OF NATURAL PARENT
40..120
CHECK DN028_AgeOfNaturalParent >= (MN808_AgeRespondent + 10) L1 " [Age should be at least ten years above respondent""s age. If age is correct, please press "suppress" and enter a remark to explain]"

ENDIF

ENDIF

ELSE

IF MN101_Longitudinal = 0

Txt_FL_DN028

DN028_AgeOfNaturalParent
How old is [your/ your] [mother/ father] now?
AGE OF NATURAL PARENT
40..120
CHECK DN028_AgeOfNaturalParent >= (MN808_AgeRespondent + 10) L1 " [Age should be at least ten years above respondent""s age. If age is correct, please press "suppress" and enter a remark to explain]"

ENDIF

ENDIF

ENDIF

ELSE

Txt_FL_DN127

DN127_YearOfDeathParent

[You told us in a previous interview that your mother is not alive anymore./ You told us in a previous interview that your father is not alive anymore.] In what year did [your/ your] [mother/ father] die?

AGE OF DEATH OF PARENT

1800..2015

ENDIF

IF MN101_Longitudinal = 0

Txt_FL_DN629

DN629_JobSitParent10

Please look at card 7. In general, which of the following best describes [your/ your] [mother/ father] employment situation had when you were about 10 years old?

NAME OR TITLE OF JOB OF PARENT

1. Retired
2. Employed or self-employed (including working for family business)
3. Unemployed
4. Permanently sick or disabled
5. Homemaker

97. Other

IF DN629_JobSitParent10 = a2

Txt_FL_DN029

DN029_JobOfParent10

What was the job [your/ your] [mother/ father] had when you were about 10 years old? Please give the exact name or title.

NAME OR TITLE OF JOB OF PARENT

STRING

IF DN029_JobOfParent10 = RESPONSE

BLOCK

JobCode

STRING

ENDBLOCK

ENDIF

*ENDIF*Txt_FL_DN051

DN051_HighestEduParent

Please look at card 2. What is the highest school certificate or degree that [your/ your] [mother/ father] has obtained?

IWER:

If respondent mentions foreign degree/certificate, please ask if he/she can fit their degree into the given categories, if they cannot, please use the other option and type it in (next screen).

HIGHEST EDUCATIONAL DEGREE OF PARENT

1. No schooling/education at all
2. Some education, but less than [instead of put respective country specific degr.]
3. Country specific category

4. Country specific category
5. Country specific category
6. Country specific category
7. Country specific category
8. Country specific category
9. Country specific category
10. Country specific category
11. Country specific category
12. Country specific category
13. Country specific category
14. Country specific category
15. Country specific category
16. Country specific category
17. Country specific category
18. Country specific category
19. Country specific category
20. Country specific category
95. No degree yet/still in school
97. Other

IF DN051_HighestEduParent = a97

Txt_FL_DN052

DN052_OtherHighestEduParent

Which other school certificate or degree has [your/ your] [mother/ father] obtained?

OTHER HIGHEST EDUCATION PARENT

STRING

*ENDIF*Txt_FL_DN053

DN053_FurtherEduParent

Please look at card 3. Which degrees of higher education or vocational training does [your/ your] [mother/ father] have?

IWER:

{CodeAll}

FURTHER EDUCATION OR VOCATIONAL TRAINING PARENT

1. No higher education/vocational training
2. Some education, but less than [ISCED 1] (instead of [ISCED 1] put respective country specific degr.)
3. Country specific category
4. Country specific category
5. Country specific category
6. Country specific category
7. Country specific category
8. Country specific category
9. Country specific category
10. Country specific category
11. Country specific category
12. Country specific category
13. Country specific category
14. Country specific category
15. Country specific category
16. Country specific category
17. Country specific category
18. Country specific category
19. Country specific category
20. Country specific category
95. Still in education/vocational training
97. Other

IF a97 IN DN053_FurtherEduParent

Txt_FL_DN054

DN054_WhichOtherEduParent
Which other degree of higher education or vocational training has [your/ your] [mother/ father] obtained?
OTHER HIGHEST PARENT
STRING

ENDIF

ENDIF
IF piParentAlive = 1

IF (piIndex = 2) OR (piIndex = 1)

IF DN026_NaturalParentAlive = a1
Txt_FL_DN030

DN030_LivingPlaceParent
Please look at card 5.
Where does [your/ your] [mother/ father] live?
WHERE DOES PARENT LIVE
1. In the same household
2. In the same building
3. Less than 1 kilometre away
4. Between 1 and 5 kilometres away
5. Between 5 and 25 kilometres away
6. Between 25 and 100 kilometres away
7. Between 100 and 500 kilometres away
8. More than 500 kilometres away

IF DN030_LivingPlaceParent > a1
Txt_FL_DN032

DN032_ContactDuringPast12Months
During the past twelve months, how often did you have contact with [your/ your] [mother/ father], either in person, by phone, mail, email or any other electronic means?
PERSONAL CONTACT WITH PARENT DURING PAST 12 MONTHS
1. Daily
2. Several times a week
3. About once a week
4. About every two weeks
5. About once a month
6. Less than once a month
7. Never

*ENDIF*Txt_FL_DN033

DN033_HealthParent
How would you describe the health of [your/ your] [mother/ father]? Would you say it is

IWER:
{ReadOut}
HEALTH OF PARENT
1. Excellent
2. Very good
3. Good
4. Fair
5. Poor

ENDIF

ELSE

Txt_FL_DN033

DN033_HealthParent
How would you describe the health of [your/ your] [mother/ father]? Would you say it is

IWER:
{ReadOut}
HEALTH OF PARENT
1. Excellent
2. Very good
3. Good
4. Fair
5. Poor

ENDIF

ENDIF

ENDBLOCK

ELSE

BLOCK

IF piParentAlive = 1

IF ((piIndex = 1) AND (Sec_SN.SN904_MotherInSocialNetwork = 1)) OR ((piIndex = 2) AND (Sec_SN.SN903_FatherInSocialNetwork = 1))
ELSE

IF (piIndex = 1) OR (piIndex = 2)
Txt_FL_DN026

DN026_NaturalParentAlive
Is [your/ your] [natural/ natural] [mother/ father] still alive?
IS NATURAL PARENT STILL ALIVE
1. Yes
5. No

IF DN026_NaturalParentAlive = a5
Txt_FL_DN127

DN127_YearOfDeathParent
[You told us in a previous interview that your mother is not alive anymore./ You told us in a previous interview that your father is not alive anymore.] In what year did [your/ your] [mother/ father] die?
AGE OF DEATH OF PARENT
1800..2015

ENDIF
IF DN026_NaturalParentAlive = a5
Txt_FL_DN027

DN027_AgeOfDeathParent
How old was [your/ your] [mother/ father] when [she/ he] died?
AGE OF DEATH OF PARENT
10..120

ELSE

IF (DN026_NaturalParentAlive = a1) AND (MN101_Longitudinal = 0)
Txt_FL_DN028

DN028_AgeOfNaturalParent
How old is [your/ your] [mother/ father] now?
AGE OF NATURAL PARENT
40..120
CHECK DN028_AgeOfNaturalParent >= (MN808_AgeRespondent + 10) L1 " [Age should be at least ten years above respondent""s age. If age is correct, please press "suppress" and enter a remark to explain]"

ENDIF

ENDIF

ELSE

IF MN101_Longitudinal = 0
Txt_FL_DN028

DN028_AgeOfNaturalParent
How old is [your/ your] [mother/ father] now?
AGE OF NATURAL PARENT
40..120
CHECK DN028_AgeOfNaturalParent >= (MN808_AgeRespondent + 10) L1 " [Age should be at least ten years above respondent""s age. If age is correct, please press "suppress" and enter a remark to explain]"

ENDIF

ENDIF

ENDIF

ELSE
Txt_FL_DN127

DN127_YearOfDeathParent
[You told us in a previous interview that your mother is not alive anymore./ You told us in a previous interview that your father is not alive anymore.] In what year did [your/ your] [mother/ father] die?
AGE OF DEATH OF PARENT
1800..2015

ENDIF
IF MN101_Longitudinal = 0
Txt_FL_DN629

DN629_JobSitParent10
Please look at card 7. In general, which of the following best describes [your/ your] [mother/ father] employment situation had when you were about 10 years old?
NAME OR TITLE OF JOB OF PARENT
1. Retired
2. Employed or self-employed (including working for family business)
3. Unemployed
4. Permanently sick or disabled
5. Homemaker
97. Other

IF DN629_JobSitParent10 = a2
Txt_FL_DN029

DN029_JobOfParent10
What was the job [your/ your] [mother/ father] had when you were about 10 years old? Please give the exact name or title.
NAME OR TITLE OF JOB OF PARENT
STRING

IF DN029_JobOfParent10 = RESPONSE

BLOCK

JobCode

STRING

ENDBLOCK

ENDIF

*ENDIF*Txt_FL_DN051

DN051_HighestEduParent
Please look at card 2. What is the highest school certificate or degree that [your/ your] [mother/ father] has obtained?

IWER:
If respondent mentions foreign degree/certificate, please ask if he/she can fit their degree into the given categories, if they cannot, please use the other option and type it in (next screen).
HIGHEST EDUCATIONAL DEGREE OF PARENT
1. No schooling/education at all
2. Some education, but less than [instead of put respective country specific degr.]
3. Country specific category
4. Country specific category
5. Country specific category
6. Country specific category
7. Country specific category
8. Country specific category
9. Country specific category
10. Country specific category
11. Country specific category
12. Country specific category
13. Country specific category
14. Country specific category
15. Country specific category
16. Country specific category
17. Country specific category
18. Country specific category
19. Country specific category
20. Country specific category
95. No degree yet/still in school
97. Other

IF DN051_HighestEduParent = a97
Txt_FL_DN052

DN052_OtherHighestEduParent
Which other school certificate or degree has [your/ your] [mother/ father] obtained?
OTHER HIGHEST EDUCATION PARENT
STRING

*ENDIF*Txt_FL_DN053

DN053_FurtherEduParent
Please look at card 3. Which degrees of higher education or vocational training does [your/ your] [mother/ father] have?

IWER:
{CodeAll}
FURTHER EDUCATION OR VOCATIONAL TRAINING PARENT

1. No higher education/vocational training
2. Some education, but less than [ISCED 1] (instead of [ISCED 1] put respective country specific degr.)
3. Country specific category
4. Country specific category
5. Country specific category
6. Country specific category
7. Country specific category
8. Country specific category
9. Country specific category
10. Country specific category
11. Country specific category
12. Country specific category
13. Country specific category
14. Country specific category
15. Country specific category
16. Country specific category
17. Country specific category
18. Country specific category
19. Country specific category
20. Country specific category
95. Still in education/vocational training
97. Other

IF a97 IN DN053_FurtherEduParent

Txt_FL_DN054

DN054_WhichOtherEduParent

Which other degree of higher education or vocational training has [your/ your] [mother/ father] obtained?

OTHER HIGHEST PARENT

STRING

ENDIF

ENDIF

IF piParentAlive = 1

IF (piIndex = 2) OR (piIndex = 1)

IF DN026_NaturalParentAlive = a1

Txt_FL_DN030

DN030_LivingPlaceParent

Please look at card 5.

Where does [your/ your] [mother/ father] live?

WHERE DOES PARENT LIVE

1. In the same household
2. In the same building
3. Less than 1 kilometre away
4. Between 1 and 5 kilometres away
5. Between 5 and 25 kilometres away
6. Between 25 and 100 kilometres away
7. Between 100 and 500 kilometres away
8. More than 500 kilometres away

IF DN030_LivingPlaceParent > a1

Txt_FL_DN032

DN032_ContactDuringPast12Months

During the past twelve months, how often did you have contact with [your/ your] [mother/ father], either in person, by phone, mail, email or any other electronic means?

PERSONAL CONTACT WITH PARENT DURING PAST 12 MONTHS

1. Daily

2. Several times a week
3. About once a week
4. About every two weeks
5. About once a month
6. Less than once a month
7. Never

*ENDIF*Txt_FL_DN033

DN033_HealthParent
How would you describe the health of [your/ your] [mother/ father]? Would you say it is

IWER:
{ReadOut}
HEALTH OF PARENT
1. Excellent
2. Very good
3. Good
4. Fair
5. Poor

ENDIF

ELSE
Txt_FL_DN033

DN033_HealthParent
How would you describe the health of [your/ your] [mother/ father]? Would you say it is

IWER:
{ReadOut}
HEALTH OF PARENT
1. Excellent
2. Very good
3. Good
4. Fair
5. Poor

ENDIF

ENDIF

ENDBLOCK

ENDIF

ENDBLOCK
BLOCK

IF MN101_Longitudinal = 0
Txt_FL_DN034

DN034_AnySiblings
Have you ever had any siblings?

IWER:
Include non-biological siblings
EVER HAD ANY SIBLINGS
1. Yes
5. No

IF DN034_AnySiblings = a1

Txt_FL_DN035

DN035_OldestYoungestBetweenChild

Talking about your siblings, were you the oldest child, the youngest child, or somewhere in-between?

OLDEST YOUNGEST CHILD

1. Oldest
2. Youngest
3. In-between

ENDIF

ENDIF

IF (DN034_AnySiblings = a1) OR ((Preload.PRELOAD_DN036_HowManyBrothersAlive <> 0) OR (Preload.PRELOAD_DN037_HowManySistersAlive <> 0))

Txt_FL_DN036

DN036_HowManyBrothersAlive

How many brothers do you have that are still alive?

IWER:

Include non-biological

HOW MANY BROTHERS ALIVE

0..20

Txt_FL_DN037

DN037_HowManySistersAlive

And how many sisters do you have that are still alive?

IWER:

Include non-biological

HOW MANY SISTERS ALIVE

0..20

ENDIF

ENDBLOCK

System preset value DN902_TimeStampEnd

IF DN902_TimeStampEnd = EMPTY

ENDIF

ENDBLOCK

ENDIF

IF (CH IN Test) OR (ALL IN Test)

BLOCK

IF MN006_NumFamR = 1

Txt_FL_CH001

CH001_NumberOfChildren

Now I will ask some questions about your children. How many children do you have that are still alive? Please count all natural children, fostered, adopted and stepchildren [, including those of/ , including those of/ , including those of/ , including those of] [your husband/ your wife/ your partner/ your partner] [{Name of partner/spouse}].

NUMBER OF CHILDREN

0..20

CHECK NOT ((Sec_SN.SN906_ChildInSocialNetwork > 0) AND (CH001_NumberOfChildren = 0)) L1 " [You mentioned children in the Social network module, please correct.]"

IF CH001_NumberOfChildren > 0

IF NOT ((Preload.PreloadedChildren[1].Name = '') OR Preload.PreloadedChildren[1].Name = EMPTY) OR (Sec_SN.SN906_ChildInSocialNetwork > 0)

Txt_FL_CH201

CH201_ChildByINTRO

I will read a list of all children we have talked about [today/ today or in a previous interview].
Some of your children may be listed twice in this list, others may be missing or we may have missing or wrong information for some children.

I would like to go through this list with you and make sure we have complete and correct information for all natural children, fostered, adopted and stepchildren. We are interested in children that are still alive.
INTRO PRELOADED CHILDREN

1. Continue

ELSE

Txt_FL_CH603

CH603_IntroTextChildren

We would like to know more about [this child/ these children. Let us begin with the oldest child]. Again, please think of all natural children, fostered, adopted and stepchildren [including those of your husband/ including those of your wife/ including those of your partner/ including those of your partner].
INTRO IF NO SN OR PRELOADED CHILDREN

1. Continue

ENDIF

CH201_ChildByEnum

:*TABLE Section_CH.TChild*
LOOP cnt:= 1 TO 20

IF cnt > 1

IF (Child[cnt - 1].CH001a_ChildCheck = a1) OR (Child[cnt - 1].CH001a_ChildCheck = a2)
ENDIF

ENDIF
IF Preload.PreloadedChildren[cnt].Kidcom <> EMPTY

BLOCK

System preset value PRELOAD_ID
System preset value DISTANCE
System preset value CONTACT
System preset value SN_ID
System preset value CH908_RosterNumber
System preset value FL_CHILD_NAME
System preset value CH904_FirstNameOfChildBeforeCleaning
System preset value CH905_ForwardedFromSN
IF (piIndex <= GridSize) AND (imForwarded = 0)
ELSE

IF piPreloadChildIndex = 0

Txt_FL_CH001a

CH001a_ChildCheck

Do you have [{dynamic constructed text based on how the child was loaded}]? Again, please think of all natural children, fostered, adopted and stepchildren [, including those of/ , including those of/ , including those of/ , including those of] [your husband/ your wife/ your partner/ your partner]. [

{dynamic constructed text based on how the child was loaded}]3
CHILD CONFIRM
1. Yes
^FL_CH001a_7
^FL_CH001a_8
^FL_CH001a_9
^FL_CH001a_10
^FL_CH001a_11
^FL_CH001a_12

ELSE

IF piPreloadChildIndex > 0
Txt_FL_CH001a

CH001a_ChildCheck
Do you have [{dynamic constructed text based on how the child was loaded}]? Again, please think of all natural children, fostered, adopted and stepchildren [, including those of/ , including those of/ , including those of/ , including those of] [your husband/ your wife/ your partner/ your partner]. [{dynamic constructed text based on how the child was loaded}]3
CHILD CONFIRM
1. Yes
^FL_CH001a_7
^FL_CH001a_8
^FL_CH001a_9
^FL_CH001a_10
^FL_CH001a_11
^FL_CH001a_12

ELSE
Txt_FL_CH001a

CH001a_ChildCheck
Do you have [{dynamic constructed text based on how the child was loaded}]? Again, please think of all natural children, fostered, adopted and stepchildren [, including those of/ , including those of/ , including those of/ , including those of] [your husband/ your wife/ your partner/ your partner]. [{dynamic constructed text based on how the child was loaded}]3
CHILD CONFIRM
1. Yes
^FL_CH001a_7
^FL_CH001a_8
^FL_CH001a_9
^FL_CH001a_10
^FL_CH001a_11
^FL_CH001a_12

ENDIF

ENDIF

ENDIF
IF piRosterChildIndex > 0
ENDIFCHECK NOT ((CH001a_ChildCheck = a97) AND (piPreloadChildIndex = 0)) L1 " {FLError[51]}"

IF (CH001a_ChildCheck = a1) OR CH001a_ChildCheck = EMPTY

IF piPreloadChildIndex = 0

IF (piRosterChildIndex > 0) AND CH908_RosterNumber = EMPTY

IF CH004_FirstNameOfChild = EMPTY

ENDIF
IF CH005_SexOfChildN = EMPTY
ENDIF
IF CH006_YearOfBirthChildN = EMPTY
ENDIF

ENDIF

ELSE

IF piPreloadChildIndex > 0
ELSE
ENDIF

*ENDIF*Txt_FL_CH004

IF CH004_FirstNameOfChild = EMPTY

CH004_FirstNameOfChild
[(' The name ' + PreloadedName) + ' was found.']

What is the [correct] first name of [this/ your next] child?

IWER:
Please enter/confirm first name
FIRST NAME OF CHILD N
STRING

ELSE
ENDIF
IF CH005_SexOfChildN = EMPTY

CH005_SexOfChildN
Is {CH004_FirstNameOfChild} male or female?

IWER:
Ask only if unclear
SEX OF CHILD N
1. Male
2. Female

ELSE
ENDIF
IF CH006_YearOfBirthChildN = EMPTY

CH006_YearOfBirthChildN
In which year was {CH004_FirstNameOfChild} born?

IWER:
Please enter/confirm year of birth
YEAR OF BIRTH CHILD N
1875..2015

ELSE
ENDIF

ELSE

IF CH001a_ChildCheck = a2
Txt_FL_CH004

CH004_FirstNameOfChild
[(' The name ' + PreloadedName) + ' was found.']

What is the [correct] first name of [this/ your next] child?

IWER:
Please enter/confirm first name
FIRST NAME OF CHILD N
STRING

CH005_SexOfChildN
Is {CH004_FirstNameOfChild} male or female?

IWER:
Ask only if unclear
SEX OF CHILD N
1. Male
2. Female

Txt_FL_CH006

CH006_YearOfBirthChildN
In which year was {CH004_FirstNameOfChild} born?

IWER:
Please enter/confirm year of birth
YEAR OF BIRTH CHILD N
1875..2015

IF piPreloadChildIndex > 0
ELSE
ENDIF

ELSE

IF ((piPreloadChildIndex > 0) OR ((piPreloadChildIndex = 0) AND (piRosterChildIndex > 0))) AND (CH001a_ChildCheck = a6)

CH505_WhichChildMentionedEarlier

IWER:
To which child that was already mentioned earlier is {FL_CHILD_NAME} equal?
EQUAL TO WHICH CHILD
^FLChild[1]
^FLChild[2]
^FLChild[3]
^FLChild[4]
^FLChild[5]
^FLChild[6]
^FLChild[7]
^FLChild[8]
^FLChild[9]
^FLChild[10]
^FLChild[11]
^FLChild[12]
^FLChild[13]
^FLChild[14]
^FLChild[15]
^FLChild[16]
^FLChild[17]
^FLChild[18]
^FLChild[19]

IF (CH505_WhichChildMentionedEarlier = RESPONSE) AND (piRosterChildIndex > 0)

ENDIF

ENDIF

ENDIF

ENDIF
IF (CH001a_ChildCheck = a1) OR (CH001a_ChildCheck = a2)
ELSE
ENDIF

ENDBLOCK

ELSE

IF (Sec_SN.SN906_ChildInSocialNetwork > 0) AND (Sec_SN.SN_Child[cnt - sn_start].Name = RESPONSE)

BLOCK

System preset value PRELOAD_ID
System preset value DISTANCE
System preset value CONTACT
System preset value SN_ID
System preset value CH908_RosterNumber
System preset value FL_CHILD_NAME
System preset value CH904_FirstNameOfChildBeforeCleaning
System preset value CH905_ForwardedFromSN
IF (piIndex <= GridSize) AND (imForwarded = 0)
ELSE

IF piPreloadChildIndex = 0
Txt_FL_CH001a

CH001a_ChildCheck
Do you have [{dynamic constructed text based on how the child was loaded}]? Again, please think of all natural children, fostered, adopted and stepchildren [, including those of/ , including those of/ , including those of/ , including those of] [your husband/ your wife/ your partner/ your partner]. [{dynamic constructed text based on how the child was loaded}]3
CHILD CONFIRM
1. Yes
^FL_CH001a_7
^FL_CH001a_8
^FL_CH001a_9
^FL_CH001a_10
^FL_CH001a_11
^FL_CH001a_12

ELSE

IF piPreloadChildIndex > 0
Txt_FL_CH001a

CH001a_ChildCheck
Do you have [{dynamic constructed text based on how the child was loaded}]? Again, please think of all natural children, fostered, adopted and stepchildren [, including those of/ , including those of/ , including those of/ , including those of] [your husband/ your wife/ your partner/ your partner]. [{dynamic constructed text based on how the child was loaded}]3
CHILD CONFIRM
1. Yes
^FL_CH001a_7
^FL_CH001a_8
^FL_CH001a_9
^FL_CH001a_10

^FL_CH001a_11
^FL_CH001a_12

ELSE

Txt_FL_CH001a

CH001a_ChildCheck
Do you have [{dynamic constructed text based on how the child was loaded}]? Again, please think of all natural children, fostered, adopted and stepchildren [, including those of/ , including those of/ , including those of/ , including those of] [your husband/ your wife/ your partner/ your partner]. [{dynamic constructed text based on how the child was loaded}]3
CHILD CONFIRM
1. Yes
^FL_CH001a_7
^FL_CH001a_8
^FL_CH001a_9
^FL_CH001a_10
^FL_CH001a_11
^FL_CH001a_12

ENDIF

ENDIF

ENDIF
IF piRosterChildIndex > 0
*ENDIF*CHECK NOT ((CH001a_ChildCheck = a97) AND (piPreloadChildIndex = 0)) L1 " {FLError[51]}"

IF (CH001a_ChildCheck = a1) OR CH001a_ChildCheck = EMPTY

IF piPreloadChildIndex = 0

IF (piRosterChildIndex > 0) AND CH908_RosterNumber = EMPTY

IF CH004_FirstNameOfChild = EMPTY
ENDIF
IF CH005_SexOfChildN = EMPTY
ENDIF
IF CH006_YearOfBirthChildN = EMPTY
ENDIF

ENDIF

ELSE

IF piPreloadChildIndex > 0
ELSE
ENDIF

*ENDIF*Txt_FL_CH004

IF CH004_FirstNameOfChild = EMPTY

CH004_FirstNameOfChild
[(' The name ' + PreloadedName) + ' was found.']

What is the [correct] first name of [this/ your next] child?

IWER:
Please enter/confirm first name
FIRST NAME OF CHILD N
STRING

ELSE
ENDIF
IF CH005_SexOfChildN = EMPTY

CH005_SexOfChildN
Is {CH004_FirstNameOfChild} male or female?

IWER:
Ask only if unclear
SEX OF CHILD N
1. Male
2. Female

ELSE
ENDIF
IF CH006_YearOfBirthChildN = EMPTY

CH006_YearOfBirthChildN
In which year was {CH004_FirstNameOfChild} born?

IWER:
Please enter/confirm year of birth
YEAR OF BIRTH CHILD N
1875..2015

ELSE
ENDIF

ELSE

IF CH001a_ChildCheck = a2
Txt_FL_CH004

CH004_FirstNameOfChild
[(' The name ' + PreloadedName) + ' was found.']

What is the [correct] first name of [this/ your next] child?

IWER:
Please enter/confirm first name
FIRST NAME OF CHILD N
STRING

CH005_SexOfChildN
Is {CH004_FirstNameOfChild} male or female?

IWER:
Ask only if unclear
SEX OF CHILD N
1. Male
2. Female

Txt_FL_CH006

CH006_YearOfBirthChildN
In which year was {CH004_FirstNameOfChild} born?

IWER:
Please enter/confirm year of birth
YEAR OF BIRTH CHILD N
1875..2015

IF piPreloadChildIndex > 0
ELSE
ENDIF

ELSE

IF ((piPreloadChildIndex > 0) OR ((piPreloadChildIndex = 0) AND (piRosterChildIndex > 0))) AND (CH001a_ChildCheck = a6)

CH505_WhichChildMentionedEarlier

IWER:
To which child that was already mentioned earlier is {FL_CHILD_NAME} equal?
EQUAL TO WHICH CHILD
^FLChild[1]
^FLChild[2]
^FLChild[3]
^FLChild[4]
^FLChild[5]
^FLChild[6]
^FLChild[7]
^FLChild[8]
^FLChild[9]
^FLChild[10]
^FLChild[11]
^FLChild[12]
^FLChild[13]
^FLChild[14]
^FLChild[15]
^FLChild[16]
^FLChild[17]
^FLChild[18]
^FLChild[19]

IF (CH505_WhichChildMentionedEarlier = RESPONSE) AND (piRosterChildIndex > 0)
ENDIF

ENDIF

ENDIF

ENDIF
IF (CH001a_ChildCheck = a1) OR (CH001a_ChildCheck = a2)
ELSE
ENDIF

ENDBLOCK

ELSE

IF (Child[cnt - 1].CH001a_ChildCheck = a5) AND (Child[cnt - 1].CH004_FirstNameOfChild = '')
ELSE

IF NumChildren < GridSize

BLOCK

System preset value PRELOAD_ID
System preset value DISTANCE
System preset value CONTACT
System preset value SN_ID
System preset value CH908_RosterNumber

System preset value FL_CHILD_NAME
System preset value CH904_FirstNameOfChildBeforeCleaning
System preset value CH905_ForwardedFromSN
IF (piIndex <= GridSize) AND (imForwarded = 0)
ELSE

IF piPreloadChildIndex = 0
Txt_FL_CH001a

CH001a_ChildCheck
Do you have [{dynamic constructed text based on how the child was loaded}]? Again, please think of all natural children, fostered, adopted and stepchildren [, including those of/ , including those of/ , including those of/ , including those of] [your husband/ your wife/ your partner/ your partner]. [{dynamic constructed text based on how the child was loaded}]3
CHILD CONFIRM
1. Yes
^FL_CH001a_7
^FL_CH001a_8
^FL_CH001a_9
^FL_CH001a_10
^FL_CH001a_11
^FL_CH001a_12

ELSE

IF piPreloadChildIndex > 0
Txt_FL_CH001a

CH001a_ChildCheck
Do you have [{dynamic constructed text based on how the child was loaded}]? Again, please think of all natural children, fostered, adopted and stepchildren [, including those of/ , including those of/ , including those of/ , including those of] [your husband/ your wife/ your partner/ your partner]. [{dynamic constructed text based on how the child was loaded}]3
CHILD CONFIRM
1. Yes
^FL_CH001a_7
^FL_CH001a_8
^FL_CH001a_9
^FL_CH001a_10
^FL_CH001a_11
^FL_CH001a_12

ELSE
Txt_FL_CH001a

CH001a_ChildCheck
Do you have [{dynamic constructed text based on how the child was loaded}]? Again, please think of all natural children, fostered, adopted and stepchildren [, including those of/ , including those of/ , including those of/ , including those of] [your husband/ your wife/ your partner/ your partner]. [{dynamic constructed text based on how the child was loaded}]3
CHILD CONFIRM
1. Yes
^FL_CH001a_7
^FL_CH001a_8
^FL_CH001a_9
^FL_CH001a_10
^FL_CH001a_11
^FL_CH001a_12

ENDIF

ENDIF

ENDIF
IF piRosterChildIndex > 0
*ENDIF*CHECK NOT ((CH001a_ChildCheck = a97) AND (piPreloadChildIndex = 0)) L1 " {FLError[51]}"

IF (CH001a_ChildCheck = a1) OR CH001a_ChildCheck = EMPTY

IF piPreloadChildIndex = 0

IF (piRosterChildIndex > 0) AND CH908_RosterNumber = EMPTY

IF CH004_FirstNameOfChild = EMPTY
ENDIF
IF CH005_SexOfChildN = EMPTY
ENDIF
IF CH006_YearOfBirthChildN = EMPTY
ENDIF

ENDIF

ELSE

IF piPreloadChildIndex > 0
ELSE
ENDIF

*ENDIF*Txt_FL_CH004

IF CH004_FirstNameOfChild = EMPTY

CH004_FirstNameOfChild
[(' The name ' + PreloadedName) + ' was found.']

What is the [correct] first name of [this/ your next] child?

IWER:
Please enter/confirm first name
FIRST NAME OF CHILD N
STRING

ELSE
ENDIF
IF CH005_SexOfChildN = EMPTY

CH005_SexOfChildN
Is {CH004_FirstNameOfChild} male or female?

IWER:
Ask only if unclear
SEX OF CHILD N
1. Male
2. Female

ELSE
ENDIF
IF CH006_YearOfBirthChildN = EMPTY

CH006_YearOfBirthChildN
In which year was {CH004_FirstNameOfChild} born?

IWER:
Please enter/confirm year of birth

YEAR OF BIRTH CHILD N
1875..2015

ELSE
ENDIF

ELSE

IF CH001a_ChildCheck = a2
Txt_FL_CH004

CH004_FirstNameOfChild
[(' The name ' + PreloadedName) + ' was found.']

What is the [correct] first name of [this/ your next] child?

IWER:
Please enter/confirm first name
FIRST NAME OF CHILD N
STRING

CH005_SexOfChildN
Is {CH004_FirstNameOfChild} male or female?

IWER:
Ask only if unclear
SEX OF CHILD N
1. Male
2. Female

Txt_FL_CH006

CH006_YearOfBirthChildN
In which year was {CH004_FirstNameOfChild} born?

IWER:
Please enter/confirm year of birth
YEAR OF BIRTH CHILD N
1875..2015

IF piPreloadChildIndex > 0
ELSE
ENDIF

ELSE

IF ((piPreloadChildIndex > 0) OR ((piPreloadChildIndex = 0) AND (piRosterChildIndex > 0))) AND (CH001a_ChildCheck = a6)

CH505_WhichChildMentionedEarlier

IWER:
To which child that was already mentioned earlier is {FL_CHILD_NAME} equal?
EQUAL TO WHICH CHILD
^FLChild[1]
^FLChild[2]
^FLChild[3]
^FLChild[4]
^FLChild[5]
^FLChild[6]
^FLChild[7]
^FLChild[8]
^FLChild[9]

^FLChild[10]
^FLChild[11]
^FLChild[12]
^FLChild[13]
^FLChild[14]
^FLChild[15]
^FLChild[16]
^FLChild[17]
^FLChild[18]
^FLChild[19]

IF (CH505_WhichChildMentionedEarlier = RESPONSE) AND (piRosterChildIndex > 0)
ENDIF

ENDIF

ENDIF

ENDIF
IF (CH001a_ChildCheck = a1) OR (CH001a_ChildCheck = a2)
ELSE
ENDIF

ENDBLOCK

ELSE

IF Child[cnt - 1].CH004_FirstNameOfChild <> EMPTY OR Preload.PreloadedChildren[cnt - 1].Kidcom <> EMPTY

BLOCK

System preset value PRELOAD_ID
System preset value DISTANCE
System preset value CONTACT
System preset value SN_ID
System preset value CH908_RosterNumber
System preset value FL_CHILD_NAME
System preset value CH904_FirstNameOfChildBeforeCleaning
System preset value CH905_ForwardedFromSN
IF (piIndex <= GridSize) AND (imForwarded = 0)
ELSE

IF piPreloadChildIndex = 0
Txt_FL_CH001a

CH001a_ChildCheck
Do you have [{dynamic constructed text based on how the child was loaded}]? Again, please think of all natural children, fostered, adopted and stepchildren [, including those of/ , including those of/ , including those of/ , including those of] [your husband/ your wife/ your partner/ your partner]. [{dynamic constructed text based on how the child was loaded}]3
CHILD CONFIRM
1. Yes
^FL_CH001a_7
^FL_CH001a_8
^FL_CH001a_9
^FL_CH001a_10
^FL_CH001a_11
^FL_CH001a_12

ELSE

IF piPreloadChildIndex > 0

Txt_FL_CH001a

CH001a_ChildCheck

Do you have [{dynamic constructed text based on how the child was loaded}]? Again, please think of all natural children, fostered, adopted and stepchildren [, including those of/ , including those of/ , including those of/ , including those of] [your husband/ your wife/ your partner/ your partner]. [{dynamic constructed text based on how the child was loaded}]3

CHILD CONFIRM

1. Yes

^FL_CH001a_7

^FL_CH001a_8

^FL_CH001a_9

^FL_CH001a_10

^FL_CH001a_11

^FL_CH001a_12

ELSE

Txt_FL_CH001a

CH001a_ChildCheck

Do you have [{dynamic constructed text based on how the child was loaded}]? Again, please think of all natural children, fostered, adopted and stepchildren [, including those of/ , including those of/ , including those of/ , including those of] [your husband/ your wife/ your partner/ your partner]. [{dynamic constructed text based on how the child was loaded}]3

CHILD CONFIRM

1. Yes

^FL_CH001a_7

^FL_CH001a_8

^FL_CH001a_9

^FL_CH001a_10

^FL_CH001a_11

^FL_CH001a_12

ENDIF

ENDIF

ENDIF

IF piRosterChildIndex > 0

*ENDIF*CHECK NOT ((CH001a_ChildCheck = a97) AND (piPreloadChildIndex = 0)) L1 " {FLError[51]}"

IF (CH001a_ChildCheck = a1) OR CH001a_ChildCheck = EMPTY

IF piPreloadChildIndex = 0

IF (piRosterChildIndex > 0) AND CH908_RosterNumber = EMPTY

IF CH004_FirstNameOfChild = EMPTY

ENDIF

IF CH005_SexOfChildN = EMPTY

ENDIF

IF CH006_YearOfBirthChildN = EMPTY

ENDIF

ENDIF

ELSE

IF piPreloadChildIndex > 0

ELSE

ENDIF

*ENDIF*Txt_FL_CH004

IF CH004_FirstNameOfChild = EMPTY

CH004_FirstNameOfChild
[(' The name ' + PreloadedName) + ' was found.']

What is the [correct] first name of [this/ your next] child?

IWER:
Please enter/confirm first name
FIRST NAME OF CHILD N
STRING

ELSE
ENDIF
IF CH005_SexOfChildN = EMPTY

CH005_SexOfChildN
Is {CH004_FirstNameOfChild} male or female?

IWER:
Ask only if unclear
SEX OF CHILD N
1. Male
2. Female

ELSE
ENDIF
IF CH006_YearOfBirthChildN = EMPTY

CH006_YearOfBirthChildN
In which year was {CH004_FirstNameOfChild} born?

IWER:
Please enter/confirm year of birth
YEAR OF BIRTH CHILD N
1875..2015

ELSE
ENDIF

ELSE

IF CH001a_ChildCheck = a2
Txt_FL_CH004

CH004_FirstNameOfChild
[(' The name ' + PreloadedName) + ' was found.']

What is the [correct] first name of [this/ your next] child?

IWER:
Please enter/confirm first name
FIRST NAME OF CHILD N
STRING

CH005_SexOfChildN
Is {CH004_FirstNameOfChild} male or female?

IWER:
Ask only if unclear

SEX OF CHILD N
1. Male
2. Female

Txt_FL_CH006

CH006_YearOfBirthChildN
In which year was {CH004_FirstNameOfChild} born?

IWER:
Please enter/confirm year of birth
YEAR OF BIRTH CHILD N
1875..2015

IF piPreloadChildIndex > 0
ELSE
ENDIF

ELSE

IF ((piPreloadChildIndex > 0) OR ((piPreloadChildIndex = 0) AND (piRosterChildIndex > 0))) AND (CH001a_ChildCheck = a6)

CH505_WhichChildMentionedEarlier

IWER:
To which child that was already mentioned earlier is {FL_CHILD_NAME} equal?
EQUAL TO WHICH CHILD
^FLChild[1]
^FLChild[2]
^FLChild[3]
^FLChild[4]
^FLChild[5]
^FLChild[6]
^FLChild[7]
^FLChild[8]
^FLChild[9]
^FLChild[10]
^FLChild[11]
^FLChild[12]
^FLChild[13]
^FLChild[14]
^FLChild[15]
^FLChild[16]
^FLChild[17]
^FLChild[18]
^FLChild[19]

IF (CH505_WhichChildMentionedEarlier = RESPONSE) AND (piRosterChildIndex > 0)
ENDIF

ENDIF

ENDIF

ENDIF
IF (CH001a_ChildCheck = a1) OR (CH001a_ChildCheck = a2)
ELSE
ENDIF

ENDBLOCK

ENDIF

ENDIF

ENDIF

ENDIF

ENDIF

*ENDLOOP*Txt_FL_CH203

CH203_Done
{Children_table}

IWER:
Please make sure that the list of children is complete. If the list is incomplete or not correct, go back by using the arrow key <-
CHILD GRID DONE
1. Continue

IF CH203_Done = a1
*ENDIF*ENDTABLETxt_FL_CH302

CH302_NatChild
[Is this child a common natural child/ Is this child a natural child/ Are all these children common natural children/ Are all these children natural children] of your own [and your current husband together/ and your current wife together/ and your current partner together/ and your current partner together]?
ALL CHILDREN NATURAL CHILD
1. Yes
5. No

IF CH302_NatChild = a5

IF NumberOFReportedChildren = 1
ELSE
Txt_FL_CH303

CH303_WhatChildren
Which of the children are not common natural children of your own [and your current husband together/ and your current wife together/ and your current partner together/ and your current partner together]?
NOT NATURAL CHILDREN
^FLChild[1]
^FLChild[2]
^FLChild[3]
^FLChild[4]
^FLChild[5]
^FLChild[6]
^FLChild[7]
^FLChild[8]
^FLChild[9]
^FLChild[10]
^FLChild[11]
^FLChild[12]
^FLChild[13]
^FLChild[14]
^FLChild[15]
^FLChild[16]
^FLChild[17]
^FLChild[18]
^FLChild[19]
^FLChild[20]

21. ^FLDefault[1]

ENDIF
LOOP i:= 1 TO 20

IF i IN CH303_WhatChildren

BLOCK

IF MN002_Person[1].MaritalStatus = a3
ELSE
Txt_FL_CH102

CH102_RNatChild
Is {FLChildName} a natural child of you?
CHILD NATURAL RESPONDENT
1. Yes
5. No

ENDIF
IF (MN002_Person[1].MaritalStatus = a1) OR (MN002_Person[1].MaritalStatus = a2)
Txt_FL_CH103

CH103_PNatChild
Is {FLChildName} a natural child of [your/ your] current [husband/ wife/ partner/ partner] [{Name of partner/spouse}]?
CHILD NATURAL PARTNER
1. Yes
5. No

ENDIF
IF NOT ((CH102_RNatChild = a1) OR (CH103_PNatChild = a1))
Txt_FL_CH104

CH104_RExChild
Is {FLChildName} a child of a former relationship of you?
CHILD FROM PREVIOUS RELATIONSHIP RESPONDENT
1. Yes
5. No

IF ((MN002_Person[1].MaritalStatus = a1) OR (MN002_Person[1].MaritalStatus = a2)) AND (CH104_RExChild = a5)
Txt_FL_CH105

CH105_PExChild
Is {FLChildName} a child of a former relationship of [your/ your] current [husband/ wife/ partner/ partner] [{Name of partner/spouse}]?
CHILD FROM PREVIOUS RELATIONSHIP PARTNER
1. Yes
5. No

ENDIF

ENDIF
IF CH102_RNatChild = a5
Txt_FL_CH106

CH106_RAdoptChild
Have you adopted {FLChildName}?

HAS BEEN ADOPTED BY RESPONDENT
1. Yes
5. No

ENDIF
IF CH103_PNatChild = a5
Txt_FL_CH107

CH107_PAdoptChild
Has [your/ your] current [husband/ wife/ partner/ partner] [{Name of partner/spouse}] adopted {FLChildName}?
HAS BEEN ADOPTED BY PARTNER
1. Yes
5. No

ENDIF
IF NOT ((((((CH102_RNatChild = a1) OR (CH103_PNatChild = a1)) OR (CH104_RExChild = a1)) OR (CH105_PExChild = a1)) OR (CH106_RAdoptChild = a1)) OR (CH107_PAdoptChild = a1))
Txt_FL_CH108

CH108_FosterChild
Is {FLChildName} a foster child?
IS FOSTERCHILD
1. Yes
5. No

ENDIF

ENDBLOCK

ENDIF

ENDLOOP

ENDIF

CH202_ChildInfoByEnum

:*TABLE Section_CH.TChildInfo*
LOOP cnt:= 1 TO 20

IF (Sec_CH.CH201_ChildByEnum.Child[cnt].CH001a_ChildCheck = a1) OR (Sec_CH.CH201_ChildByEnum.Child[cnt].CH001a_ChildCheck = a2)

BLOCK

IF piRosterChildIndex > 0
ELSE

IF Sec_CH.CH201_ChildByEnum.Child[piIndex].CONTACT = RESPONSE
ELSE
Txt_FL_CH014

CH014_ContactChild
During the past twelve months, how often did you have contact with {CH004_FirstNameOfChild}, either in person, by phone, mail, email or any other electronic means?
CONTACT WITH CHILD
1. Daily
2. Several times a week

3. About once a week
4. About every two weeks
5. About once a month
6. Less than once a month
7. Never

ENDIF

ENDIF
IF piPreloadChildIndex = '0'

IF piRosterChildIndex > 0
ELSE

IF Sec_CH.CH201_ChildByEnum.Child[piIndex].DISTANCE <> EMPTY
ELSE

CH007_ChLWh
Please look at card 5.
Where does {CH004_FirstNameOfChild} live?
WHERE DOES CHILD N LIVE
1. In the same household
2. In the same building
3. Less than 1 kilometre away
4. Between 1 and 5 kilometres away
5. Between 5 and 25 kilometres away
6. Between 25 and 100 kilometres away
7. Between 100 and 500 kilometres away
8. More than 500 kilometres away

ENDIF

ENDIF
IF piYearOfBirthChild < (YEAR (SYSDATE) - 16)

CH012_MaritalStatusChildN
Please look at card 4.
What is the marital status of {CH004_FirstNameOfChild}?
MARITAL STATUS OF CHILD
1. Married and living together with spouse
2. Registered partnership
3. Married, living separated from spouse
4. Never married
5. Divorced
6. Widowed

IF CH012_MaritalStatusChildN.ORD > 2
Txt_FL_CH013

CH013_PartnerChildN
Does {CH004_FirstNameOfChild} have a partner who lives with [him/ her]?
DOES CHILD HAVE PARTNER
1. Yes
5. No

ENDIF

ENDIF
IF CH007_ChLWh = a1
ELSE

Txt_FL_CH015

CH015_YrChldMoveHh
In which year did {CH004_FirstNameOfChild} move from the parental household?

IWER:
The last move to count. Type "2999" if child still lives at home (e.g. with divorced mother)
YEAR CHILD MOVED FROM HOUSEHOLD
1900..2999
CHECK (CH015_YrChldMoveHh >= piYearOfBirthChild) OR (CH015_YrChldMoveHh = DONTKNOW) L1 " [Year should be greater than or equal to birthyear. If year is correct, please press "suppress" and enter a remark to explain]"

ENDIF

ENDIF
IF piYearOfBirthChild < (YEAR (SYSDATE) - 16)

CH016_ChildOcc
Please look at card 6.
What is {CH004_FirstNameOfChild}'s employment status?
CHILD OCCUPATION
1. Full-time employed
2. Part-time employed
3. Self-employed or working for own family business
4. Unemployed
5. In vocational training/retraining/education
6. Parental leave
7. In retirement or early retirement
8. Permanently sick or disabled
9. Looking after home or family
97. Other

IF (piPreloadChildIndex = '0') OR piPreloadChildIndex = EMPTY
Txt_FL_CH017

CH017_EducChild
Please look at card 2.
What is the highest school leaving certificate or school degree {CH004_FirstNameOfChild} has obtained?

IWER:
If respondent mentions foreign degree/certificate, please ask if he/she can fit their degree into the given categories, if they cannot, please use the other option and type it in (next screen).
CHILD EDUCATION
1. No schooling/education at all
2. Some education, but less than [instead of put respective country specific degr.]
3. Country specific category
4. Country specific category
5. Country specific category
6. Country specific category
7. Country specific category
8. Country specific category
9. Country specific category
10. Country specific category
11. Country specific category
12. Country specific category
13. Country specific category
14. Country specific category
15. Country specific category
16. Country specific category
17. Country specific category
18. Country specific category

19. Country specific category
20. Country specific category
95. No degree yet/still in school
97. Other

CH018_EdInstChild
Please look at card 3.
Which degrees of higher education or vocational training does {CH004_FirstNameOfChild} have?

IWER:
{CodeAll}
If respondent answers 'still in education/vocational training' please ask if he/she already holds one of the other degrees on the showcard.
FURTHER EDUCATION OR VOCATIONAL TRAINING
1. No higher education/vocational training
2. Some education, but less than [ISCED 1] (instead of [ISCED 1] put respective country specific degr.)
3. Country specific category
4. Country specific category
5. Country specific category
6. Country specific category
7. Country specific category
8. Country specific category
9. Country specific category
10. Country specific category
11. Country specific category
12. Country specific category
13. Country specific category
14. Country specific category
15. Country specific category
16. Country specific category
17. Country specific category
18. Country specific category
19. Country specific category
20. Country specific category
95. Still in education/vocational training
97. Other

CH019_NoChildren
How many children - if any - does {CH004_FirstNameOfChild} have?

IWER:
Please count all natural children, fostered, adopted and stepchildren, including those of a spouse or partner
NUMBER OF CHILDREN OF CHILD
0..25

IF CH019_NoChildren > 0
Txt_FL_CH020

CH020_YrBrthYCh
In which year was the [youngest] child of {CH004_FirstNameOfChild} born?
YEAR OF BIRTH YOUNGEST CHILD
1875..2015

ENDIF

ENDIF

ENDIF

ENDBLOCK

ENDIF

*ENDLOOP*ENDTABLE
IF MN101_Longitudinal = 1

LOOP i:= 1 TO 20

IF (Sec_CH.CH201_ChildByEnum.Child[i].PRELOAD_ID <> '0') AND ((Sec_CH.CH201_ChildByEnum.Child[i].CH001a_ChildCheck = a1) OR (Sec_CH.CH201_ChildByEnum.Child[i].CH001a_ChildCheck = a2))
ENDIF

ENDLOOP
IF numberofcheckedpreloadchildren > 0
Txt_FL_CH507

CH507_IntroCheckChildren
We would like to update some of the information we have on your [child/ children].
INTRODUCTION TEXT CHILDREN CHECK
1. Continue

Txt_FL_CH524

CH524_LocationCheckChildren
Has [your child/ any of your children/ your child/ any of your children] changed residence since the interview in {FLLastInterviewMonthYear}?
CHECK LOCATION OF CHILDREN CHANGED
1. Yes
5. No

IF CH524_LocationCheckChildren = a1

IF NumberOFReportedChildren > 1
Txt_FL_CH525

CH525_LocationWhom
Which child has moved house?

IWER:
{CodeAll}
WHICH CHILD
^FLChild[1]
^FLChild[2]
^FLChild[3]
^FLChild[4]
^FLChild[5]
^FLChild[6]
^FLChild[7]
^FLChild[8]
^FLChild[9]
^FLChild[10]
^FLChild[11]
^FLChild[12]
^FLChild[13]
^FLChild[14]
^FLChild[15]
^FLChild[16]
^FLChild[17]
^FLChild[18]
^FLChild[19]
^FLChild[20]
21. ^FLDefault[1]

ENDIF
LOOP i:= 1 TO 20

IF NumberOFReportedChildren = 1

IF (Sec_CH.CH201_ChildByEnum.Child[i].CH001a_ChildCheck = a1) OR (Sec_CH.CH201_ChildByEnum.Child[i].CH001a_ChildCheck = a2)

BLOCK
Txt_FL_CH526

CH526_LocationChanged
Please look at card 5: Where does {FL_CH526_1} live?
CHILD LOCATION
1. In the same household
2. In the same building
3. Less than 1 kilometre away
4. Between 1 and 5 kilometres away
5. Between 5 and 25 kilometres away
6. Between 25 and 100 kilometres away
7. Between 100 and 500 kilometres away
8. More than 500 kilometres away

ENDBLOCK

ENDIF

ELSE

IF (i IN CH525_LocationWhom) AND (Sec_CH.CH201_ChildByEnum.Child[i].PRELOAD_ID <> '0')

BLOCK
Txt_FL_CH526

CH526_LocationChanged
Please look at card 5: Where does {FL_CH526_1} live?
CHILD LOCATION
1. In the same household
2. In the same building
3. Less than 1 kilometre away
4. Between 1 and 5 kilometres away
5. Between 5 and 25 kilometres away
6. Between 25 and 100 kilometres away
7. Between 100 and 500 kilometres away
8. More than 500 kilometres away

ENDBLOCK

ENDIF

ENDIF

ENDLOOP

ENDIF
LOOP i:= 1 TO 20

IF ildren">NumberOFReportedChildren >= i

IF Sec_CH.CH201_ChildByEnum.Child[i].PRELOAD_ID <> '0'

IF (YEAR (SYSDATE) - Sec_CH.CH201_ChildByEnum.Child[i].CH006_YearOfBirthChildN) < 22
ENDIF
IF (YEAR (SYSDATE) - Sec_CH.CH201_ChildByEnum.Child[i].CH006_YearOfBirthChildN) < 32
ENDIF
IF (YEAR (SYSDATE) - Sec_CH.CH201_ChildByEnum.Child[i].CH006_YearOfBirthChildN) > 16
ENDIF

ENDIF

ENDIF

ENDLOOP
IF a_preloaded_child_aged_smaller_22 = 1
Txt_FL_CH508

CH508_SchoolCheckChildren
Please look at card 2.
Since the interview in {FLLastInterviewMonthYear}, has [your child/ any of your children/ your child/ any of your children] obtained one of the school leaving certificates listed on this card?
CHECK SCHOOL CHANGED
1. Yes
5. No

IF CH508_SchoolCheckChildren = a1

IF NumberOFReportedChildren > 1
Txt_FL_CH509

CH509_SchoolWhom
Which child?

IWER:
{CodeAll}
WHICH CHILD
^FLChild[1]
^FLChild[2]
^FLChild[3]
^FLChild[4]
^FLChild[5]
^FLChild[6]
^FLChild[7]
^FLChild[8]
^FLChild[9]
^FLChild[10]
^FLChild[11]
^FLChild[12]
^FLChild[13]
^FLChild[14]
^FLChild[15]
^FLChild[16]
^FLChild[17]
^FLChild[18]
^FLChild[19]
^FLChild[20]
21. ^FLDefault[1]

ENDIF
LOOP i:= 1 TO 20

IF NumberOFReportedChildren = 1

IF (Sec_CH.CH201_ChildByEnum.Child[i].CH001a_ChildCheck = a1) OR

(Sec_CH.CH201_ChildByEnum.Child[i].CH001a_ChildCheck = a2)

BLOCK

Txt_FL_CH510

CH510_Leaving_certificate

What is the highest school leaving certificate or school degree that {FL_CH510_1} has obtained?

IWER:

If respondent mentions foreign degree/certificate, please ask if he/she can fit their degree into the given categories, if they cannot, please use the other option and type it in (next screen).

LEAVING_CERTIFICATE

1. No schooling/education at all
2. Some education, but less than [instead of put respective country specific degr.]
3. Country specific category
4. Country specific category
5. Country specific category
6. Country specific category
7. Country specific category
8. Country specific category
9. Country specific category
10. Country specific category
11. Country specific category
12. Country specific category
13. Country specific category
14. Country specific category
15. Country specific category
16. Country specific category
17. Country specific category
18. Country specific category
19. Country specific category
20. Country specific category
95. No degree yet/still in school
97. Other

ENDBLOCK

ENDIF

ELSE

IF (i IN CH509_SchoolWhom) AND (Sec_CH.CH201_ChildByEnum.Child[i].PRELOAD_ID <> '0')

BLOCK

Txt_FL_CH510

CH510_Leaving_certificate

What is the highest school leaving certificate or school degree that {FL_CH510_1} has obtained?

IWER:

If respondent mentions foreign degree/certificate, please ask if he/she can fit their degree into the given categories, if they cannot, please use the other option and type it in (next screen).

LEAVING_CERTIFICATE

1. No schooling/education at all
2. Some education, but less than [instead of put respective country specific degr.]
3. Country specific category
4. Country specific category
5. Country specific category
6. Country specific category
7. Country specific category
8. Country specific category
9. Country specific category
10. Country specific category

11. Country specific category
12. Country specific category
13. Country specific category
14. Country specific category
15. Country specific category
16. Country specific category
17. Country specific category
18. Country specific category
19. Country specific category
20. Country specific category
95. No degree yet/still in school
97. Other

ENDBLOCK

ENDIF

ENDIF

ENDLOOP

ENDIF

ENDIF
IF a_preloaded_child_aged_smaller_32 = 1
Txt_FL_CH511

CH511_DegreeCheckChildren
Please look at card 3.
Since the interview in {FLLastInterviewMonthYear}, has [your child/ any of your children/ your child/ any of your children] obtained one of the degrees of higher education or vocational training listed on this card?
CHECK DEGREE CHANGED
1. Yes
5. No

IF CH511_DegreeCheckChildren = a1

IF NumberOFReportedChildren > 1
Txt_FL_CH512

CH512_DegreeWhom
Which child?

IWER:
{CodeAll}
WHICH CHILD
^FLChild[1]
^FLChild[2]
^FLChild[3]
^FLChild[4]
^FLChild[5]
^FLChild[6]
^FLChild[7]
^FLChild[8]
^FLChild[9]
^FLChild[10]
^FLChild[11]
^FLChild[12]
^FLChild[13]
^FLChild[14]
^FLChild[15]

^FLChild[16]
^FLChild[17]
^FLChild[18]
^FLChild[19]
^FLChild[20]
21. ^FLDefault[1]

ENDIF
LOOP i:= 1 TO 20

IF NumberOFReportedChildren = 1

IF (Sec_CH.CH201_ChildByEnum.Child[i].CH001a_ChildCheck = a1) OR (Sec_CH.CH201_ChildByEnum.Child[i].CH001a_ChildCheck = a2)

BLOCK
Txt_FL_CH513

CH513_DegreeObtained
Which degrees of higher education or vocational training has {FL_CH513_1} obtained?

IWER:
{CodeAll}
If respondent answers <> please ask if he/she already holds one of the other degrees on the showcard.
DEGREE OBTAINED
1. No higher education/vocational training
2. Some education, but less than [ISCED 1] (instead of [ISCED 1] put respective country specific degr.)
3. Country specific category
4. Country specific category
5. Country specific category
6. Country specific category
7. Country specific category
8. Country specific category
9. Country specific category
10. Country specific category
11. Country specific category
12. Country specific category
13. Country specific category
14. Country specific category
15. Country specific category
16. Country specific category
17. Country specific category
18. Country specific category
19. Country specific category
20. Country specific category
95. Still in education/vocational training
97. Other

ENDBLOCK

ENDIF

ELSE

IF (i IN CH512_DegreeWhom) AND (Sec_CH.CH201_ChildByEnum.Child[i].PRELOAD_ID <> '0')

BLOCK
Txt_FL_CH513

CH513_DegreeObtained

Which degrees of higher education or vocational training has {FL_CH513_1} obtained?

IWER:
{CodeAll}
If respondent answers <> please ask if he/she already holds one of the other degrees on the showcard.
DEGREE OBTAINED
1. No higher education/vocational training
2. Some education, but less than [ISCED 1] (instead of [ISCED 1] put respective country specific degr.)
3. Country specific category
4. Country specific category
5. Country specific category
6. Country specific category
7. Country specific category
8. Country specific category
9. Country specific category
10. Country specific category
11. Country specific category
12. Country specific category
13. Country specific category
14. Country specific category
15. Country specific category
16. Country specific category
17. Country specific category
18. Country specific category
19. Country specific category
20. Country specific category
95. Still in education/vocational training
97. Other

ENDBLOCK

ENDIF

ENDIF

ENDLOOP

ENDIF

ENDIF
IF a_preloaded_child_aged_bigger_16 = 1
Txt_FL_CH514

CH514_MaritalStatusCheckChildren
Since the interview in {FLLastInterviewMonthYear}, has [your child/ any of your children/ your child/ any of your children] changed his or her marital status?
CHECK MARITAL STATUS CHANGED
1. Yes
5. No

IF CH514_MaritalStatusCheckChildren = a1

IF NumberOFReportedChildren > 1
Txt_FL_CH515

CH515_MaritalStatusWhom
Which child has changed his or her marital status?

IWER:
{CodeAll}

WHICH CHILD
^FLChild[1]
^FLChild[2]
^FLChild[3]
^FLChild[4]
^FLChild[5]
^FLChild[6]
^FLChild[7]
^FLChild[8]
^FLChild[9]
^FLChild[10]
^FLChild[11]
^FLChild[12]
^FLChild[13]
^FLChild[14]
^FLChild[15]
^FLChild[16]
^FLChild[17]
^FLChild[18]
^FLChild[19]
^FLChild[20]
21. ^FLDefault[1]

ENDIF
LOOP i:= 1 TO 20

IF NumberOFReportedChildren = 1

IF (Sec_CH.CH201_ChildByEnum.Child[i].CH001a_ChildCheck = a1) OR (Sec_CH.CH201_ChildByEnum.Child[i].CH001a_ChildCheck = a2)

BLOCK
Txt_FL_CH516

CH516_MaritalStatus
Please look at card 4. What is [{Name of child}] 's marital status?
MARITAL STATUS
1. Married and living together with spouse
2. Registered partnership
3. Married, living separated from spouse
4. Never married
5. Divorced
6. Widowed

ENDBLOCK

ENDIF

ELSE

IF (i IN CH515_MaritalStatusWhom) AND (Sec_CH.CH201_ChildByEnum.Child[i].PRELOAD_ID <> '0')

BLOCK
Txt_FL_CH516

CH516_MaritalStatus
Please look at card 4. What is [{Name of child}] 's marital status?
MARITAL STATUS
1. Married and living together with spouse
2. Registered partnership
3. Married, living separated from spouse
4. Never married

5. Divorced
6. Widowed

ENDBLOCK

ENDIF

ENDIF

ENDLOOP

*ENDIF*Txt_FL_CH517

CH517_BecomeParent
Since the interview in {FLLastInterviewMonthYear}, has [your child/ any of your children/ your child/ any of your children] become parent of a new child?

IWER:
Please include natural children, fostered, adopted and stepchildren, including those of a spouse or partner.
CHECK GRANDCHILDREN CHANGED
1. Yes
5. No

IF CH517_BecomeParent = a1

IF NumberOFReportedChildren > 1
Txt_FL_CH518

CH518_ParentWhom
Which child has become parent of a new child?

IWER:
Check all children that apply
WHICH CHILD
^FLChild[1]
^FLChild[2]
^FLChild[3]
^FLChild[4]
^FLChild[5]
^FLChild[6]
^FLChild[7]
^FLChild[8]
^FLChild[9]
^FLChild[10]
^FLChild[11]
^FLChild[12]
^FLChild[13]
^FLChild[14]
^FLChild[15]
^FLChild[16]
^FLChild[17]
^FLChild[18]
^FLChild[19]
^FLChild[20]
21. ^FLDefault[1]

ENDIF
LOOP i:= 1 TO 20

IF NumberOFReportedChildren = 1

IF (Sec_CH.CH201_ChildByEnum.Child[i].CH001a_ChildCheck = a1) OR (Sec_CH.CH201_ChildByEnum.Child[i].CH001a_ChildCheck = a2)

BLOCK

Txt_FL_CH519

CH519_NewK

How many children does [{Name of child}] have altogether?

HOW MANY NEW CHILDREN

0..25

IF CH519_NewK > 0

Txt_FL_CH520

CH520_YoungestBorn

In which year was [this child/ the youngest of these children] born?

YOUNGEST BORN

1900..2015

ENDIF

ENDBLOCK

ENDIF

ELSE

IF (i IN CH518_ParentWhom) AND (Sec_CH.CH201_ChildByEnum.Child[i].PRELOAD_ID <> '0')

BLOCK

Txt_FL_CH519

CH519_NewK

How many children does [{Name of child}] have altogether?

HOW MANY NEW CHILDREN

0..25

IF CH519_NewK > 0

Txt_FL_CH520

CH520_YoungestBorn

In which year was [this child/ the youngest of these children] born?

YOUNGEST BORN

1900..2015

ENDIF

ENDBLOCK

ENDIF

ENDIF

ENDLOOP

ENDIF

ENDIF

ENDIF

ENDIF

*ENDIF*Txt_FL_CH021

CH021_NoGrandChild
Talking about grandchildren, how many grandchildren do you [and your/ and your/ and your/ and your] [husband/ wife/ partner/ partner] have altogether?

IWER:
Include grandchildren from previous relationships
NUMBER OF GRANDCHILDREN
-1000000000000000000..1000000000000000000

IF CH021_NoGrandChild > 0
Txt_FL_CH022

CH022_GreatGrChild
Do you [or your/ or your/ or your/ or your] [husband/ wife/ partner/ partner] have any great-grandchildren?
HAS GREAT-GRANDCHILDREN
1. Yes
5. No

ENDIF

CH023_IntCheck

IWER:
CHECK:
Who answered the questions in this section?
WHO ANSWERED QUESTIONS IN SECTION CH
1. Respondent only
2. Respondent and proxy
3. Proxy only

ELSE

LOOP i:= 1 TO 20

IF NOT (Preload.PartnerChildren[i].Name = '')

IF Preload.PartnerChildren[i].Gender = a1
ELSE

IF Preload.PartnerChildren[i].Gender = a2
ELSE
ENDIF

ENDIF

ENDIF

ENDLOOP
LOOP i:= 1 TO 7

IF num_of_children < 20

IF Sec_SN.SocialNetworkInfo[i].SN018_PreloadMatch = a96

IF Sec_SN.SocialNetworkInfo[i].SN005a_Gender = a1
ELSE

IF Sec_SN.SocialNetworkInfo[i].SN005a_Gender = a2
ELSE

ENDIF

ENDIF

ENDIF

ENDIF

ENDLOOP

ENDIF

ENDBLOCK

ENDIF
IF (PH IN Test) OR (ALL IN Test)

BLOCK
Txt_FL_PH001

PH001_Intro
Now I have some questions about your health.
INTRO HEALTH
1. Continue

System preset value PH901_TimeStampStart
IF PH901_TimeStampStart = EMPTY AND PH001_Intro <> EMPTY
ENDIF
BLOCK

System preset value PH199_Random
IF PH199_Random = EMPTY
*ENDIF*Txt_FL_PH003

PH003_HealthGen2
Would you say your health is...

IWER:
{ReadOut}
HEALTH IN GENERAL QUESTION 2
1. Excellent
2. Very good
3. Good
4. Fair
5. Poor

Txt_FL_PH004

PH004_LStIll
Some people suffer from chronic or long-term health problems. By chronic or long-term we mean it has troubled you over a period of time or is likely to affect you over a period of time. Do you have any such health problems, illness, disability or infirmity?

IWER:
Including mental health problems
LONG-TERM ILLNESS
1. Yes
5. No

Txt_FL_PH005

PH005_LimAct
For the past six months at least, to what extent have you been limited because of a health problem in activities

people usually do?

IWER:
{ReadOut}
LIMITED ACTIVITIES
1. Severely limited
2. Limited, but not severely
3. Not limited

IF (MN808_AgeRespondent <= 75) AND (MN024_NursingHome = a1)
Txt_FL_PH061

PH061_LimPaidWork
Do you have any health problem or disability that limits the kind or amount of paid work you can do?
PROBLEM THAT LIMITS PAID WORK
1. Yes
5. No

*ENDIF*Txt_FL_PH006

PH006_DocCond
Please look at card 8.
[Has a doctor ever told you that you had/ Do you currently have] any of the conditions on this card? [With this we mean that a doctor has told you that you have this condition, and that you are either currently being treated for or bothered by this condition.] Please tell me the number or numbers of the conditions.

IWER:
{CodeAll}
DOCTOR TOLD YOU HAD CONDITIONS
1. A heart attack including myocardial infarction or coronary thrombosis or any other heart problem including congestive heart failure
2. High blood pressure or hypertension
3. High blood cholesterol
4. A stroke or cerebral vascular disease
5. Diabetes or high blood sugar
6. Chronic lung disease such as chronic bronchitis or emphysema
10. Cancer or malignant tumour, including leukaemia or lymphoma, but excluding minor skin cancers
11. Stomach or duodenal ulcer, peptic ulcer
12. Parkinson disease
13. Cataracts
14. Hip fracture
15. Other fractures
16. Alzheimer's disease, dementia, organic brain syndrome, senility or any other serious memory impairment
18. Other affective or emotional disorders, including anxiety, nervous or psychiatric problems
19. Rheumatoid Arthritis
20. Osteoarthritis, or other rheumatism
21. Chronic kidney disease
96. None
97. Other conditions, not yet mentioned

CHECK NOT ((PH006_DocCond.CARDINAL > 1) AND (96 IN PH006_DocCond)) L1 " [You cannot select ""None of the above"" together with any other answer. Please change your answer]"

IF a97 IN PH006_DocCond
Txt_FL_PH007

PH007_OthCond
What other conditions have you had?

IWER:
Probe
OTHER CONDITIONS

STRING

ENDIF
LOOP cnt:= 1 TO 21

IF cnt IN PH006_DocCond

BLOCK

IF piIndexSub = 10
Txt_FL_PH008

PH008_OrgCan
In which organ or part of the body do you have or have you had cancer?

IWER:
{CodeAll}
CANCER IN WHICH ORGANS
1. Brain
2. Oral cavity
3. Larynx
4. Other pharynx
5. Thyroid
6. Lung
7. Breast
8. Oesophagus
9. Stomach
10. Liver
11. Pancreas
12. Kidney
13. Prostate
14. Testicle
15. Ovary
16. Cervix
17. Endometrium
18. Colon or rectum
19. Bladder
20. Skin
21. Lymphoma
22. Leukemia
97. Other organ

ENDIF
IF MN101_Longitudinal = 0
Txt_FL_PH009

PH009_AgeCond
About how old were you when you were first told by a doctor that you had [a heart attack or any other heart problem/ high blood pressure/ high blood cholesterol/ a stroke or cerebral vascular disease/ diabetes or high blood sugar/ chronic lung disease/ cancer/ stomach or duodenal ulcer/ parkinson disease/ cataracts/ hip fracture/ other fractures/ Alzheimer""s disease, dementia or other serious memory impairment/ Affective or emotional disorders/ Rheumatoid Arthritis/ Osteoarthritis, or other rheumatism/ Chronic kidney disease]?
AGE WHEN CONDITION STARTED
0..125
CHECK NOT (PH009_AgeCond > MN808_AgeRespondent) L1 " [Age should be less than or equal to respondent""s age]"

ENDIF

ENDBLOCK

ENDIF

ENDLOOP
IF 97.00000000000001 IN [PH006_DocCond]

BLOCK

IF [piIndexSub] = 10
Txt_FL_PH008

PH008_OrgCan
In which organ or part of the body do you have or have you had cancer?

IWER:
{CodeAll}
CANCER IN WHICH ORGANS
1. Brain
2. Oral cavity
3. Larynx
4. Other pharynx
5. Thyroid
6. Lung
7. Breast
8. Oesophagus
9. Stomach
10. Liver
11. Pancreas
12. Kidney
13. Prostate
14. Testicle
15. Ovary
16. Cervix
17. Endometrium
18. Colon or rectum
19. Bladder
20. Skin
21. Lymphoma
22. Leukemia
97. Other organ

ENDIF
IF MN101_Longitudinal = 0
Txt_FL_PH009

PH009_AgeCond
About how old were you when you were first told by a doctor that you had [a heart attack or any other heart problem/ high blood pressure/ high blood cholesterol/ a stroke or cerebral vascular disease/ diabetes or high blood sugar/ chronic lung disease/ cancer/ stomach or duodenal ulcer/ parkinson disease/ cataracts/ hip fracture/ other fractures/ Alzheimer""s disease, dementia or other serious memory impairment/ Affective or emotional disorders/ Rheumatoid Arthritis/ Osteoarthritis, or other rheumatism/ Chronic kidney disease]?
AGE WHEN CONDITION STARTED
0..125
CHECK NOT (PH009_AgeCond > MN808_AgeRespondent) L1 " [Age should be less than or equal to respondent""s age]"

ENDIF

ENDBLOCK

ENDIF
IF MN101_Longitudinal = 1

BLOCK

Txt_FL_PH072

PH072_HadCondition
[For a few conditions, we would like to know exactly what has happened in the past couple of years. @/]
Since our interview in {FLLastInterviewMonthYear} have you [had a heart attack/ had a stroke or been diagnosed with cerebral vascular disease/ been diagnosed with cancer/ suffered a hip fracture]?
HAD CONDITION
1. Yes
5. No

IF PH072_HadCondition = a1

IF piIndex = 3
Txt_FL_PH080

PH080_OrgCan
In which organ or part of the body do you have or have you had cancer?

IWER:
{CodeAll}
CANCER IN WHICH ORGANS
1. Brain
2. Oral cavity
3. Larynx
4. Other pharynx
5. Thyroid
6. Lung
7. Breast
8. Oesophagus
9. Stomach
10. Liver
11. Pancreas
12. Kidney
13. Prostate
14. Testicle
15. Ovary
16. Cervix
17. Endometrium
18. Colon or rectum
19. Bladder
20. Skin
21. Lymphoma
22. Leukemia
97. Other organ

*ENDIF*Txt_FL_PH076

PH076_YearCondition
In what year was your most recent [heart attack/ stroke or cerebral vascular disease/ cancer/ hip fracture]?
YEAR MOST RECENT CONDITION
1. 2006
2. 2007
3. 2008
4. 2009
5. 2010
6. 2011
7. 2012
8. 2013
9. 2014
10. 2015

PH077_MonthCondition
In what month was that?
MONTH MOST RECENT CONDITION
1. January
2. February
3. March
4. April
5. May
6. June
7. July
8. August
9. September
10. October
11. November
12. December

Txt_FL_PH071

PH071_HadConditionHowMany
How many [heart attacks/ strokes or cerebral vascular diseases/ cancers/ hip fractures] have you had since we talked to you in {FLLastInterviewMonthYear}?
HOW MANY
1. 1
2. 2
3. 3 or more

ENDIF

ENDBLOCK
BLOCK
Txt_FL_PH072

PH072_HadCondition
[For a few conditions, we would like to know exactly what has happened in the past couple of years. @/] Since our interview in {FLLastInterviewMonthYear} have you [had a heart attack/ had a stroke or been diagnosed with cerebral vascular disease/ been diagnosed with cancer/ suffered a hip fracture]?
HAD CONDITION
1. Yes
5. No

IF PH072_HadCondition = a1

IF piIndex = 3
Txt_FL_PH080

PH080_OrgCan
In which organ or part of the body do you have or have you had cancer?

IWER:
{CodeAll}
CANCER IN WHICH ORGANS
1. Brain
2. Oral cavity
3. Larynx
4. Other pharynx
5. Thyroid
6. Lung
7. Breast
8. Oesophagus
9. Stomach
10. Liver
11. Pancreas

12. Kidney
13. Prostate
14. Testicle
15. Ovary
16. Cervix
17. Endometrium
18. Colon or rectum
19. Bladder
20. Skin
21. Lymphoma
22. Leukemia
97. Other organ

*ENDIF*Txt_FL_PH076

PH076_YearCondition
In what year was your most recent [heart attack/ stroke or cerebral vascular disease/ cancer/ hip fracture]?
YEAR MOST RECENT CONDITION
1. 2006
2. 2007
3. 2008
4. 2009
5. 2010
6. 2011
7. 2012
8. 2013
9. 2014
10. 2015

PH077_MonthCondition
In what month was that?
MONTH MOST RECENT CONDITION
1. January
2. February
3. March
4. April
5. May
6. June
7. July
8. August
9. September
10. October
11. November
12. December

Txt_FL_PH071

PH071_HadConditionHowMany
How many [heart attacks/ strokes or cerebral vascular diseases/ cancers/ hip fractures] have you had since we talked to you in {FLLastInterviewMonthYear}?
HOW MANY
1. 1
2. 2
3. 3 or more

ENDIF

ENDBLOCK
BLOCK
Txt_FL_PH072

PH072_HadCondition
[For a few conditions, we would like to know exactly what has happened in the past couple of years. @/]
Since our interview in {FLLastInterviewMonthYear} have you [had a heart attack/ had a stroke or been diagnosed with cerebral vascular disease/ been diagnosed with cancer/ suffered a hip fracture]?
HAD CONDITION
1. Yes
5. No

IF PH072_HadCondition = a1

IF piIndex = 3
Txt_FL_PH080

PH080_OrgCan
In which organ or part of the body do you have or have you had cancer?

IWER:
{CodeAll}
CANCER IN WHICH ORGANS
1. Brain
2. Oral cavity
3. Larynx
4. Other pharynx
5. Thyroid
6. Lung
7. Breast
8. Oesophagus
9. Stomach
10. Liver
11. Pancreas
12. Kidney
13. Prostate
14. Testicle
15. Ovary
16. Cervix
17. Endometrium
18. Colon or rectum
19. Bladder
20. Skin
21. Lymphoma
22. Leukemia
97. Other organ

*ENDIF*Txt_FL_PH076

PH076_YearCondition
In what year was your most recent [heart attack/ stroke or cerebral vascular disease/ cancer/ hip fracture]?
YEAR MOST RECENT CONDITION
1. 2006
2. 2007
3. 2008
4. 2009
5. 2010
6. 2011
7. 2012
8. 2013
9. 2014
10. 2015

PH077_MonthCondition
In what month was that?

MONTH MOST RECENT CONDITION
1. January
2. February
3. March
4. April
5. May
6. June
7. July
8. August
9. September
10. October
11. November
12. December

Txt_FL_PH071

PH071_HadConditionHowMany
How many [heart attacks/ strokes or cerebral vascular diseases/ cancers/ hip fractures] have you had since we talked to you in {FLLastInterviewMonthYear}?
HOW MANY
1. 1
2. 2
3. 3 or more

ENDIF

ENDBLOCK
BLOCK

Txt_FL_PH072

PH072_HadCondition
[For a few conditions, we would like to know exactly what has happened in the past couple of years. @/] Since our interview in {FLLastInterviewMonthYear} have you [had a heart attack/ had a stroke or been diagnosed with cerebral vascular disease/ been diagnosed with cancer/ suffered a hip fracture]?
HAD CONDITION
1. Yes
5. No

IF PH072_HadCondition = a1

IF piIndex = 3

Txt_FL_PH080

PH080_OrgCan
In which organ or part of the body do you have or have you had cancer?

IWER:
{CodeAll}
CANCER IN WHICH ORGANS
1. Brain
2. Oral cavity
3. Larynx
4. Other pharynx
5. Thyroid
6. Lung
7. Breast
8. Oesophagus
9. Stomach
10. Liver
11. Pancreas
12. Kidney
13. Prostate

14. Testicle
15. Ovary
16. Cervix
17. Endometrium
18. Colon or rectum
19. Bladder
20. Skin
21. Lymphoma
22. Leukemia
97. Other organ

*ENDIF*Txt_FL_PH076

PH076_YearCondition
In what year was your most recent [heart attack/ stroke or cerebral vascular disease/ cancer/ hip fracture]?
YEAR MOST RECENT CONDITION
1. 2006
2. 2007
3. 2008
4. 2009
5. 2010
6. 2011
7. 2012
8. 2013
9. 2014
10. 2015

PH077_MonthCondition
In what month was that?
MONTH MOST RECENT CONDITION
1. January
2. February
3. March
4. April
5. May
6. June
7. July
8. August
9. September
10. October
11. November
12. December

Txt_FL_PH071

PH071_HadConditionHowMany
How many [heart attacks/ strokes or cerebral vascular diseases/ cancers/ hip fractures] have you had since we talked to you in {FLLastInterviewMonthYear}?
HOW MANY
1. 1
2. 2
3. 3 or more

ENDIF

ENDBLOCK

*ENDIF*Txt_FL_PH089

PH089_Frailty_Symptoms
Please look at card 9.

For the past six months at least, have you been bothered by any of the health conditions on this card? Please tell me the number or numbers.

IWER:
{CodeAll}
BOTHERED BY SYMPTOMS
1. Falling down
2. Fear of falling down
3. Dizziness, faints or blackouts
4. Fatigue
96. None

CHECK NOT ((PH089_Frailty_Symptoms.CARDINAL > 1) AND (96 IN PH089_Frailty_Symptoms)) L1 " [You cannot select ""None of the above"" together with any other answer. Please change your answer]"
Txt_FL_PH084

PH084_TroubledPain
Are you troubled with pain?
TROUBLED BY PAIN
1. Yes
5. No

IF PH084_TroubledPain = a1

PH085_PainLevel
How bad is the pain most of the time? Is it..

IWER:
{ReadOut}
HOW BAD PAIN
1. Mild
3. Moderate
5. Severe

Txt_FL_PH087

PH087_PainJointLoc
Look at card 10.
In which parts of the body do you feel pain?

IWER:
{CodeAll}
SIX MONTHS BOTHERED BY PAIN
1. Back
2. Hips
3. Knees
4. Other joints
5. Mouth/Teeth
6. Other parts of the body, but not joints
7. All over

CHECK NOT ((PH087_PainJointLoc.CARDINAL > 1) AND (7 IN PH087_PainJointLoc)) L1 " {FLError[49]}"

*ENDIF*Txt_FL_PH011

PH011_CurrentDrugs
Our next question is about the medication you may be taking. Please look at card 11. Do you currently take drugs @bat least once a week@b for problems mentioned on this card?

IWER:
{CodeAll}
CURRENT DRUGS AT LEAST ONCE A WEEK
1. Drugs for high blood cholesterol

2. Drugs for high blood pressure
3. Drugs for coronary or cerebrovascular diseases
4. Drugs for other heart diseases
6. Drugs for diabetes
7. Drugs for joint pain or for joint inflammation
8. Drugs for other pain (e.g. headache, back pain, etc.)
9. Drugs for sleep problems
10. Drugs for anxiety or depression
11. Drugs for osteoporosis
13. Drugs for stomach burns
14. Drugs for chronic bronchitis
15. Drugs for suppressing inflammation (only glucocorticoids or steroids)
96. None
97. Other drugs, not yet mentioned

CHECK NOT ((PH011_CurrentDrugs.CARDINAL > 1) AND (96 IN PH011_CurrentDrugs)) L1 " [You cannot select ""None of the above"" together with any other answer. Please change your answer]"

IF NOT (96 IN PH011_CurrentDrugs)

Txt_FL_PH082

PH082_PolyPharmacy

Do you take at least five @Bdifferent@B drugs on a typical day?
Please include drugs prescribed by your doctor, drugs you buy without prescription, and dietary supplements such as vitamins and minerals.
AT LEAST FIVE PER DAY

1. Yes
5. No

*ENDIF*Txt_FL_PH012

PH012_Weight

Approximately how much do you weigh?

IWER:
Weight in kilos (in UK stone-dot-pounds)
WEIGHT OF RESPONDENT
0..250

CHECK NOT (((PH012_Weight >= 125) OR (PH012_Weight <= 40)) AND (PH012_Weight = RESPONSE)) L1 " [Please confirm: Respondent weights] {PH012_Weight} [kilos, is that correct If no, go back to previous answer and correct the answer.]"

Txt_FL_PH065

PH065_CheckLossWeight

Have you lost any weight during the last 12 months?
CHECK LOSS WEIGHT

1. Yes
5. No

IF PH065_CheckLossWeight = a1

Txt_FL_PH095

PH095_HowMuchLostWeight

How much weight did you lose?

IWER:
Only lost weight in whole KG e.g. 1 kg 2 kg 3 kg and so forth
HOW MUCH LOSS WEIGHT
1..50

Txt_FL_PH066

PH066_ReasonLostWeight

Why did you lose weight?

IWER:
{ReadOut}
REASON LOST WEIGHT
1. Due to illness
2. You followed a special diet
3. Due to both illness and followed a special diet
97. Other reasons for weight loss

*ENDIF*Txt_FL_PH013

PH013_HowTall
How tall are you?

IWER:
Length in centimetres (in UK: feet-dot-inches)
HOW TALL ARE YOU?
60..230
CHECK NOT (((PH013_HowTall >= 200) OR (PH013_HowTall <= 130)) AND (PH013_HowTall = RESPONSE))
L1 " {FLError[41]} {PH013_HowTall} {FLError[46]}"

ENDBLOCK
BLOCK
Txt_FL_PH041

PH041_UseGlasses
Do you usually wear glasses or contact lenses?

IWER:
all types of glasses, also glasses used only for reading.
USE GLASSES
1. Yes
5. No

IF PH041_UseGlasses = a1
Txt_FL_PH690

PH690_BifocGlasLenses
What type of glasses or contact lenses do you wear?

IWER:
{CodeAll} {ReadOut}
USE BIFOCAL GLASSES/LENSES
1. Bifocals or progressive glasses or contact lenses
2. Reading glasses or contact lenses (single vision glasses)
3. Distance glasses or contact lenses (single vision glasses)
4. Other glasses or contact lenses

IF ((a1 IN PH690_BifocGlasLenses) OR (a2 IN PH690_BifocGlasLenses)) OR (a4 IN PH690_BifocGlasLenses)
ENDIF

ENDIF
IF (PH041_UseGlasses = a5) OR ((a2 IN PH690_BifocGlasLenses) AND (PH690_BifocGlasLenses.CARDINAL = 1))
Txt_FL_PH043

PH043_EyeSightDist
How good is your eyesight for seeing things at a distance, like recognising a friend across the street {FL_PH043_1}? Would you say it is...

IWER:
{ReadOut}
EYESIGHT DISTANCE
1. Excellent
2. Very good
3. Good
4. Fair
5. Poor

ELSE
Txt_FL_PH043

PH043_EyeSightDist
How good is your eyesight for seeing things at a distance, like recognising a friend across the street {FL_PH043_1}? Would you say it is...

IWER:
{ReadOut}
EYESIGHT DISTANCE
1. Excellent
2. Very good
3. Good
4. Fair
5. Poor

ENDIF
IF (PH041_UseGlasses = a5) OR ((a3 IN PH690_BifocGlasLenses) AND (PH690_BifocGlasLenses.CARDINAL = 1))
Txt_FL_PH044

PH044_EyeSightPap
How good is your eyesight for seeing things up close, like reading ordinary newspaper print {FL_PH044_1}? Would you say it is...

IWER:
{ReadOut}
EYESIGHT READING
1. Excellent
2. Very good
3. Good
4. Fair
5. Poor

ELSE
Txt_FL_PH044

PH044_EyeSightPap
How good is your eyesight for seeing things up close, like reading ordinary newspaper print {FL_PH044_1}? Would you say it is...

IWER:
{ReadOut}
EYESIGHT READING
1. Excellent
2. Very good
3. Good
4. Fair
5. Poor

*ENDIF*Txt_FL_PH045

PH045_UseHearingAid
Are you usually wearing a hearing aid?
USE HEARING AID
1. Yes
5. No

Txt_FL_PH046

PH046_Hearing
Is your hearing [using a hearing aid as usual]...

IWER:
{ReadOut}
HEARING
1. Excellent
2. Very good
3. Good
4. Fair
5. Poor

Txt_FL_PH048

PH048_HeADLa
Please look at card 12.
Please tell me whether you have any difficulty doing each of the everyday activities on this card. Exclude any difficulties that you expect to last less than three months.

IWER:
Probe: any others?
{CodeAll}
HEALTH AND ACTIVITIES
1. Walking 100 metres
2. Sitting for about two hours
3. Getting up from a chair after sitting for long periods
4. Climbing several flights of stairs without resting
5. Climbing one flight of stairs without resting
6. Stooping, kneeling, or crouching
7. Reaching or extending your arms above shoulder level
8. Pulling or pushing large objects like a living room chair
9. Lifting or carrying weights over 10 pounds/5 kilos, like a heavy bag of groceries
10. Picking up a small coin from a table
96. None of these

CHECK NOT ((PH048_HeADLa.CARDINAL > 1) AND (96 IN PH048_HeADLa)) L1 " [You cannot select ""None of the above"" together with any other answer. Please change your answer]"
Txt_FL_PH049

PH049_HeADLb
Please look at card 13.
Please tell me if you have any difficulty with these activities because of a physical, mental, emotional or memory problem. Again exclude any difficulties you expect to last less than three months.

IWER:
Probe: any others?
{CodeAll}
MORE HEALTH AND ACTIVITIES
1. Dressing, including putting on shoes and socks
2. Walking across a room
3. Bathing or showering
4. Eating, such as cutting up your food
5. Getting in or out of bed
6. Using the toilet, including getting up or down

7. Using a map to figure out how to get around in a strange place
8. Preparing a hot meal
9. Shopping for groceries
10. Making telephone calls
11. Taking medications
12. Doing work around the house or garden
13. Managing money, such as paying bills and keeping track of expenses
14. Leaving the house independently and accessing transportation services
15. Doing personal laundry
96. None of these

CHECK NOT ((PH049_HeADLb.CARDINAL > 1) AND (96 IN PH049_HeADLb)) L1 " [You cannot select ""None of the above"" together with any other answer. Please change your answer]"

IF NOT ((((96 IN PH048_HeADLa) OR (PH048_HeADLa = DONTKNOW)) OR (PH048_HeADLa = REFUSAL)) AND (((96 IN PH049_HeADLb) OR (PH049_HeADLb = DONTKNOW)) OR (PH049_HeADLb = REFUSAL)))

Txt_FL_PH050

PH050_HelpAct

Thinking about the activities that you have problems with, does anyone ever help you with these activities?

IWER:

Including your partner or other people in your household

HELP ACTIVITIES

1. Yes
5. No

IF PH050_HelpAct = a1

Txt_FL_PH051

PH051_HelpMeetsN

Would you say that the help you receive meets your needs?

IWER:

{ReadOut}

HELP MEETS NEEDS

1. All the time
2. Usually
3. Sometimes
4. Hardly ever

*ENDIF*Txt_FL_PH059

PH059_UseAids

Please look at card 14. Do you use any of the items listed on this card?

IWER:

No. 7. Only include personal alarms used to call for assistance after falls etc.

USE OF AIDS

1. A cane or walking stick
2. A zimmer frame or walker
3. A manual wheelchair
4. An electric wheelchair
5. A buggy or scooter
6. Special eating utensils
7. A personal alarm
8. Bars, grabs, rails (to facilitate movements and to keep ones balance)
9. Raised toilet seat with/without arms
10. Incontinence pads
96. None of these
97. other items (specify)

CHECK NOT ((PH059_UseAids.CARDINAL > 1) AND (96 IN PH059_UseAids)) L1 " [You cannot select ""None of the above"" together with any other answer. Please change your answer]"

IF a97 IN PH059_UseAids

PH659_UseAidsOther
What other items?
USE OF AIDS
STRING

ENDIF

ENDIF

ENDBLOCK

PH054_IntCheck

IWER:
CHECK:
Who answered the questions in this section?
WHO ANSWERED THE QUESTIONS IN PH
1. Respondent only
2. Respondent and proxy
3. Proxy only

System preset value PH902_TimeStampEnd
IF PH902_TimeStampEnd = EMPTY AND PH054_IntCheck <> EMPTY
ENDIF

ENDBLOCK

ENDIF
IF (BR IN Test) OR (ALL IN Test)

BLOCK

IF MN101_Longitudinal = 0
Txt_FL_BR001

BR001_EverSmokedDaily
The following questions are about smoking and drinking alcoholic beverages. Have you ever smoked cigarettes, cigars, cigarillos
or a pipe daily for a period of at least one year?
EVER SMOKED DAILY
1. Yes
5. No

IF BR001_EverSmokedDaily = a1
Txt_FL_BR002

BR002_StillSmoking
[The following questions are about smoking and drinking alcoholic beverages.]
Do you smoke at the present time?
SMOKE AT THE PRESENT TIME
1. Yes
5. No

Txt_FL_BR003

BR003_HowManyYearsSmoked

For how many years have you smoked all together?

IWER:
Don't include periods without smoking
Code 1 if respondent smoked for less than one year
HOW MANY YEARS SMOKED
1..99

IF ((BR002_StillSmoking = a1) OR ((MN101_Longitudinal = 0) AND (BR002_StillSmoking = a5))) OR ((MN101_Longitudinal = 1) AND (BR002_StillSmoking = a5))

Txt_FL_BR005

BR005_WhatSmoke
What [do/ did] [you/ you] [smoke/ smoke before you stopped]?

IWER:
Cigarettes include 'roll-your-own'. {ReadOut} {CodeAll}
WHAT DO OR DID YOU SMOKE
1. Cigarettes
2. Pipe
3. Cigars or cigarillos
4. E-cigarettes with nicotine solution.

IF 1 IN BR005_WhatSmoke

Txt_FL_BR006

BR006_AmManCig
How many cigarettes [do/ did] [you/ you] [smoke/ smoke] on average per day?
AVERAGE AMOUNT OF CIGARETTES PER DAY
0..120

ENDIF

ENDIF

ENDIF

ENDIF
System preset value BR901_TimeStampStart
IF BR901_TimeStampStart = EMPTY AND (BR001_EverSmokedDaily <> EMPTY OR BR039_Drinklastsevendays <> EMPTY)
*ENDIF*Txt_FL_BR039

BR039_Drinklastsevendays
During the last 7 days, have you had at least one alcoholic beverage?
ANY DRINK LAST SEVEN DAYS
1. Yes
5. No

IF BR039_Drinklastsevendays = a1

Txt_FL_BR040

BR040_Drinklastsevendays
Please look at card 15, which shows standard units of alcoholic beverages. During the last 7 days, overall how many units of alcoholic beverages did you have?

IWER:
Please open the booklet and calculate the No. of units/week together with the respondent. If none, please enter '0'. Please round to the nearest whole number.
HOW OFTEN DRINKS LAST SEVEN DAYS
0..70

*ENDIF*Txt_FL_BR623

BR623_SixOrMoreDrinks
Please look at card 16.
In the last three months, how often did you have six or more units of alcoholic beverages on one occasion?
HOW OFTEN SIX OR MORE DRINKS LAST 3 MONTHS
1. Daily or almost daily
2. Five or six days a week
3. Three or four days a week
4. Once or twice a week
5. Once or twice a month
6. Less than once a month
7. Not at all in the last 3 months

Txt_FL_BR015

BR015_PartInVigSprtsAct
We would like to know about the type and amount of physical activity you do in your daily life. How often do you engage in @Bvigorous physical activity@B, such as sports, heavy housework, or a job that involves physical labour?

IWER:
{ReadOut}
SPORTS OR ACTIVITIES THAT ARE VIGOROUS
1. More than once a week
2. Once a week
3. One to three times a month
4. Hardly ever, or never

Txt_FL_BR016

BR016_ModSprtsAct
How often do you engage in activities that require a @Bmoderate@B level of energy such as gardening, cleaning the car, or doing a walk?

IWER:
{ReadOut}
ACTIVITIES REQUIRING A MODERATE LEVEL OF ENERGY
1. More than once a week
2. Once a week
3. One to three times a month
4. Hardly ever, or never

IF MN101_Longitudinal = 0
Txt_FL_BR026

BR026_DairyProd
Please look at card 17. In a regular @bweek@b, how often do you have a serving of dairy products such as a glass of milk, cheese in a sandwich, a cup of yogurt or a can of high protein supplement?
HOW OFTEN SERVING OF DAIRY PRODUCTS
1. Every day
2. 3-6 times a week
3. Twice a week
4. Once a week
5. Less than once a week

Txt_FL_BR027

BR027_LegumesEggs
(Please look at card 17.) In a regular week, how often do you have a serving of legumes, beans or eggs?
HOW OFTEN A WEEK SERVING OF LEGUMES OR EGGS
1. Every day
2. 3-6 times a week

3. Twice a week
4. Once a week
5. Less than once a week

Txt_FL_BR028

BR028_MeatWeek
(Please look at card 17.) In a regular week, how often do you eat meat, fish or poultry?
HOW OFTEN A DAY DO YOU EAT MEAT, FISH OR POULTRY
1. Every day
2. 3-6 times a week
3. Twice a week
4. Once a week
5. Less than once a week

IF (BR028_MeatWeek > a2) AND (MN032_socex = 1)
Txt_FL_BR033

BR033_MeatAfford
Would you say that you do not eat meat, fish or poultry more often because...

IWER:
{ReadOut}
MEAT AFFORD
1. you cannot afford to eat it more often
2. for other reasons

*ENDIF*Txt_FL_BR029

BR029_FruitsVegWeek
(Please look at card 17.) In a regular week, how often do you consume a serving of fruits or vegetables?
HOW OFTEN A WEEK DO YOU CONSUME A SERVING OF FRUITS OR VEGETABLES
1. Every day
2. 3-6 times a week
3. Twice a week
4. Once a week
5. Less than once a week

ENDIF

BR017_IntCheck

IWER:
CHECK:
Who answered the questions in this section?
INTERVIEWER CHECK BR
1. Respondent only
2. Respondent and proxy
3. Proxy only

System preset value BR902_TimeStampEnd
IF BR902_TimeStampEnd = EMPTY AND BR017_IntCheck <> EMPTY
ENDIF

ENDBLOCK

ENDIF
IF (CF IN Test) OR (ALL IN Test)

BLOCK

IF MN025_RandomCF102 = 1

Txt_FL_CF104

System preset value CF104_Learn1

IF CF104_Learn1 <> EMPTY

ELSE

IF ACTIVELANGUAGE = L1

ENDIF

ENDIF

ELSE

IF MN025_RandomCF102 = 2

Txt_FL_CF105

System preset value CF105_Learn1

IF CF105_Learn1 <> EMPTY

ELSE

IF ACTIVELANGUAGE = L1

ENDIF

ENDIF

ELSE

IF MN025_RandomCF102 = 3

Txt_FL_CF106

System preset value CF106_Learn1

IF CF106_Learn1 <> EMPTY

ELSE

IF ACTIVELANGUAGE = L1

ENDIF

ENDIF

ELSE

IF MN025_RandomCF102 = 4

Txt_FL_CF107

System preset value CF107_Learn1

IF CF107_Learn1 <> EMPTY

ELSE

IF ACTIVELANGUAGE = L1

ENDIF

ENDIF

ELSE

ENDIF

ENDIF

ENDIF

ENDIF

System preset value CF010_Animals
IF CF010_Animals <> EMPTY
ELSE

IF ACTIVELANGUAGE = L1
ENDIF

ENDIF

CF019_CFInstruct

IWER:
This is the cognitive test section: while you complete this section, make sure that no third persons are present. Start of a @Bnon-proxy@B section . No proxy allowed. If the respondent is not capable of answering any of these questions on her/his own, please select '5'.
INSTRUCTION FOR CF
1. Continue
5. Proxy-interview

System preset value CF901_TimeStampStart
IF CF901_TimeStampStart = EMPTY AND CF019_CFInstruct <> EMPTY
ENDIF
IF (CF019_CFInstruct = RESPONSE) AND NOT (CF019_CFInstruct = a5)

IF MN101_Longitudinal = 0
Txt_FL_CF001

CF001_SRRead
Now I would like to ask some questions about your reading and writing skills. How would you rate your reading skills needed in your daily life? Would you say they are...

IWER:
{ReadOut}
SELF-RATED READING SKILLS
1. Excellent
2. Very good
3. Good
4. Fair
5. Poor

Txt_FL_CF002

CF002_SRWrite
How would you rate your writing skills needed in your daily life? Would you say they are...

IWER:
{ReadOut}
SELF-RATED WRITING SKILLS
1. Excellent
2. Very good
3. Good
4. Fair
5. Poor

ENDIF

CF003_DateDay
Part of this study is concerned with people's memory AND ability to think about things. First, I am going to ask about today's date. Which day of the month is it?

IWER:

Code whether day of month ({FLDAY}) is given correctly
DATE-DAY OF MONTH
1. Day of month given correctly
2. Day of month given incorrectly/doesn't know day

CF004_DateMonth
Which month is it?

IWER:
Code whether month ([FLDefault[10]/FLDefault[11]/FLDefault[12]/FLDefault[13]/FLDefault[14]/FLDefault[15]/FLDefault[16]/FLDefault[17]/FLDefault[18]/FLDefault[19]/FLDefault[20]/FLDefault[21]]) is given correctly
DATE-MONTH
1. Month given correctly
2. Month given incorrectly/doesn't know month

CF005_DateYear
Which year is it?

IWER:
Code whether year ([STR (Year)]) is given correctly
DATE-YEAR
1. Year given correctly
2. Year given incorrectly/doesn't know year

Txt_FL_CF006

CF006_DayWeek
Can you tell me what day of the week it is?

IWER:
Correct answer: ({FLTODAY})
DAY OF THE WEEK
1. Day of week given correctly
2. Day of week given incorrectly/doesn't know day

IF MN101_Longitudinal = 0

Txt_FL_CF103

CF103_Memory
How would you rate your memory at the present time? Would you say it is excellent, very good, good, fair or poor?
SELF-RATED WRITING SKILLS
1. Excellent
2. Very good
3. Good
4. Fair
5. Poor

*ENDIF*Txt_FL_CF007

CF007_Learn1Intro
Now, I am going to read a list of words from my computer screen. We have purposely made the list long so it will be difficult for anyone to recall all the words. Most people recall just a few. Please listen carefully, as the set of words cannot be repeated. When I have finished, I will ask you to recall aloud as many of the words as you can, in any order. Is this clear?

IWER:
Have booklet ready
INTRODUCTION TEN WORDS LIST LEARNING
1. Continue

Txt_FL_CF009

System preset value CF009_VerbFluIntro
IF CF007_Learn1Intro = RESPONSE

IF CF009_VerbFluIntro = EMPTY
Txt_FL_CF101

CF101_Learn1
Ready?

IWER:
Wait until words appear on the screen. Write words on sheet provided. Allow up to one minute for recall.
Enter the words respondent correctly recalls.
TEN WORDS LIST LEARNING FIRST TRIAL
1. Start test

IF CF101_Learn1 <> REFUSAL

CF102_Learn1

TEN WORDS LIST LEARNING SHOW MOVIE
1. Continue

IF MN025_RandomCF102 = 1

CF104_Learn1
Now please tell me all the words you can recall.
TEN WORDS LIST LEARNING FIRST TRIAL
1. Hotel
2. River
3. Tree
4. Skin
5. Gold
6. Market
7. Paper
8. Child
9. King
10. Book
96. None of these

CHECK NOT ((CF104_Learn1.CARDINAL > 1) AND (96 IN CF104_Learn1)) L1 " [You cannot select ""None of the above"" together with any other answer. Please change your answer]"

IF 96 IN CF104_Learn1
ELSE
ENDIF

ELSE

IF MN025_RandomCF102 = 2

CF105_Learn1
Now please tell me all the words you can recall.
TEN WORDS LIST LEARNING FIRST TRIAL
1. Sky
2. Ocean
3. Flag
4. Dollar
5. Wife

6. Machine
7. Home
8. Earth
9. College
10. Butter
96. None of these

CHECK NOT ((CF105_Learn1.CARDINAL > 1) AND (96 IN CF105_Learn1)) L1 " [You cannot select ""None of the above"" together with any other answer. Please change your answer]"

IF 96 IN CF105_Learn1
ELSE
ENDIF

ELSE

IF MN025_RandomCF102 = 3

CF106_Learn1
Now please tell me all the words you can recall.
TEN WORDS LIST LEARNING FIRST TRIAL
1. Woman
2. Rock
3. Blood
4. Corner
5. Shoes
6. Letter
7. Girl
8. House
9. Valley
10. Engine
96. None of these

CHECK NOT ((CF106_Learn1.CARDINAL > 1) AND (96 IN CF106_Learn1)) L1 " [You cannot select ""None of the above"" together with any other answer. Please change your answer]"

IF 96 IN CF106_Learn1
ELSE
ENDIF

ELSE

IF MN025_RandomCF102 = 4

CF107_Learn1
Now please tell me all the words you can recall.
TEN WORDS LIST LEARNING FIRST TRIAL
1. Water
2. Church
3. Doctor
4. Palace
5. Fire
6. Garden
7. Sea
8. Village
9. Baby
10. Table
96. None of these

CHECK NOT ((CF107_Learn1.CARDINAL > 1) AND (96 IN CF107_Learn1)) L1 " [You cannot select ""None of the above"" together with any other answer. Please change your answer]"

IF 96 IN CF107_Learn1
ELSE

ENDIF

ENDIF

ENDIF

ENDIF

ENDIF

ENDIF

ELSE

System preset value CF104_Learn1
System preset value CF105_Learn1
System preset value CF106_Learn1
System preset value CF107_Learn1
System preset value CF904_Learn1Tot
System preset value CF905_Learn1Tot
System preset value CF906_Learn1Tot
System preset value CF907_Learn1Tot

ENDIF

ENDIF

CF009_VerbFluIntro
Now I would like you to name as many different animals as you can think of. You have one minute to do this.

Ready, go.

IWER:
Allow one minute precisely. If the respondent stops before the end of the time, encourage him/her to try to find more words. If he/she is silent for 15 seconds repeat the basic instruction ('I want you to tell me all the animals you can think of'). No extension on the time limit is made in the event that the instruction has to be repeated.
VERBAL FLUENCY INTRO
1. Continue

System preset value CF011_IntroNum
IF CF009_VerbFluIntro = RESPONSE

IF CF011_IntroNum = EMPTY

CF010_Animals

IWER:
The score is the sum of acceptable animals. Any member of the animal kingdom, real or mythical is scored correct, except repetitions and proper nouns. Specifically each of the following gets credit: a species name and any accompanying breeds within the species; male, female and infant names within the species. Code number of animals (0..100)
VERBAL FLUENCY SCORE
0..100

ELSE

System preset value CF010_Animals

ENDIF

ENDIF
IF MN101_Longitudinal = 0

Txt_FL_CF011

CF011_IntroNum
Next I would like to ask you some questions which assess how people use numbers in everyday life.

IWER:
If necessary, encourage the respondent to try to answer each of the numeracy questions
INTRODUCTION NUMERACY
1. Continue

CF012_NumDis
If the chance of getting a disease is 10 percent, how many people out of 1000 (one thousand) would be expected to get the disease?

IWER:
Do not read out the answers
NUMERACY-CHANCE DISEASE 10 PERC. OF 1000
1. 100
2. 10
3. 90
4. 900
97. Other answer

IF CF012_NumDis <> a1

CF013_NumHalfPrice
In a sale, a shop is selling all items at half price. Before the sale, a sofa costs 300 [FLDefault[9]]. How much will it cost in the sale?

IWER:
Do not read out the answers
NUMERACY-HALF PRICE
1. 150 ^FLCurr
2. 600 ^FLCurr
97. Other answer

ENDIF
IF CF012_NumDis = a1

CF014_NumCar
A second hand car dealer is selling a car for 6,000 [FLDefault[9]]. This is two-thirds of what it costs new. How much did the car cost new?

IWER:
Do not read out the answers
Paper and pencil should not be used by the respondent.
NUMERACY-6000 IS TWO-THIRDS WHAT IS TOTAL PRICE
1. 9,000 ^FLCurr
2. 4,000 ^FLCurr
3. 8,000 ^FLCurr
4. 12,000 ^FLCurr
5. 18,000 ^FLCurr
97. Other answer

IF CF014_NumCar = a1
Txt_FL_CF015

CF015_Savings
Let's say you have 2000 [FLDefault[9]] in a savings account. The account earns ten per cent interest each year. How much would you have in the account at the end of two years?

IWER:
Do not read out the answers
AMOUNT IN THE SAVINGS ACCOUNT
1. 2420 ^FLCurr
2. 2020 ^FLCurr
3. 2040 ^FLCurr
4. 2100 ^FLCurr
5. 2200 ^FLCurr
6. 2400 ^FLCurr
97. Other answer

ENDIF

ENDIF

ENDIF

CF108_Serial
Now let's try some subtraction of numbers. One hundred minus 7 equals what?

IWER:
Paper and pencil should not be used by the respondent.
If R adds 7 instead, you may repeat question.
NUMERACY-SUBTRACTION 1
-1000000000000000000..10000000000000000000

IF (CF108_Serial < 99999998) AND NOT ((CF108_Serial = REFUSAL) OR (CF108_Serial = DONTKNOW))

CF109_Serial
And 7 from that

IWER:
This is the second subtraction
NUMERACY-SUBTRACTION 2
-1000000000000000000..10000000000000000000

IF (CF109_Serial < 99999998) AND NOT ((CF109_Serial = REFUSAL) OR (CF109_Serial = DONTKNOW))

CF110_Serial
And 7 from that

IWER:
This is the third subtraction
NUMERACY-SUBTRACTION 3
-1000000000000000000..10000000000000000000

IF (CF110_Serial < 99999998) AND NOT ((CF110_Serial = REFUSAL) OR (CF110_Serial = DONTKNOW))

CF111_Serial
And 7 from that

IWER:
This is the fourth subtraction
NUMERACY-SUBTRACTION 4
-1000000000000000000..10000000000000000000

IF (CF111_Serial < 99999998) AND NOT ((CF111_Serial = REFUSAL) OR (CF111_Serial = DONTKNOW))

CF112_Serial
And 7 from that

IWER:

This is the fifth subtraction
NUMERACY-SUBTRACTION 5
-1000000000000000000..1000000000000000000

ENDIF

ENDIF

ENDIF

ENDIF
IF [CF007_Learn1Intro](#) = RESPONSE

IF MN025_RandomCF102 = 1
Txt_FL_CF113

CF113_Learn4
A little while ago, I read you a list of words and you repeated the ones you could remember. Please tell me any of the words that you can remember now?

IWER:
Write words on sheet provided. Allow up to one minute for recall. Enter the words respondent correctly recalls.
TEN WORDS LIST LEARNING DELAYED RECALL
1. Hotel
2. River
3. Tree
4. Skin
5. Gold
6. Market
7. Paper
8. Child
9. King
10. Book
96. None of these

CHECK NOT ((CF113_Learn4.CARDINAL > 1) AND (96 IN CF113_Learn4)) L1 " [You cannot select ""None of the above"" together with any other answer. Please change your answer]"

IF 96 IN [CF113_Learn4](#)
ELSE
ENDIF

ELSE

IF MN025_RandomCF102 = 2
Txt_FL_CF114

CF114_Learn4
A little while ago, I read you a list of words and you repeated the ones you could remember. Please tell me any of the words that you can remember now?

IWER:
Write words on sheet provided. Allow up to one minute for recall. Enter the words respondent correctly recalls.
TEN WORDS LIST LEARNING DELAYED RECALL
1. Sky
2. Ocean
3. Flag
4. Dollar
5. Wife
6. Machine
7. Home
8. Earth

9. College
10. Butter
96. None of these

CHECK NOT ((CF114_Learn4.CARDINAL > 1) AND (96 IN CF114_Learn4)) L1 " [You cannot select ""None of the above"" together with any other answer. Please change your answer]"

IF 96 IN CF114_Learn4
ELSE
ENDIF

ELSE

IF MN025_RandomCF102 = 3

Txt_FL_CF115

CF115_Learn4

A little while ago, I read you a list of words and you repeated the ones you could remember. Please tell me any of the words that you can remember now?

IWER:
Write words on sheet provided. Allow up to one minute for recall. Enter the words respondent correctly recalls.
TEN WORDS LIST LEARNING DELAYED RECALL

1. Woman
2. Rock
3. Blood
4. Corner
5. Shoes
6. Letter
7. Girl
8. House
9. Valley
10. Engine
96. None of these

CHECK NOT ((CF115_Learn4.CARDINAL > 1) AND (96 IN CF115_Learn4)) L1 " [You cannot select ""None of the above"" together with any other answer. Please change your answer]"

IF 96 IN CF115_Learn4
ELSE
ENDIF

ELSE

Txt_FL_CF116

CF116_Learn4

A little while ago, I read you a list of words and you repeated the ones you could remember. Please tell me any of the words that you can remember now?

IWER:
Write words on sheet provided. Allow up to one minute for recall. Enter the words respondent correctly recalls.
TEN WORDS LIST LEARNING DELAYED RECALL

1. Water
2. Church
3. Doctor
4. Palace
5. Fire
6. Garden
7. Sea
8. Village
9. Baby
10. Table

96. None of these

CHECK NOT ((CF116_Learn4.CARDINAL > 1) AND (96 IN CF116_Learn4)) L1 " [You cannot select ""None of the above"" together with any other answer. Please change your answer]"

IF 96 IN CF116_Learn4
ELSE
ENDIF

ENDIF

ENDIF

ENDIF

ENDIF

CF017_Factors

IWER:
Were there any factors that may have impaired the respondent's performance on the tests?
If you want to comment, use CTRL+M
CONTEXTUAL FACTORS DURING THE COGNITIVE FUNCTION TEST
1. Yes
5. No

CF018_IntCheck
INTERVIEWER CHECK: WHO WAS PRESENT DURING THIS SECTION?

IWER:
{CodeAll}
WHO WAS PRESENT DURING CF
1. Respondent alone
2. Partner present
3. Child(ren) present
4. Other(s)

CHECK NOT ((CF018_IntCheck.CARDINAL > 1) AND (a1 IN CF018_IntCheck)) L1 " [Cannot select -respondent alone- with any other category]"
CHECK NOT ((Sec_CH.NumberOFReportedChildren = 0) AND (a3 IN CF018_IntCheck)) L1 " [You answered earlier you had no children]"

System preset value CF902_TimeStampEnd
IF CF902_TimeStampEnd = EMPTY AND CF018_IntCheck <> EMPTY
ENDIF

ENDIF

ENDBLOCK

ENDIF
IF (MH IN Test) OR (ALL IN Test)

BLOCK
Txt_FL_MH001

MH001_Intro
Earlier we talked about your physical health. Another measure of health is your emotional health or well being -- that is, how you feel about things that happen around you.

IWER:
Start of a @BNon-proxy section@B. No proxy allowed. If the respondent is not present or not capable to give

consent to participation on her/his own, please select '5'.
INTRO MENTAL HEALTH
1. Continue
5. Proxy-interview

System preset value MH901_TimeStampStart
IF MH901_TimeStampStart = EMPTY AND MH001_Intro <> EMPTY
ENDIF
IF (MH001_Intro = RESPONSE) AND NOT (MH001_Intro = a5)
Txt_FL_MH002

MH002_Depression
In the last month, have you been sad or depressed?

IWER:
If participant asks for clarification, say 'by sad or depressed, we mean miserable, in low spirits, or blue'
DEPRESSION
1. Yes
5. No

Txt_FL_MH003

MH003_Hopes
What are your hopes for the future?

IWER:
Note only whether hopes are mentioned or not
HOPES FOR THE FUTURE
1. Any hopes mentioned
2. No hopes mentioned

Txt_FL_MH004

MH004_WishDeath
In the last month, have you felt that you would rather be dead?
FELT WOULD RATHER BE DEAD
1. Any mention of suicidal feelings or wishing to be dead
2. No such feelings

Txt_FL_MH005

MH005_Guilt
Do you tend to blame yourself or feel guilty about anything?
FEELS GUILTY
1. Obvious excessive guilt or self-blame
2. No such feelings
3. Mentions guilt or self-blame, but it is unclear if these constitute obvious or excessive guilt or self-blame

IF MH005_Guilt = a3
Txt_FL_MH006

MH006_BlameForWhat
So, for what do you blame yourself?

IWER:
Note - Only code 1 for an exaggerated feeling of guilt, which is clearly out of proportion to the circumstances. The fault will often have been very minor, if there was one at all. Justifiable or appropriate guilt should be coded 2.
BLAME FOR WHAT
1. Example(s) given constitute obvious excessive guilt or self-blame
2. Example(s) do not constitute obvious excessive guilt or self-blame, or it remains unclear if these constitute obvious or excessive guilt or self-blame

*ENDIF*Txt_FL_MH007

MH007_Sleep
Have you had trouble sleeping recently?
TROUBLE SLEEPING
1. Trouble with sleep or recent change in pattern
2. No trouble sleeping

Txt_FL_MH008

MH008_Interest
In the last month, what is your interest in things?
LESS OR SAME INTEREST IN THINGS
1. Less interest than usual mentioned
2. No mention of loss of interest
3. Non-specific or uncodeable response

IF MH008_Interest = a3
Txt_FL_MH009

MH009_KeepUpInt
So, do you keep up your interests?
KEEPS UP INTEREST
1. Yes
5. No

*ENDIF*Txt_FL_MH010

MH010_Irritability
Have you been irritable recently?
IRRITABILITY
1. Yes
5. No

Txt_FL_MH011

MH011_Appetite
What has your appetite been like in the last month?
APPETITE
1. Diminution in desire for food
2. No diminution in desire for food
3. Non-specific or uncodeable response

IF MH011_Appetite = a3
Txt_FL_MH012

MH012_EatMoreLess
So, have you been eating more or less than usual?
EATING MORE OR LESS
1. Less
2. More
3. Neither more nor less

*ENDIF*Txt_FL_MH013

MH013_Fatigue
In the last month, have you had too little energy to do the things you wanted to do?
FATIGUE

1. Yes
5. No

Txt_FL_MH014

MH014_ConcEnter
How is your concentration? For example, can you concentrate on a television programme, film or radio programme?
CONCENTRATION ON ENTERTAINMENT
1. Difficulty in concentrating on entertainment
2. No such difficulty mentioned

Txt_FL_MH015

MH015_ConcRead
Can you concentrate on something you read?
CONCENTRATION ON READING
1. Difficulty in concentrating on reading
2. No such difficulty mentioned

Txt_FL_MH016

MH016_Enjoyment
What have you enjoyed doing recently?
ENJOYMENT
1. Fails to mention any enjoyable activity
2. Mentions ANY enjoyment from activity

Txt_FL_MH017

MH017_Tear
In the last month, have you cried at all?
TEARFULNESS
1. Yes
5. No

Txt_FL_MH033

MH033_Intro
I will now read some statements and would like to ask you to answer how much of the time you feel certain ways: often, some of the time, hardly ever or never.
INTRODUCTION HOW MUCH YOU FEEL
1. Continue

Txt_FL_MH034

MH034_companionship
How much of the time do you feel you lack companionship?

IWER:
{ReadOut}
HOW OFTEN LACK COMPANIONSHIP
1. Often
2. Some of the time
3. Hardly ever or never

Txt_FL_MH035

MH035_LeftOut
How much of the time do you feel left out?

IWER:
Repeat if necessary
HOW OFTEN LEFT OUT

1. Often
2. Some of the time
3. Hardly ever or never

Txt_FL_MH036

MH036_Isolated
How much of the time do you feel isolated from others?

IWER:
Repeat if necessary
HOW OFTEN ISOLATED
1. Often
2. Some of the time
3. Hardly ever or never

Txt_FL_MH037

MH037_lonely
How much of the time do you feel lonely?

IWER:
Repeat if necessary
HOW OFTEN LONELY
1. Often
2. Some of the time
3. Hardly ever or never

IF MH902_TimeStampEnd = EMPTY
ENDIF

ENDIF

ENDBLOCK

ENDIF
IF (HC IN Test) OR (ALL IN Test)

BLOCK
Txt_FL_HC601

HC601_Intro
Now we have some questions about your doctor visits, hospital stays, or the medication you took in the last 12 months, that is since {FLLastYearMonth}. It is also important to us to learn about how much you paid yourself for your health care without getting reimbursed by your health insurance/national health system/third party payer.
CONTACTS WITH GENERAL PRACTITIONER
1. Continue

System preset value HC901_TimeStampStart
IF HC901_TimeStampStart = EMPTY AND HC601_Intro <> EMPTY
*ENDIF*Txt_FL_HC125

HC125_Satisfaction_with_Insurance
Let us begin with your health insurance. Overall, how satisfied are you with your own coverage in your basic health insurance/national health system? Are you

IWER:
{ReadOut}
SATISFACTION WITH INSURANCE
1. very satisfied
2. somewhat satisfied

3. somewhat dissatisfied
4. very dissatisfied

Txt_FL_HC113

HC113_SuppHealthInsurance
Do you have any supplementary health insurance that pays for services not covered by your basic health insurance/national health system/ third party payer. These services may include in-patient services, health examinations, office visits, dental care, other treatments or drugs.
ANY SUPPLEMENTARY HEALTH INSURANCE
1. Yes
5. No

Txt_FL_HC116

HC116_LongTermCareInsurance
Do you have any of the following public or private long-term care insurances?

IWER:
{ReadOut}
{CodeAll}
If unclear, explain: Long-term care insurance helps cover the cost of long-term care. It generally covers home care, assisted living, adult daycare, respite care, hospice care, and stays in nursing homes or residential care facilities. Some of the long term care services might be covered by your health insurance.
HAS LONGTERM CARE INSURANCE
1. Public
2. Private mandatory
3. Private voluntary/supplementary
96. None

CHECK NOT ((HC116_LongTermCareInsurance.CARDINAL > 1) AND (a96 IN HC116_LongTermCareInsurance))
L1 " [You cannot select ""None of the above"" together with any other answer. Please change your answer]"
Txt_FL_HC602

HC602_STtoMDoctor
Now please think about the last 12 months. Since {FLLastYearMonth} about how many times in total have you seen or talked to a medical doctor or qualified/registered nurse about your health? Please exclude dentist visits and hospital stays, but include emergency room or outpatient clinic visits.

IWER:
Please also count contacts by telephone or other means.
SEEN OR TALKED TO MEDICAL DOCTOR
0..98

IF HC602_STtoMDoctor > 0
Txt_FL_HC682

HC682_OOPDocsYesNo
Did you pay anything yourself for your doctor visits (in the last twelve months)? Please also include expenses for diagnostic exams, such as imaging or laboratory diagnostics.
PAYED ANYTHING OUT OF POCKET
1. Yes
5. No

IF HC682_OOPDocsYesNo = a1
Txt_FL_HC683

HC683_OOPDocsAmount
Overall, how much did you pay yourself for your doctor visits (in the last twelve months), that is how much did you pay without getting reimbursed by [a health insurance/your national health system/a third party payer]?

IWER:

Dentist visits excluded. Enter an amount in [FLDefault[9]].
HOW MUCH PAYED OUT OF POCKET
-1000000000000000000..10000000000000000000
CHECK NOT ((HC683_OOPDocsAmount <= 0) AND (HC683_OOPDocsAmount = RESPONSE)) L1 " [Amount is expected to be higher than zero]"

IF HC683_OOPDocsAmount = NONRESPONSE

BLOCK

IF (((((MN001_Country = a1) OR (MN001_Country = a2)) OR (MN001_Country = a3)) OR (MN001_Country = a5)) OR (MN001_Country = a12)) OR (MN001_Country = a17)

IF piIndex = 1
ELSE

IF piIndex = 2
ELSE

IF piIndex = 3
ELSE

IF piIndex = 4
ELSE

IF piIndex = 5
ELSE

IF piIndex = 6
ELSE

IF piIndex = 7
ELSE

IF piIndex = 8
ELSE

IF piIndex = 9
ENDIF

ENDIF

ENDIF

ENDIF

ENDIF

ENDIF

ENDIF

ENDIF

ENDIF

ELSE

IF ((MN001_Country = a6) OR (MN001_Country = a7)) OR (MN001_Country = a24)

IF piIndex = 1
ELSE

IF piIndex = 2

ELSE

IF piIndex = 3
ELSE

IF piIndex = 4
ELSE

IF piIndex = 5
ELSE

IF piIndex = 6
ELSE

IF piIndex = 7
ELSE

IF piIndex = 8
ELSE

IF piIndex = 9
ENDIF

ENDIF

ENDIF

ENDIF

ENDIF

ENDIF

ENDIF

ENDIF

ENDIF

ELSE

IF MN001_Country = a8

IF piIndex = 1
ELSE

IF piIndex = 2
ELSE

IF piIndex = 3
ELSE

IF piIndex = 4
ELSE

IF piIndex = 5
ELSE

IF piIndex = 6
ELSE

IF piIndex = 7
ELSE

IF piIndex = 8
ELSE
IF piIndex = 9
ENDIF
ENDIF
ENDIF
ENDIF
ENDIF
ENDIF
ENDIF
ENDIF
ENDIF
ELSE
IF ((MN001_Country = a13) OR (MN001_Country = a18)) OR (MN001_Country = a19)
IF piIndex = 1
ELSE
IF piIndex = 2
ELSE
IF piIndex = 3
ELSE
IF piIndex = 4
ELSE
IF piIndex = 5
ELSE
IF piIndex = 6
ELSE
IF piIndex = 7
ELSE
IF piIndex = 8
ELSE
IF piIndex = 9
ENDIF
ENDIF
ENDIF
ENDIF
ENDIF
ENDIF
ENDIF

ENDIF

ENDIF

ELSE

IF MN001_Country = a22

IF piIndex = 1
ELSE

IF piIndex = 2
ELSE

IF piIndex = 3
ELSE

IF piIndex = 4
ELSE

IF piIndex = 5
ELSE

IF piIndex = 6
ELSE

IF piIndex = 7
ELSE

IF piIndex = 8
ELSE

IF piIndex = 9
ENDIF

ENDIF

ENDIF

ENDIF

ENDIF

ENDIF

ENDIF

ENDIF

ENDIF

ELSE

IF MN001_Country = a21

IF piIndex = 1
ELSE

IF piIndex = 2
ELSE

IF piIndex = 3
ELSE

IF piIndex = 4
ELSE
IF piIndex = 5
ELSE
IF piIndex = 6
ELSE
IF piIndex = 7
ELSE
IF piIndex = 8
ELSE
IF piIndex = 9
ENDIF
ENDIF
ENDIF
ENDIF
ENDIF
ENDIF
ENDIF
ENDIF
ENDIF
ELSE
IF MN001_Country = a4
IF piIndex = 1
ELSE
IF piIndex = 2
ELSE
IF piIndex = 3
ELSE
IF piIndex = 4
ELSE
IF piIndex = 5
ELSE
IF piIndex = 6
ELSE
IF piIndex = 7
ELSE
IF piIndex = 8
ELSE
IF piIndex = 9

ENDIF

ENDIF

ENDIF

ENDIF

ENDIF

ENDIF

ENDIF

ENDIF

ENDIF

ELSE

IF MN001_Country = a10

IF piIndex = 1
ELSE

IF piIndex = 2
ELSE

IF piIndex = 3
ELSE

IF piIndex = 4
ELSE

IF piIndex = 5
ELSE

IF piIndex = 6
ELSE

IF piIndex = 7
ELSE

IF piIndex = 8
ELSE

IF piIndex = 9
ENDIF

ENDIF

ENDIF

ENDIF

ENDIF

ENDIF

ENDIF

ENDIF

ENDIF

ELSE
IF MN001_Country = a11
IF piIndex = 1
ELSE
IF piIndex = 2
ELSE
IF piIndex = 3
ELSE
IF piIndex = 4
ELSE
IF piIndex = 5
ELSE
IF piIndex = 6
ELSE
IF piIndex = 7
ELSE
IF piIndex = 8
ELSE
IF piIndex = 9
ENDIF
ENDIF
ENDIF
ENDIF
ENDIF
ENDIF
ENDIF
ENDIF
ENDIF
ELSE
IF MN001_Country = a11
IF piIndex = 1
ELSE
IF piIndex = 2
ELSE
IF piIndex = 3
ELSE
IF piIndex = 4
ELSE

IF piIndex = 5
ELSE

IF piIndex = 6
ELSE

IF piIndex = 7
ELSE

IF piIndex = 8
ELSE

IF piIndex = 9
ENDIF

ENDIF

ENDIF

ENDIF

ENDIF

ENDIF

ENDIF

ENDIF

ENDIF

ELSE

IF MN001_Country = a24

IF piIndex = 1
ELSE

IF piIndex = 2
ELSE

IF piIndex = 3
ELSE

IF piIndex = 4
ELSE

IF piIndex = 5
ELSE

IF piIndex = 6
ELSE

IF piIndex = 7
ELSE

IF piIndex = 8
ELSE

IF piIndex = 9
ENDIF

ENDIF

ENDIF

ENDIF

ENDIF

ENDIF

ENDIF

ENDIF

ENDIF

ELSE

IF MN001_Country = a16

IF piIndex = 1
ELSE

IF piIndex = 2
ELSE

IF piIndex = 3
ELSE

IF piIndex = 4
ELSE

IF piIndex = 5
ELSE

IF piIndex = 6
ELSE

IF piIndex = 7
ELSE

IF piIndex = 8
ELSE

IF piIndex = 9
ENDIF

ENDIF

ENDIF

ENDIF

ENDIF

ENDIF

ENDIF

ENDIF

ENDIF

ELSE

IF MN001_Country = a16

IF piIndex = 1
ELSE
IF piIndex = 2
ELSE
IF piIndex = 3
ELSE
IF piIndex = 4
ELSE
IF piIndex = 5
ELSE
IF piIndex = 6
ELSE
IF piIndex = 7
ELSE
IF piIndex = 8
ELSE
IF piIndex = 9
ENDIF
ENDIF
ENDIF
ENDIF
ENDIF
ENDIF
ENDIF
ENDIF
ENDIF
ELSE
IF MN001_Country = a14
IF piIndex = 1
ELSE
IF piIndex = 2
ELSE
IF piIndex = 3
ELSE
IF piIndex = 4
ELSE
IF piIndex = 5
ELSE
IF piIndex = 6

ELSE

IF piIndex = 7
ELSE

IF piIndex = 8
ELSE

IF piIndex = 9
ENDIF

ENDIF

ENDIF

ENDIF

ENDIF

ENDIF

ENDIF

ENDIF

ENDIF

ELSE

IF MN001_Country = a15

IF piIndex = 1
ELSE

IF piIndex = 2
ELSE

IF piIndex = 3
ELSE

IF piIndex = 4
ELSE

IF piIndex = 5
ELSE

IF piIndex = 6
ELSE

IF piIndex = 7
ELSE

IF piIndex = 8
ELSE

IF piIndex = 9
ENDIF

ENDIF

ENDIF

ENDIF

ENDIF

ENDIF

ENDIF

ENDIF

ENDIF

ELSE

IF MN001_Country = a20

IF piIndex = 1
ELSE

IF piIndex = 2
ELSE

IF piIndex = 3
ELSE

IF piIndex = 4
ELSE

IF piIndex = 5
ELSE

IF piIndex = 6
ELSE

IF piIndex = 7
ELSE

IF piIndex = 8
ELSE

IF piIndex = 9
ENDIF

ENDIF

ENDIF

ENDIF

ENDIF

ENDIF

ENDIF

ENDIF

ENDIF

ENDIF

ENDIF

ENDIF

ENDIF

ENDIF

ENDIF

ENDIF

ENDIF

ENDIF

ENDIF

ENDIF

ENDIF

ENDIF

ENDIF

ENDIF

ENDIF

A
[Was is less or more than] {Amount} [FLDefault[9]]?
HOW MUCH
1. Less
2. About ^Amount
3. More

ENDBLOCK

ENDIF

ENDIF

*ENDIF*Txt_FL_HC114

HC114_UnmetNeedCost
Was there a time in the past 12 months when you needed to see a doctor but could not because of cost?

IWER:
Any type of doctor or qualified nurse, emergency room or outpatient clinic visits included.
DID NOT GO TO DOCTOR BECAUSE OF COST
1. Yes
5. No

Txt_FL_HC115

HC115_UnmetNeedWait
Was there a time in the past 12 months when you needed to see a doctor but could not because you had to wait too long?

IWER:
Any type of doctor or qualified nurse, emergency room or outpatient clinic visits included.
DID NOT GO TO DOCTOR BECAUSE OF LONG WAIT
1. Yes
5. No

Txt_FL_HC688

HC688_OOPDRUGSYesNo
Earlier we talked about medication you may take. In the last twelve months, that is since {FLLastYearMonth}, did you pay anything yourself for your medication? Please include both drugs that were prescribed by your doctor and those you bought without prescription.
PAYED ANYTHING OUT OF POCKET DRUGS
1. Yes
5. No

IF HC688_OOPDRUGSYesNo = a1
Txt_FL_HC689

HC689_OOPDRUGSAmount
Overall, about how much did you pay yourself for your medication in the last twelve months (that is how much did you pay without getting reimbursed by [a health insurance/your national health system/ a third party payer])?

IWER:
Include both drugs prescribed by your doctor and drugs bought over the counter.
Enter an amount in [FLDefault[9]]
HOW MUCH PAYED OUT OF POCKET DRUGS
-1000000000000000000..10000000000000000000
CHECK NOT ((HC689_OOPDRUGSAmount <= 0) AND (HC689_OOPDRUGSAmount = RESPONSE)) L1 "[Amount is expected to be higher than zero]"

IF HC689_OOPDRUGSAmount = DONTKNOW
Txt_FL_HC630

HC630_OOPDRUGSAmountMonth
Can you tell me about how much you pay yourself for your medication in a typical month?

IWER:
Include both drugs prescribed by your doctor and drugs bought without prescription
HOW MUCH PAYED OUT OF POCKET DRUGS
-1000000000000000000..10000000000000000000

IF HC630_OOPDRUGSAmountMonth = NONRESPONSE

BLOCK

IF (((((MN001_Country = a1) OR (MN001_Country = a2)) OR (MN001_Country = a3)) OR (MN001_Country = a5)) OR (MN001_Country = a12)) OR (MN001_Country = a17)

IF piIndex = 1
ELSE

IF piIndex = 2
ELSE

IF piIndex = 3
ELSE

IF piIndex = 4
ELSE

IF piIndex = 5
ELSE

IF piIndex = 6
ELSE

IF piIndex = 7
ELSE

IF piIndex = 8
ELSE

IF piIndex = 9
ENDIF

ENDIF

ENDIF

ENDIF

ENDIF

ENDIF

ENDIF

ENDIF

ENDIF

ELSE

IF ((MN001_Country = a6) OR (MN001_Country = a7)) OR (MN001_Country = a24)

IF piIndex = 1
ELSE

IF piIndex = 2
ELSE

IF piIndex = 3
ELSE

IF piIndex = 4
ELSE

IF piIndex = 5
ELSE

IF piIndex = 6
ELSE

IF piIndex = 7
ELSE

IF piIndex = 8
ELSE

IF piIndex = 9
ENDIF

ENDIF

ENDIF

ENDIF

ENDIF

ENDIF

ENDIF

ENDIF

ENDIF

ELSE

IF MN001_Country = a8

IF piIndex = 1
ELSE

IF piIndex = 2
ELSE

IF piIndex = 3
ELSE

IF piIndex = 4
ELSE

IF piIndex = 5
ELSE

IF piIndex = 6
ELSE

IF piIndex = 7
ELSE

IF piIndex = 8
ELSE

IF piIndex = 9
ENDIF

ENDIF

ENDIF

ENDIF

ENDIF

ENDIF

ENDIF

ENDIF

ENDIF

ELSE

IF ((MN001_Country = a13) OR (MN001_Country = a18)) OR (MN001_Country = a19)

IF piIndex = 1
ELSE

IF piIndex = 2
ELSE

IF piIndex = 3
ELSE

IF piIndex = 4
ELSE
IF piIndex = 5
ELSE
IF piIndex = 6
ELSE
IF piIndex = 7
ELSE
IF piIndex = 8
ELSE
IF piIndex = 9
ENDIF
ENDIF
ENDIF
ENDIF
ENDIF
ENDIF
ENDIF
ENDIF
ENDIF
ELSE
IF MN001_Country = a22
IF piIndex = 1
ELSE
IF piIndex = 2
ELSE
IF piIndex = 3
ELSE
IF piIndex = 4
ELSE
IF piIndex = 5
ELSE
IF piIndex = 6
ELSE
IF piIndex = 7
ELSE
IF piIndex = 8
ELSE
IF piIndex = 9

ENDIF

ENDIF

ENDIF

ENDIF

ENDIF

ENDIF

ENDIF

ENDIF

ENDIF

ELSE

IF MN001_Country = a21

IF piIndex = 1
ELSE

IF piIndex = 2
ELSE

IF piIndex = 3
ELSE

IF piIndex = 4
ELSE

IF piIndex = 5
ELSE

IF piIndex = 6
ELSE

IF piIndex = 7
ELSE

IF piIndex = 8
ELSE

IF piIndex = 9
ENDIF

ENDIF

ENDIF

ENDIF

ENDIF

ENDIF

ENDIF

ENDIF

ENDIF

ELSE
IF MN001_Country = a4
IF piIndex = 1
ELSE
IF piIndex = 2
ELSE
IF piIndex = 3
ELSE
IF piIndex = 4
ELSE
IF piIndex = 5
ELSE
IF piIndex = 6
ELSE
IF piIndex = 7
ELSE
IF piIndex = 8
ELSE
IF piIndex = 9
ENDIF
ENDIF
ENDIF
ENDIF
ENDIF
ENDIF
ENDIF
ENDIF
ENDIF
ELSE
IF MN001_Country = a10
IF piIndex = 1
ELSE
IF piIndex = 2
ELSE
IF piIndex = 3
ELSE
IF piIndex = 4
ELSE

IF piIndex = 5
ELSE

IF piIndex = 6
ELSE

IF piIndex = 7
ELSE

IF piIndex = 8
ELSE

IF piIndex = 9
ENDIF

ENDIF

ENDIF

ENDIF

ENDIF

ENDIF

ENDIF

ENDIF

ENDIF

ELSE

IF MN001_Country = a11

IF piIndex = 1
ELSE

IF piIndex = 2
ELSE

IF piIndex = 3
ELSE

IF piIndex = 4
ELSE

IF piIndex = 5
ELSE

IF piIndex = 6
ELSE

IF piIndex = 7
ELSE

IF piIndex = 8
ELSE

IF piIndex = 9
ENDIF

ENDIF

ENDIF

ENDIF

ENDIF

ENDIF

ENDIF

ENDIF

ENDIF

ELSE

IF MN001_Country = a11

IF piIndex = 1
ELSE

IF piIndex = 2
ELSE

IF piIndex = 3
ELSE

IF piIndex = 4
ELSE

IF piIndex = 5
ELSE

IF piIndex = 6
ELSE

IF piIndex = 7
ELSE

IF piIndex = 8
ELSE

IF piIndex = 9
ENDIF

ENDIF

ENDIF

ENDIF

ENDIF

ENDIF

ENDIF

ENDIF

ENDIF

ELSE

IF MN001_Country = a24

IF piIndex = 1
ELSE
IF piIndex = 2
ELSE
IF piIndex = 3
ELSE
IF piIndex = 4
ELSE
IF piIndex = 5
ELSE
IF piIndex = 6
ELSE
IF piIndex = 7
ELSE
IF piIndex = 8
ELSE
IF piIndex = 9
ENDIF
ENDIF
ENDIF
ENDIF
ENDIF
ENDIF
ENDIF
ENDIF
ENDIF
ELSE
IF MN001_Country = a16
IF piIndex = 1
ELSE
IF piIndex = 2
ELSE
IF piIndex = 3
ELSE
IF piIndex = 4
ELSE
IF piIndex = 5
ELSE
IF piIndex = 6

ELSE

IF piIndex = 7
ELSE

IF piIndex = 8
ELSE

IF piIndex = 9
ENDIF

ENDIF

ENDIF

ENDIF

ENDIF

ENDIF

ENDIF

ENDIF

ENDIF

ELSE

IF MN001_Country = a16

IF piIndex = 1
ELSE

IF piIndex = 2
ELSE

IF piIndex = 3
ELSE

IF piIndex = 4
ELSE

IF piIndex = 5
ELSE

IF piIndex = 6
ELSE

IF piIndex = 7
ELSE

IF piIndex = 8
ELSE

IF piIndex = 9
ENDIF

ENDIF

ENDIF

ENDIF

ENDIF

ENDIF

ENDIF

ENDIF

ENDIF

ELSE

IF MN001_Country = a14

IF piIndex = 1
ELSE

IF piIndex = 2
ELSE

IF piIndex = 3
ELSE

IF piIndex = 4
ELSE

IF piIndex = 5
ELSE

IF piIndex = 6
ELSE

IF piIndex = 7
ELSE

IF piIndex = 8
ELSE

IF piIndex = 9
ENDIF

ENDIF

ENDIF

ENDIF

ENDIF

ENDIF

ENDIF

ENDIF

ENDIF

ELSE

IF MN001_Country = a15

IF piIndex = 1
ELSE

IF piIndex = 2
ELSE
IF piIndex = 3
ELSE
IF piIndex = 4
ELSE
IF piIndex = 5
ELSE
IF piIndex = 6
ELSE
IF piIndex = 7
ELSE
IF piIndex = 8
ELSE
IF piIndex = 9
ENDIF
ENDIF
ENDIF
ENDIF
ENDIF
ENDIF
ENDIF
ENDIF
ENDIF
ELSE
IF MN001_Country = a20
IF piIndex = 1
ELSE
IF piIndex = 2
ELSE
IF piIndex = 3
ELSE
IF piIndex = 4
ELSE
IF piIndex = 5
ELSE
IF piIndex = 6
ELSE
IF piIndex = 7
ELSE

IF piIndex = 8
ELSE

IF piIndex = 9
ENDIF

ENDIF

ENDIF

ENDIF

ENDIF

ENDIF

ENDIF

ENDIF

ENDIF

ENDIF

ENDIF

ENDIF

ENDIF

ENDIF

ENDIF

ENDIF

ENDIF

ENDIF

ENDIF

ENDIF

ENDIF

ENDIF

ENDIF

ENDIF

ENDIF

A
[Was is less or more than] {Amount} [FLDefault[9]]?
HOW MUCH
1. Less
2. About ^Amount
3. More

ENDBLOCK

ENDIF

ENDIF

*ENDIF*Txt_FL_HC010

HC010_SNaDentist
During the last twelve months, that is since {FLLastYearMonth}, have you seen a @Bdentist@B or a dental hygienist?

IWER:
Visits for routine controls, for dentures and stomatology consultations included
SEEN A DENTIST/DENTAL HYGIENIST
1. Yes
5. No

IF HC010_SNaDentist = a1
Txt_FL_HC692

HC692_OOPDentistYesNo
In the last twelve months, did you pay anything yourself for your dental care? Please include payments for diagnoses, treatments, and dental prostheses.
PAYED ANYTHING OUT OF POCKET DENTIST
1. Yes
5. No

IF HC692_OOPDentistYesNo = a1
Txt_FL_HC693

HC693_OOPDentistAmount
Overall, about how much did you pay yourself for your dental care in the last twelve months (that is how much did you pay without getting reimbursed by [a health insurance/your national health system/ a third party payer])?

IWER:
Enter an amount in [FLDefault[9]]
HOW MUCH PAYED OUT OF POCKET DENTIST
-1000000000000000000..1000000000000000000

IF HC693_OOPDentistAmount = NONRESPONSE

BLOCK

IF (((((MN001_Country = a1) OR (MN001_Country = a2)) OR (MN001_Country = a3)) OR (MN001_Country = a5)) OR (MN001_Country = a12)) OR (MN001_Country = a17)

IF piIndex = 1
ELSE

IF piIndex = 2
ELSE

IF piIndex = 3
ELSE

IF piIndex = 4
ELSE

IF piIndex = 5
ELSE

IF piIndex = 6
ELSE
IF piIndex = 7
ELSE
IF piIndex = 8
ELSE
IF piIndex = 9
ENDIF
ENDIF
ENDIF
ENDIF
ENDIF
ENDIF
ENDIF
ENDIF
ENDIF
ELSE
IF ((MN001_Country = a6) OR (MN001_Country = a7)) OR (MN001_Country = a24)
IF piIndex = 1
ELSE
IF piIndex = 2
ELSE
IF piIndex = 3
ELSE
IF piIndex = 4
ELSE
IF piIndex = 5
ELSE
IF piIndex = 6
ELSE
IF piIndex = 7
ELSE
IF piIndex = 8
ELSE
IF piIndex = 9
ENDIF
ENDIF
ENDIF
ENDIF

ENDIF

ENDIF

ENDIF

ENDIF

ENDIF

ELSE

IF MN001_Country = a8

IF piIndex = 1
ELSE

IF piIndex = 2
ELSE

IF piIndex = 3
ELSE

IF piIndex = 4
ELSE

IF piIndex = 5
ELSE

IF piIndex = 6
ELSE

IF piIndex = 7
ELSE

IF piIndex = 8
ELSE

IF piIndex = 9
ENDIF

ENDIF

ENDIF

ENDIF

ENDIF

ENDIF

ENDIF

ENDIF

ENDIF

ELSE

IF ((MN001_Country = a13) OR (MN001_Country = a18)) OR (MN001_Country = a19)

IF piIndex = 1
ELSE

IF piIndex = 2
ELSE
IF piIndex = 3
ELSE
IF piIndex = 4
ELSE
IF piIndex = 5
ELSE
IF piIndex = 6
ELSE
IF piIndex = 7
ELSE
IF piIndex = 8
ELSE
IF piIndex = 9
ENDIF
ENDIF
ENDIF
ENDIF
ENDIF
ENDIF
ENDIF
ENDIF
ENDIF
ELSE
IF MN001_Country = a22
IF piIndex = 1
ELSE
IF piIndex = 2
ELSE
IF piIndex = 3
ELSE
IF piIndex = 4
ELSE
IF piIndex = 5
ELSE
IF piIndex = 6
ELSE
IF piIndex = 7

ELSE

IF piIndex = 8
ELSE

IF piIndex = 9
ENDIF

ENDIF

ENDIF

ENDIF

ENDIF

ENDIF

ENDIF

ENDIF

ENDIF

ELSE

IF MN001_Country = a21

IF piIndex = 1
ELSE

IF piIndex = 2
ELSE

IF piIndex = 3
ELSE

IF piIndex = 4
ELSE

IF piIndex = 5
ELSE

IF piIndex = 6
ELSE

IF piIndex = 7
ELSE

IF piIndex = 8
ELSE

IF piIndex = 9
ENDIF

ENDIF

ENDIF

ENDIF

ENDIF

ENDIF

ENDIF

ENDIF

ENDIF

ELSE

IF MN001_Country = a4

IF piIndex = 1
ELSE

IF piIndex = 2
ELSE

IF piIndex = 3
ELSE

IF piIndex = 4
ELSE

IF piIndex = 5
ELSE

IF piIndex = 6
ELSE

IF piIndex = 7
ELSE

IF piIndex = 8
ELSE

IF piIndex = 9
ENDIF

ENDIF

ENDIF

ENDIF

ENDIF

ENDIF

ENDIF

ENDIF

ENDIF

ELSE

IF MN001_Country = a10

IF piIndex = 1
ELSE

IF piIndex = 2
ELSE

IF piIndex = 3
ELSE
IF piIndex = 4
ELSE
IF piIndex = 5
ELSE
IF piIndex = 6
ELSE
IF piIndex = 7
ELSE
IF piIndex = 8
ELSE
IF piIndex = 9
ENDIF
ENDIF
ENDIF
ENDIF
ENDIF
ENDIF
ENDIF
ENDIF
ENDIF
ELSE
IF MN001_Country = a11
IF piIndex = 1
ELSE
IF piIndex = 2
ELSE
IF piIndex = 3
ELSE
IF piIndex = 4
ELSE
IF piIndex = 5
ELSE
IF piIndex = 6
ELSE
IF piIndex = 7
ELSE
IF piIndex = 8
ELSE

IF piIndex = 9
ENDIF

ENDIF

ENDIF

ENDIF

ENDIF

ENDIF

ENDIF

ENDIF

ENDIF

ELSE

IF MN001_Country = a11

IF piIndex = 1
ELSE

IF piIndex = 2
ELSE

IF piIndex = 3
ELSE

IF piIndex = 4
ELSE

IF piIndex = 5
ELSE

IF piIndex = 6
ELSE

IF piIndex = 7
ELSE

IF piIndex = 8
ELSE

IF piIndex = 9
ENDIF

ENDIF

ENDIF

ENDIF

ENDIF

ENDIF

ENDIF

ENDIF

ENDIF

ELSE

IF MN001_Country = a24

IF piIndex = 1
ELSE

IF piIndex = 2
ELSE

IF piIndex = 3
ELSE

IF piIndex = 4
ELSE

IF piIndex = 5
ELSE

IF piIndex = 6
ELSE

IF piIndex = 7
ELSE

IF piIndex = 8
ELSE

IF piIndex = 9
ENDIF

ENDIF

ENDIF

ENDIF

ENDIF

ENDIF

ENDIF

ENDIF

ENDIF

ELSE

IF MN001_Country = a16

IF piIndex = 1
ELSE

IF piIndex = 2
ELSE

IF piIndex = 3
ELSE

IF piIndex = 4

ELSE
IF [piIndex] = 5
ELSE
IF [piIndex] = 6
ELSE
IF [piIndex] = 7
ELSE
IF [piIndex] = 8
ELSE
IF [piIndex] = 9
ENDIF
ENDIF
ENDIF
ENDIF
ENDIF
ENDIF
ENDIF
ENDIF
ENDIF
ELSE
IF MN001_Country = a16
IF [piIndex] = 1
ELSE
IF [piIndex] = 2
ELSE
IF [piIndex] = 3
ELSE
IF [piIndex] = 4
ELSE
IF [piIndex] = 5
ELSE
IF [piIndex] = 6
ELSE
IF [piIndex] = 7
ELSE
IF [piIndex] = 8
ELSE
IF [piIndex] = 9
ENDIF

ENDIF

ENDIF

ENDIF

ENDIF

ENDIF

ENDIF

ENDIF

ENDIF

ELSE

IF MN001_Country = a14

IF piIndex = 1
ELSE

IF piIndex = 2
ELSE

IF piIndex = 3
ELSE

IF piIndex = 4
ELSE

IF piIndex = 5
ELSE

IF piIndex = 6
ELSE

IF piIndex = 7
ELSE

IF piIndex = 8
ELSE

IF piIndex = 9
ENDIF

ENDIF

ENDIF

ENDIF

ENDIF

ENDIF

ENDIF

ENDIF

ENDIF

ELSE

IF MN001_Country = a15
IF piIndex = 1
ELSE
IF piIndex = 2
ELSE
IF piIndex = 3
ELSE
IF piIndex = 4
ELSE
IF piIndex = 5
ELSE
IF piIndex = 6
ELSE
IF piIndex = 7
ELSE
IF piIndex = 8
ELSE
IF piIndex = 9
ENDIF
ENDIF
ENDIF
ENDIF
ENDIF
ENDIF
ENDIF
ENDIF
ENDIF
ELSE
IF MN001_Country = a20
IF piIndex = 1
ELSE
IF piIndex = 2
ELSE
IF piIndex = 3
ELSE
IF piIndex = 4
ELSE
IF piIndex = 5
ELSE

IF piIndex = 6
ELSE
IF piIndex = 7
ELSE
IF piIndex = 8
ELSE
IF piIndex = 9
ENDIF
ENDIF
ENDIF
ENDIF
ENDIF
ENDIF
ENDIF
ENDIF
ENDIF
ENDIF
ENDIF
ENDIF
ENDIF
ENDIF
ENDIF
ENDIF
ENDIF
ENDIF
ENDIF
ENDIF
ENDIF
ENDIF
ENDIF
ENDIF

A
[Was is less or more than] {Amount} [FLDefault[9]]?
HOW MUCH

1. Less
2. About ^Amount
3. More

ENDBLOCK

ENDIF

ENDIF

*ENDIF*Txt_FL_CO211

CO211_PovertyPostponedDentist
In the last twelve months, to help you keep your living costs down, have you postponed visits to the dentist?
POSTPONED DENTIST
1. Yes
5. No

Txt_FL_HC012

HC012_PTinHos
During the last twelve months, that is since {FLLastYearMonth}, have you been in a hospital overnight? Please consider stays in medical, surgical, psychiatric or in any other specialised wards.
IN HOSPITAL LAST 12 MONTHS
1. Yes
5. No

IF HC012_PTinHos = a1
Txt_FL_HC013

HC013_TiminHos
How many times have you been a patient in a hospital overnight during the last twelve months?

IWER:
Count separate occasions only.
TIMES BEING PATIENT IN HOSPITAL
-1000000000000000000..1000000000000000000
Txt_FL_HC014

HC014_TotNightsinPT
How many nights altogether have you spent in hospitals during the last twelve months?
TOTAL NIGHTS STAYED IN HOSPITAL
1..365

*ENDIF*Txt_FL_HC064

HC064_InOthInstLast12Mon
During the last twelve months, have you been a patient overnight in any health care facility other than a hospital, for instance in institutions for medical rehabilitation, convalescence, etc.? Please do not include stays in nursing homes/residential care facilities.
IN OTHER INSTITUTIONS LAST 12 MONTHS
1. Yes
5. No

IF HC064_InOthInstLast12Mon = a1
Txt_FL_HC066

HC066_TotNightStayOthInst
How many nights altogether have you spent in any institution other than a hospital or a nursing home during the last twelve months?
TOTAL NIGHTS STAYED IN OTHER INSTITUTIONS

1..365

ENDIF
IF (HC064_InOthInstLast12Mon = a1) OR (HC012_PTinHos = a1)

Txt_FL_HC694

HC694_PayedOutOfPocketHospital
Did you pay anything yourself, including for non-care related costs such as meals, [for your stays in hospitals and other health care facilities/ for your hospital stays/ for your stays in health care facilities other than hospitals/ for your stays in hospitals and other health care facilities/ for your hospital stays/ for your stays in health care facilities other than hospitals] in the last twelve months?
PAYED ANYTHING OUT OF POCKET HOSPITAL
1. Yes
5. No

IF HC694_PayedOutOfPocketHospital = a1

Txt_FL_HC695

HC695_HowMuchPayedHospital
Overall, about how much did you pay yourselft for your stays in hospitals or other health care facilities in the last twelve months (that is how much did you pay without getting reimbursed by [a health insurance/your national health system/ a third party payer])?

IWER:
Enter an amount in [FLDefault[9]]
HOW MUCH PAYED OUT OF POCKET HOSPITAL
-1000000000000000000..1000000000000000000
CHECK NOT ((HC695_HowMuchPayedHospital <= 0) AND (HC695_HowMuchPayedHospital = RESPONSE))
L1 " [Amount is expected to be higher than zero]"

IF HC695_HowMuchPayedHospital = NONRESPONSE

BLOCK

IF (((((MN001_Country = a1) OR (MN001_Country = a2)) OR (MN001_Country = a3)) OR (MN001_Country = a5)) OR (MN001_Country = a12)) OR (MN001_Country = a17)

IF piIndex = 1
ELSE

IF piIndex = 2
ELSE

IF piIndex = 3
ELSE

IF piIndex = 4
ELSE

IF piIndex = 5
ELSE

IF piIndex = 6
ELSE

IF piIndex = 7
ELSE

IF piIndex = 8
ELSE

IF piIndex = 9
ENDIF

ENDIF

ENDIF

ENDIF

ENDIF

ENDIF

ENDIF

ENDIF

ENDIF

ELSE

IF ((MN001_Country = a6) OR (MN001_Country = a7)) OR (MN001_Country = a24)

IF piIndex = 1
ELSE

IF piIndex = 2
ELSE

IF piIndex = 3
ELSE

IF piIndex = 4
ELSE

IF piIndex = 5
ELSE

IF piIndex = 6
ELSE

IF piIndex = 7
ELSE

IF piIndex = 8
ELSE

IF piIndex = 9
ENDIF

ENDIF

ENDIF

ENDIF

ENDIF

ENDIF

ENDIF

ENDIF

ENDIF

ELSE

IF MN001_Country = a8

IF piIndex = 1
ELSE

IF piIndex = 2
ELSE

IF piIndex = 3
ELSE

IF piIndex = 4
ELSE

IF piIndex = 5
ELSE

IF piIndex = 6
ELSE

IF piIndex = 7
ELSE

IF piIndex = 8
ELSE

IF piIndex = 9
ENDIF

ENDIF

ENDIF

ENDIF

ENDIF

ENDIF

ENDIF

ENDIF

ENDIF

ELSE

IF ((MN001_Country = a13) OR (MN001_Country = a18)) OR (MN001_Country = a19)

IF piIndex = 1
ELSE

IF piIndex = 2
ELSE

IF piIndex = 3
ELSE

IF piIndex = 4
ELSE

IF piIndex = 5

ELSE

IF piIndex = 6
ELSE

IF piIndex = 7
ELSE

IF piIndex = 8
ELSE

IF piIndex = 9
ENDIF

ENDIF

ENDIF

ENDIF

ENDIF

ENDIF

ENDIF

ENDIF

ENDIF

ELSE

IF MN001_Country = a22

IF piIndex = 1
ELSE

IF piIndex = 2
ELSE

IF piIndex = 3
ELSE

IF piIndex = 4
ELSE

IF piIndex = 5
ELSE

IF piIndex = 6
ELSE

IF piIndex = 7
ELSE

IF piIndex = 8
ELSE

IF piIndex = 9
ENDIF

ENDIF

ENDIF

ENDIF

ENDIF

ENDIF

ENDIF

ENDIF

ENDIF

ELSE

IF MN001_Country = a21

IF piIndex = 1
ELSE

IF piIndex = 2
ELSE

IF piIndex = 3
ELSE

IF piIndex = 4
ELSE

IF piIndex = 5
ELSE

IF piIndex = 6
ELSE

IF piIndex = 7
ELSE

IF piIndex = 8
ELSE

IF piIndex = 9
ENDIF

ENDIF

ENDIF

ENDIF

ENDIF

ENDIF

ENDIF

ENDIF

ENDIF

ELSE

IF MN001_Country = a4

IF piIndex = 1
ELSE
IF piIndex = 2
ELSE
IF piIndex = 3
ELSE
IF piIndex = 4
ELSE
IF piIndex = 5
ELSE
IF piIndex = 6
ELSE
IF piIndex = 7
ELSE
IF piIndex = 8
ELSE
IF piIndex = 9
ENDIF
ENDIF
ENDIF
ENDIF
ENDIF
ENDIF
ENDIF
ENDIF
ENDIF
ELSE
IF MN001_Country = a10
IF piIndex = 1
ELSE
IF piIndex = 2
ELSE
IF piIndex = 3
ELSE
IF piIndex = 4
ELSE
IF piIndex = 5
ELSE
IF piIndex = 6
ELSE

IF piIndex = 7
ELSE
IF piIndex = 8
ELSE
IF piIndex = 9
ENDIF
ENDIF
ENDIF
ENDIF
ENDIF
ENDIF
ENDIF
ENDIF
ENDIF
ELSE
IF MN001_Country = a11
IF piIndex = 1
ELSE
IF piIndex = 2
ELSE
IF piIndex = 3
ELSE
IF piIndex = 4
ELSE
IF piIndex = 5
ELSE
IF piIndex = 6
ELSE
IF piIndex = 7
ELSE
IF piIndex = 8
ELSE
IF piIndex = 9
ENDIF
ENDIF
ENDIF
ENDIF
ENDIF

ENDIF

ENDIF

ENDIF

ENDIF

ELSE

IF MN001_Country = a11

IF piIndex = 1
ELSE

IF piIndex = 2
ELSE

IF piIndex = 3
ELSE

IF piIndex = 4
ELSE

IF piIndex = 5
ELSE

IF piIndex = 6
ELSE

IF piIndex = 7
ELSE

IF piIndex = 8
ELSE

IF piIndex = 9
ENDIF

ENDIF

ENDIF

ENDIF

ENDIF

ENDIF

ENDIF

ENDIF

ENDIF

ELSE

IF MN001_Country = a24

IF piIndex = 1
ELSE

IF piIndex = 2

ELSE

IF piIndex = 3
ELSE

IF piIndex = 4
ELSE

IF piIndex = 5
ELSE

IF piIndex = 6
ELSE

IF piIndex = 7
ELSE

IF piIndex = 8
ELSE

IF piIndex = 9
ENDIF

ENDIF

ENDIF

ENDIF

ENDIF

ENDIF

ENDIF

ENDIF

ENDIF

ELSE

IF MN001_Country = a16

IF piIndex = 1
ELSE

IF piIndex = 2
ELSE

IF piIndex = 3
ELSE

IF piIndex = 4
ELSE

IF piIndex = 5
ELSE

IF piIndex = 6
ELSE

IF piIndex = 7
ELSE

IF piIndex = 8
ELSE
IF piIndex = 9
ENDIF
ENDIF
ENDIF
ENDIF
ENDIF
ENDIF
ENDIF
ENDIF
ENDIF
ELSE
IF MN001_Country = a16
IF piIndex = 1
ELSE
IF piIndex = 2
ELSE
IF piIndex = 3
ELSE
IF piIndex = 4
ELSE
IF piIndex = 5
ELSE
IF piIndex = 6
ELSE
IF piIndex = 7
ELSE
IF piIndex = 8
ELSE
IF piIndex = 9
ENDIF
ENDIF
ENDIF
ENDIF
ENDIF
ENDIF
ENDIF

ENDIF

ENDIF

ELSE

IF MN001_Country = a14

IF piIndex = 1
ELSE

IF piIndex = 2
ELSE

IF piIndex = 3
ELSE

IF piIndex = 4
ELSE

IF piIndex = 5
ELSE

IF piIndex = 6
ELSE

IF piIndex = 7
ELSE

IF piIndex = 8
ELSE

IF piIndex = 9
ENDIF

ENDIF

ENDIF

ENDIF

ENDIF

ENDIF

ENDIF

ENDIF

ENDIF

ELSE

IF MN001_Country = a15

IF piIndex = 1
ELSE

IF piIndex = 2
ELSE

IF piIndex = 3
ELSE

IF piIndex = 4
ELSE
IF piIndex = 5
ELSE
IF piIndex = 6
ELSE
IF piIndex = 7
ELSE
IF piIndex = 8
ELSE
IF piIndex = 9
ENDIF
ENDIF
ENDIF
ENDIF
ENDIF
ENDIF
ENDIF
ENDIF
ENDIF
ELSE
IF MN001_Country = a20
IF piIndex = 1
ELSE
IF piIndex = 2
ELSE
IF piIndex = 3
ELSE
IF piIndex = 4
ELSE
IF piIndex = 5
ELSE
IF piIndex = 6
ELSE
IF piIndex = 7
ELSE
IF piIndex = 8
ELSE
IF piIndex = 9

ENDIF

ENDIF

ENDIF

ENDIF

ENDIF

ENDIF

ENDIF

ENDIF

ENDIF

ENDIF

ENDIF

ENDIF

ENDIF

ENDIF

ENDIF

ENDIF

ENDIF

ENDIF

ENDIF

ENDIF

ENDIF

ENDIF

ENDIF

ENDIF

ENDIF

A
[Was is less or more than] {Amount} [FLDefault[9]]?
HOW MUCH
1. Less
2. About ^Amount
3. More

ENDBLOCK

ENDIF

ENDIF

*ENDIF*Txt_FL_HC140

HC140_WhatAidsAppliancesPhysicalTherapy
Please look at Card 18. In the last 12 months, that is since {FLLastYearMonth}, did you pay anything yourself for items or services mentioned on this card? Please include items that were prescribed by your doctor and those you bought or received without prescription.

IWER:
{CodeAll}
WHAT PAYED OOP AIDS APPLIANCES PHYSICAL THERAPY
1. Aids and appliances (e.g. Wheelchairs; Rollators; Zimmer Frame; Walking Sticks and Crutches; Orthoses; Protheses)
2. Ambulatory therapies (e.g. Physiotherapy; Vocational therapy; Osteopathy; Homeopathy; Counseling Psychology; Chiropractic)
96. None of these

CHECK NOT ((HC140_WhatAidsAppliancesPhysicalTherapy.CARDINAL > 1) AND (a96 IN HC140_WhatAidsAppliancesPhysicalTherapy)) L1 " [You cannot select ""None of the above"" together with any other answer. Please change your answer]"

IF a1 IN HC140_WhatAidsAppliancesPhysicalTherapy
Txt_FL_HC142

HC142_AmountAidsAppliances
How much did you pay overall in the last 12 months, that is since {FLLastYearMonth}, for aids and appliances?
(Please do not include expenses reimbursed by a health insurance.)

IWER:
Includes items prescribed by the doctor and bought without prescription.
Enter an amount in [FLDefault[9]]
HOW MUCH PAYED OOP AIDs, APPLIANCES PHYSICAL THERAPY
-1000000000000000000..1000000000000000000

IF HC142_AmountAidsAppliances = NONRESPONSE

BLOCK

IF (((((MN001_Country = a1) OR (MN001_Country = a2)) OR (MN001_Country = a3)) OR (MN001_Country = a5)) OR (MN001_Country = a12)) OR (MN001_Country = a17)

IF piIndex = 1
ELSE

IF piIndex = 2
ELSE

IF piIndex = 3
ELSE

IF piIndex = 4
ELSE

IF piIndex = 5
ELSE

IF piIndex = 6
ELSE

IF piIndex = 7
ELSE

IF piIndex = 8
ELSE

IF piIndex = 9
ENDIF

ENDIF

ENDIF

ENDIF

ENDIF

ENDIF

ENDIF

ENDIF

ENDIF

ELSE

IF ((MN001_Country = a6) OR (MN001_Country = a7)) OR (MN001_Country = a24)

IF piIndex = 1
ELSE

IF piIndex = 2
ELSE

IF piIndex = 3
ELSE

IF piIndex = 4
ELSE

IF piIndex = 5
ELSE

IF piIndex = 6
ELSE

IF piIndex = 7
ELSE

IF piIndex = 8
ELSE

IF piIndex = 9
ENDIF

ENDIF

ENDIF

ENDIF

ENDIF

ENDIF

ENDIF

ENDIF

ENDIF

ELSE

IF MN001_Country = a8

IF piIndex = 1
ELSE

IF piIndex = 2
ELSE

IF piIndex = 3
ELSE

IF piIndex = 4
ELSE

IF piIndex = 5
ELSE

IF piIndex = 6
ELSE

IF piIndex = 7
ELSE

IF piIndex = 8
ELSE

IF piIndex = 9
ENDIF

ENDIF

ENDIF

ENDIF

ENDIF

ENDIF

ENDIF

ENDIF

ENDIF

ELSE

IF ((MN001_Country = a13) OR (MN001_Country = a18)) OR (MN001_Country = a19)

IF piIndex = 1
ELSE

IF piIndex = 2
ELSE

IF piIndex = 3
ELSE

IF piIndex = 4

ELSE

IF piIndex = 5
ELSE

IF piIndex = 6
ELSE

IF piIndex = 7
ELSE

IF piIndex = 8
ELSE

IF piIndex = 9
ENDIF

ENDIF

ENDIF

ENDIF

ENDIF

ENDIF

ENDIF

ENDIF

ENDIF

ELSE

IF MN001_Country = a22

IF piIndex = 1
ELSE

IF piIndex = 2
ELSE

IF piIndex = 3
ELSE

IF piIndex = 4
ELSE

IF piIndex = 5
ELSE

IF piIndex = 6
ELSE

IF piIndex = 7
ELSE

IF piIndex = 8
ELSE

IF piIndex = 9
ENDIF

ENDIF

ENDIF

ENDIF

ENDIF

ENDIF

ENDIF

ENDIF

ENDIF

ELSE

IF MN001_Country = a21

IF piIndex = 1
ELSE

IF piIndex = 2
ELSE

IF piIndex = 3
ELSE

IF piIndex = 4
ELSE

IF piIndex = 5
ELSE

IF piIndex = 6
ELSE

IF piIndex = 7
ELSE

IF piIndex = 8
ELSE

IF piIndex = 9
ENDIF

ENDIF

ENDIF

ENDIF

ENDIF

ENDIF

ENDIF

ENDIF

ENDIF

ELSE

IF MN001_Country = a4

IF piIndex = 1
ELSE

IF piIndex = 2
ELSE

IF piIndex = 3
ELSE

IF piIndex = 4
ELSE

IF piIndex = 5
ELSE

IF piIndex = 6
ELSE

IF piIndex = 7
ELSE

IF piIndex = 8
ELSE

IF piIndex = 9
ENDIF

ENDIF

ENDIF

ENDIF

ENDIF

ENDIF

ENDIF

ENDIF

ENDIF

ELSE

IF MN001_Country = a10

IF piIndex = 1
ELSE

IF piIndex = 2
ELSE

IF piIndex = 3
ELSE

IF piIndex = 4
ELSE

IF piIndex = 5
ELSE

IF piIndex = 6
ELSE
IF piIndex = 7
ELSE
IF piIndex = 8
ELSE
IF piIndex = 9
ENDIF
ENDIF
ENDIF
ENDIF
ENDIF
ENDIF
ENDIF
ENDIF
ENDIF
ELSE
IF MN001_Country = a11
IF piIndex = 1
ELSE
IF piIndex = 2
ELSE
IF piIndex = 3
ELSE
IF piIndex = 4
ELSE
IF piIndex = 5
ELSE
IF piIndex = 6
ELSE
IF piIndex = 7
ELSE
IF piIndex = 8
ELSE
IF piIndex = 9
ENDIF
ENDIF
ENDIF

ENDIF

ENDIF

ENDIF

ENDIF

ENDIF

ENDIF

ELSE

IF MN001_Country = a11

IF piIndex = 1
ELSE

IF piIndex = 2
ELSE

IF piIndex = 3
ELSE

IF piIndex = 4
ELSE

IF piIndex = 5
ELSE

IF piIndex = 6
ELSE

IF piIndex = 7
ELSE

IF piIndex = 8
ELSE

IF piIndex = 9
ENDIF

ENDIF

ENDIF

ENDIF

ENDIF

ENDIF

ENDIF

ENDIF

ENDIF

ELSE

IF MN001_Country = a24

IF piIndex = 1

ELSE

IF piIndex = 2
ELSE

IF piIndex = 3
ELSE

IF piIndex = 4
ELSE

IF piIndex = 5
ELSE

IF piIndex = 6
ELSE

IF piIndex = 7
ELSE

IF piIndex = 8
ELSE

IF piIndex = 9
ENDIF

ENDIF

ENDIF

ENDIF

ENDIF

ENDIF

ENDIF

ENDIF

ENDIF

ELSE

IF MN001_Country = a16

IF piIndex = 1
ELSE

IF piIndex = 2
ELSE

IF piIndex = 3
ELSE

IF piIndex = 4
ELSE

IF piIndex = 5
ELSE

IF piIndex = 6
ELSE

IF piIndex = 7
ELSE

IF piIndex = 8
ELSE

IF piIndex = 9
ENDIF

ENDIF

ENDIF

ENDIF

ENDIF

ENDIF

ENDIF

ENDIF

ENDIF

ELSE

IF MN001_Country = a16

IF piIndex = 1
ELSE

IF piIndex = 2
ELSE

IF piIndex = 3
ELSE

IF piIndex = 4
ELSE

IF piIndex = 5
ELSE

IF piIndex = 6
ELSE

IF piIndex = 7
ELSE

IF piIndex = 8
ELSE

IF piIndex = 9
ENDIF

ENDIF

ENDIF

ENDIF

ENDIF

ENDIF

ENDIF

ENDIF

ENDIF

ELSE

IF MN001_Country = a14

IF piIndex = 1
ELSE

IF piIndex = 2
ELSE

IF piIndex = 3
ELSE

IF piIndex = 4
ELSE

IF piIndex = 5
ELSE

IF piIndex = 6
ELSE

IF piIndex = 7
ELSE

IF piIndex = 8
ELSE

IF piIndex = 9
ENDIF

ENDIF

ENDIF

ENDIF

ENDIF

ENDIF

ENDIF

ENDIF

ENDIF

ELSE

IF MN001_Country = a15

IF piIndex = 1
ELSE

IF piIndex = 2
ELSE

IF piIndex = 3
ELSE
IF piIndex = 4
ELSE
IF piIndex = 5
ELSE
IF piIndex = 6
ELSE
IF piIndex = 7
ELSE
IF piIndex = 8
ELSE
IF piIndex = 9
ENDIF
ENDIF
ENDIF
ENDIF
ENDIF
ENDIF
ENDIF
ENDIF
ENDIF
ELSE
IF MN001_Country = a20
IF piIndex = 1
ELSE
IF piIndex = 2
ELSE
IF piIndex = 3
ELSE
IF piIndex = 4
ELSE
IF piIndex = 5
ELSE
IF piIndex = 6
ELSE
IF piIndex = 7
ELSE
IF piIndex = 8

ELSE

IF piIndex = 9
ENDIF

ENDIF

ENDIF

ENDIF

ENDIF

ENDIF

ENDIF

ENDIF

ENDIF

ENDIF

ENDIF

ENDIF

ENDIF

ENDIF

ENDIF

ENDIF

ENDIF

ENDIF

ENDIF

ENDIF

ENDIF

ENDIF

ENDIF

ENDIF

ENDIF

A
[Was is less or more than] {Amount} [FLDefault[9]]?
HOW MUCH
1. Less
2. About ^Amount
3. More

ENDBLOCK

ENDIF

ENDIF
IF a2 IN HC140_WhatAidsAppliancesPhysicalTherapy

Txt_FL_HC143

HC143_AmountPhysicalTherapy
How much did you pay overall in the last 12 months, that is since {FLLastYearMonth}, for ambulatory therapies?
(Please do not include expenses reimbursed by a health insurance.)

IWER:
Includes services prescribed by the doctor and bought without prescription
Enter an amount in [FLDefault[9]]
HOW MUCH PAYED OOP AIDs, APPLIANCES PHYSICAL THERAPY
-1000000000000000000..10000000000000000000

IF HC143_AmountPhysicalTherapy = NONRESPONSE

BLOCK

IF (((((MN001_Country = a1) OR (MN001_Country = a2)) OR (MN001_Country = a3)) OR (MN001_Country = a5)) OR (MN001_Country = a12)) OR (MN001_Country = a17)

IF piIndex = 1
ELSE

IF piIndex = 2
ELSE

IF piIndex = 3
ELSE

IF piIndex = 4
ELSE

IF piIndex = 5
ELSE

IF piIndex = 6
ELSE

IF piIndex = 7
ELSE

IF piIndex = 8
ELSE

IF piIndex = 9
ENDIF

ENDIF

ENDIF

ENDIF

ENDIF

ENDIF

ENDIF

ENDIF

ENDIF

ELSE

IF ((MN001_Country = a6) OR (MN001_Country = a7)) OR (MN001_Country = a24)

IF piIndex = 1
ELSE

IF piIndex = 2
ELSE

IF piIndex = 3
ELSE

IF piIndex = 4
ELSE

IF piIndex = 5
ELSE

IF piIndex = 6
ELSE

IF piIndex = 7
ELSE

IF piIndex = 8
ELSE

IF piIndex = 9
ENDIF

ENDIF

ENDIF

ENDIF

ENDIF

ENDIF

ENDIF

ENDIF

ENDIF

ELSE

IF MN001_Country = a8

IF piIndex = 1
ELSE

IF piIndex = 2
ELSE

IF piIndex = 3
ELSE

IF piIndex = 4
ELSE

IF piIndex = 5
ELSE

IF piIndex = 6
ELSE

IF piIndex = 7
ELSE

IF piIndex = 8
ELSE

IF piIndex = 9
ENDIF

ENDIF

ENDIF

ENDIF

ENDIF

ENDIF

ENDIF

ENDIF

ENDIF

ELSE

IF ((MN001_Country = a13) OR (MN001_Country = a18)) OR (MN001_Country = a19)

IF piIndex = 1
ELSE

IF piIndex = 2
ELSE

IF piIndex = 3
ELSE

IF piIndex = 4
ELSE

IF piIndex = 5
ELSE

IF piIndex = 6
ELSE

IF piIndex = 7
ELSE

IF piIndex = 8
ELSE

IF piIndex = 9
ENDIF

ENDIF

ENDIF

ENDIF

ENDIF

ENDIF

ENDIF

ENDIF

ENDIF

ELSE

IF MN001_Country = a22

IF piIndex = 1
ELSE

IF piIndex = 2
ELSE

IF piIndex = 3
ELSE

IF piIndex = 4
ELSE

IF piIndex = 5
ELSE

IF piIndex = 6
ELSE

IF piIndex = 7
ELSE

IF piIndex = 8
ELSE

IF piIndex = 9
ENDIF

ENDIF

ENDIF

ENDIF

ENDIF

ENDIF

ENDIF

ENDIF

ENDIF

ELSE

IF MN001_Country = a21

IF piIndex = 1
ELSE

IF piIndex = 2
ELSE

IF piIndex = 3
ELSE

IF piIndex = 4
ELSE

IF piIndex = 5
ELSE

IF piIndex = 6
ELSE

IF piIndex = 7
ELSE

IF piIndex = 8
ELSE

IF piIndex = 9
ENDIF

ENDIF

ENDIF

ENDIF

ENDIF

ENDIF

ENDIF

ENDIF

ENDIF

ELSE

IF MN001_Country = a4

IF piIndex = 1
ELSE

IF piIndex = 2
ELSE

IF piIndex = 3
ELSE

IF piIndex = 4
ELSE

IF piIndex = 5
ELSE

IF [piIndex](#) = 6
ELSE
IF [piIndex](#) = 7
ELSE
IF [piIndex](#) = 8
ELSE
IF [piIndex](#) = 9
ENDIF
ENDIF
ENDIF
ENDIF
ENDIF
ENDIF
ENDIF
ENDIF
ENDIF
ELSE
IF MN001_Country = a10
IF [piIndex](#) = 1
ELSE
IF [piIndex](#) = 2
ELSE
IF [piIndex](#) = 3
ELSE
IF [piIndex](#) = 4
ELSE
IF [piIndex](#) = 5
ELSE
IF [piIndex](#) = 6
ELSE
IF [piIndex](#) = 7
ELSE
IF [piIndex](#) = 8
ELSE
IF [piIndex](#) = 9
ENDIF
ENDIF
ENDIF
ENDIF

ENDIF

ENDIF

ENDIF

ENDIF

ENDIF

ELSE

IF MN001_Country = a11

IF piIndex = 1
ELSE

IF piIndex = 2
ELSE

IF piIndex = 3
ELSE

IF piIndex = 4
ELSE

IF piIndex = 5
ELSE

IF piIndex = 6
ELSE

IF piIndex = 7
ELSE

IF piIndex = 8
ELSE

IF piIndex = 9
ENDIF

ENDIF

ENDIF

ENDIF

ENDIF

ENDIF

ENDIF

ENDIF

ENDIF

ELSE

IF MN001_Country = a11

IF piIndex = 1
ELSE

IF piIndex = 2
ELSE
IF piIndex = 3
ELSE
IF piIndex = 4
ELSE
IF piIndex = 5
ELSE
IF piIndex = 6
ELSE
IF piIndex = 7
ELSE
IF piIndex = 8
ELSE
IF piIndex = 9
ENDIF
ENDIF
ENDIF
ENDIF
ENDIF
ENDIF
ENDIF
ENDIF
ENDIF
ELSE
IF MN001_Country = a24
IF piIndex = 1
ELSE
IF piIndex = 2
ELSE
IF piIndex = 3
ELSE
IF piIndex = 4
ELSE
IF piIndex = 5
ELSE
IF piIndex = 6
ELSE
IF piIndex = 7

ELSE

IF piIndex = 8
ELSE

IF piIndex = 9
ENDIF

ENDIF

ENDIF

ENDIF

ENDIF

ENDIF

ENDIF

ENDIF

ENDIF

ELSE

IF MN001_Country = a16

IF piIndex = 1
ELSE

IF piIndex = 2
ELSE

IF piIndex = 3
ELSE

IF piIndex = 4
ELSE

IF piIndex = 5
ELSE

IF piIndex = 6
ELSE

IF piIndex = 7
ELSE

IF piIndex = 8
ELSE

IF piIndex = 9
ENDIF

ENDIF

ENDIF

ENDIF

ENDIF

ENDIF

ENDIF

ENDIF

ENDIF

ELSE

IF MN001_Country = a16

IF [piIndex] = 1
ELSE

IF [piIndex] = 2
ELSE

IF [piIndex] = 3
ELSE

IF [piIndex] = 4
ELSE

IF [piIndex] = 5
ELSE

IF [piIndex] = 6
ELSE

IF [piIndex] = 7
ELSE

IF [piIndex] = 8
ELSE

IF [piIndex] = 9
ENDIF

ENDIF

ENDIF

ENDIF

ENDIF

ENDIF

ENDIF

ENDIF

ENDIF

ELSE

IF MN001_Country = a14

IF [piIndex] = 1
ELSE

IF [piIndex] = 2
ELSE

IF piIndex = 3
ELSE
IF piIndex = 4
ELSE
IF piIndex = 5
ELSE
IF piIndex = 6
ELSE
IF piIndex = 7
ELSE
IF piIndex = 8
ELSE
IF piIndex = 9
ENDIF
ENDIF
ENDIF
ENDIF
ENDIF
ENDIF
ENDIF
ENDIF
ENDIF
ELSE
IF MN001_Country = a15
IF piIndex = 1
ELSE
IF piIndex = 2
ELSE
IF piIndex = 3
ELSE
IF piIndex = 4
ELSE
IF piIndex = 5
ELSE
IF piIndex = 6
ELSE
IF piIndex = 7
ELSE
IF piIndex = 8
ELSE

IF piIndex = 9
ENDIF
ENDIF
ENDIF
ENDIF
ENDIF
ENDIF
ENDIF
ENDIF
ENDIF
ELSE
IF MN001_Country = a20
IF piIndex = 1
ELSE
IF piIndex = 2
ELSE
IF piIndex = 3
ELSE
IF piIndex = 4
ELSE
IF piIndex = 5
ELSE
IF piIndex = 6
ELSE
IF piIndex = 7
ELSE
IF piIndex = 8
ELSE
IF piIndex = 9
ENDIF
ENDIF
ENDIF
ENDIF
ENDIF
ENDIF
ENDIF
ENDIF

ENDIF

ENDIF

ENDIF

ENDIF

ENDIF

ENDIF

ENDIF

ENDIF

ENDIF

ENDIF

ENDIF

ENDIF

ENDIF

ENDIF

ENDIF

ENDIF

ENDIF

A
[Was is less or more than] {Amount} [FLDefault[9]]?
HOW MUCH
1. Less
2. About ^Amount
3. More

ENDBLOCK

ENDIF

ENDIF
IF MN024_NursingHome = a1
Txt_FL_HC127

HC127_AtHomeCare
Please look at Card 19. During the last twelve months, that is since {FLLastYearMonth}, did you receive in your own home any professional or paid services listed on this card due to a physical, mental, emotional or memory problem?

IWER:
{CodeAll}
TYPE OF HOME CARE
1. Help with @Bpersonal care@B, (e.g. getting in and out of bed, dressing, bathing and showering)
2. Help with @Bdomestic tasks@B (e.g. cleaning, ironing, cooking)
3. @BMeals-on-wheels@B (i.e. ready made meals provided by a municipality or a private provider)
4. Help with other activities (e.g. filling a drug dispenser)
96. None of the above

CHECK NOT ((HC127_AtHomeCare.CARDINAL > 1) AND (a96 IN HC127_AtHomeCare)) L1 " [You cannot select ""None of the above"" together with any other answer. Please change your answer]"

IF NOT (a96 IN HC127_AtHomeCare)

IF a1 IN HC127_AtHomeCare
ENDIF
IF a2 IN HC127_AtHomeCare
ENDIF
IF a3 IN HC127_AtHomeCare
ENDIF
IF a4 IN HC127_AtHomeCare
*ENDIF*Txt_FL_HC628

HC628_OOP_AtHomeCareYesNo
In the last twelve months, did you pay anything yourself for {FL_HC628_2} without getting reimbursed by your health or long-term care insurance?
PAYED ANYTHING OUT OF POCKET HOME CARE
1. Yes
5. No

IF HC628_OOP_AtHomeCareYesNo = a1
Txt_FL_HC629

HC629_OOP_AtHomeCareAmount
Overall, how much did you pay yourself for {FL_HC628_2} in the last twelve months?

IWER:
Enter an amount in [FLDefault[9]]
AMOUNT OUT OF POCKET HOME CARE
-1000000000000000000..10000000000000000000

IF HC629_OOP_AtHomeCareAmount = DONTKNOW
Txt_FL_HC144

HC144_OOP_MonthlyAtHomeCareAmount
Can you tell me about how much you pay in a typical month for personal care, domestic tasks, meals on wheels or other help you receive in your home?
AMOUNT OUT OF POCKET HOME CARE MONTHLY
-1000000000000000000..10000000000000000000

IF HC144_OOP_MonthlyAtHomeCareAmount = NONRESPONSE

BLOCK

IF (((((MN001_Country = a1) OR (MN001_Country = a2)) OR (MN001_Country = a3)) OR (MN001_Country = a5)) OR (MN001_Country = a12)) OR (MN001_Country = a17)

IF piIndex = 1
ELSE

IF piIndex = 2
ELSE

IF piIndex = 3
ELSE

IF piIndex = 4
ELSE

IF piIndex = 5
ELSE

IF [piIndex](#) = 6
ELSE
IF [piIndex](#) = 7
ELSE
IF [piIndex](#) = 8
ELSE
IF [piIndex](#) = 9
ENDIF
ENDIF
ENDIF
ENDIF
ENDIF
ENDIF
ENDIF
ENDIF
ENDIF
ELSE
IF ((MN001_Country = a6) OR (MN001_Country = a7)) OR (MN001_Country = a24)
IF [piIndex](#) = 1
ELSE
IF [piIndex](#) = 2
ELSE
IF [piIndex](#) = 3
ELSE
IF [piIndex](#) = 4
ELSE
IF [piIndex](#) = 5
ELSE
IF [piIndex](#) = 6
ELSE
IF [piIndex](#) = 7
ELSE
IF [piIndex](#) = 8
ELSE
IF [piIndex](#) = 9
ENDIF
ENDIF
ENDIF

ENDIF

ENDIF

ENDIF

ENDIF

ENDIF

ENDIF

ELSE

IF MN001_Country = a8

IF piIndex = 1
ELSE

IF piIndex = 2
ELSE

IF piIndex = 3
ELSE

IF piIndex = 4
ELSE

IF piIndex = 5
ELSE

IF piIndex = 6
ELSE

IF piIndex = 7
ELSE

IF piIndex = 8
ELSE

IF piIndex = 9
ENDIF

ENDIF

ENDIF

ENDIF

ENDIF

ENDIF

ENDIF

ENDIF

ENDIF

ELSE

IF ((MN001_Country = a13) OR (MN001_Country = a18)) OR (MN001_Country = a19)

IF piIndex = 1

ELSE

IF piIndex = 2
ELSE

IF piIndex = 3
ELSE

IF piIndex = 4
ELSE

IF piIndex = 5
ELSE

IF piIndex = 6
ELSE

IF piIndex = 7
ELSE

IF piIndex = 8
ELSE

IF piIndex = 9
ENDIF

ENDIF

ENDIF

ENDIF

ENDIF

ENDIF

ENDIF

ENDIF

ENDIF

ELSE

IF MN001_Country = a22

IF piIndex = 1
ELSE

IF piIndex = 2
ELSE

IF piIndex = 3
ELSE

IF piIndex = 4
ELSE

IF piIndex = 5
ELSE

IF piIndex = 6
ELSE

IF piIndex = 7
ELSE
IF piIndex = 8
ELSE
IF piIndex = 9
ENDIF
ENDIF
ENDIF
ENDIF
ENDIF
ENDIF
ENDIF
ENDIF
ENDIF
ELSE
IF MN001_Country = a21
IF piIndex = 1
ELSE
IF piIndex = 2
ELSE
IF piIndex = 3
ELSE
IF piIndex = 4
ELSE
IF piIndex = 5
ELSE
IF piIndex = 6
ELSE
IF piIndex = 7
ELSE
IF piIndex = 8
ELSE
IF piIndex = 9
ENDIF
ENDIF
ENDIF
ENDIF
ENDIF

ENDIF

ENDIF

ENDIF

ENDIF

ELSE

IF MN001_Country = a4

IF [piIndex]() = 1
ELSE

IF [piIndex]() = 2
ELSE

IF [piIndex]() = 3
ELSE

IF [piIndex]() = 4
ELSE

IF [piIndex]() = 5
ELSE

IF [piIndex]() = 6
ELSE

IF [piIndex]() = 7
ELSE

IF [piIndex]() = 8
ELSE

IF [piIndex]() = 9
ENDIF

ENDIF

ENDIF

ENDIF

ENDIF

ENDIF

ENDIF

ENDIF

ENDIF

ELSE

IF MN001_Country = a10

IF [piIndex]() = 1
ELSE

IF [piIndex]() = 2
ELSE

IF piIndex = 3
ELSE
IF piIndex = 4
ELSE
IF piIndex = 5
ELSE
IF piIndex = 6
ELSE
IF piIndex = 7
ELSE
IF piIndex = 8
ELSE
IF piIndex = 9
ENDIF
ENDIF
ENDIF
ENDIF
ENDIF
ENDIF
ENDIF
ENDIF
ENDIF
ELSE
IF MN001_Country = a11
IF piIndex = 1
ELSE
IF piIndex = 2
ELSE
IF piIndex = 3
ELSE
IF piIndex = 4
ELSE
IF piIndex = 5
ELSE
IF piIndex = 6
ELSE
IF piIndex = 7
ELSE
IF piIndex = 8

ELSE

IF piIndex = 9
ENDIF

ENDIF

ENDIF

ENDIF

ENDIF

ENDIF

ENDIF

ENDIF

ENDIF

ELSE

IF MN001_Country = a11

IF piIndex = 1
ELSE

IF piIndex = 2
ELSE

IF piIndex = 3
ELSE

IF piIndex = 4
ELSE

IF piIndex = 5
ELSE

IF piIndex = 6
ELSE

IF piIndex = 7
ELSE

IF piIndex = 8
ELSE

IF piIndex = 9
ENDIF

ENDIF

ENDIF

ENDIF

ENDIF

ENDIF

ENDIF

ENDIF

ENDIF

ELSE

IF MN001_Country = a24

IF piIndex = 1
ELSE

IF piIndex = 2
ELSE

IF piIndex = 3
ELSE

IF piIndex = 4
ELSE

IF piIndex = 5
ELSE

IF piIndex = 6
ELSE

IF piIndex = 7
ELSE

IF piIndex = 8
ELSE

IF piIndex = 9
ENDIF

ENDIF

ENDIF

ENDIF

ENDIF

ENDIF

ENDIF

ENDIF

ENDIF

ELSE

IF MN001_Country = a16

IF piIndex = 1
ELSE

IF piIndex = 2
ELSE

IF piIndex = 3
ELSE

IF piIndex = 4
ELSE
IF piIndex = 5
ELSE
IF piIndex = 6
ELSE
IF piIndex = 7
ELSE
IF piIndex = 8
ELSE
IF piIndex = 9
ENDIF
ENDIF
ENDIF
ENDIF
ENDIF
ENDIF
ENDIF
ENDIF
ENDIF
ELSE
IF MN001_Country = a16
IF piIndex = 1
ELSE
IF piIndex = 2
ELSE
IF piIndex = 3
ELSE
IF piIndex = 4
ELSE
IF piIndex = 5
ELSE
IF piIndex = 6
ELSE
IF piIndex = 7
ELSE
IF piIndex = 8
ELSE
IF piIndex = 9
ENDIF

ENDIF

ENDIF

ENDIF

ENDIF

ENDIF

ENDIF

ENDIF

ENDIF

ELSE

IF MN001_Country = a14

IF piIndex = 1
ELSE

IF piIndex = 2
ELSE

IF piIndex = 3
ELSE

IF piIndex = 4
ELSE

IF piIndex = 5
ELSE

IF piIndex = 6
ELSE

IF piIndex = 7
ELSE

IF piIndex = 8
ELSE

IF piIndex = 9
ENDIF

ENDIF

ENDIF

ENDIF

ENDIF

ENDIF

ENDIF

ENDIF

ENDIF

ELSE

IF MN001_Country = a15

IF [piIndex] = 1
ELSE

IF [piIndex] = 2
ELSE

IF [piIndex] = 3
ELSE

IF [piIndex] = 4
ELSE

IF [piIndex] = 5
ELSE

IF [piIndex] = 6
ELSE

IF [piIndex] = 7
ELSE

IF [piIndex] = 8
ELSE

IF [piIndex] = 9
ENDIF

ENDIF

ENDIF

ENDIF

ENDIF

ENDIF

ENDIF

ENDIF

ENDIF

ELSE

IF MN001_Country = a20

IF [piIndex] = 1
ELSE

IF [piIndex] = 2
ELSE

IF [piIndex] = 3
ELSE

IF [piIndex] = 4
ELSE

IF [piIndex] = 5

ELSE

IF piIndex = 6
ELSE

IF piIndex = 7
ELSE

IF piIndex = 8
ELSE

IF piIndex = 9
ENDIF

ENDIF

ENDIF

ENDIF

ENDIF

ENDIF

ENDIF

ENDIF

ENDIF

ENDIF

ENDIF

ENDIF

ENDIF

ENDIF

ENDIF

ENDIF

ENDIF

ENDIF

ENDIF

ENDIF

ENDIF

ENDIF

ENDIF

ENDIF

ENDIF

A
[Was is less or more than] {Amount} [FLDefault[9]]?

HOW MUCH
1. Less
2. About ^Amount
3. More

ENDBLOCK

ENDIF

ENDIF

ENDIF

*ENDIF*Txt_FL_HC029

HC029_NursHome
During the last twelve months, that is since {FLLastYearMonth}, have you been in a nursing home/residential care facility overnight?

IWER:
When a respondent definitely moved to a nursing home less than 12 months ago, answer 1 (yes temporarily)
IN A NURSING HOME
1. Yes, temporarily
3. Yes, permanently
5. No

CHECK NOT (HC029_NursHome = a3) L1 " [At the beginning of this interview you entered that the R""s home is not a nursing home. Now you have entered that the R lives permanently in a nursing home. Please enter a remark to explain]"

IF HC029_NursHome = a1
Txt_FL_HC031

HC031_WksNursHome
During the last 12 months, how many weeks altogether did you stay in a nursing home?

IWER:
Count 4 weeks for each full month; count 1 for part of one week
WEEKS STAYED IN A NURSING HOME
1..52

ENDIF
IF (HC029_NursHome = a1) OR (HC029_NursHome = a3)

IF a96 IN HC116_LongTermCareInsurance
ELSE
*ENDIF*Txt_FL_HC696

HC696_OOP_NursingHomeYesNo
Did you pay anything yourself for nursing home stays in the last twelve months?
PAYED ANYTHING OUT OF POCKET NURSING HOME
1. Yes
5. No

IF HC696_OOP_NursingHomeYesNo = a1
Txt_FL_HC097

HC097_OOP_NursingHomeAmount
How much did you pay overall for your nursing home stays in the last twelve months?

IWER:
Enter an amount in [FLDefault[9]]

HOW MUCH PAYED OUT OF POCKET NURSING HOME
-100000000000000000..1000000000000000000

IF HC097_OOP_NursingHomeAmount = NONRESPONSE

BLOCK

IF (((((MN001_Country = a1) OR (MN001_Country = a2)) OR (MN001_Country = a3)) OR (MN001_Country = a5)) OR (MN001_Country = a12)) OR (MN001_Country = a17)

IF piIndex = 1
ELSE

IF piIndex = 2
ELSE

IF piIndex = 3
ELSE

IF piIndex = 4
ELSE

IF piIndex = 5
ELSE

IF piIndex = 6
ELSE

IF piIndex = 7
ELSE

IF piIndex = 8
ELSE

IF piIndex = 9
ENDIF

ENDIF

ENDIF

ENDIF

ENDIF

ENDIF

ENDIF

ENDIF

ENDIF

ELSE

IF ((MN001_Country = a6) OR (MN001_Country = a7)) OR (MN001_Country = a24)

IF piIndex = 1
ELSE

IF piIndex = 2
ELSE

IF piIndex = 3

ELSE

IF piIndex = 4
ELSE

IF piIndex = 5
ELSE

IF piIndex = 6
ELSE

IF piIndex = 7
ELSE

IF piIndex = 8
ELSE

IF piIndex = 9
ENDIF

ENDIF

ENDIF

ENDIF

ENDIF

ENDIF

ENDIF

ENDIF

ENDIF

ELSE

IF MN001_Country = a8

IF piIndex = 1
ELSE

IF piIndex = 2
ELSE

IF piIndex = 3
ELSE

IF piIndex = 4
ELSE

IF piIndex = 5
ELSE

IF piIndex = 6
ELSE

IF piIndex = 7
ELSE

IF piIndex = 8
ELSE

IF piIndex = 9
ENDIF
ENDIF
ENDIF
ENDIF
ENDIF
ENDIF
ENDIF
ENDIF
ENDIF
ELSE
IF ((MN001_Country = a13) OR (MN001_Country = a18)) OR (MN001_Country = a19)
IF piIndex = 1
ELSE
IF piIndex = 2
ELSE
IF piIndex = 3
ELSE
IF piIndex = 4
ELSE
IF piIndex = 5
ELSE
IF piIndex = 6
ELSE
IF piIndex = 7
ELSE
IF piIndex = 8
ELSE
IF piIndex = 9
ENDIF
ENDIF
ENDIF
ENDIF
ENDIF
ENDIF
ENDIF
ENDIF

ENDIF

ELSE

IF MN001_Country = a22

IF piIndex = 1
ELSE

IF piIndex = 2
ELSE

IF piIndex = 3
ELSE

IF piIndex = 4
ELSE

IF piIndex = 5
ELSE

IF piIndex = 6
ELSE

IF piIndex = 7
ELSE

IF piIndex = 8
ELSE

IF piIndex = 9
ENDIF

ENDIF

ENDIF

ENDIF

ENDIF

ENDIF

ENDIF

ENDIF

ENDIF

ELSE

IF MN001_Country = a21

IF piIndex = 1
ELSE

IF piIndex = 2
ELSE

IF piIndex = 3
ELSE

IF piIndex = 4
ELSE

IF piIndex = 5
ELSE
IF piIndex = 6
ELSE
IF piIndex = 7
ELSE
IF piIndex = 8
ELSE
IF piIndex = 9
ENDIF
ENDIF
ENDIF
ENDIF
ENDIF
ENDIF
ENDIF
ENDIF
ENDIF
ELSE
IF MN001_Country = a4
IF piIndex = 1
ELSE
IF piIndex = 2
ELSE
IF piIndex = 3
ELSE
IF piIndex = 4
ELSE
IF piIndex = 5
ELSE
IF piIndex = 6
ELSE
IF piIndex = 7
ELSE
IF piIndex = 8
ELSE
IF piIndex = 9
ENDIF
ENDIF

ENDIF

ENDIF

ENDIF

ENDIF

ENDIF

ENDIF

ENDIF

ELSE

IF MN001_Country = a10

IF piIndex = 1
ELSE

IF piIndex = 2
ELSE

IF piIndex = 3
ELSE

IF piIndex = 4
ELSE

IF piIndex = 5
ELSE

IF piIndex = 6
ELSE

IF piIndex = 7
ELSE

IF piIndex = 8
ELSE

IF piIndex = 9
ENDIF

ENDIF

ENDIF

ENDIF

ENDIF

ENDIF

ENDIF

ENDIF

ENDIF

ELSE

IF MN001_Country = a11

IF piIndex = 1
ELSE

IF piIndex = 2
ELSE

IF piIndex = 3
ELSE

IF piIndex = 4
ELSE

IF piIndex = 5
ELSE

IF piIndex = 6
ELSE

IF piIndex = 7
ELSE

IF piIndex = 8
ELSE

IF piIndex = 9
ENDIF

ENDIF

ENDIF

ENDIF

ENDIF

ENDIF

ENDIF

ENDIF

ENDIF

ELSE

IF MN001_Country = a11

IF piIndex = 1
ELSE

IF piIndex = 2
ELSE

IF piIndex = 3
ELSE

IF piIndex = 4
ELSE

IF piIndex = 5
ELSE

IF piIndex = 6
ELSE
IF piIndex = 7
ELSE
IF piIndex = 8
ELSE
IF piIndex = 9
ENDIF
ENDIF
ENDIF
ENDIF
ENDIF
ENDIF
ENDIF
ENDIF
ENDIF
ELSE
IF MN001_Country = a24
IF piIndex = 1
ELSE
IF piIndex = 2
ELSE
IF piIndex = 3
ELSE
IF piIndex = 4
ELSE
IF piIndex = 5
ELSE
IF piIndex = 6
ELSE
IF piIndex = 7
ELSE
IF piIndex = 8
ELSE
IF piIndex = 9
ENDIF
ENDIF
ENDIF
ENDIF

ENDIF

ENDIF

ENDIF

ENDIF

ENDIF

ELSE

IF MN001_Country = a16

IF [piIndex]() = 1
ELSE

IF [piIndex]() = 2
ELSE

IF [piIndex]() = 3
ELSE

IF [piIndex]() = 4
ELSE

IF [piIndex]() = 5
ELSE

IF [piIndex]() = 6
ELSE

IF [piIndex]() = 7
ELSE

IF [piIndex]() = 8
ELSE

IF [piIndex]() = 9
ENDIF

ENDIF

ENDIF

ENDIF

ENDIF

ENDIF

ENDIF

ENDIF

ENDIF

ELSE

IF MN001_Country = a16

IF [piIndex]() = 1
ELSE

IF piIndex = 2
ELSE
IF piIndex = 3
ELSE
IF piIndex = 4
ELSE
IF piIndex = 5
ELSE
IF piIndex = 6
ELSE
IF piIndex = 7
ELSE
IF piIndex = 8
ELSE
IF piIndex = 9
ENDIF
ENDIF
ENDIF
ENDIF
ENDIF
ENDIF
ENDIF
ENDIF
ENDIF
ELSE
IF MN001_Country = a14
IF piIndex = 1
ELSE
IF piIndex = 2
ELSE
IF piIndex = 3
ELSE
IF piIndex = 4
ELSE
IF piIndex = 5
ELSE
IF piIndex = 6
ELSE
IF piIndex = 7

ELSE

IF piIndex = 8
ELSE

IF piIndex = 9
ENDIF

ENDIF

ENDIF

ENDIF

ENDIF

ENDIF

ENDIF

ENDIF

ENDIF

ELSE

IF MN001_Country = a15

IF piIndex = 1
ELSE

IF piIndex = 2
ELSE

IF piIndex = 3
ELSE

IF piIndex = 4
ELSE

IF piIndex = 5
ELSE

IF piIndex = 6
ELSE

IF piIndex = 7
ELSE

IF piIndex = 8
ELSE

IF piIndex = 9
ENDIF

ENDIF

ENDIF

ENDIF

ENDIF

ENDIF

ENDIF

ENDIF

ENDIF

ELSE

IF MN001_Country = a20

IF piIndex = 1
ELSE

IF piIndex = 2
ELSE

IF piIndex = 3
ELSE

IF piIndex = 4
ELSE

IF piIndex = 5
ELSE

IF piIndex = 6
ELSE

IF piIndex = 7
ELSE

IF piIndex = 8
ELSE

IF piIndex = 9
ENDIF

ENDIF

ENDIF

ENDIF

ENDIF

ENDIF

ENDIF

ENDIF

ENDIF

ENDIF

ENDIF

ENDIF

ENDIF

ENDIF

ENDIF

ENDIF

ENDIF

ENDIF

ENDIF

ENDIF

ENDIF

ENDIF

ENDIF

ENDIF

ENDIF

A
[Was is less or more than] {Amount} [FLDefault[9]]?
HOW MUCH
1. Less
2. About ^Amount
3. More

ENDBLOCK

ENDIF

ENDIF

ENDIF

ENDIF
System preset value HC902_TimeStampEnd
IF HC902_TimeStampEnd = EMPTY
ENDIF

ENDBLOCK

ENDIF
IF MN029_linkage = 1

BLOCK

IF ((MN001_Country = a2) OR (MN001_Country = a5)) OR (MN001_Country = a21)

LI006_consent
Intro and consent question (EACH COUNTRY FILLS IN COUNTRY SPECIFIC CONSENT INTRO AND CONSENT QUESTION)
LINKAGE CONSENT QUESTION
1. Consent
5. No consent

IF (MN001_Country = a2) AND (LI006_consent = a1)

LI007_SSN
What is your Social Security Number?

IWER:
Ask for Social Security Number and enter the 10 digit SSN into CAPI
if SSN is not accepted, set a remark by pressing Ctrl+M. Then press Ctrl+K to continue with the interview.
SOCIAL SECURITY NUMBER
STRING[10]

IF LI007_SSN = RESPONSE
CHECK_ID

IF checked = 0
CHECK ERROR INVOLVING (LI007_SSN) L1 " [SSN is incorrect please try again!]"

ENDIF

ENDIF

ENDIF

ELSE

IF (((MN001_Country = a3) OR (MN001_Country = a4)) OR (MN001_Country = a19)) OR (MN001_Country = a23)

LI004_Intro
We are now changing the topic. The researchers of this study are interested in people's employment history. Important research questions could be answered with data collected by the [German Pension Fund]. We would like to link interview responses with data of the [German Pension Fund]. For reasons of data protection, this cannot be done without your consent. Giving us your permission is completely voluntary. I would kindly ask your consent to do this.

Please take a few minutes and to read this form.

IWER:
Take the 2 consent forms and hand out 1 to the respondent.
LINKING INTRO
1. Continue

IF ((MN001_Country = a3) OR (MN001_Country = a4)) OR (MN001_Country = a23)

LI001_Number

IWER:
Take the other consent form and enter the 6 digit key number (on the top right of the form) into CAPI.
ID RECORD LINKAGE
STRING[6]

LI002_Number_Check

IWER:
REPEAT THE NUMBER.
ID RECORD LINKAGE AGAIN
STRING[6]
CHECK LI001_Number = LI002_Number_Check L1 " [values should be equal]"

ENDIF

LI003_Consent

IWER:

Did R consent to the record linkage?
Assist respondent if necessary. Cross the form if R refuses.
Please insert the consent form in the envelope [addressed DRV] and bring it to the mail box.
The blank form remains with respondent.
LINKAGE COMPLETED
1. Yes. Respondent consented, completed the form and returned the form to me in the envelope.
2. Unclear. Respondent may complete the form later and sent it back himself/herself.
5. No, respondent did not consent to record linkage (Cross the form and send it back anyway).

ENDIF

ENDIF

ENDBLOCK

ENDIF
IF (EP IN Test) OR (ALL IN Test)

BLOCK

IF MN024_NursingHome = a1
Txt_FL_EP001

EP001_Intro
Now I am going to ask you some questions about your current employment situation.
INTRODUCTION EMPLOYMENT AND PENSIONS
1. Continue

System preset value EP901_TimeStampStart
IF EP901_TimeStampStart = EMPTY AND EP001_Intro <> EMPTY
*ENDIF*Txt_FL_EP005

EP005_CurrentJobSit
Please look at card 7. In general, which of the following best describes your @bcurrent@b employment situation?

IWER:
Code only one
Only if R in doubt then refer to the following:
1. Retired (retired from own work, including semi-retired, partially retired, early retired, pre-retired). Retired refers to retired from own work only. Recipients of survivor pensions who do not receive pensions from own work should not be coded as retired. If they do not fit in categories 2 through 5, they should go into other.
CURRENT JOB SITUATION
1. Retired
2. Employed or self-employed (including working for family business)
3. Unemployed
4. Permanently sick or disabled
5. Homemaker
97. Other

IF EP005_CurrentJobSit = a1
Txt_FL_EP329

EP329_RetYear
In which year did you retire?
RETIREMENT YEAR
1900..2015
CHECK NOT (EP329_RetYear < (MN002_Person[1].Year + 16)) L1 " [Retirement year lies before 16th birthday. If year is correct, please press "suppress" and enter a remark to explain]"

IF EP329_RetYear > 2008

Txt_FL_EP328

EP328_RetMonth
Do you remember in what month that was?
RETIREMENT MONTH
1. January
2. February
3. March
4. April
5. May
6. June
7. July
8. August
9. September
10. October
11. November
12. December

ENDIF
IF (MN101_Longitudinal = 0) OR ((MN101_Longitudinal = 1) AND ((Preload.InterviewYear_Last < EP329_RetYear) OR ((Preload.InterviewYear_Last = EP329_RetYear) AND (Preload.InterviewMonth_Last <= EP328_RetMonth))))

Txt_FL_EP064

EP064_ResForRet
Please look at card 20.
For which reasons did you retire?

IWER:
{CodeAll}
MAIN REASON FOR EARLY RETIREMENT
1. Became eligible for public pension
2. Became eligible for private occupational pension
3. Became eligible for a private pension
4. Was offered an early retirement option/window with special incentives or bonus
5. Made redundant (for example pre-retirement)
6. Own ill health
7. Ill health of relative or friend
8. To retire at same time as spouse or partner
9. To spend more time with family
10. To enjoy life

ENDIF

ENDIF
IF EP005_CurrentJobSit = a3

Txt_FL_EP337

EP337_LookingForJob
Are you currently looking for a job?
LOOKING FOR JOB
1. Yes
5. No

Txt_FL_EP067

EP067_HowUnempl
Would you tell us how you became unemployed? Was it

IWER:
{ReadOut}
For seasonal workers code 5

HOW BECAME UNEMPLOYED
1. Because your place of work or office closed
2. Because you resigned
3. Because you were laid off
4. By mutual agreement between you and your employer
5. Because a temporary job had been completed
6. Because you moved to another town
97. Other reason

ENDIF
IF EP005_CurrentJobSit <> a2
Txt_FL_EP002

EP002_PaidWork
[We are interested in your work experiences since our last interview.] Did you do any paid work [since our last interview in/ during the last four weeks], either as an employee or self-employed, even if this was only for a few hours?
DID ANY PAID WORK
1. Yes
5. No

ENDIF
IF MN101_Longitudinal = 0

IF (((EP005_CurrentJobSit.ORD = 4) OR (EP005_CurrentJobSit.ORD = 5)) OR (EP005_CurrentJobSit.ORD = 97.00000000000001)) AND (EP002_PaidWork = a5)
Txt_FL_EP006

EP006_EverWorked
Have you ever done any paid work?
EVER DONE PAID WORK
1. Yes
5. No

ENDIF

ENDIF
IF MN101_Longitudinal = 1

IF (EP005_CurrentJobSit = a2) OR (EP002_PaidWork = a1)
Txt_FL_EP125

EP125_ContWork
I'd like to know about all of the work for pay that you may have done since {FLLastInterviewMonthYear} through the present. During that time, have you been working continuously?

IWER:
Vacation period should not be counted as interruptions.
CONTINUOUSLY WORKING
1. Yes
5. No

ENDIF
IF EP125_ContWork = a1
Txt_FL_EP141

EP141_ChangeInJob
Please look at card 21. Even though you have been working continuously since {FLLastInterviewMonthYear}, have you experienced any of the changes listed on this card?

IWER:
{CodeAll}
CHANGE IN JOB
1. A change in type of employment (for instance from dependent employment to self-employment)
2. A change in employer
3. A promotion
4. A change in job location
5. A change in contract length (from long term to short term or viceversa)
96. None of the above

CHECK NOT ((EP141_ChangeInJob.CARDINAL > 1) AND (96 IN EP141_ChangeInJob)) L1 " [You cannot select ""None of the above"" together with any other answer. Please change your answer]"

ENDIF
IF EP125_ContWork = a5

BLOCK
Txt_FL_EP127

EP127_PeriodFromMonth
From what @bmonth@b and year have you been [working/ unemployed]?

@bMONTH@b:
YEAR:
PERIOD FROM MONTH
1. January
2. February
3. March
4. April
5. May
6. June
7. July
8. August
9. September
10. October
11. November
12. December

Txt_FL_EP128

EP128_PeriodFromYear
From what month and @byear@b have you been [working/ unemployed]?

MONTH {EP127_PeriodFromMonth}
@bYEAR@b
PERIOD FROM YEAR
1. 2005 or earlier
2. 2006
3. 2007
4. 2008
5. 2009
6. 2010
7. 2011
8. 2012
9. 2013
10. 2014
11. 2015

Txt_FL_EP129

EP129_PeriodToMonth
To what @bmonth@b and year have you been [working/ unemployed]?

@bMONTH@b:

YEAR:

IWER:
If spell still ongoing type 13. Today
PERIOD TO MONTH
1. January
2. February
3. March
4. April
5. May
6. June
7. July
8. August
9. September
10. October
11. November
12. December
13. Today

IF EP129_PeriodToMonth <> a13
Txt_FL_EP130

EP130_PeriodToYear
To what month and @byear@b have you been [working/ unemployed]?

MONTH: {EP129_PeriodToMonth}

@bYEAR@b:

IWER:
To year
PERIOD TO YEAR
1. 2005 or earlier
2. 2006
3. 2007
4. 2008
5. 2009
6. 2010
7. 2011
8. 2012
9. 2013
10. 2014
11. 2015

*ENDIF*Txt_FL_EP133

EP133_PeriodOtherEp
Were there other times since {FLLastInterviewMonthYear} when you have been [working for pay/ unemployed]?
OTHER PERIODS
1. Yes
5. No

ENDBLOCK
IF PeriodOtherEpisodes[1].EP129_PeriodToMonth = a13
ENDIF
LOOP cnt:= 2 TO 20

IF PeriodOtherEpisodes[cnt - 1].EP133_PeriodOtherEp = a1

BLOCK

Txt_FL_EP127

EP127_PeriodFromMonth

From what @bmonth@b and year have you been [working/ unemployed]?

@bMONTH@b:
YEAR:
PERIOD FROM MONTH

1. January
2. February
3. March
4. April
5. May
6. June
7. July
8. August
9. September
10. October
11. November
12. December

Txt_FL_EP128

EP128_PeriodFromYear

From what month and @byear@b have you been [working/ unemployed]?

MONTH {EP127_PeriodFromMonth}
@bYEAR@b
PERIOD FROM YEAR

1. 2005 or earlier
2. 2006
3. 2007
4. 2008
5. 2009
6. 2010
7. 2011
8. 2012
9. 2013
10. 2014
11. 2015

Txt_FL_EP129

EP129_PeriodToMonth

To what @bmonth@b and year have you been [working/ unemployed]?

@bMONTH@b:
YEAR:

IWER:
If spell still ongoing type 13. Today
PERIOD TO MONTH

1. January
2. February
3. March
4. April
5. May
6. June
7. July
8. August
9. September
10. October
11. November

12. December
13. Today

IF EP129_PeriodToMonth <> a13

Txt_FL_EP130

EP130_PeriodToYear
To what month and @byear@b have you been [working/ unemployed]?

MONTH: {EP129_PeriodToMonth}

@bYEAR@b:

IWER:
To year
PERIOD TO YEAR
1. 2005 or earlier
2. 2006
3. 2007
4. 2008
5. 2009
6. 2010
7. 2011
8. 2012
9. 2013
10. 2014
11. 2015

*ENDIF*Txt_FL_EP133

EP133_PeriodOtherEp
Were there other times since {FLLastInterviewMonthYear} when you have been [working for pay/ unemployed]?
OTHER PERIODS
1. Yes
5. No

ENDBLOCK
IF PeriodOtherEpisodes[cnt].EP129_PeriodToMonth = a13
ENDIF

ENDIF

ENDLOOP

ENDIF

ENDIF
IF (((MN101_Longitudinal = 0) AND (EP006_EverWorked = a1)) AND (EP005_CurrentJobSit = a5)) OR ((((MN101_Longitudinal = 1) AND (EP005_CurrentJobSit = a5)) AND (EP002_PaidWork = a1)) AND (EP335_Today = a5))

Txt_FL_EP069

EP069_ResStopWork
You said you are currently a homemaker, but you have done paid work in the past. Why did you stop working?

IWER:
{ReadOut} {CodeAll}
REASON STOP WORKING

1. Because of health problems
2. It was too tiring
3. It was too expensive to hire someone to look after home or family
4. Because you wanted to take care of children or grandchildren
5. Because you were laid off, or your place of work or office closed
6. Because family income was sufficient
7. To care for an old or sick family member
97. Other

ENDIF
IF MN101_Longitudinal = 1

IF ((EP005_CurrentJobSit <> a3) AND ((EP125_ContWork = a5) OR ((EP005_CurrentJobSit <> a2) AND (EP002_PaidWork = a5)))) AND (MN808_AgeRespondent <= 75)

Txt_FL_EP325

EP325_UnEmpl
Were there any times since {FLLastInterviewMonthYear}, when you were unemployed?
UNEMPLOYED
1. Yes
5. No

ENDIF
IF EP005_CurrentJobSit = a3

Txt_FL_EP632

EP632_Intro
Now I'd like to know about the times since our last interview through the present in which you were unemployed.
INTRODUCTION WHEN UNEMPLOYED
1. Continue

ENDIF
IF (EP325_UnEmpl = a1) OR (EP005_CurrentJobSit = a3)

Txt_FL_EP633

EP633_Intro
When were you unemployed? Please give me all of your start and stop dates.
INTRODUCTION DATES UNEMPLOYED
1. Continue

BLOCK

Txt_FL_EP127

EP127_PeriodFromMonth
From what @bmonth@b and year have you been [working/ unemployed]?

@bMONTH@b:
YEAR:
PERIOD FROM MONTH
1. January
2. February
3. March
4. April
5. May
6. June
7. July
8. August
9. September
10. October

11. November
12. December

Txt_FL_EP128

EP128_PeriodFromYear
From what month and @byear@b have you been [working/ unemployed]?

MONTH {EP127_PeriodFromMonth}
@bYEAR@b
PERIOD FROM YEAR
1. 2005 or earlier
2. 2006
3. 2007
4. 2008
5. 2009
6. 2010
7. 2011
8. 2012
9. 2013
10. 2014
11. 2015

Txt_FL_EP129

EP129_PeriodToMonth
To what @bmonth@b and year have you been [working/ unemployed]?

@bMONTH@b:
YEAR:

IWER:
If spell still ongoing type 13. Today
PERIOD TO MONTH
1. January
2. February
3. March
4. April
5. May
6. June
7. July
8. August
9. September
10. October
11. November
12. December
13. Today

IF EP129_PeriodToMonth <> a13

Txt_FL_EP130

EP130_PeriodToYear
To what month and @byear@b have you been [working/ unemployed]?

MONTH: {EP129_PeriodToMonth}

@bYEAR@b:

IWER:
To year
PERIOD TO YEAR
1. 2005 or earlier

2. 2006
3. 2007
4. 2008
5. 2009
6. 2010
7. 2011
8. 2012
9. 2013
10. 2014
11. 2015

*ENDIF*Txt_FL_EP133

EP133_PeriodOtherEp
Were there other times since {FLLastInterviewMonthYear} when you have been [working for pay/ unemployed]?
OTHER PERIODS
1. Yes
5. No

ENDBLOCK
LOOP cnt:= 22 TO 40

IF PeriodOtherEpisodes[cnt - 1].EP133_PeriodOtherEp = a1

BLOCK
Txt_FL_EP127

EP127_PeriodFromMonth
From what @bmonth@b and year have you been [working/ unemployed]?

@bMONTH@b:
YEAR:
PERIOD FROM MONTH
1. January
2. February
3. March
4. April
5. May
6. June
7. July
8. August
9. September
10. October
11. November
12. December

Txt_FL_EP128

EP128_PeriodFromYear
From what month and @byear@b have you been [working/ unemployed]?

MONTH {EP127_PeriodFromMonth}
@bYEAR@b
PERIOD FROM YEAR
1. 2005 or earlier
2. 2006
3. 2007
4. 2008
5. 2009
6. 2010
7. 2011

8. 2012
9. 2013
10. 2014
11. 2015

Txt_FL_EP129

EP129_PeriodToMonth
To what @bmonth@b and year have you been [working/ unemployed]?

@bMONTH@b:
YEAR:

IWER:
If spell still ongoing type 13. Today
PERIOD TO MONTH
1. January
2. February
3. March
4. April
5. May
6. June
7. July
8. August
9. September
10. October
11. November
12. December
13. Today

IF EP129_PeriodToMonth <> a13
Txt_FL_EP130

EP130_PeriodToYear
To what month and @byear@b have you been [working/ unemployed]?

MONTH: {EP129_PeriodToMonth}

@bYEAR@b:

IWER:
To year
PERIOD TO YEAR
1. 2005 or earlier
2. 2006
3. 2007
4. 2008
5. 2009
6. 2010
7. 2011
8. 2012
9. 2013
10. 2014
11. 2015

*ENDIF*Txt_FL_EP133

EP133_PeriodOtherEp
Were there other times since {FLLastInterviewMonthYear} when you have been [working for pay/ unemployed]?
OTHER PERIODS

1. Yes
5. No

ENDBLOCK

ENDIF

ENDLOOP

*ENDIF*Txt_FL_EP326

EP326_ReceivedSeverancePayment
Since our last interview in {FLLastInterviewMonthYear}, have you received any severance payment?
RECEIVED SEVERANCE PAYMENT
1. Yes
5. No

IF EP326_ReceivedSeverancePayment = a1
Txt_FL_EP123

EP123_ReceiveSeveranceYear
In what YEAR did you receive the severance pay?

IWER:
If more than one code most recent
RECEIVE SEVERANCE YEAR
1. 2005 or earlier
2. 2006
3. 2007
4. 2008
5. 2009
6. 2010
7. 2011
8. 2012
9. 2013
10. 2014
11. 2015

ENDIF

ENDIF
IF ((EP005_CurrentJobSit = a2) OR ((MN101_Longitudinal = 0) AND (EP002_PaidWork = a1))) OR ((MN101_Longitudinal = 1) AND (EP335_Today = a1))
Txt_FL_EP008

EP008_Intro1
The following questions are about your current main job.

IWER:
Including seasonal job. The main job is the job the respondent is working most hours for. If same hours then choose the one the respondent gets more money from.
INTRODUCTION CURRENT JOB
1. Continue

Txt_FL_EP009

EP009_EmployeeOrSelf
In this job were you a private-sector employee, a public sector employee or self-employed?
EMPLOYEE OR SELF-EMPLOYED
1. Private sector employee
2. Public sector employee

3. Self-employed

IF ((MN101_Longitudinal = 0) OR NOT (a96 IN EP141_ChangeInJob)) OR (EP125_ContWork = a5)

Txt_FL_EP010

EP010_CurJobYear
In which year did you start this job?
START OF CURRENT JOB (YEAR)
1940..2015

IF EP010_CurJobYear = RESPONSE

CHECK NOT (((YEAR (SYSDATE) - EP010_CurJobYear) + 10) > MN808_AgeRespondent) L1 " [Year should be at least 10 years after year of birth. If year is correct, please press "suppress" and enter a remark to explain]"

*ENDIF*Txt_FL_EP616

EP616_NTofJob
What is this job called? Please give the exact name or title.
NAME OR TITLE OF JOB
STRING

IF EP616_NTofJob = RESPONSE

BLOCK

JobCode

STRING

ENDBLOCK

*ENDIF*Txt_FL_EP018

EP018_WhichIndustry
Please look at card 22. What kind of business, industry or services do you work in?
WHICH INDUSTRY ACTIVE
1. Agriculture, hunting, forestry, fishing
2. Mining and quarrying
3. Manufacturing
4. Electricity, gas and water supply
5. Construction
6. Wholesale and retail trade; repair of motor vehicles, motorcycles and personal and household goods
7. Hotels and restaurants
8. Transport, storage and communication
9. Financial intermediation
10. Real estate, renting and business activities
11. Public administration and defence; compulsory social security
12. Education
13. Health and social work
14. Other community, social and personal service activities

IF EP009_EmployeeOrSelf.ORD = 3

Txt_FL_EP024

EP024_NrOfEmployees
How many employees, if any, do you have in this job?

IWER:
Excluding respondent; only count people who work for or under the supervision of the respondent
{ReadOut}

NUMBER OF EMPLOYEES
0. None
1. 1 to 5
2. 6 to 15
3. 16 to 24
4. 25 to 199
5. 200 to 499
6. 500 or more

ENDIF
IF (EP009_EmployeeOrSelf = a1) OR (EP009_EmployeeOrSelf.ORD = 2)
Txt_FL_EP011

EP011_TermJob
In this job, do you have a short-term or a permanent contract?

IWER:
By short-term we mean less than 3 years
TERM OF JOB
1. Short-term
2. Permanent

ENDIF

*ENDIF*Txt_FL_EP013

EP013_TotWorkedHours
Including any paid or unpaid overtime, but not counting meal breaks, how many hours a week do you usually work in this job?

IWER:
This refers to the 'usual' working week. A seasonal worker working 40 hours a week for three months a year, should answer 40.
TOTAL HOURS WORKED PER WEEK
0..168
CHECK EP013_TotWorkedHours < 71 L1 " [Please check, number of hours seems to high]"

ENDIF
IF (MN101_Longitudinal = 1) AND (EP005_CurrentJobSit = a2)
Txt_FL_EP025

EP025_Intro
Please look at card 23.
Regarding your present job we would like to know whether you strongly agree, agree, disagree or strongly disagree with the following statements.
INTRODUCTION WORK SATISFACTION
1. Continue

Txt_FL_EP026

EP026_SatJob
All things considered I am satisfied with my job. Would you say you strongly agree, agree, disagree or strongly disagree?

IWER:
Show card 23
SATISFIED WITH JOB
1. Strongly agree
2. Agree
3. Disagree
4. Strongly disagree

Txt_FL_EP027

EP027_JobPhDem
My job is physically demanding. Would you say you strongly agree, agree, disagree or strongly disagree?

IWER:
Show card 23
JOB PHYSICALLY DEMANDING
1. Strongly agree
2. Agree
3. Disagree
4. Strongly disagree

Txt_FL_EP028

EP028_TimePress
I am under constant time pressure due to a heavy workload. (Would you say you strongly agree, agree, disagree or strongly disagree?)

IWER:
Show card 23
TIME PRESSURE DUE TO A HEAVY WORKLOAD
1. Strongly agree
2. Agree
3. Disagree
4. Strongly disagree

Txt_FL_EP029

EP029_LitFreeWork
I have very little freedom to decide how I do my work. (Would you say you strongly agree, agree, disagree or strongly disagree?)

IWER:
Show card 23
LITTLE FREEDOM TO DECIDE HOW I DO MY WORK
1. Strongly agree
2. Agree
3. Disagree
4. Strongly disagree

Txt_FL_EP030

EP030_NewSkill
I have an opportunity to develop new skills. (Would you say you strongly agree, agree, disagree or strongly disagree?)

IWER:
Show card 23
I HAVE AN OPPORTUNITY TO DEVELOP NEW SKILLS
1. Strongly agree
2. Agree
3. Disagree
4. Strongly disagree

Txt_FL_EP031

EP031_SuppDiffSit
I receive adequate support in difficult situations. (Would you say you strongly agree, agree, disagree or strongly disagree?)

IWER:
Show card 23
SUPPORT IN DIFFICULT SITUATIONS

1. Strongly agree
2. Agree
3. Disagree
4. Strongly disagree

Txt_FL_EP032

EP032_RecognWork
I receive the recognition I deserve for my work. (Would you say you strongly agree, agree, disagree or strongly disagree?)

IWER:
Show card 23
RECEIVE THE RECOGNITION DESERVING FOR MY WORK
1. Strongly agree
2. Agree
3. Disagree
4. Strongly disagree

Txt_FL_EP033

EP033_SalAdequate
Considering all my efforts and achievements, my [salary is/ earnings are/ salary is] adequate. (Would you say you strongly agree, agree, disagree or strongly disagree?)

IWER:
Show card 23. In case of doubt explain: we mean adequate for the work done.
SALARY OR EARNINGS ARE ADEQUATE
1. Strongly agree
2. Agree
3. Disagree
4. Strongly disagree

Txt_FL_EP034

EP034_JobPromPoor
My [job promotion prospects/ prospects for job advancement/ job promotion prospects] are poor. (Would you say you strongly agree, agree, disagree or strongly disagree?)

IWER:
Show card 23
PROSPECTS FOR JOB ADVANCEMENT ARE POOR
1. Strongly agree
2. Agree
3. Disagree
4. Strongly disagree

Txt_FL_EP035

EP035_JobSecPoor
My job security is poor. (Would you say you strongly agree, agree, disagree or strongly disagree?)

IWER:
Show card 23
JOB SECURITY IS POOR
1. Strongly agree
2. Agree
3. Disagree
4. Strongly disagree

ENDIF
IF ((EP005_CurrentJobSit = a2) OR ((MN101_Longitudinal = 0) AND (EP002_PaidWork = a1))) OR ((MN101_Longitudinal = 1) AND (EP335_Today = a1))

IF [EP005_CurrentJobSit](#) = a2

Txt_FL_EP036

EP036_LookForRetirement

[(' Now we will not use card ' + piSHOWCARD_ID) + ' any longer.'] Thinking about your present job, would you like to retire as early as you can from this job?

LOOK FOR EARLY RETIREMENT

1. Yes
5. No

Txt_FL_EP037

EP037_AfraidHRet

Are you afraid that your health will limit your ability to work in this job before regular retirement?

AFRAID HEALTH LIMITS ABILITY TO WORK BEFORE REGULAR RETIREMENT

1. Yes
5. No

Txt_FL_EP007

EP007_MoreThanOneJob

So far we have talked about your main job. Do you currently have a second job besides your main job?

IWER:

Please consider only paid jobs

CURRENTLY MORE THAN ONE JOB

1. Yes
5. No

IF [EP007_MoreThanOneJob](#) = a1

Txt_FL_EP321

EP321_TotWorkedHrsSecJob

Including any paid or unpaid overtime, but not counting meal breaks, how many hours a week do you usually work in this job?

IWER:

This refers to the 'usual' working week. A seasonal worker working 40 hours a week for three months a year, should answer 40.

TOTAL HOURS WORKED PER WEEK SECOND JOB

0..168

Txt_FL_EP322

EP322_NumMPerYearSecJob

How many months a year are you normally working in this job (including paid holidays)?

MONTHS WORKED IN SECOND JOB (NUMBER)

1..12

ENDIF

ENDIF

ENDIF

IF (MN101_Longitudinal = 0) AND ((([EP006_EverWorked](#) = a1) OR ([EP005_CurrentJobSit](#) = a1)) OR ([EP005_CurrentJobSit](#) = a3))

Txt_FL_EP048

EP048_IntroPastJob

We are now going to talk about the last job you had [before you retired/ before you became unemployed].

INTRODUCTION PAST JOB

1. Continue

Txt_FL_EP050

EP050_YrLastJobEnd
In which year did your last job end?
YEAR LAST JOB END
1900..2015

IF EP050_YrLastJobEnd = RESPONSE
CHECK ((YEAR (SYSDATE) - EP050_YrLastJobEnd) + 10) < MN808_AgeRespondent L1 " [Year should be at least 10 years after year of birth. If year is correct, please press "suppress" and enter a remark to explain]"

*ENDIF*Txt_FL_EP649

EP649_YrsInLastJob
How many years did you work in your last job?

IWER:
If more than one job, the question should refer to the one considered as main job by respondent. Include periods of unpaid leave. 1 for 6 months or more, 0 for less than 6 months.
YEARS WORKING IN LAST JOB
0..99

IF EP649_YrsInLastJob = RESPONSE
CHECK EP649_YrsInLastJob < MN808_AgeRespondent L1 " [Number should be less than or equal to respondent""s age. If age is correct, please press "suppress" and enter a remark to explain]"

*ENDIF*Txt_FL_EP051

EP051_EmployeeORSelf
In this job were you a private sector employee, a public sector employee or self-employed?
EMPLOYEE OR A SELF EMPLOYED IN LAST JOB
1. Private sector employee
2. Public sector employee
3. Self-employed

Txt_FL_EP152

EP152_NTofJob
What was this job called? Please give the exact name or title.
NAME OR TITLE OF JOB
STRING

IF EP152_NTofJob = RESPONSE

BLOCK

JobCode

STRING

ENDBLOCK

*ENDIF*Txt_FL_EP054

EP054_WhichIndustry
Please look at card 22. What kind of business, industry or services did you work in?

IWER:
Code answers 1..14.
WHICH INDUSTRY ACTIVE
1. Agriculture, hunting, forestry, fishing
2. Mining and quarrying
3. Manufacturing

4. Electricity, gas and water supply
5. Construction
6. Wholesale and retail trade; repair of motor vehicles, motorcycles and personal and household goods
7. Hotels and restaurants
8. Transport, storage and communication
9. Financial intermediation
10. Real estate, renting and business activities
11. Public administration and defence; compulsory social security
12. Education
13. Health and social work
14. Other community, social and personal service activities

IF EP051_EmployeeORSelf.ORD = 3

Txt_FL_EP061

EP061_NrOfEmployees

How many employees, if any, did you have?

IWER:
Read answers out
NUMBER OF EMPLOYEES

0. None
1. 1 to 5
2. 6 to 15
3. 16 to 24
4. 25 to 199
5. 200 to 499
6. 500 or more

ENDIF

ENDIF

*ENDIF*Txt_FL_EP203

EP203_IntroEarnings

We would now like to know more about your earnings and income during the last year, that is in [STR (Year - 1)].
INTRO INDIVIDUAL INCOME

1. Continue

Txt_FL_EP204

EP204_AnyEarnEmpl

Have you had any wages, salaries or other earnings from dependent employment in [STR (Year - 1)]?
ANY EARNINGS FROM EMPLOYMENT LAST YEAR

1. Yes
5. No

IF EP204_AnyEarnEmpl = a1

Txt_FL_EP205

EP205_EarningsEmplAT

After any taxes and contributions, what was your approximate annual income from employment in the year [STR (Year - 1)]? Please include any additional or extra or lump sum payment, such as bonuses, 13 month, Christmas or Summer pays.

IWER:
Amount in [FLDefault[9]]
EARNINGS EMPLOYMENT PER YEAR AFTER TAXES

-1000000000000000000..1000000000000000000

CHECK NOT ((ROUND (EP205_EarningsEmplAT) = 0) AND EP205_EarningsEmplAT <> NONRESPONSE) L1 " [Amount is expected to be lower or higher than zero]"
CHECK EP205_EarningsEmplAT <> EMPTY L1 " [Please enter a value]"

IF EP205_EarningsEmplAT = NONRESPONSE
UB SEQUENCE UB_EP905

ENDIF

*ENDIF*Txt_FL_EP206

EP206_AnyIncSelfEmpl
Have you had any income at all from self-employment or work for a family business in [STR (Year - 1)]?
INCOME FROM SELF-EMPLOYMENT LAST YEAR
1. Yes
5. No

IF EP206_AnyIncSelfEmpl = a1
Txt_FL_EP207

EP207_EarningsSelfAT
After any taxes and contributions and after paying for any materials, equipment or goods that you use in your work, what was your approximate annual income from self-employment in the year [STR (Year - 1)]?

IWER:
Amount in [FLDefault[9]]
EARNINGS PER YEAR AFTER TAXES FROM SELF-EMPLOYMENT
-100000000000000000..1000000000000000000
CHECK EP207_EarningsSelfAT <> EMPTY L1 " [Please enter a value]"

IF EP207_EarningsSelfAT = NONRESPONSE
UB SEQUENCE UB_EP907

ENDIF

*ENDIF*Txt_FL_EP303

EP303_Intro
Now we are going to ask you a set of questions regarding income from different public pensions and benefits. We are interested in the amounts, timing of these payments, and finally for how long you have received them.
INTRODUCTION INCOME FROM PUBLIC PENSIONS
1. Continue

Txt_FL_EP671

EP671_IncomeSources
Please look at card 24.
Have you received income from any of these sources in the year [STR (Year - 1)]?

IWER:
Main public sickness benefits: they are contribution-based payments received as an earnings replacement when an employee is off sick.

Main public disability insurance pension: if the sickness turns out to be long-standing, and a return to work is not to be expected, then the claimant will typically be transferred to a disability insurance pension (e.g. invalidity or incapacity benefit). The term 'pension' in the heading of this category is to be meant as 'regular payment', rather than relating to old age.

Public unemployment benefit or insurance: they are received, for a limited time period, by previous employees, later finding themselves unemployed. Eligibility is based on a history of insurance contribution.

Public long-term care insurance: it includes cash payments meant to provide for long term care needs; receipt does not necessarily depend on having previously paid contributions.

Social assistance: it includes cash or voucher programmes meant to provide a general 'safety net', guaranteeing minimum resources to those otherwise lacking resources from either employment or contributory based social security benefits/pensions.

{CodeAll}
INCOME FROM PUBLIC PENSIONS IN LAST YEAR
1. Public old age pension
2. Public old age supplementary pension or public old age second pension
3. Public early retirement or pre-retirement pension
4. Main public sickness benefits
5. Main public disability insurance pension
6. Secondary public disability insurance pension
7. Secondary public sickness benefits
8. Public unemployment benefit or insurance
9. Main public survivor pension from your spouse or partner
10. Secondary public survivor pension from your spouse or partner
11. Public war pension
12. Public long-term care insurance
13. Social assistance
96. None of these

CHECK NOT ((EP671_IncomeSources.CARDINAL > 1) AND (96 IN EP671_IncomeSources)) L1 " [You cannot select ""None of the above"" together with any other answer. Please change your answer]"

LOOP cnt:= 1 TO 13

IF cnt IN EP671_IncomeSources

BLOCK
Txt_FL_EP078

EP078_AvPaymPens
After taxes, about how large was a typical payment of [your public old age pension/ your public old age supplementary pension or public old age second pension/ your public early retirement or pre-retirement pension/ your main public sickness benefits/ your main public disability insurance pension/ your secondary public disability insurance pension/ your Secondary public sickness benefits"/ your public unemployment benefit or insurance/ your main public survivor pension from your spouse or partner/ your secondary public survivor pension from your spouse or partner/ your public war pension/ your public long-term care insurance/ your social assistance] in [STR (Year - 1)]?

IWER:
Amount in [FLDefault[9]]
It is an ordinary typical-regular payment, excluding any extras, such as bonuses, 13th month, lump-sum payments etc.
The time period will be asked in the next question: it could be monthly, quarterly or weekly, for example. The R should tell what the typical payment was for such a period during the year indicated in the question.
TYPICAL PAYMENT OF PENSIONS
-1000000000000000000..1000000000000000000
CHECK NOT ((EP078_AvPaymPens = 0) AND (EP078_AvPaymPens = RESPONSE)) L1 " [Amount is expected to be lower or higher than zero]"

IF EP078_AvPaymPens = NONRESPONSE
UB SEQUENCE UB_EP978

ENDIF

EP074_PeriodBenefit
What period did that payment cover?

IWER:
Do not include lump-sum payments. This will be asked later.
PERIOD OF INCOME SOURCE
1. One week

2. Two weeks
3. Calendar month/4 weeks
4. Three months/13 weeks
5. Six months/26 weeks
6. Full year/12 months/52 weeks
97. Other (specify)

IF EP074_PeriodBenefit = a97

EP075_OthPeriodBenefits

IWER:
Note other period
OTHER PERIOD OF RECEIVING BENEFITS
STRING

*ENDIF*Txt_FL_EP208

EP208_MonthsRecIncSource
For how many months altogether did you receive [the public old age pension/ the public old age supplementary pension or public old age second pension/ the public early retirement or pre-retirement pension/ the main public sickness benefits/ the main public disability insurance pension/ the secondary public disability insurance pension/ the secondary public sickness benefits/ the public unemployment benefit or insurance/ the main public survivor pension from your spouse or partner/ the secondary public survivor pension from your spouse or partner/ the public war pension/ the public long-term care insurance/ the social assistance] in [STR (Year - 1)]?

IWER:
Not how many payments were made, but the time-span. Example: the pension was received throughout the whole year, the answer is 12. In case the respondent started receiving it in November, the answer is 2.
HOW MANY MONTHS RECEIVED INCOME SOURCE
1..12

IF MN101_Longitudinal = 1
Txt_FL_EP612

EP612_WhenSource_long
Did you first receive this [the public old age pension/ the public old age supplementary pension or public old age second pension/ the public early retirement or pre-retirement pension/ the main public sickness benefits/ the main public disability insurance pension/ the secondary public disability insurance pension/ the secondary public sickness benefits/ the public unemployment benefit or insurance/ the main public survivor pension from your spouse or partner/ the secondary public survivor pension from your spouse or partner/ the public war pension/ the public long-term care insurance/ the social assistance] before our last interview in {FLLastInterviewMonthYear}?
BENEFIT BEFORE LAST INTERVIEW
1. Yes, before last interview
5. No, after last interview

ENDIF
IF NOT (MN101_Longitudinal = 1) OR (EP612_WhenSource_long = a5)
Txt_FL_EP213

EP213_YearRecIncSource
In which year did you first receive your [public old age pension/ public old age supplementary pension or public old age second pension/ public early retirement or pre-retirement pension/ main public sickness benefits/ main public disability insurance pension/ secondary public disability insurance pension/ secondary public sickness benefits/ public unemployment benefit or insurance/ main public survivor pension from your spouse or partner/ secondary public survivor pension from your spouse or partner/ public war pension/ public long-term care insurance/ social assistance]?

IWER:

In case of benefit received discontinuously during life (e.g., unemployment benefits received for different unemployment episodes), refer to the first payment of current stream of benefit, NOT to the first in life.
YEAR RECEIVED INCOME SOURCE
1930..2015

IF EP213_YearRecIncSource = RESPONSE
CHECK (CURRENTDATE.YEAR - EP213_YearRecIncSource) < MN808_AgeRespondent L1 " [Year should be greater than or equal to birthyear. If year is correct, please press "suppress" and enter a remark to explain]"

ENDIF

*ENDIF*Txt_FL_EP081

EP081_LumpSumPenState
Did you receive any additional, or extra or lump sum (one off) payment from [your public old age pension/ your public old age supplementary pension or public old age second pension/ your public early retirement or pre-retirement pension/ your main public sickness benefits/ your main public disability insurance pension/ your secondary public disability insurance pension/ your secondary public sickness benefits/ your public unemployment benefit or insurance/ your main public survivor pension from your spouse or partner/ your secondary public survivor pension from your spouse or partner/ your public war pension/ your public long-term care insurance/ your social assistance] during the year [STR (Year - 1)]?

IWER:
Please make sure that R takes into account all additional/extra/lump-sum payments received (including Christmas and Summer pays, if any) to answer this question.
LUMP SUM PAYMENT INCOME SOURCE
1. Yes
5. No

IF EP081_LumpSumPenState = a1
Txt_FL_EP082

EP082_TotAmountLS
After taxes, about how much did you receive overall as additional or extra payments in [STR (Year - 1)] from [this public old age pension/ this public old age supplementary pension or public old age second pension/ this public early retirement or pre-retirement pension/ this main public sickness benefits/ this main public disability insurance pension/ this secondary public disability insurance pension/ this secondary public sickness benefits/ this public unemployment benefit or insurance/ this main public survivor pension from your spouse or partner/ this secondary public survivor pension from your spouse or partner/ this public war pension/ this public long-term care insurance/ this social assistance]?

IWER:
Amount in [FLDefault[9]]
Include all additional or extra payments
TOTAL AMOUNT OF LUMP SUM PAYMENT FROM INCOME SOURCE
-1000000000000000000..10000000000000000000

IF EP082_TotAmountLS = NONRESPONSE
UB SEQUENCE UB_EP982

ENDIF

ENDIF

ENDBLOCK

ENDIF

*ENDLOOP*Txt_FL_EP624

EP624_OccPensInc
In addition to public pension benefits, pensions can also be provided through your employer. Have you

received income from any occupational pension in the year [STR (Year - 1)]?

IWER:
Occupational old age pension from your last or former jobs, from an early retirement pension, from disability or invalidity insurance or survivor pension from your spouse or partner's job.
HAD OCCUPATIONAL PENSION INCOME SOURCES
1. Yes
5. No

IF EP624_OccPensInc = a1

BLOCK
Txt_FL_EP678

EP678_AvPaymPens
After taxes, what was the approximate annual amount received from all your occupational pensions in [STR (Year - 1)]?

IWER:
Amount in [FLDefault[9]] Please exclude additional or extra, or lump-sum (one off) payments, such as bonuses, 13th month, Christmas and Summer pays.
APPROXIMATE ANUAL PAYMENT OF PENSIONS
-100000000000000000..1000000000000000000

IF MN101_Longitudinal = 1
Txt_FL_EP621

EP621_WhenSource_long
Did you start collecting your first occupational pension before our last interview in {FLLastInterviewMonthYear}?

IWER:
The first occupational pension is the first occupational pension the R has started collecting.
BENEFIT BEFORE LAST INTERVIEW
1. Yes, before last interview
5. No, after last interview

ENDIF
IF NOT (MN101_Longitudinal = 1) OR (EP621_WhenSource_long = a5)
Txt_FL_EP613

EP613_YearRecIncSource
In which year did you start collecting your first occupational pension?

IWER:
The first occupational pension is the first occupational pension the R has started collecting.
YEAR RECEIVED INCOME SOURCE
1930..2015

IF EP613_YearRecIncSource = RESPONSE
CHECK (CURRENTDATE.YEAR - EP613_YearRecIncSource) < MN808_AgeRespondent L1 " [Year should be greater than or equal to birthyear. If year is correct, please press "suppress" and enter a remark to explain]"

ENDIF

*ENDIF*Txt_FL_EP681

EP681_LumpSumPenState
Did you receive any additional, or extra or lump-sum (one off) payment from any of your occupational pensions during the year [STR (Year - 1)]?

IWER:
Please make sure that R takes into account all additional or extra or lump-sum (one off) payments received from any occupational pension (including bonuses, 13th month, Christmas and Summer pays, if any) to answer this question.
LUMP SUM PAYMENT INCOME SOURCE
1. Yes
5. No

IF EP681_LumpSumPenState = a1

Txt_FL_EP682

EP682_TotAmountLS
After taxes, about how much did you receive overall as additional or extra or lump-sum (one off) payments in [STR (Year - 1)] from your occupational pensions?

IWER:
Amount in [FLDefault[9]]
Include all additional or extra or lump-sum (one off) payments
TOTAL AMOUNT OF LUMP SUM PAYMENT FROM INCOME SOURCE
-1000000000000000000..1000000000000000000

CHECK NOT (EP682_TotAmountLS <= 0) L1 " [Amount is expected to be higher than zero]"

IF EP682_TotAmountLS = NONRESPONSE
UB SEQUENCE UB_EP982

ENDIF

ENDIF

ENDBLOCK

*ENDIF*Txt_FL_EP089

EP089_AnyRegPay
Please look at card 25. Did you receive any of the following regular payments or transfers during the year [STR (Year - 1)]?

IWER:
{CodeAll}
ANY OTHER REGULAR PAYMENTS RECEIVED
1. Life insurance payments from a private insurance company
2. Regular private annuity or private personal pension payments
3. Alimony
4. Regular payments from charities
5. Long-term care insurance payments from a private insurance company
96. None of these

CHECK NOT ((EP089_AnyRegPay.CARDINAL > 1) AND (96 IN EP089_AnyRegPay)) L1 " [You cannot select ""None of the above"" together with any other answer. Please change your answer]"

LOOP cnt:= 1 TO 5

IF cnt IN EP089_AnyRegPay

BLOCK

Txt_FL_EP094

EP094_TotalAmountBenLP
After any taxes and contributions, about how large was the average payment of [you life insurance payments from a private insurance company/ your private annuity or private personal pension payments/ your alimony/ your regular payments from charities/ your long-term care insurance payments] in [STR (Year - 1)]?

IWER:
Amount in [FLDefault[9]]
Do not include lump-sum payments. This will be asked later.
TOTAL AMOUNT IN THE LAST PAYMENT
-1000000000000000000..1000000000000000000
CHECK NOT ((EP094_TotalAmountBenLP <= 0) AND (EP094_TotalAmountBenLP = RESPONSE)) L1 "[Amount is expected to be higher than zero]"

IF EP094_TotalAmountBenLP = NONRESPONSE
UB SEQUENCE UB_EP994

ENDIF

EP090_PeriodPaym
Which period did that payment cover?
PERIOD RECEIVED REGULAR PAYMENTS
1. One week
2. Two weeks
3. Calendar month/4 weeks
4. Three months/13 weeks
5. Six months/26 weeks
6. Full year/12 months/52 weeks
97. Other (specify)

IF EP090_PeriodPaym = a97

EP091_OthPeriodPaym

IWER:
Specify other
OTHER PERIOD OF RECEIVING REGULAR PAYMENTS
STRING

*ENDIF*Txt_FL_EP096

EP096_MonthsRegPaym
For how many months altogether did you receive [life insurance payments/ private annuity or private personal pension payments/ alimony/ regular payments from charities/ long-term care insurance payments] in [STR (Year - 1)]?
MONTHS RECEIVED REGULAR PAYMENTS
1..12
Txt_FL_EP092

EP092_AddPayments
For [your life insurance payments/ your private annuity or private personal pension payments/ your alimony/ your regular payments from charities/ your long-term care insurance payments], did you get additional or lump sum payments in [STR (Year - 1)]?
ADDITIONAL PAYMENTS FOR THIS BENEFIT IN LAST YEAR
1. Yes
5. No

IF EP092_AddPayments = a1
Txt_FL_EP209

EP209_AddPaymAT
After any taxes and contributions, about how much did you get in additional payments?

IWER:
Amount in [FLDefault[9]]
ADDITIONAL PAYMENTS AFTER TAXES
-1000000000000000000..1000000000000000000

CHECK NOT ((EP209_AddPaymAT <= 0) AND (EP209_AddPaymAT = RESPONSE)) L1 " [Amount is expected to be higher than zero]"

IF EP209_AddPaymAT = NONRESPONSE

UB SEQUENCE UB_EP909

ENDIF

ENDIF

ENDBLOCK

ENDIF

ENDLOOP

IF (MN024_NursingHome = a1) AND (MN808_AgeRespondent < 76)

Txt_FL_EP097

EP097_PensClaim

Now we are talking about pensions you might receive in the future but you do not receive currently. Will you be entitled to at least one pension listed on card 26 which you do not receive currently?

PENSION CLAIMS

1. Yes
5. No

IF EP097_PensClaim = a1

Txt_FL_EP098

EP098_TypeOfPension

Which type or types of pension will you be entitled to?

IWER:

{CodeAll}

Respondent must not receive these pensions already

TYPE OF PENSION YOU WILL BE ENTITLED TO

1. Public old age pension
2. Public early retirement or pre-retirement pension
3. Public disability insurance; sickness/invalidity/incapacity pension
4. Private (occupational) old age pension
5. Private (occupational) early retirement pension

LOOP cnt:= 1 TO 5

IF cnt IN EP098_TypeOfPension

BLOCK

Txt_FL_EP102

EP102_CompVolun

Is participation in [this public old age pension/ this public early retirement or pre-retirement pension/ this public disability insurance; sickness/invalidity/incapacity pension/ this private (occupational) old age pension/ this private (occupational) early retirement pension] compulsory or voluntary?

COMPULSORY OF VOLUNTARY PLAN OR FUND

1. Compulsory
2. Voluntary

Txt_FL_EP103

EP103_YrsContrToPlan

How many years have you been contributing to [your public old age pension/ your public early retirement or pre-retirement pension/ your public disability insurance; sickness/invalidity/incapacity pension/ your private (occupational) old age pension/ your private (occupational) early retirement pension] ?

IWER:
Contribution by employer should be counted as well.
YEARS CONTRIBUTING TO PLAN
0..75
CHECK EP103_YrsContrToPlan <= MN808_AgeRespondent L1 " [Number should be less than or equal to respondent""s age. If age is correct, please press "suppress" and enter a remark to explain]"
Txt_FL_EP106

EP106_ExpRetAge
At what age do you yourself expect to start collecting this pension payment for the first time?
EXPECTED AGE TO COLLECT THIS PENSION
30..75
CHECK NOT ((EP106_ExpRetAge < MN808_AgeRespondent) AND (EP106_ExpRetAge = RESPONSE)) L1 " [Expected age should be higher than or equal to current age. If age is correct, please press "suppress" and enter a remark to explain]"

IF EP005_CurrentJobSit = a2
Txt_FL_EP609

EP609_PWExpPensStatAge
Please think about the time at which you will start collecting this pension. How much will be your first monthly benefit after taxes from [your public old age pension/ your public early retirement or pre-retirement pension/ your public disability insurance; sickness/invalidity/incapacity pension/ your private (occupational) old age pension/ your private (occupational) early retirement pension]?

IWER:
Amount in [FLDefault[9]]
EXPECTED AMOUNT OF PENSION BENEFIT
-1000000000000000000..1000000000000000000

ENDIF

ENDBLOCK

ENDIF

ENDLOOP

ENDIF

ENDIF

EP210_IntCheck

IWER:
Who answered the questions in this section?
WHO ANSWERED SECTION EP
1. Respondent only
2. Respondent and proxy
3. Proxy only

System preset value EP902_TimeStampEnd
IF EP902_TimeStampEnd = EMPTY AND EP210_IntCheck <> EMPTY
ENDIF

ENDBLOCK

ENDIF
IF (IT IN Test) OR (ALL IN Test)

BLOCK

Txt_FL_IT005

IT005_Continue
Now we are going to talk about computers
INTRO IT MODULE
1. Continue

System preset value IT901_TimeStampStart
IF IT901_TimeStampStart = EMPTY AND IT005_Continue <> EMPTY
ENDIF
IF Sec_EP.EP005_CurrentJobSit = a2
Txt_FL_IT001

IT001_PC_work
Does your current job require using a computer?

IWER:
Computer could be a PC (Personal Computer), or a tablet (I-Pad or the like)
CURRENT JOB REQUIRES COMPUTER
1. Yes
5. No

ELSE

IF Sec_EP.EP005_CurrentJobSit = a1
Txt_FL_IT002

IT002_PC_work
Did your last job before retiring require using a computer?

IWER:
Computer could be a PC (Personal Computer), or a tablet (I-Pad or the like)
LAST JOB REQUIRED COMPUTER
1. Yes
5. No

ENDIF

*ENDIF*Txt_FL_IT003

IT003_PC_skills
How would you rate your computer skills? Would you say they are...

IWER:
{ReadOut}
PC skills
1. Excellent
2. Very good
3. Good
4. Fair
5. Poor
6. I never used a computer (SPONTANEOUS ONLY)

Txt_FL_IT004

IT004_UseWWW
During the past 7 days, have you used the Internet, for e-mailing, searching for information, making purchases, or for any other purpose at least once?
USE WORLD WIDE WEB
1. Yes
5. No

System preset value IT902_TimeStampEnd
IF IT902_TimeStampEnd = EMPTY AND IT004_UseWWW <> EMPTY
ENDIF

ENDBLOCK

ENDIF
IF (GS IN Test) OR (ALL IN Test)

BLOCK
Txt_FL_GS001

GS001_Willingness
Now I would like to assess the strength of your hand in a gripping exercise. I will ask you to squeeze this handle as hard as you can, just for a couple of seconds and then let go. I will take two alternate measurements from your right and your left hand. Would you be willing to have your handgrip strength measured?

IWER:
Demonstrate grip strength measure
If R is unsure explain: Also weak people or people with dementia, arthrosis, rheumatism, Parkinson´s disease or a stroke can do the measure
Start of a @BNon-proxy section@B. No proxy allowed.
If the respondent is not doing test on her/his own please, select '5'.
WILLING TO HAVE HANDGRIP MEASURED
1. R agrees to take measurement
2. R refuses to take measurement
3. R is unable to take measurement
5. Proxy-interview

System preset value GS901_TimeStampStart
IF GS901_TimeStampStart = EMPTY AND GS001_Willingness <> EMPTY
ENDIF
IF (GS001_Willingness = RESPONSE) AND NOT (GS001_Willingness = a5)

IF GS001_Willingness <> a1

GS010_WhyNotCompl

IWER:
Why didn't R complete the grip strength test?
{CodeAll}
WHY NOT COMPLETED GS TEST
1. R felt it would not be safe
2. IWER felt it would not be safe
3. R refused, no reason given
4. R tried but was unable to complete test
5. R did not understand the instructions
6. R had surgery, injury, swelling, etc. on both hands in past 6 months
97. Other (Specify)

IF a97 IN GS010_WhyNotCompl

GS011_OthReason

IWER:
Specify other reason
OTHER REASON
STRING

ENDIF

ENDIF
IF GS001_Willingness = a1

GS002_RespStatus

IWER:
Record respondent status
RECORD RESPONDENT STATUS
1. Respondent has the use of both hands
2. Respondent is unable to use right hand
3. Respondent is unable to use left hand

ENDIF
IF GS001_Willingness <> a1

GS003_StopTest
INTERVIEWER STOP TEST.

IWER:
No handgrip measurement to be taken.
END OF TEST BECAUSE RESPONDENT IS UNABLE OR NOT WILLING TO DO TEST
1. Continue

ENDIF
IF GS001_Willingness = a1

IF GS002_RespStatus = a1
Txt_FL_GS004

GS004_DominantHand
Which is your dominant hand?

IWER:
Natural ambidexterity is the state of being born with equally adept in the use of both left and right hands, not adapted to.
DOMINANT HAND
1. Right hand
2. Left hand
3. Ambidexterity

ENDIF

GS005_IntroTest

IWER:
Position the respondent correctly. Adjust dynamometer to hand size by turning the lever and reset arrow at zero. Explain the procedure once again. Let respondent practice with one hand. Use scorecard to record the results and enter results into computer after test is finished.
INTRODUCTION TO TEST
1. Continue

IF (GS002_RespStatus = a1) OR (GS002_RespStatus = a2)

GS006_FirstLHand
LEFT HAND, FIRST MEASUREMENT.

IWER:
Enter the results to the nearest integer value.
FIRST MEASUREMENT, LEFT HAND
0..100

ENDIF
IF (GS002_RespStatus = a1) OR (GS002_RespStatus = a3)

GS008_FirstRHand
RIGHT HAND, FIRST MEASUREMENT.

IWER:
Enter the results to the nearest integer value.
FIRST MEASUREMENT, RIGHT HAND
0..100

ENDIF
IF (GS002_RespStatus = a1) OR (GS002_RespStatus = a2)

GS007_SecondLHand
LEFT HAND, SECOND MEASUREMENT.

IWER:
Enter the results to the nearest integer value.
SECOND MEASUREMENT, LEFT HAND
0..100

ENDIF
IF (GS002_RespStatus = a1) OR (GS002_RespStatus = a3)

GS009_SecondRHand
RIGHT HAND, SECOND MEASUREMENT.

IWER:
Enter the results to the nearest integer value.
SECOND MEASUREMENT, RIGHT HAND
0..100

*ENDIF*CHECK NOT ((GS007_SecondLHand <= (GS006_FirstLHand - 20)) OR (GS007_SecondLHand >= (GS006_FirstLHand + 20))) L1 " [The difference between the first and second measurement with the left hand is very large; Have you entered the correct numbers]"
CHECK NOT ((GS009_SecondRHand <= (GS008_FirstRHand - 20)) OR (GS009_SecondRHand >= (GS008_FirstRHand + 20))) L1 " [The difference between the first and second measurement with the right hand is very large; Have you entered the correct numbers]"

GS012_Effort

IWER:
How much effort did R give to this measurement?
HOW MUCH EFFORT R GAVE
1. R gave full effort
2. R was prevented from giving full effort by illness, pain, or other symptoms or discomforts
3. R did not appear to give full effort, but no obvious reason for this

GS013_Position

IWER:
What was the R's position for this test?
THE POSITION OF R FOR THIS TEST
1. Standing

2. Sitting
3. Lying down

GS014_RestArm

IWER:
Did R rest his/her arms on a support while performing this test?
R RESTED HIS/HER ARMS ON A SUPPORT
1. Yes
5. No

ENDIF
System preset value GS902_TimeStampEnd
IF GS902_TimeStampEnd = EMPTY AND ((GS003_StopTest <> EMPTY OR GS007_SecondLHand <> EMPTY) OR GS009_SecondRHand <> EMPTY)
ENDIF

ENDIF

ENDBLOCK

ENDIF
IF (BS IN Test) OR (ALL IN Test)

IF ((((MN028_bio = 1) OR (MN028_bio = 2)) OR (MN028_bio = 3)) OR (MN028_bio = 4)) OR (MN028_bio = 5)

BLOCK
Txt_FL_BS023

BS023_bsnonproxy

IWER:
Start of a @BNon-proxy section@B. No proxy allowed.
If the respondent is not present or not capable to give consent to participation on [his/ her] own, please select '5'.
NON PROXY MODULE
1. Continue
5. Proxy-interview

System preset value BS901_TimeStampStart
IF BS901_TimeStampStart = EMPTY AND BS023_bsnonproxy <> EMPTY
ENDIF
IF (BS023_bsnonproxy = RESPONSE) AND NOT (BS023_bsnonproxy = a5)

IF MN028_bio = 5
Txt_FL_BS026

BS026_nursevisit
Since your health is a very important element of this interview and of our research project we have arranged several medical procedures, such as measuring your blood pressure and taking samples of your blood which would be conducted by a qualified nurse on a separate visit. We would be very grateful if you agreed to participate in this additional part of the project. Would you be willing to take part in this part and agree to be visited by a qualified nurse in the next few weeks?
NURSE INTRO
1. Yes
5. No

IF BS026_nursevisit = a1

Txt_FL_BS027

BS027_nurseagreement
Thank you very much for your agreement. What is the best way the nurse could contact you to arrange her visit? Could you give us a telephone number she could call?

IWER:
Please note contact details. Hand out information leaflet to the respondent.
THANKS
1. Continue

ELSE

BS003_EndDBS
Thank you. We will continue with the next topic.
END DBS
1. Continue

ENDIF

ELSE

IF (MN028_bio = 1) OR (MN028_bio = 3)
Txt_FL_BS001

BS001_introduction
To assess the health status of the general population age 50 and above, we would like to collect a few drops of blood. This will be done using just a finger prick as it is done daily by millions of people with diabetes. We would be very grateful if you agreed to participate. Yet, this blood collection is absolutely voluntary.

Before we begin, I would like to have you read this information sheet.

IWER:
1. Take the "information leaflet" and the "Dried Blood Spots Collection" consent forms and hand them to the respondent.
2. Let respondent read the information leaflet. Allow sufficient time for reading.
INTRODUCTION
1. Continue

ELSE

IF (MN028_bio = 2) OR (MN028_bio = 4)
Txt_FL_BS025

BS025_alternativeintro
We now move on to the collection of blood samples we explained to you in our information letter. To assess the health status of the population, we would like to collect a few drops of blood. This will be done using just a finger prick as it is done daily by people with diabetes. We would be very grateful if you agreed to participate. Yet, this blood collection is absolutely voluntary.

Before I ask you to sign this consent form, do you want me to explain the procedure again?

IWER:
1. If respondent wants, please explain procedure and purpose of the dried blood spots collection.
2. Take the Dried Blood Spots consent forms and hand them to the respondent.
INTRODUCTION
1. Continue

ENDIF

*ENDIF*Txt_FL_BS006

BS006_MedicalReasons
From your point of view: are there any medical reasons which would prevent you from participating?
MEDICAL REASONS
1. Yes
5. No

IF BS006_MedicalReasons = a5
Txt_FL_BS002

BS002_consent
Do you have any further questions?

IWER:
1. If the respondent has any questions, please answer them
2. Let respondent sign the consent forms
3. Leave one copy of the consent form with the respondent
CONSENT
1. R signed the consent form
5. R did NOT sign the consent form

ENDIF
IF (((((BS006_MedicalReasons = a1) OR (BS006_MedicalReasons = REFUSAL)) OR (BS006_MedicalReasons = DONTKNOW)) OR (BS002_consent = a5)) OR (BS002_consent = REFUSAL)) OR (BS002_consent = DONTKNOW)

BS003_EndDBS
Thank you. We will continue with the next topic.
END DBS
1. Continue

ELSE

BS004_ForbiddenAnalyses

IWER:
Please copy the excluded analyses that the respondent specified on the consent form.
Type "none" if respondent did not write anything down.
FORBIDDEN ANALSES
STRING
Txt_FL_BS008

BS008_DBSSinstruction

IWER:
Please be aware: if respondent takes blood thinning medication, it may take longer time to stop the bleeding. In this case make the respondent elevate his/her hand and have gauze pads available.

Take DBS Interviewer Short Instructions and follow the instructions 1 to 15.
DBSS INSTRUCTION
1. Continue

BS009_BarcodeFirst

IWER:

Enter barcode number from barcode label into CAPI.
BARCODE
STRING[8]
CHECK LEN (BS009_BarcodeFirst) = 8 L1 " [Barcode must have 8 digits]"

BS010_BarcodeSecond

IWER:
Repeat barcode number
BARCODE REPEAT
STRING[8]
CHECK BS009_BarcodeFirst = BS010_BarcodeSecond L1 " [values should be equal]"

IF (MN028_bio = 3) OR (MN028_bio = 4)
Txt_FL_BS021

BS021_Feedback
Do you wish to be informed [OR country-specific: Do you wish to be informed via your general practitioner] about blood results if a value lies outside the normal range? Please be aware that a lot of time may pass until these results are available, and that this information does not replace consultation with a physician.

IWER:
If the respondent wants to be informed, take the "TRANSMISSION OF DBS ANALYSES RESULTS" consent form and hand it to the respondent. Let respondent read and sign the consent form. Allow sufficient time for reading.
FEEDBACK
1. Yes, R wants to be informed and signed the consent form.
5. No, R doesn't want to be informed/did not sign the consent form

*ENDIF*Txt_FL_BS011

BS011_Thanks
Thank you for your cooperation.
This completes the collection of dried blood spots.
Before we continue with the interview, I need a moment to enter a few pieces of information in the computer.
THANKS
1. Continue

BS012_Problems

IWER:
What, if any, problems occured during the collection of the blood sample? {CodeAll}
PROBLEMS
1. R became light-headed, fainted, or nauseous
2. R had difficulty getting finger to stop bleeding
3. Unable to obtain enough blood
4. Problem with equipment or supplies
96. None
97. Other

CHECK NOT ((BS012_Problems.CARDINAL > 1) AND (a96 IN BS012_Problems)) L1 " [You cannot select ""None of the above"" together with any other answer. Please change your answer]"

IF a97 IN BS012_Problems

BS022_OtherProblems

IWER:
Please specify.
OTHER PROBLEMS
STRING

ENDIF

BS013_WhoPricked

IWER:
Who pricked the respondent's finger(s)?
WHO PRICKED
1. R pricked own finger(s)
2. IWER pricked R's finger(s)
3. Both R and IWER pricked R's finger(s)
4. Someone else pricked R's finger(s)

BS014_HowManyPricks

IWER:
How many pricks have been made?
HOW MANY PRICKS
1. One prick
2. Two pricks

BS015_Circles

IWER:
How many circles on the card could be filled?
CIRCLES
0. No circles could be filled
1. 1
2. 2
3. 3
4. 4
5. 5

BS016_Compliance

IWER:
How compliant was the respondent during this measurement?/

1. R was fully compliant
2. R was prevented from fully complying due to illness, pain, or other symptoms or discomforts
3. R was not fully compliant, due to other reasons

ENDIF

ENDIF
System preset value BS902_TimeStampEnd
IF BS902_TimeStampEnd = EMPTY AND (BS003_EndDBS <> EMPTY OR BS016_Compliance <> EMPTY)
ENDIF

ENDIF

ENDBLOCK

ENDIF

ENDIF
IF (PF IN Test) OR (ALL IN Test)

BLOCK
Txt_FL_PF001

PF001_Intro
The next test that I am going to ask you to perform measures your lung function, - to be more precise, how fast you can expel air from your lungs.
Having a lung disease is not a hindrance for carrying out the test. It is important that you blow as hard and as fast as you can.

Like this...

IWER:
Demonstrate the test.
Start of a @BNon-proxy section@B. No proxy allowed.
If the respondent is not doing test on her/his own, please select '5'.
INTRODUCTION
1. Continue
5. Proxy-interview

System preset value PF901_TimeStampStart
IF PF901_TimeStampStart = EMPTY AND PF001_Intro <> EMPTY
ENDIF
IF (PF001_Intro = RESPONSE) AND NOT (PF001_Intro = a5)
Txt_FL_PF002

PF002_Safe
Would you be willing to do the test?

IWER:
If R is unsure explain: It is a common test carried out by those with asthma and other chronic lung diseases for self-monitoring purposes. Blowing hard may result in unharmful coughing.
SAFE TO DO THE TEST
1. Yes
5. No

IF PF002_Safe = a1

PF003_ValFirstMeas

IWER:
Enter value first measurement
(Record 30 if less than 60;
record 890 if past last tick mark;
record 993 if R tried but was unable;
or record 999 if R chose not to do it.)
VALUE FIRST MEASUREMENT
30..999

PF004_ValSecMeas

IWER:
Enter value second measurement
(Record 30 if less than 60;
record 890 if past last tick mark;

record 993 if R tried but was unable;
or record 999 if R chose not to do it.)
VALUE SECOND MEASUREMENT
30..999

IF (PF003_ValFirstMeas < 999) AND (PF004_ValSecMeas < 999)

PF005_EffortR

IWER:
How much effort did R give to this measurement?
EFFORT R GAVE TO THIS MEASUREMENT
1. R gave full effort
2. R was prevented from giving full effort by illness, pain, or other symptoms or discomforts
3. R did not appear to give full effort, but no obvious reason for this

PF006_PositionR

IWER:
What was the R's position for this test?
POSITION OF R FOR THIS TEST
1. Standing
2. Sitting
3. Lying down

ENDIF

ENDIF
IF (PF002_Safe = a5) OR ((PF003_ValFirstMeas > 890) OR (PF004_ValSecMeas > 890))

PF007_WhyNotCompl

IWER:
Why didn't R complete the breathing test?
{CodeAll}
WHY PF NOT COMPLETED
1. R felt it would not be safe
2. IWER felt it would not be safe
3. R refused or was not willing to complete the test
4. R tried but was unable to complete test
5. R did not understand the instructions
97. Other (Specify)

IF a97 IN PF007_WhyNotCompl

PF008_OthReason

IWER:
Record other reason
OTHER REASON NOT COMPLETED PF
STRING

ENDIF

ENDIF
System preset value PF902_TimeStampEnd
IF PF902_TimeStampEnd = EMPTY AND (PF006_PositionR <> EMPTY OR PF007_WhyNotCompl <> EMPTY)

ENDIF

ENDIF

ENDBLOCK

ENDIF
IF (SP IN Test) OR (ALL IN Test)

BLOCK
Txt_FL_SP001

SP001_Intro
The next questions are about the help that you may have given to people you know or that you may have received from people you know.
INTRODUCTION SP
1. Continue

System preset value SP901_TimeStampStart
IF SP901_TimeStampStart = EMPTY AND SP001_Intro <> EMPTY
*ENDIF*Txt_FL_SP002

SP002_HelpFrom
Please look at card 27. Thinking about the last twelve months, has any family member from outside the household, any friend or neighbour given you any kind of help listed on this card?
RECEIVED HELP FROM OTHERS
1. Yes
5. No

IF SP002_HelpFrom = a1

BLOCK
Txt_FL_SP003

SP003_FromWhoHelp
Which [other] family member from outside the household, friend or neighbour has helped you in the last twelve months?
WHO GAVE YOU HELP
1. Spouse/Partner
2. Mother
3. Father
4. Mother-in-law
5. Father-in-law
6. Stepmother
7. Stepfather
8. Brother
9. Sister
10. Child
11. Step-child/your current partner's child
12. Son-in-law
13. Daughter-in-law
14. Grandchild
15. Grandparent
16. Aunt
17. Uncle
18. Niece
19. Nephew
20. Other relative
21. Friend
22. (Ex-)colleague/co-worker
23. Neighbour
24. Ex-spouse/partner

25. Minister, priest, or other clergy
26. Therapist or other professional helper
27. Housekeeper/Home health care provider
96. None of these

IF (SP003_FromWhoHelp = a10) OR (SP003_FromWhoHelp = a11)

SP027_WhatChild
Which child?
WHAT CHILD GIVEN FINANCIAL GIFT
^FLChild[1]
^FLChild[2]
^FLChild[3]
^FLChild[4]
^FLChild[5]
^FLChild[6]
^FLChild[7]
^FLChild[8]
^FLChild[9]
^FLChild[10]
^FLChild[11]
^FLChild[12]
^FLChild[13]
^FLChild[14]
^FLChild[15]
^FLChild[16]
^FLChild[17]
^FLChild[18]
^FLChild[19]
^FLChild[20]
96. ^FLDefault[79]

IF SP027_WhatChild = a96

SP023_NameOthChild

IWER:
Record child´s name
NAME OTHER CHILD
STRING

ENDIF

ELSE

LOOP i:= 1 TO 7

IF Sec_SN.SN_Roster[i].SN005_NetworkRelationship = SP003_FromWhoHelp
ELSE
ENDIF

ENDLOOP
IF FoundAPotentialMatchingSNMember = 1
Txt_FL_SP028

SP028_WhatSNmember
Is this [{Relationship string is loaded}] you mentioned earlier?
WHAT SNMEMBER GIVEN FINANCIAL GIFT
^FLSNmember[1]
^FLSNmember[2]
^FLSNmember[3]

^FLSNmember[4]
^FLSNmember[5]
^FLSNmember[6]
^FLSNmember[7]
96. ^FLDefault[80]

ENDIF

*ENDIF*Txt_FL_SP004

SP004_TypesOfHelp
Please look at card 28. Which types of help has this person provided in the last twelve months?

IWER:
{CodeAll}
WHICH TYPES OF HELP
1. personal care, e.g. dressing, bathing or showering, eating, getting in or out of bed, using the toilet
2. practical household help, e.g. with home repairs, gardening, transportation, shopping, household chores
3. help with paperwork, such as filling out forms, settling financial or legal matters

Txt_FL_SP005

SP005_HowOftenHelpRec
In the last twelve months how often altogether have you received such help from this person? Was it...

IWER:
{ReadOut}
HOW OFTEN RECEIVED HELP FROM THIS PERSON
1. About daily
2. About every week
3. About every month
4. Less often

IF piIndex <> 3
Txt_FL_SP007

SP007_OtherHelper
(Please look at card 27) Is there any other family member from outside the household, friend or neighbour who has given you personal care or practical household help?
ANY OTHER HELPER FROM OUTSIDE THE HOUSEHOLD
1. Yes
5. No

ENDIF

ENDBLOCK
LOOP cnt1:= 2 TO 3

IF HelpFromOther[cnt1 - 1].SP007_OtherHelper = a1

BLOCK
Txt_FL_SP003

SP003_FromWhoHelp
Which [other] family member from outside the household, friend or neighbour has helped you in the last twelve months?
WHO GAVE YOU HELP
1. Spouse/Partner
2. Mother
3. Father
4. Mother-in-law

5. Father-in-law
6. Stepmother
7. Stepfather
8. Brother
9. Sister
10. Child
11. Step-child/your current partner's child
12. Son-in-law
13. Daughter-in-law
14. Grandchild
15. Grandparent
16. Aunt
17. Uncle
18. Niece
19. Nephew
20. Other relative
21. Friend
22. (Ex-)colleague/co-worker
23. Neighbour
24. Ex-spouse/partner
25. Minister, priest, or other clergy
26. Therapist or other professional helper
27. Housekeeper/Home health care provider
96. None of these

IF (SP003_FromWhoHelp = a10) OR (SP003_FromWhoHelp = a11)

SP027_WhatChild
Which child?
WHAT CHILD GIVEN FINANCIAL GIFT
^FLChild[1]
^FLChild[2]
^FLChild[3]
^FLChild[4]
^FLChild[5]
^FLChild[6]
^FLChild[7]
^FLChild[8]
^FLChild[9]
^FLChild[10]
^FLChild[11]
^FLChild[12]
^FLChild[13]
^FLChild[14]
^FLChild[15]
^FLChild[16]
^FLChild[17]
^FLChild[18]
^FLChild[19]
^FLChild[20]
96. ^FLDefault[79]

IF SP027_WhatChild = a96

SP023_NameOthChild

IWER:
Record child´s name
NAME OTHER CHILD
STRING

ENDIF

ELSE

LOOP i:= 1 TO 7

IF Sec_SN.SN_Roster[i].SN005_NetworkRelationship = SP003_FromWhoHelp
ELSE
ENDIF

ENDLOOP
IF FoundAPotentialMatchingSNMember = 1
Txt_FL_SP028

SP028_WhatSNmember
Is this [{Relationship string is loaded}] you mentioned earlier?
WHAT SNMEMBER GIVEN FINANCIAL GIFT
^FLSNmember[1]
^FLSNmember[2]
^FLSNmember[3]
^FLSNmember[4]
^FLSNmember[5]
^FLSNmember[6]
^FLSNmember[7]
96. ^FLDefault[80]

ENDIF

*ENDIF*Txt_FL_SP004

SP004_TypesOfHelp
Please look at card 28. Which types of help has this person provided in the last twelve months?

IWER:
{CodeAll}
WHICH TYPES OF HELP
1. personal care, e.g. dressing, bathing or showering, eating, getting in or out of bed, using the toilet
2. practical household help, e.g. with home repairs, gardening, transportation, shopping, household chores
3. help with paperwork, such as filling out forms, settling financial or legal matters

Txt_FL_SP005

SP005_HowOftenHelpRec
In the last twelve months how often altogether have you received such help from this person? Was it...

IWER:
{ReadOut}
HOW OFTEN RECEIVED HELP FROM THIS PERSON
1. About daily
2. About every week
3. About every month
4. Less often

IF piIndex <> 3
Txt_FL_SP007

SP007_OtherHelper
(Please look at card 27) Is there any other family member from outside the household, friend or neighbour who has given you personal care or practical household help?
ANY OTHER HELPER FROM OUTSIDE THE HOUSEHOLD
1. Yes
5. No

ENDIF

ENDBLOCK

ENDIF

ENDLOOP

*ENDIF*Txt_FL_SP008

SP008_GiveHelp
Now I would like to ask you about the help you have @bgiven@b to others.
Please look at card 27.
In the last twelve months, have you @bpersonally@b given any kind of help listed on this card to a family member from outside the household, a friend or neighbour?

IWER:
QUESTION DOES NOT INCLUDE LOOKING AFTER YOUR OWN GRANDCHILDREN; THIS IS ASKED LATER IN SP014
GIVEN HELP IN THE TIME SINCE THE LAST INTERVIEW
1. Yes
5. No

System preset value SP901_TimeStampStart
IF SP901_TimeStampStart = EMPTY AND SP008_GiveHelp <> EMPTY
ENDIF
IF SP008_GiveHelp = a1

BLOCK
Txt_FL_SP009

SP009_ToWhomGiveHelp
Which [other] family member from outside the household, friend or neighbour have you helped [most often] in the last twelve months?
TO WHOM DID YOU GIVE HELP
1. Spouse/Partner
2. Mother
3. Father
4. Mother-in-law
5. Father-in-law
6. Stepmother
7. Stepfather
8. Brother
9. Sister
10. Child
11. Step-child/your current partner's child
12. Son-in-law
13. Daughter-in-law
14. Grandchild
15. Grandparent
16. Aunt
17. Uncle
18. Niece
19. Nephew
20. Other relative
21. Friend
22. (Ex-)colleague/co-worker
23. Neighbour
24. Ex-spouse/partner
25. Minister, priest, or other clergy
26. Therapist or other professional helper

27. Housekeeper/Home health care provider
96. None of these

IF (SP009_ToWhomGiveHelp = a10) OR (SP009_ToWhomGiveHelp = a11)

SP029_WhatChild
Which child?
WHAT CHILD GIVEN FINANCIAL GIFT
^FLChild[1]
^FLChild[2]
^FLChild[3]
^FLChild[4]
^FLChild[5]
^FLChild[6]
^FLChild[7]
^FLChild[8]
^FLChild[9]
^FLChild[10]
^FLChild[11]
^FLChild[12]
^FLChild[13]
^FLChild[14]
^FLChild[15]
^FLChild[16]
^FLChild[17]
^FLChild[18]
^FLChild[19]
^FLChild[20]
96. ^FLDefault[79]

IF SP029_WhatChild = a96

SP024_NameOthChild

IWER:
Record child´s name
NAME OTHER CHILD
STRING

ENDIF

ELSE

LOOP i:= 1 TO 7

IF Sec_SN.SN_Roster[i].SN005_NetworkRelationship = SP009_ToWhomGiveHelp
ELSE
ENDIF

ENDLOOP
IF FoundAPotentialMatchingSNMember = 1
Txt_FL_SP030

SP030_WhatSNmember
Is this a [{Relationship string}] you mentioned earlier?
WHAT SNMEMBER GIVEN FINANCIAL GIFT
^FLSNmember[1]
^FLSNmember[2]
^FLSNmember[3]
^FLSNmember[4]
^FLSNmember[5]

^FLSNmember[6]
^FLSNmember[7]
96. ^FLDefault[80]

ENDIF

*ENDIF*Txt_FL_SP010

SP010_TypesOfHelpGiven
Please look at card 28. Which types of help have you given to this person in the last twelve months?

IWER:
{CodeAll}
WHICH TYPES OF HELP
1. personal care, e.g. dressing, bathing or showering, eating, getting in or out of bed, using the toilet
2. practical household help, e.g. with home repairs, gardening, transportation, shopping, household chores
3. help with paperwork, such as filling out forms, settling financial or legal matters

Txt_FL_SP011

SP011_HowOftGiveHelp
In the last twelve months, how often altogether have you given such help to this person? Was it...

IWER:
{ReadOut}
HOW OFTEN GIVE HELP
1. About daily
2. About every week
3. About every month
4. Less often

IF piIndex <> 3
Txt_FL_SP013

SP013_GiveHelpToOth
(Please look at card 27) Is there any other family member from outside the household, friend, or neighbour to whom you have given personal care or practical household help?
HAVE YOU GIVEN HELP TO OTHERS
1. Yes
5. No

ENDIF

ENDBLOCK
LOOP cnt2:= 2 TO 3

IF HelpFromOutside[cnt2 - 1].SP013_GiveHelpToOth = a1

BLOCK
Txt_FL_SP009

SP009_ToWhomGiveHelp
Which [other] family member from outside the household, friend or neighbour have you helped [most often] in the last twelve months?
TO WHOM DID YOU GIVE HELP
1. Spouse/Partner
2. Mother
3. Father
4. Mother-in-law
5. Father-in-law
6. Stepmother

7. Stepfather
8. Brother
9. Sister
10. Child
11. Step-child/your current partner's child
12. Son-in-law
13. Daughter-in-law
14. Grandchild
15. Grandparent
16. Aunt
17. Uncle
18. Niece
19. Nephew
20. Other relative
21. Friend
22. (Ex-)colleague/co-worker
23. Neighbour
24. Ex-spouse/partner
25. Minister, priest, or other clergy
26. Therapist or other professional helper
27. Housekeeper/Home health care provider
96. None of these

IF (SP009_ToWhomGiveHelp = a10) OR (SP009_ToWhomGiveHelp = a11)

SP029_WhatChild
Which child?
WHAT CHILD GIVEN FINANCIAL GIFT
^FLChild[1]
^FLChild[2]
^FLChild[3]
^FLChild[4]
^FLChild[5]
^FLChild[6]
^FLChild[7]
^FLChild[8]
^FLChild[9]
^FLChild[10]
^FLChild[11]
^FLChild[12]
^FLChild[13]
^FLChild[14]
^FLChild[15]
^FLChild[16]
^FLChild[17]
^FLChild[18]
^FLChild[19]
^FLChild[20]
96. ^FLDefault[79]

IF SP029_WhatChild = a96

SP024_NameOthChild

IWER:
Record child´s name
NAME OTHER CHILD
STRING

ENDIF

ELSE

LOOP i:= 1 TO 7

IF Sec_SN.SN_Roster[i].SN005_NetworkRelationship = SP009_ToWhomGiveHelp
ELSE
ENDIF

ENDLOOP
IF FoundAPotentialMatchingSNMember = 1
Txt_FL_SP030

SP030_WhatSNmember
Is this a [{Relationship string}] you mentioned earlier?
WHAT SNMEMBER GIVEN FINANCIAL GIFT
^FLSNmember[1]
^FLSNmember[2]
^FLSNmember[3]
^FLSNmember[4]
^FLSNmember[5]
^FLSNmember[6]
^FLSNmember[7]
96. ^FLDefault[80]

ENDIF

*ENDIF*Txt_FL_SP010

SP010_TypesOfHelpGiven
Please look at card 28. Which types of help have you given to this person in the last twelve months?

IWER:
{CodeAll}
WHICH TYPES OF HELP
1. personal care, e.g. dressing, bathing or showering, eating, getting in or out of bed, using the toilet
2. practical household help, e.g. with home repairs, gardening, transportation, shopping, household chores
3. help with paperwork, such as filling out forms, settling financial or legal matters

Txt_FL_SP011

SP011_HowOftGiveHelp
In the last twelve months, how often altogether have you given such help to this person? Was it...

IWER:
{ReadOut}
HOW OFTEN GIVE HELP
1. About daily
2. About every week
3. About every month
4. Less often

IF piIndex <> 3
Txt_FL_SP013

SP013_GiveHelpToOth
(Please look at card 27) Is there any other family member from outside the household, friend, or neighbour to whom you have given personal care or practical household help?
HAVE YOU GIVEN HELP TO OTHERS
1. Yes
5. No

ENDIF

ENDBLOCK

ENDIF

ENDLOOP

ENDIF
IF Sec_CH.CH021_NoGrandChild > 0
Txt_FL_SP014

SP014_LkAftGrCh
During the last twelve months, have you regularly or occasionally looked after [your grandchild/ your grandchildren] without the presence of the parents?
LOOK AFTER GRANDCHILDREN
1. Yes
5. No

IF SP014_LkAftGrCh = a1
Txt_FL_SP015

SP015_ParentLkAftGrChild
Which of your children [is the parent of the grandchild/ are the parents of the grandchildren] you have looked after?

IWER:
{CodeAll}
PARENTS FROM GRANDCHILDREN
^FLChild[1]
^FLChild[2]
^FLChild[3]
^FLChild[4]
^FLChild[5]
^FLChild[6]
^FLChild[7]
^FLChild[8]
^FLChild[9]
^FLChild[10]
^FLChild[11]
^FLChild[12]
^FLChild[13]
^FLChild[14]
^FLChild[15]
^FLChild[16]
^FLChild[17]
^FLChild[18]
^FLChild[19]
^FLChild[20]
21. ^FLDefault[1]

LOOP cnt3:= 1 TO 20

IF cnt3 IN SP015_ParentLkAftGrChild

BLOCK
Txt_FL_SP016

SP016_HowOftGrCh
On average, how often did you look after the child(ren) of {FLChildName[i]} in the last twelve months? Was it...

IWER:
{ReadOut}
HOW OFTEN DO YOU LOOK AFTER GRANDCHILDREN
1. About daily
2. About every week
3. About every month
4. Less often

ENDBLOCK

ENDIF

ENDLOOP

ENDIF

ENDIF
IF MN013_HHSize > 1
Txt_FL_SP018

SP018_GiveHelpInHH
Let us now talk about help within your household. Is there someone living in this household whom you have helped regularly during the last twelve months with personal care, such as washing, getting out of bed, or dressing?

IWER:
By regularly we mean daily or almost daily during at least three months. We do not want to capture help during short-term sickness of family members.
GIVEN HELP TO SOMEONE IN THE HOUSEHOLD
1. Yes
5. No

IF SP018_GiveHelpInHH = a1

SP019_ToWhomGiveHelpInHH
Who is that?

IWER:
{CodeAll}
TO WHOM GIVEN HELP IN THIS HOUSEHOLD
1. Spouse/Partner
2. Mother
3. Father
4. Mother-in-law
5. Father-in-law
6. Stepmother
7. Stepfather
8. Brother
9. Sister
10. Child
11. Step-child/your current partner's child
12. Son-in-law
13. Daughter-in-law
14. Grandchild
15. Grandparent
16. Aunt
17. Uncle
18. Niece
19. Nephew
20. Other relative
21. Friend
22. (Ex-)colleague/co-worker

23. Neighbour
24. Ex-spouse/partner
25. Minister, priest, or other clergy
26. Therapist or other professional helper
27. Housekeeper/Home health care provider
96. None of these

CHECK NOT ((SP019_ToWhomGiveHelpInHH.CARDINAL > 1) AND (96 IN SP019_ToWhomGiveHelpInHH)) L1
" [You cannot select ""None of the above"" together with any other answer. Please change your answer]"

IF (a10 IN SP019_ToWhomGiveHelpInHH) OR (a11 IN SP019_ToWhomGiveHelpInHH)

SP031_WhatChild
Which child(ren)?
WHAT CHILD GIVEN FINANCIAL GIFT
^FLChild[1]
^FLChild[2]
^FLChild[3]
^FLChild[4]
^FLChild[5]
^FLChild[6]
^FLChild[7]
^FLChild[8]
^FLChild[9]
^FLChild[10]
^FLChild[11]
^FLChild[12]
^FLChild[13]
^FLChild[14]
^FLChild[15]
^FLChild[16]
^FLChild[17]
^FLChild[18]
^FLChild[19]
^FLChild[20]
96. ^FLDefault[79]

IF a96 IN SP031_WhatChild

SP025_NameOthChild

IWER:
Record child's name
NAME OTHER CHILD
STRING

ENDIF

ELSE

LOOP i:= 1 TO 7

IF Sec_SN.SN_Roster[i].SN005_NetworkRelationship IN SP019_ToWhomGiveHelpInHH
ELSE
ENDIF

ENDLOOP
IF FoundAPotentialMatchingSNMember = 1
Txt_FL_SP032

SP032_WhatSNmember
Are these person(s) you mentioned earlier?

WHAT SNMEMBER GIVEN FINANCIAL GIFT
^FLSNmember[1]
^FLSNmember[2]
^FLSNmember[3]
^FLSNmember[4]
^FLSNmember[5]
^FLSNmember[6]
^FLSNmember[7]
96. ^FLDefault[80]

ENDIF

ENDIF

ENDIF
IF NOT (a96 IN Sec_PH.Health_B2.PH048_HeADLa) AND NOT (a96 IN Sec_PH.Health_B2.PH049_HeADLb)
Txt_FL_SP020

SP020_RecHelpPersCareInHH
And is there someone living in this household who has helped you regularly during the last twelve months with personal care, such as washing, getting out of bed, or dressing?

IWER:
By regularly we mean daily or almost daily during at least three months. We do not want to capture help during short-term sickness.
SOMEONE IN THIS HOUSEHOLD HELPED YOU REGULARLY WITH PERSONAL CARE
1. Yes
5. No

IF SP020_RecHelpPersCareInHH = a1

SP021_FromWhomHelpInHH
Who is that?

IWER:
{CodeAll}
WHO HELPES YOU WITH PERSONAL CARE IN THE HOUSEHOLD
1. Spouse/Partner
2. Mother
3. Father
4. Mother-in-law
5. Father-in-law
6. Stepmother
7. Stepfather
8. Brother
9. Sister
10. Child
11. Step-child/your current partner's child
12. Son-in-law
13. Daughter-in-law
14. Grandchild
15. Grandparent
16. Aunt
17. Uncle
18. Niece
19. Nephew
20. Other relative
21. Friend
22. (Ex-)colleague/co-worker
23. Neighbour
24. Ex-spouse/partner
25. Minister, priest, or other clergy

26. Therapist or other professional helper
27. Housekeeper/Home health care provider
96. None of these

CHECK NOT ((SP021_FromWhomHelpInHH.CARDINAL > 1) AND (96 IN SP021_FromWhomHelpInHH)) L1 "[You cannot select ""None of the above"" together with any other answer. Please change your answer]"

IF (a10 IN SP021_FromWhomHelpInHH) OR (a11 IN SP021_FromWhomHelpInHH)

SP033_WhatChild
Which child(ren)?
WHAT CHILD GIVEN FINANCIAL GIFT
^FLChild[1]
^FLChild[2]
^FLChild[3]
^FLChild[4]
^FLChild[5]
^FLChild[6]
^FLChild[7]
^FLChild[8]
^FLChild[9]
^FLChild[10]
^FLChild[11]
^FLChild[12]
^FLChild[13]
^FLChild[14]
^FLChild[15]
^FLChild[16]
^FLChild[17]
^FLChild[18]
^FLChild[19]
^FLChild[20]
96. ^FLDefault[79]

IF a96 IN SP033_WhatChild

SP026_NameOthChild

IWER:
Record child´s name
NAME OTHER CHILD
STRING

ENDIF

ELSE

LOOP i:= 1 TO 7

IF Sec_SN.SN_Roster[i].SN005_NetworkRelationship IN SP021_FromWhomHelpInHH
ELSE
ENDIF

ENDLOOP
IF FoundAPotentialMatchingSNMember = 1
Txt_FL_SP034

SP034_WhatSNmember
Are these person(s) you mentioned earlier?
WHAT SNMEMBER GIVEN FINANCIAL GIFT
^FLSNmember[1]
^FLSNmember[2]

^FLSNmember[3]
^FLSNmember[4]
^FLSNmember[5]
^FLSNmember[6]
^FLSNmember[7]
96. ^FLDefault[80]

ENDIF

ENDIF

ENDIF

ENDIF

ENDIF

SP022_IntCheck

IWER:
CHECK:
Who answered the questions in this section?
WHO ANSWERED THE QUESTIONS IN SP
1. Respondent only
2. Respondent and proxy
3. Proxy only

System preset value SP902_TimeStampEnd
IF SP902_TimeStampEnd = EMPTY AND SP022_IntCheck <> EMPTY
ENDIF

ENDBLOCK

ENDIF
IF (FT IN Test) OR (ALL IN Test)

BLOCK

IF MN007_NumFinR = 1
Txt_FL_FT001

FT001_Intro
Some people provide financial or material gifts, or support to others such as parents, children, grandchildren, some other kin, or friends or neighbours, and some people don't.
INTRODUCTION FINANCIAL TRANSFERS
1. Continue

System preset value FT901_TimeStampStart
IF FT901_TimeStampStart = EMPTY AND FT001_Intro <> EMPTY
*ENDIF*Txt_FL_FT002

FT002_GiveFiGift250
Now please think about the last twelve months. Not counting any shared housing or shared food, have you [or/ or/ or/ or] [your/ your/ your/ your] [husband/ wife/ partner/ partner] @Bgiven@B any financial or material gift or support to any person inside or outside this household amounting to [FLDefault[32]] [FLDefault[9]] or more?

IWER:
By financial gift we mean giving money, or covering specific types of costs such as those for medical care or insurance, schooling, down payment for a home. Do not include loans or donations to charities.

GIVEN FINANCIAL GIFT 250 OR MORE
1. Yes
5. No

IF [FT002_GiveFiGift250](#) = a1

BLOCK
Txt_FL_FT003

FT003_ToWhomFiGift250
To whom [else] did you [or/ or/ or/ or] [your/ your/ your/ your] [husband/ wife/ partner/ partner] provide a financial gift or assistance [in the last twelve months]?
[Please name the person that you gave or helped most.]

IWER:
Instrument allows to go through the 'give' loop up to three times.
TO WHOM DID YOU PROVIDE FINANCIAL GIFT 250 OR MORE
1. Spouse/Partner
2. Mother
3. Father
4. Mother-in-law
5. Father-in-law
6. Stepmother
7. Stepfather
8. Brother
9. Sister
10. Child
11. Step-child/your current partner's child
12. Son-in-law
13. Daughter-in-law
14. Grandchild
15. Grandparent
16. Aunt
17. Uncle
18. Niece
19. Nephew
20. Other relative
21. Friend
22. (Ex-)colleague/co-worker
23. Neighbour
24. Ex-spouse/partner
25. Minister, priest, or other clergy
26. Therapist or other professional helper
27. Housekeeper/Home health care provider
96. None of these

IF ([FT003_ToWhomFiGift250](#) = a10) OR ([FT003_ToWhomFiGift250](#) = a11)

FT032_WhatChild
Which child?
WHAT CHILD GIVEN FINANCIAL GIFT
^FLChild[1]
^FLChild[2]
^FLChild[3]
^FLChild[4]
^FLChild[5]
^FLChild[6]
^FLChild[7]
^FLChild[8]
^FLChild[9]
^FLChild[10]
^FLChild[11]

^FLChild[12]
^FLChild[13]
^FLChild[14]
^FLChild[15]
^FLChild[16]
^FLChild[17]
^FLChild[18]
^FLChild[19]
^FLChild[20]
96. ^FLDefault[79]

IF FT032_WhatChild = a96

FT022_NameOthChild

IWER:
Record child's name
NAME OTHER CHILD
STRING

ENDIF

ELSE

LOOP i:= 1 TO 7

IF Sec_SN.SN_Roster[i].SN005_NetworkRelationship = FT003_ToWhomFiGift250
ELSE
ENDIF

ENDLOOP
IF FoundAPotentialMatchingSNMember = 1
Txt_FL_FT033

FT033_WhatSNmember
Is this a [{Relationship string}] you mentioned earlier?
WHAT SNMEMBER GIVEN FINANCIAL GIFT
^FLSNmember[1]
^FLSNmember[2]
^FLSNmember[3]
^FLSNmember[4]
^FLSNmember[5]
^FLSNmember[6]
^FLSNmember[7]
96. ^FLDefault[80]

ENDIF

ENDIF
IF piIndex <> 3
Txt_FL_FT007

FT007_OthPFiGift250
Still thinking about the last twelve months: Is there anyone else inside or outside this household whom you [or/ or/ or/ or] [your/ your/ your/ your] [husband/ wife/ partner/ partner] have @bgiven@b any financial or material gift or support amounting to [FLDefault[32]] [FLDefault[9]] or more?
OTHER PERSONS GIVEN FINANCIAL GIFT 250 OR MORE
1. Yes
5. No

ENDIF

ENDBLOCK
LOOP cnt1:= 2 TO 3

IF FT_Given_FinancialAssistance_LOOP[cnt1 - 1].FT007_OthPFiGift250 = a1

BLOCK
Txt_FL_FT003

FT003_ToWhomFiGift250
To whom [else] did you [or/ or/ or/ or] [your/ your/ your/ your] [husband/ wife/ partner/ partner] provide a financial gift or assistance [in the last twelve months]?
[Please name the person that you gave or helped most.]

IWER:
Instrument allows to go through the 'give' loop up to three times.
TO WHOM DID YOU PROVIDE FINANCIAL GIFT 250 OR MORE
1. Spouse/Partner
2. Mother
3. Father
4. Mother-in-law
5. Father-in-law
6. Stepmother
7. Stepfather
8. Brother
9. Sister
10. Child
11. Step-child/your current partner's child
12. Son-in-law
13. Daughter-in-law
14. Grandchild
15. Grandparent
16. Aunt
17. Uncle
18. Niece
19. Nephew
20. Other relative
21. Friend
22. (Ex-)colleague/co-worker
23. Neighbour
24. Ex-spouse/partner
25. Minister, priest, or other clergy
26. Therapist or other professional helper
27. Housekeeper/Home health care provider
96. None of these

IF (FT003_ToWhomFiGift250 = a10) OR (FT003_ToWhomFiGift250 = a11)

FT032_WhatChild
Which child?
WHAT CHILD GIVEN FINANCIAL GIFT
^FLChild[1]
^FLChild[2]
^FLChild[3]
^FLChild[4]
^FLChild[5]
^FLChild[6]
^FLChild[7]
^FLChild[8]
^FLChild[9]
^FLChild[10]
^FLChild[11]

^FLChild[12]
^FLChild[13]
^FLChild[14]
^FLChild[15]
^FLChild[16]
^FLChild[17]
^FLChild[18]
^FLChild[19]
^FLChild[20]
96. ^FLDefault[79]

IF FT032_WhatChild = a96

FT022_NameOthChild

IWER:
Record child's name
NAME OTHER CHILD
STRING

ENDIF

ELSE

LOOP i:= 1 TO 7

IF Sec_SN.SN_Roster[i].SN005_NetworkRelationship = FT003_ToWhomFiGift250
ELSE
ENDIF

ENDLOOP
IF FoundAPotentialMatchingSNMember = 1
Txt_FL_FT033

FT033_WhatSNmember
Is this a [{Relationship string}] you mentioned earlier?
WHAT SNMEMBER GIVEN FINANCIAL GIFT
^FLSNmember[1]
^FLSNmember[2]
^FLSNmember[3]
^FLSNmember[4]
^FLSNmember[5]
^FLSNmember[6]
^FLSNmember[7]
96. ^FLDefault[80]

ENDIF

ENDIF
IF piIndex <> 3
Txt_FL_FT007

FT007_OthPFiGift250
Still thinking about the last twelve months: Is there anyone else inside or outside this household whom you [or/ or/ or/ or] [your/ your/ your/ your] [husband/ wife/ partner/ partner] have @bgiven@b any financial or material gift or support amounting to [FLDefault[32]] [FLDefault[9]] or more?
OTHER PERSONS GIVEN FINANCIAL GIFT 250 OR MORE
1. Yes
5. No

ENDIF

ENDBLOCK

ENDIF

ENDLOOP

*ENDIF*Txt_FL_FT008

FT008_Intro2
We have just asked you about financial or material gifts or support that you may have given someone. Now we would like to know about such gifts and support that you may have received.
INTRODUCTION RECEIVE
1. Continue

Txt_FL_FT009

FT009_RecFiGift250
Please think of the last twelve months. Not counting any shared housing or shared food, have you [or/ or/ or/ or] [your/ your/ your/ your] [husband/ wife/ partner/ partner] @breceived@b any financial or material gift or support from anyone inside or outside this household amounting to [FLDefault[32]] [FLDefault[9]] or more?

IWER:
By financial gift, we mean giving money as a gift or to cover specific types of costs such as those for medical care or insurance, schooling, down payment for a home. Do not include loans or inheritances.
RECEIVED FINANCIAL GIFT OF 250 OR MORE
1. Yes
5. No

IF FT009_RecFiGift250 = a1

BLOCK
Txt_FL_FT010

FT010_FromWhoFiGift250
Who [else] has given you [or/ or/ or/ or] [your/ your/ your/ your] [husband/ wife/ partner/ partner] a financial gift or assistance [in the past twelve months]? [Please name the person that has given or helped you most.]

IWER:
Instrument allows to go through the 'receive' loop up to three times
FROM WHOM RECEIVED FINANCIAL GIFT 250 OR MORE
1. Spouse/Partner
2. Mother
3. Father
4. Mother-in-law
5. Father-in-law
6. Stepmother
7. Stepfather
8. Brother
9. Sister
10. Child
11. Step-child/your current partner's child
12. Son-in-law
13. Daughter-in-law
14. Grandchild
15. Grandparent
16. Aunt
17. Uncle
18. Niece
19. Nephew
20. Other relative

21. Friend
22. (Ex-)colleague/co-worker
23. Neighbour
24. Ex-spouse/partner
25. Minister, priest, or other clergy
26. Therapist or other professional helper
27. Housekeeper/Home health care provider
96. None of these

IF (FT010_FromWhoFiGift250 = a10) OR (FT010_FromWhoFiGift250 = a11)

FT034_WhatChild
Which child?
WHAT CHILD GIVEN FINANCIAL GIFT
^FLChild[1]
^FLChild[2]
^FLChild[3]
^FLChild[4]
^FLChild[5]
^FLChild[6]
^FLChild[7]
^FLChild[8]
^FLChild[9]
^FLChild[10]
^FLChild[11]
^FLChild[12]
^FLChild[13]
^FLChild[14]
^FLChild[15]
^FLChild[16]
^FLChild[17]
^FLChild[18]
^FLChild[19]
^FLChild[20]
96. ^FLDefault[79]

IF FT034_WhatChild = a96

FT023_NameOthChild

IWER:
Record child's name
NAME OTHER CHILD
STRING

ENDIF

ELSE

LOOP i:= 1 TO 7

IF Sec_SN.SN_Roster[i].SN005_NetworkRelationship = FT010_FromWhoFiGift250
ELSE
ENDIF

ENDLOOP
IF FoundAPotentialMatchingSNMember = 1
Txt_FL_FT035

FT035_WhatSNmember
Is this a [{Relationship string}] you mentioned earlier?

WHAT SNMEMBER GIVEN FINANCIAL GIFT
^FLSNmember[1]
^FLSNmember[2]
^FLSNmember[3]
^FLSNmember[4]
^FLSNmember[5]
^FLSNmember[6]
^FLSNmember[7]
96. ^FLDefault[80]

ENDIF

ENDIF
IF piIndex <> 3
Txt_FL_FT014

FT014_FromOthPFiGift250
(Still thinking about the last twelve months). Is there anyone else inside or outside this household who has given you [or/ or/ or/ or] [your/ your/ your/ your] [husband/ wife/ partner/ partner] any financial or material gift or support amounting to [FLDefault[32]] [FLDefault[9]] or more?
FROM OTHER PERSONS RECEIVED FINANCIAL GIFT 250 OR MORE
1. Yes
5. No

ENDIF

ENDBLOCK
LOOP cnt2:= 2 TO 3

IF FT_Provide_FinancialAssistance_LOOP[cnt2 - 1].FT014_FromOthPFiGift250 = a1

BLOCK
Txt_FL_FT010

FT010_FromWhoFiGift250
Who [else] has given you [or/ or/ or/ or] [your/ your/ your/ your] [husband/ wife/ partner/ partner] a financial gift or assistance [in the past twelve months]? [Please name the person that has given or helped you most.]

IWER:
Instrument allows to go through the 'receive' loop up to three times
FROM WHOM RECEIVED FINANCIAL GIFT 250 OR MORE
1. Spouse/Partner
2. Mother
3. Father
4. Mother-in-law
5. Father-in-law
6. Stepmother
7. Stepfather
8. Brother
9. Sister
10. Child
11. Step-child/your current partner's child
12. Son-in-law
13. Daughter-in-law
14. Grandchild
15. Grandparent
16. Aunt
17. Uncle
18. Niece
19. Nephew
20. Other relative

21. Friend
22. (Ex-)colleague/co-worker
23. Neighbour
24. Ex-spouse/partner
25. Minister, priest, or other clergy
26. Therapist or other professional helper
27. Housekeeper/Home health care provider
96. None of these

IF (FT010_FromWhoFiGift250 = a10) OR (FT010_FromWhoFiGift250 = a11)

FT034_WhatChild
Which child?
WHAT CHILD GIVEN FINANCIAL GIFT
^FLChild[1]
^FLChild[2]
^FLChild[3]
^FLChild[4]
^FLChild[5]
^FLChild[6]
^FLChild[7]
^FLChild[8]
^FLChild[9]
^FLChild[10]
^FLChild[11]
^FLChild[12]
^FLChild[13]
^FLChild[14]
^FLChild[15]
^FLChild[16]
^FLChild[17]
^FLChild[18]
^FLChild[19]
^FLChild[20]
96. ^FLDefault[79]

IF FT034_WhatChild = a96

FT023_NameOthChild

IWER:
Record child's name
NAME OTHER CHILD
STRING

ENDIF

ELSE

LOOP i:= 1 TO 7

IF Sec_SN.SN_Roster[i].SN005_NetworkRelationship = FT010_FromWhoFiGift250
ELSE
ENDIF

ENDLOOP
IF FoundAPotentialMatchingSNMember = 1
Txt_FL_FT035

FT035_WhatSNmember
Is this a [{Relationship string}] you mentioned earlier?

WHAT SNMEMBER GIVEN FINANCIAL GIFT
^FLSNmember[1]
^FLSNmember[2]
^FLSNmember[3]
^FLSNmember[4]
^FLSNmember[5]
^FLSNmember[6]
^FLSNmember[7]
96. ^FLDefault[80]

ENDIF

ENDIF
IF piIndex <> 3
Txt_FL_FT014

FT014_FromOthPFiGift250
(Still thinking about the last twelve months). Is there anyone else inside or outside this household who has given you [or/ or/ or/ or] [your/ your/ your/ your] [husband/ wife/ partner/ partner] any financial or material gift or support amounting to [FLDefault[32]] [FLDefault[9]] or more?
FROM OTHER PERSONS RECEIVED FINANCIAL GIFT 250 OR MORE
1. Yes
5. No

ENDIF

ENDBLOCK

ENDIF

ENDLOOP

*ENDIF*Txt_FL_FT015

FT015_EverRecInh5000
[Not counting any large gift we may have already talked about/ Since our interview in], have you [or/ or/ or/ or] [your/ your/ your/ your] [husband/ wife/ partner/ partner] [ever/ {Preloaded month and year}] @breceived@b a gift or inherited money, goods, or property worth more than [FLDefault[33]] [FLDefault[9]] ?

IWER:
Not including any gifts you have already mentioned
EVER RECEIVED GIFT OR INHERITED MONEY 5000 OR MORE
1. Yes
5. No

IF FT015_EverRecInh5000 = a1

BLOCK
Txt_FL_FT016

FT016_YearRecInh5000
[Think of the largest gift or inheritance you received.] In which year did you [or/ or/ or/ or] [your/ your/ your/ your] [husband/ wife/ partner/ partner] receive it?
IN WHICH YEAR GIFT OR INHERITANCE RECEIVED
1905..2015
Txt_FL_FT017

FT017_FromWhomRecInh5000
From whom did you [or/ or/ or/ or] [your/ your/ your/ your] [husband/ wife/ partner/ partner] receive this gift or inheritance?
FROM WHOM INHERITED 5000 OR MORE

1. Spouse/Partner
2. Mother
3. Father
4. Mother-in-law
5. Father-in-law
6. Stepmother
7. Stepfather
8. Brother
9. Sister
10. Child
11. Step-child/your current partner's child
12. Son-in-law
13. Daughter-in-law
14. Grandchild
15. Grandparent
16. Aunt
17. Uncle
18. Niece
19. Nephew
20. Other relative
21. Friend
22. (Ex-)colleague/co-worker
23. Neighbour
24. Ex-spouse/partner
25. Minister, priest, or other clergy
26. Therapist or other professional helper
27. Housekeeper/Home health care provider
96. None of these

IF (FT017_FromWhomRecInh5000 = a10) OR (FT017_FromWhomRecInh5000 = a11)

FT036_WhatChild
Which child?
WHAT CHILD GIVEN FINANCIAL GIFT
^FLChild[1]
^FLChild[2]
^FLChild[3]
^FLChild[4]
^FLChild[5]
^FLChild[6]
^FLChild[7]
^FLChild[8]
^FLChild[9]
^FLChild[10]
^FLChild[11]
^FLChild[12]
^FLChild[13]
^FLChild[14]
^FLChild[15]
^FLChild[16]
^FLChild[17]
^FLChild[18]
^FLChild[19]
^FLChild[20]
96. ^FLDefault[79]

IF FT036_WhatChild = a96

FT024_NameOthChild

IWER:

Record child's name
NAME OTHER CHILD
STRING

ENDIF

ELSE

LOOP i:= 1 TO 7

IF Sec_SN.SN_Roster[i].SN005_NetworkRelationship = FT017_FromWhomRecInh5000
ELSE
ENDIF

ENDLOOP
IF FoundAPotentialMatchingSNMember = 1
Txt_FL_FT037

FT037_WhatSNmember
Is this a [{Relationship string}] you mentioned earlier?
WHAT SNMEMBER GIVEN FINANCIAL GIFT
^FLSNmember[1]
^FLSNmember[2]
^FLSNmember[3]
^FLSNmember[4]
^FLSNmember[5]
^FLSNmember[6]
^FLSNmember[7]
96. ^FLDefault[80]

ENDIF

ENDIF
IF piIndex <> 5
Txt_FL_FT020

FT020_MoreRecInh5000
Did you [or/ or/ or/ or] [your/ your/ your/ your] [husband/ wife/ partner/ partner] receive any further gift or inheritance worth more than [FLDefault[33]] [FLDefault[9]] [Since our interview in] {FLLastInterviewMonthYear}?
ANY FURTHER GIFT OR INHERITANCE
1. Yes
5. No

ENDIF

ENDBLOCK
LOOP cnt3:= 2 TO 5

IF FT_Receive_FinancialAssistance_LOOP[cnt3 - 1].FT020_MoreRecInh5000 = a1

BLOCK
Txt_FL_FT016

FT016_YearRecInh5000
[Think of the largest gift or inheritance you received.] In which year did you [or/ or/ or/ or] [your/ your/ your/ your] [husband/ wife/ partner/ partner] receive it?
IN WHICH YEAR GIFT OR INHERITANCE RECEIVED
1905..2015
Txt_FL_FT017

FT017_FromWhomRecInh5000

From whom did you [or/ or/ or/ or] [your/ your/ your/ your] [husband/ wife/ partner/ partner] receive this gift or inheritance?
FROM WHOM INHERITED 5000 OR MORE
1. Spouse/Partner
2. Mother
3. Father
4. Mother-in-law
5. Father-in-law
6. Stepmother
7. Stepfather
8. Brother
9. Sister
10. Child
11. Step-child/your current partner's child
12. Son-in-law
13. Daughter-in-law
14. Grandchild
15. Grandparent
16. Aunt
17. Uncle
18. Niece
19. Nephew
20. Other relative
21. Friend
22. (Ex-)colleague/co-worker
23. Neighbour
24. Ex-spouse/partner
25. Minister, priest, or other clergy
26. Therapist or other professional helper
27. Housekeeper/Home health care provider
96. None of these

IF (FT017_FromWhomRecInh5000 = a10) OR (FT017_FromWhomRecInh5000 = a11)

FT036_WhatChild
Which child?
WHAT CHILD GIVEN FINANCIAL GIFT
^FLChild[1]
^FLChild[2]
^FLChild[3]
^FLChild[4]
^FLChild[5]
^FLChild[6]
^FLChild[7]
^FLChild[8]
^FLChild[9]
^FLChild[10]
^FLChild[11]
^FLChild[12]
^FLChild[13]
^FLChild[14]
^FLChild[15]
^FLChild[16]
^FLChild[17]
^FLChild[18]
^FLChild[19]
^FLChild[20]
96. ^FLDefault[79]

IF FT036_WhatChild = a96

FT024_NameOthChild

IWER:
Record child's name
NAME OTHER CHILD
STRING

ENDIF

ELSE

LOOP i:= 1 TO 7

IF Sec_SN.SN_Roster[i].SN005_NetworkRelationship = FT017_FromWhomRecInh5000
ELSE
ENDIF

ENDLOOP
IF FoundAPotentialMatchingSNMember = 1
Txt_FL_FT037

FT037_WhatSNmember
Is this a [{Relationship string}] you mentioned earlier?
WHAT SNMEMBER GIVEN FINANCIAL GIFT
^FLSNmember[1]
^FLSNmember[2]
^FLSNmember[3]
^FLSNmember[4]
^FLSNmember[5]
^FLSNmember[6]
^FLSNmember[7]
96. ^FLDefault[80]

ENDIF

ENDIF
IF piIndex <> 5
Txt_FL_FT020

FT020_MoreRecInh5000
Did you [or/ or/ or/ or] [your/ your/ your/ your] [husband/ wife/ partner/ partner] receive any further gift or inheritance worth more than [FLDefault[33]] [FLDefault[9]] [Since our interview in] {FLLastInterviewMonthYear}?
ANY FURTHER GIFT OR INHERITANCE
1. Yes
5. No

ENDIF

ENDBLOCK

ENDIF

ENDLOOP

*ENDIF*Txt_FL_FT025

FT025_EVER_GIFT_5000_OR_MORE
[Not counting any large gift we may have already talked about/ Since our last interview in], have you [or/ or/ or/ or] [your/ your/ your/ your] [husband/ wife/ partner/ partner] {FL_FT025_7} [ever] @bgiven@b a gift of money, goods, or property worth more than [FLDefault[33]] [FLDefault[9]] ?

IWER:
Not including any gifts you have already mentioned
EVER GIVEN GIFT 5000 OR MORE
1. Yes
5. No

IF FT025_EVER_GIFT_5000_OR_MORE = a1

BLOCK
Txt_FL_FT026

FT026_YearGivInh5000
[Think of the largest gift you gave.] In which year did you [or/ or/ or/ or] [your/ your/ your/ your] [husband/ wife/ partner/ partner] give it?
IN WHICH YEAR GIFT GIVEN
1905..2015
Txt_FL_FT027

FT027_ToWhomGivInh5000
To whom did you [or/ or/ or/ or] [your/ your/ your/ your] [husband/ wife/ partner/ partner] give this gift?
TO WHOM GIVEN 5000 OR MORE
1. Spouse/Partner
2. Mother
3. Father
4. Mother-in-law
5. Father-in-law
6. Stepmother
7. Stepfather
8. Brother
9. Sister
10. Child
11. Step-child/your current partner's child
12. Son-in-law
13. Daughter-in-law
14. Grandchild
15. Grandparent
16. Aunt
17. Uncle
18. Niece
19. Nephew
20. Other relative
21. Friend
22. (Ex-)colleague/co-worker
23. Neighbour
24. Ex-spouse/partner
25. Minister, priest, or other clergy
26. Therapist or other professional helper
27. Housekeeper/Home health care provider
96. None of these

IF (FT027_ToWhomGivInh5000 = a10) OR (FT027_ToWhomGivInh5000 = a11)

FT038_WhatChild
Which child?
WHAT CHILD GIVEN FINANCIAL GIFT
^FLChild[1]
^FLChild[2]
^FLChild[3]
^FLChild[4]
^FLChild[5]
^FLChild[6]
^FLChild[7]

^FLChild[8]
^FLChild[9]
^FLChild[10]
^FLChild[11]
^FLChild[12]
^FLChild[13]
^FLChild[14]
^FLChild[15]
^FLChild[16]
^FLChild[17]
^FLChild[18]
^FLChild[19]
^FLChild[20]
96. ^FLDefault[79]

IF FT038_WhatChild = a96

FT028_NameOthChild

IWER:
Record child's name
NAME OTHER CHILD
STRING

ENDIF

ELSE

LOOP i:= 1 TO 7

IF Sec_SN.SN_Roster[i].SN005_NetworkRelationship = FT027_ToWhomGivInh5000
ELSE
ENDIF

ENDLOOP
IF FoundAPotentialMatchingSNMember = 1
Txt_FL_FT039

FT039_WhatSNmember
Is this a [{Relationship string}] you mentioned earlier?
WHAT SNMEMBER GIVEN FINANCIAL GIFT
^FLSNmember[1]
^FLSNmember[2]
^FLSNmember[3]
^FLSNmember[4]
^FLSNmember[5]
^FLSNmember[6]
^FLSNmember[7]
96. ^FLDefault[80]

ENDIF

ENDIF
IF piIndex <> 5
Txt_FL_FT031

FT031_MoreGivInh5000
Did you [or/ or/ or/ or] [your/ your/ your/ your] [husband/ wife/ partner/ partner] give any further gift worth more than [FLDefault[33]] [FLDefault[9]] {FL_FT031_4}?
ANY FURTHER GIFT
1. Yes

5. No

ENDIF

ENDBLOCK
LOOP cnt4:= 2 TO 5

IF FT_Give_FinancialAssistance_LOOP[cnt4 - 1].FT031_MoreGivInh5000 = a1

BLOCK
Txt_FL_FT026

FT026_YearGivInh5000
[Think of the largest gift you gave.] In which year did you [or/ or/ or/ or] [your/ your/ your/ your] [husband/ wife/ partner/ partner] give it?
IN WHICH YEAR GIFT GIVEN
1905..2015
Txt_FL_FT027

FT027_ToWhomGivInh5000
To whom did you [or/ or/ or/ or] [your/ your/ your/ your] [husband/ wife/ partner/ partner] give this gift?
TO WHOM GIVEN 5000 OR MORE
1. Spouse/Partner
2. Mother
3. Father
4. Mother-in-law
5. Father-in-law
6. Stepmother
7. Stepfather
8. Brother
9. Sister
10. Child
11. Step-child/your current partner's child
12. Son-in-law
13. Daughter-in-law
14. Grandchild
15. Grandparent
16. Aunt
17. Uncle
18. Niece
19. Nephew
20. Other relative
21. Friend
22. (Ex-)colleague/co-worker
23. Neighbour
24. Ex-spouse/partner
25. Minister, priest, or other clergy
26. Therapist or other professional helper
27. Housekeeper/Home health care provider
96. None of these

IF (FT027_ToWhomGivInh5000 = a10) OR (FT027_ToWhomGivInh5000 = a11)

FT038_WhatChild
Which child?
WHAT CHILD GIVEN FINANCIAL GIFT
^FLChild[1]
^FLChild[2]
^FLChild[3]
^FLChild[4]
^FLChild[5]
^FLChild[6]

^FLChild[7]
^FLChild[8]
^FLChild[9]
^FLChild[10]
^FLChild[11]
^FLChild[12]
^FLChild[13]
^FLChild[14]
^FLChild[15]
^FLChild[16]
^FLChild[17]
^FLChild[18]
^FLChild[19]
^FLChild[20]
96. ^FLDefault[79]

IF FT038_WhatChild = a96

FT028_NameOthChild

IWER:
Record child's name
NAME OTHER CHILD
STRING

ENDIF

ELSE

LOOP i:= 1 TO 7

IF Sec_SN.SN_Roster[i].SN005_NetworkRelationship = FT027_ToWhomGivInh5000
ELSE
ENDIF

ENDLOOP
IF FoundAPotentialMatchingSNMember = 1
Txt_FL_FT039

FT039_WhatSNmember
Is this a [{Relationship string}] you mentioned earlier?
WHAT SNMEMBER GIVEN FINANCIAL GIFT
^FLSNmember[1]
^FLSNmember[2]
^FLSNmember[3]
^FLSNmember[4]
^FLSNmember[5]
^FLSNmember[6]
^FLSNmember[7]
96. ^FLDefault[80]

ENDIF

ENDIF
IF piIndex <> 5
Txt_FL_FT031

FT031_MoreGivInh5000
Did you [or/ or/ or/ or] [your/ your/ your/ your] [husband/ wife/ partner/ partner] give any further gift worth more than [FLDefault[33]] [FLDefault[9]] {FL_FT031_4}?
ANY FURTHER GIFT

1. Yes
5. No

ENDIF

ENDBLOCK

ENDIF

ENDLOOP

ENDIF

FT021_IntCheck

IWER:
CHECK:
Who answered the questions in this section?
WHO ANSWERED THE QUESTIONS IN FT
1. Respondent only
2. Respondent and proxy
3. Proxy only

System preset value FT902_TimeStampEnd
IF FT902_TimeStampEnd = EMPTY AND FT021_IntCheck <> EMPTY
ENDIF

ENDIF

ENDBLOCK

ENDIF
IF (HO IN Test) OR (ALL IN Test)

BLOCK

IF MN008_NumHHR = 1

IF MN024_NursingHome = a2
Txt_FL_HO061

HO061_YrsAcc
Now I have a few questions about your residence. How many years have you lived in your present accommodation?

IWER:
Round up to full years
YEARS IN ACCOMMODATION
1..120

System preset value HO901_TimeStampStart
IF HO901_TimeStampStart = EMPTY AND HO061_YrsAcc <> EMPTY
*ENDIF*Txt_FL_HO662

HO662_PayNursHome
Do you have to pay "out of pocket" for your nursing home accommodation? @\"Out of pocket" are expenses that are not reimbursed by private or public insurance or covered by benefits. @\Expenses can be room, meals, care, laundry or charges and services, such as water, electricity, gas, or heating etc.
OUT OF POCKET FOR NURSING HOME
1. Yes
5. No

IF HO662_PayNursHome = a1

Txt_FL_HO665

HO665_LastPayment

Can you please estimate how much do you pay out of pocket for a typical month?

IWER:
Amount in [FLDefault[9]]
LAST PAYMENT
-100000000000000000..1000000000000000000

IF HO665_LastPayment = NONRESPONSE

UB SEQUENCE UB_HO965

ENDIF

HO666_PayCoverNursHome

Please look at card 29. What did this payment cover?

IWER:
{CodeAll}. Read out if necessary.
PAYMENT COVERING NURSING HOME

1. Lodging (room)
2. Meals
3. Nursing and care services
4. Rehabilitation and other health services
5. Laundry
6. Charges and services, such as water, electricity, gas, or heating
7. Other expenses
96. None of the above

CHECK NOT ((HO666_PayCoverNursHome.CARDINAL > 1) AND (96 IN HO666_PayCoverNursHome)) L1 "[You cannot select ""None of the above"" together with any other answer. Please change your answer]"

Txt_FL_HO080

HO080_NHCosts

It is important to understand how people cope with nursing home expenses. We have one more question to assess how you manage. Please look at card 30. Which of these income sources are used in order to cover your expenses?

IWER:
{CodeAll}
INCOME SOURCES USED TO COVER NURSING HOME EXPENSES

1. Pensions (yours or your spouse)
2. Other sources of income, such as rents from real estate, annuities etc.
3. Assets or savings (yours or your spouse), including life insurance policies
4. Contributions from children or grandchildren
5. Housing allowances or other public benefits
6. Payments from a public long-term care insurance
7. Payments from a private long-term care insurance
97. Other income sources (specify)

IF a97 IN HO080_NHCosts

HO081_OtherNHCosts

What other income sources are used?
OTHER INCOME SOURCES USED TO COVER NURSING HOME EXPENSES
STRING

ENDIF

*ENDIF*Txt_FL_HO075

HO075_OwnRealEstate
Do you own secondary homes, holiday homes, other real estate, land or forestry, including the home you occupied before living in this NH?

IWER:
Please do not include: time-sharing arrangement, own business
OWN REAL ESTATE
1. Yes
5. No

IF HO075_OwnRealEstate = a1
Txt_FL_HO076

HO076_ValueRE
In your opinion, how much would this or these properties be worth now if you sold it?

IWER:
If R owns property abroad, give value in [FLDefault[9]]
VALUE OF REAL ESTATE
-1000000000000000000..1000000000000000000
CHECK NOT ((HO076_ValueRE = 0) AND (HO076_ValueRE = RESPONSE)) L1 " [Amount is expected to be higher than zero]"

IF HO076_ValueRE = NONRESPONSE
UB SEQUENCE UB_HO976

*ENDIF*Txt_FL_HO077

HO077_RecIncRe
Did you receive any income or rent from these properties in [STR (Year - 1)]?
RECEIVE INCOME OR RENT OF REAL ESTATE
1. Yes
5. No

IF HO077_RecIncRe = a1
Txt_FL_HO078

HO078_AmIncRe
How much income or rent did you receive from these properties during [STR (Year - 1)], after taxes?

IWER:
Amount in [FLDefault[9]]
AMOUNT INCOME OR RENT OF REAL ESTATE LAST YEAR
-1000000000000000000..1000000000000000000

IF HO078_AmIncRe = NONRESPONSE
UB SEQUENCE UB_HO978

ENDIF

ENDIF

ENDIF

ELSE

IF MN024_NursingHome = a1

HO001_Place

IWER:
Does the interview take place in the respondent's house or flat?
INTERVIEW IN HOUSE R
1. Yes
5. No

System preset value HO901_TimeStampStart
IF HO901_TimeStampStart = EMPTY AND HO001_Place <> EMPTY
ENDIF
IF MN101_Longitudinal = 1
Txt_FL_HO044

HO044_ChangeResidence
Now I have a few questions about your residence. Since {FLLastInterviewMonthYear}, have you moved to another residence, house, or flat?
CHANGE PLACE OF RESIDENCE
1. Yes
5. No

*ENDIF*Txt_FL_HO002

HO002_OwnerTenant
Please look at card 31. Is your household occupying the dwelling you live in as

IWER:
{ReadOut}
OWNER, TENANT OR RENT FREE
1. Owner
2. Member of a cooperative
3. Tenant
4. Subtenant
5. Rent free

IF ((HO002_OwnerTenant = a1) OR (HO002_OwnerTenant = a2)) OR (HO002_OwnerTenant = a5)
Txt_FL_HO067

HO067_PaymSimDwel
In your opinion, how much would you pay as monthly rent if you rented a similar dwelling, unfurnished, on the free market today?

IWER:
Exclude charges and services such as electricity or heating. Amount in [FLDefault[9]]
PAYMENT SIMILAR DWELLING
-1000000000000000000..1000000000000000000
CHECK NOT ((HO067_PaymSimDwel = 0) AND (HO067_PaymSimDwel = RESPONSE)) L1 " [Amount is expected to be higher than zero]"

IF HO067_PaymSimDwel = NONRESPONSE
UB SEQUENCE UB_HO967

ENDIF

ENDIF
IF (HO002_OwnerTenant = a3) OR (HO002_OwnerTenant = a4)
Txt_FL_HO003

HO003_Period
[Coming back to your current rent and thinking about your last payment/ Thinking about your last rent payment], what period did this cover? Was that

IWER:
{ReadOut}
RENT PAYMENT PERIOD
1. A week
2. A month
3. Three months
4. Six months
5. A year
97. Other period of time

IF HO003_Period = a97
Txt_FL_HO004

HO004_OthPer
What other period do you mean?
OTHER PERIOD
STRING

ENDIFTxt_FL_HO605

HO605_LastPayment
How much was your last gross rent payment, that is not subtracting housing subsidies or allowances you might get?

IWER:
Amount in [FLDefault[9]]
LAST PAYMENT
-100000000000000000..1000000000000000000
CHECK NOT ((HO605_LastPayment = 0) AND (HO605_LastPayment = RESPONSE)) L1 " [Amount is expected to be higher than zero]"

IF HO605_LastPayment = NONRESPONSE
UB SEQUENCE UB_HO905

ENDIF
IF HO002_OwnerTenant = a3
Txt_FL_HO079

HO079_SocialHousing
Do you live in a social or public housing accommodation, or equivalent?
SOCIAL HOUSING
1. Yes
5. No

ENDIFTxt_FL_HO007

HO007_LastPayIncl
Did your last payment include all charges and services, such as water charges, garbage removal, upkeep of common space, electricity, gas, or heating?
LAST PAYMENT INCLUDE ALL CHARGES AND SERVICES
1. Yes
5. No

IF HO007_LastPayIncl = a5
Txt_FL_HO008

HO008_ExtRentIncl
About how much did you pay for charges and services that were not included in your rent during the last [week/ month/ three months/ six months/ year/piHO004_OthPer]?

IWER:

Amount in [FLDefault[9]]
CHARGES AND SERVICES
-1000000000000000000..1000000000000000000
CHECK NOT ((HO008_ExtRentIncl = 0) AND (HO008_ExtRentIncl = RESPONSE)) L1 " [Amount is expected to be higher than zero]"

IF HO008_ExtRentIncl = NONRESPONSE
UB SEQUENCE UB_HO908

ENDIF

ENDIF
IF (HO002_OwnerTenant = a3) OR (HO002_OwnerTenant = a4)
Txt_FL_HO010

HO010_BehRent
In the last twelve months, have you ever found yourself more than two months behind with your rent?
BEHIND WITH RENT
1. Yes
5. No

ENDIF

ENDIF
IF (HO002_OwnerTenant = a1) OR (HO002_OwnerTenant = a2)
Txt_FL_HO070

HO070_PercHouseOwn
What percentage or share of this dwelling is owned by you [and/ and/ and/ and] [your/ your/ your/ your] [husband/ wife/ partner/ partner]?

IWER:
Enter percentage
For partners: The requested percentage refers to the sum of both shares.
0 is allowed only if neither partner owns any fraction!
PERCENTAGE HOUSE OWNED
0..100

IF HO070_PercHouseOwn > 0

IF ((MN101_Longitudinal = 1) AND (HO044_ChangeResidence = a1)) OR (MN101_Longitudinal = 0)
Txt_FL_HO611

HO611_AcqProp
Please look at card 32. How did you acquire this property?

IWER:
{CodeAll}
HOW PROPERTY ACQUIRED
1. Purchased or built it with own means
2. Purchased or built it with a loan or mortgage
3. Purchased or built it with help from family
4. Received it as a bequest
5. Received it as a gift
6. Acquired it through other means

Txt_FL_HO012

HO012_YearHouse
In which year was that?
YEAR ACQUIRED THE HOUSE
1900..2015

*ENDIF*Txt_FL_HO013

HO013_MortLoanProp
Do you have mortgages or loans on this property?
MORTGAGES OR LOANS ON PROPERTY
1. Yes
5. No

IF HO013_MortLoanProp = a1

HO014_YrsLMortLoan
How many years do your mortgages or loans on this property have left to run?

IWER:
If less than one year, code 1, if more than 50 or no fixed limit code 51
YEARS LEFT OF MORTGAGE OR LOAN
1..51
Txt_FL_HO015

HO015_AmToPayMortLoan
How much do you [or/ or/ or/ or] [your/ your/ your/ your] [husband/ wife/ partner/ partner] still have to pay on your mortgages or loans, excluding interest?

IWER:
Total amount in [FLDefault[9]]
AMOUNT STILL TO PAY ON MORTGAGE OR LOAN
-1000000000000000000..1000000000000000000
CHECK NOT ((HO015_AmToPayMortLoan = 0) AND (HO015_AmToPayMortLoan = RESPONSE)) L1 "[Amount is expected to be higher than zero]"

IF HO015_AmToPayMortLoan = NONRESPONSE
UB SEQUENCE UB_HO915

*ENDIF*Txt_FL_HO017

HO017_RepayMortgLoans
Do you regularly repay your mortgages or loans?
REGULARLY REPAY MORTGAGE OR LOANS
1. Yes
5. No

IF HO017_RepayMortgLoans = a1
Txt_FL_HO620

HO620_RegRepayMortLoan
In the last twelve months, about how much did you pay for all mortgages and loans outstanding on this property?

IWER:
Amount in [FLDefault[9]]
AMOUNT REGULAR REPAYMENTS ON MORTGAGE OR LOAN
-1000000000000000000..1000000000000000000
CHECK NOT ((HO620_RegRepayMortLoan = 0) AND (HO620_RegRepayMortLoan = RESPONSE)) L1 "[Amount is expected to be higher than zero]"

IF HO620_RegRepayMortLoan = NONRESPONSE
UB SEQUENCE UB_HO920

*ENDIF*Txt_FL_HO022

HO022_BehRepayMortLoan
In the last twelve months, have you ever found yourself more than two months behind with these

repayments?
BEHIND WITH REPAYMENTS MORTGAGE OR LOAN
1. Yes
5. No

ENDIF

ENDIF

ENDIF

ENDIF
IF HO002_OwnerTenant <> a5
Txt_FL_HO023

HO023_SuBLAcc
Do you [let/ sublet] parts of this accommodation?
SUBLET OR LET PARTS OF ACCOMMODATION
1. Yes
5. No

IF HO023_SuBLAcc = a1
Txt_FL_HO074

HO074_IncSuBLAcc
How much income or rent did you [or/ or/ or/ or] [your/ your/ your/ your] [husband/ wife/ partner/ partner] receive from letting this accomodation during [STR (Year - 1)], after taxes?

IWER:
Amount in [FLDefault[9]]
INCOME FROM SUBLET OR LET PARTS OF ACCOMMODATION
-100000000000000000..1000000000000000000
CHECK NOT ((HO074_IncSuBLAcc <= 0) AND (HO074_IncSuBLAcc = RESPONSE)) L1 " [Amount is expected to be higher than zero]"

ENDIF

ENDIF
IF (HO002_OwnerTenant = a1) OR (HO002_OwnerTenant = a2)
Txt_FL_HO024

HO024_ValueH
In your opinion, how much would you receive if you sold your property today?

IWER:
Amount in [FLDefault[9]]
VALUE OF THE HOUSE
-100000000000000000..1000000000000000000
CHECK NOT ((HO024_ValueH = 0) AND (HO024_ValueH = RESPONSE)) L1 " [Amount is expected to be higher than zero]"

IF HO024_ValueH = NONRESPONSE
UB SEQUENCE UB_HO924

ENDIF

ENDIF
IF ((MN101_Longitudinal = 1) AND (HO044_ChangeResidence = a1)) OR (MN101_Longitudinal = 0)
Txt_FL_HO032

HO032_NoRoomSqm
How many rooms do you have for your household members' personal use, including bedrooms but

excluding kitchen, bathrooms, and hallways [and any rooms you may let or sublet]?

IWER:
Do not count boxroom, cellar, attic etc.
NUMBER OF ROOMS
1..25

*ENDIF*Txt_FL_HO633

HO633_SpecFeat
Please look at card 33. Which of the following special features that assist people who have physical impairments or health problems does your home have, if any?

IWER:
{CodeAll}
SPECIAL FEATURES IN THE HOUSE
1. Widened doors or corridors
2. Ramps or street level entrances
3. Hand rails
4. Automatic or easy open doors or gates
5. Bathroom or toilet modifications
6. Kitchen modifications
7. Chair lifts or stair glides
8. Alerting devices (button alarms, detectors...)
96. None of these
97. Other (specify)

CHECK NOT ((HO633_SpecFeat.CARDINAL > 1) AND (96 IN HO633_SpecFeat)) L1 " [You cannot select ""None of the above"" together with any other answer. Please change your answer]"

IF a97 IN HO633_SpecFeat

HO631_SpecFeat

IWER:
Note other feature
OTHER SPECIAL FEATURES
STRING

ENDIF
IF ((MN101_Longitudinal = 1) AND (HO044_ChangeResidence = a1)) OR (MN101_Longitudinal = 0)
Txt_FL_HO034

HO034_YrsAcc
How many years have you been living in your present accommodation?

IWER:
Round up to full years
YEARS IN ACCOMMODATION
0..120

IF MN002_Person[2].RespId <> EMPTY
Txt_FL_HO060

HO060_PartnerYrsAcc
How many years has [your/ your/ your/ your] [husband/ wife/ partner/ partner] been living in your present accommodation?

IWER:
Round up to full years
PARTNER YEARS IN ACCOMMODATION
0..120

ENDIF
IF HO001_Place = a5
Txt_FL_HO636

HO636_TypeAcc
Please look at card 34.
What type of building does your household live in?

IWER:
{ReadOut}
A nursing home provides all of the following services for its residents: dispensing of medication, available, 24-hour personal assistance and supervision (not necessarily a nurse), and room & meals
TYPE OF BUILDING
1. A farm house
2. A free standing one or two family house
3. A one or two family house as row or double house
4. A building with 3 to 8 flats
5. A building with 9 or more flats but no more than 8 floors
6. A high-rise with 9 or more floors
7. A housing complex with services for older people (residential home or sheltered housing, but not a nursing home)
8. A nursing home

Txt_FL_HO043

HO043_StepstoEntrance
How many steps have to be climbed (up or down) to get to the main entrance of your flat?

IWER:
Do not include steps that are avoided, because the block has an elevator
NUMBER OF STEPS TO ENTRANCE
1. Up to 5
2. 6 to 15
3. 16 to 25
4. More than 25

Txt_FL_HO037

HO037_CityTown
Please look at card 35.
How would you describe the area where you live?

IWER:
{ReadOut}
AREA WHERE YOU LIVE
1. A big city
2. The suburbs or outskirts of a big city
3. A large town
4. A small town
5. A rural area or village

*ENDIF*Txt_FL_HO054

HO054_Elevator
Does your home have an elevator?
ELEVATOR
1. Yes
5. No

*ENDIF*Txt_FL_HO026

HO026_OwnSecHome

Do you [or/ or/ or/ or] [your/ your/ your/ your] [husband/ wife/ partner/ partner] own secondary homes, holiday homes, other real estate, land or forestry?

IWER:
Please do not include: time-sharing arrangement, own business
OWN SECONDARY HOMES ETC
1. Yes
5. No

IF HO026_OwnSecHome = a1
Txt_FL_HO027

HO027_ValueRE
In your opinion, how much would this or these properties be worth now if you sold it?

IWER:
If owns property abroad, give value in [FLDefault[9]]
VALUE OF REAL ESTATE
-100000000000000000..1000000000000000000
CHECK NOT ((HO027_ValueRE <= 0) AND (HO027_ValueRE = RESPONSE)) L1 " [Amount is expected to be higher than zero]"

IF HO027_ValueRE = NONRESPONSE
UB SEQUENCE UB_HO927

*ENDIF*Txt_FL_HO029

HO029_RecIncRe
Did you [or/ or/ or/ or] [your/ your/ your/ your] [husband/ wife/ partner/ partner] receive any income or rent from these properties in [STR (Year - 1)]?
RECEIVE INCOME OR RENT OF REAL ESTATE
1. Yes
5. No

IF HO029_RecIncRe = a1
Txt_FL_HO030

HO030_AmIncRe
How much income or rent did you [or/ or/ or/ or] [your/ your/ your/ your] [husband/ wife/ partner/ partner] receive from these properties during [STR (Year - 1)], after taxes?

IWER:
Amount in [FLDefault[9]]
AMOUNT INCOME OR RENT OF REAL ESTATE LAST YEAR
-100000000000000000..1000000000000000000
CHECK NOT ((HO030_AmIncRe = 0) AND (HO030_AmIncRe = RESPONSE)) L1 " [Amount is expected to be higher than zero]"

IF HO030_AmIncRe = NONRESPONSE
UB SEQUENCE UB_HO930

ENDIF

ENDIF

ENDIF

ENDIF

ENDIF

HO041_IntCheck

IWER:
CHECK:
Who answered the questions in this section?
WHO ANSWERED THE QUESTIONS IN HO
1. Respondent only
2. Respondent and proxy
3. Proxy only

System preset value HO902_TimeStampEnd
IF HO902_TimeStampEnd = EMPTY AND HO041_IntCheck <> EMPTY
ENDIF

ENDIF

ENDBLOCK

ENDIF
IF (HH IN Test) OR (ALL IN Test)

BLOCK

IF MN008_NumHHR = 1

IF MN024_NursingHome = a1
Txt_FL_HH001

HH001_OtherContribution
Although we may have asked you [or other members of your household] some of the details earlier, it is important for us to understand your household's situation correctly. In the last year, that is in [STR (Year - 1)], was there any household member who contributed to your household income and who is not part of this interview?

IWER:
If necessary read list of eligibles: part of this interview are {MN015_ELIGIBLES}
OTHER CONTRIBUTION TO HOUSEHOLD INCOME
1. Yes
5. No

System preset value HH901_TimeStampStart
IF HH901_TimeStampStart = EMPTY AND HH001_OtherContribution <> EMPTY
*ENDIF*Txt_FL_HH010

HH010_OtherIncome
Some households receive payments such as housing allowances, child benefits, poverty relief etc. Has your household or anyone in your household received any such payments in [STR (Year - 1)]?
INCOME FROM OTHER SOURCES
1. Yes
5. No

IF HH010_OtherIncome = a1
Txt_FL_HH011

HH011_TotAddHHinc
Please give us the approximate total amount of income from these benefits that you received as a household in [STR (Year - 1)], after taxes and contributions.

IWER:
Here the giver is the government or a local authority. Amount in [FLDefault[9]]
ADDITIONAL INCOME RECEIVED BY ALL HOUSEHOLD MEMBERS IN LAST YEAR

-1000000000000000000..1000000000000000000
CHECK NOT ((HH011_TotAddHHinc <= 0) AND (HH011_TotAddHHinc = RESPONSE)) L1 " [Amount is expected to be higher than zero]"

IF HH011_TotAddHHinc = NONRESPONSE
UB SEQUENCE UB_HH911

ENDIF

ENDIF

ENDIF

ENDIF
IF MN101_Longitudinal = 0
Txt_FL_HH021

HH021_Intro
Please look at card 23. I am now going to read some statements concerning the way you may feel about your local area, that is everywhere within a 20 minute walk or a kilometre of your home. Please tell me whether you strongly agree, agree, disagree or strongly disagree with each statement.
INTRODUCTION LOCAL AREA
1. Continue

Txt_FL_HH022

HH022_LocalFeelPart
I really feel part of this area. Would you say you strongly agree, agree, disagree or strongly disagree?

IWER:
Show card 23
LOCAL AREA FEEL PART
1. Strongly agree
2. Agree
3. Disagree
4. Strongly disagree

Txt_FL_HH023

HH023_LocalVandalism
Vandalism [m]or[/m] crime [m]is[/m] a big problem in this area. (Would you say you strongly agree, agree, disagree or strongly disagree?)

IWER:
Show card 23
LOCAL AREA VANDALISM
1. Strongly agree
2. Agree
3. Disagree
4. Strongly disagree

Txt_FL_HH024

HH024_LocalClean
This area is kept very clean. (Would you say you strongly agree, agree, disagree or strongly disagree?)

IWER:
Show card 23
LOCAL AREA CLEAN
1. Strongly agree
2. Agree
3. Disagree
4. Strongly disagree

Txt_FL_HH025

HH025_LocalPeopleHelpful
If I were in trouble, there are people in this area who would help me. (Would you say you strongly agree, agree, disagree or strongly disagree?)

IWER:
Show card 23
LOCAL AREA PEOPLE HELPFUL
1. Strongly agree
2. Agree
3. Disagree
4. Strongly disagree

ENDIF
IF ((MN008_NumHHR = 1) AND (MN024_NursingHome = a1)) OR (MN101_Longitudinal = 0)

HH014_IntCheck

IWER:
CHECK:
Who answered the questions in this section?
WHO ANSWERED THE QUESTIONS IN HH
1. Respondent only
2. Respondent and proxy
3. Proxy only

ENDIF
System preset value HH902_TimeStampEnd
IF HH902_TimeStampEnd = EMPTY AND HH014_IntCheck <> EMPTY
ENDIF

ENDBLOCK

ENDIF
IF (CO IN Test) OR (ALL IN Test)

BLOCK

IF MN008_NumHHR = 1

IF MN024_NursingHome = a1
Txt_FL_CO001

CO001_Intro1
We would now like to ask some questions about your household's usual expenditures and how your household is managing financially.
INTRODUCTION TEXT
1. Continue

System preset value CO901_TimeStampStart
IF CO901_TimeStampStart = EMPTY AND CO001_Intro1 <> EMPTY
*ENDIF*Txt_FL_CO002

CO002_ExpFoodAtHome
Thinking about the last 12 months:
about how much did your household spend in a typical month on food to be consumed at home?

IWER:
Amount in [FLDefault[9]]

AMOUNT SPENT ON FOOD AT HOME
-1000000000000000000..10000000000000000000
CHECK NOT ((CO002_ExpFoodAtHome <= 0) AND (CO002_ExpFoodAtHome = RESPONSE)) L1 " [Amount is expected to be higher than zero]"

IF CO002_ExpFoodAtHome = NONRESPONSE
UB SEQUENCE UB_CO902

*ENDIF*Txt_FL_CO003

CO003_ExpFoodOutsHme
Still thinking about the last 12 months:
about how much did your household spend in a typical month on food to be consumed outside home?

IWER:
Amount in [FLDefault[9]]
AMOUNT SPENT ON FOOD OUTSIDE THE HOME
-1000000000000000000..10000000000000000000

IF CO003_ExpFoodOutsHme = NONRESPONSE
UB SEQUENCE UB_CO903

*ENDIF*Txt_FL_CO010

CO010_HomeProducedFood
Do you [and other members of your household] consume vegetables, fruit or meat that you have grown, produced, caught or gathered yourselves?
CONSUME HOME PRODUCED FOOD
1. Yes
5. No

IF CO010_HomeProducedFood = a1
Txt_FL_CO011

CO011_ValHomeProducedFood
Thinking about the last 12 months, what is the value of the home produced food that you consumed in a typical month? In other words, how much would you have paid for this food if you had to buy it?

IWER:
Enter an amount in [FLDefault[9]]
VALUE OF HOME PRODUCED FOOD
-1000000000000000000..10000000000000000000

IF CO011_ValHomeProducedFood = NONRESPONSE
UB SEQUENCE UB_CO911

ENDIF

*ENDIF*Txt_FL_HH017

HH017_TotAvHHincMonth
How much was the overall income, after taxes and contributions, that your entire household had in an average month in [STR (Year - 1)]?

IWER:
Enter an amount in [FLDefault[9]]
TOTAL INCOME RECEIVED BY ALL HOUSEHOLD MEMBERS IN LAST MONTH
-1000000000000000000..10000000000000000000
CHECK NOT ((HH017_TotAvHHincMonth <= 0) AND (HH017_TotAvHHincMonth = RESPONSE)) L1 " [Amount is expected to be higher than zero]"

IF HH017_TotAvHHincMonth = NONRESPONSE
UB SEQUENCE UB_HH917

*ENDIF*Txt_FL_CO007

CO007_AbleMakeEndsMeet
Thinking of your household's total monthly income, would you say that your household is able to make ends meet...

IWER:
{ReadOut}
IS HOUSEHOLD ABLE TO MAKE ENDS MEET
1. With great difficulty
2. With some difficulty
3. Fairly easily
4. Easily

IF ((CO007_AbleMakeEndsMeet = a1) OR (CO007_AbleMakeEndsMeet = a2)) OR (CO007_AbleMakeEndsMeet = a3)
Txt_FL_CO020

CO020_HowMuchNeeded
Which minimum amount of money in total would your household need per month to easily make ends meet?

IWER:
Enter an amount in [FLDefault[9]]
HOW MUCH NEEDED TO MAKE ENDS MEET
-1000000000000000000..10000000000000000000

ENDIF
IF MN032_socex = 1
Txt_FL_CO206

CO206_AffordExpense
Could your household afford to pay an unexpected expense of {AffordExpenseAmount} [FLDefault[9]] without borrowing any money?
AFFORD EXPENSE
1. Yes
5. No

Txt_FL_CO209

CO209_PovertyPutUpWithCold
In the last twelve months, have you put up with feeling cold to save heating costs, to help you keep your living costs down?
PUT UP WITH COLD
1. Yes
5. No

ENDIF

CO009_IntCheck

IWER:
CHECK:
Who answered the questions in this section?
WHO ANSWERED THE QUESTIONS IN CO
1. Respondent only
2. Respondent and proxy
3. Proxy only

System preset value CO902_TimeStampEnd
IF CO902_TimeStampEnd = EMPTY AND CO009_IntCheck <> EMPTY
ENDIF

ENDIF

ENDIF

ENDBLOCK

ENDIF
IF (AS IN Test) OR (ALL IN Test)

BLOCK

IF MN007_NumFinR = 1
Txt_FL_AS001

AS001_Intro1
The next questions ask about a number of different kinds of savings or investments that you [or/ or/ or/ or] [your/ your/ your/ your] [husband/ wife/ partner/ partner] may have.
INTRODUCTION 1 TO ASSETS
1. Continue

System preset value AS901_TimeStampStart
IF AS901_TimeStampStart = EMPTY AND AS001_Intro1 <> EMPTY
*ENDIF*Txt_FL_AS065

AS065_HasIndRetAcc
Do you [or/ or/ or/ or] [your/ your/ your/ your] [husband/ wife/ partner/ partner] currently have any money in individual retirement accounts?

IWER:
An individual retirement account is a retirement plan that lets the person put some money away each year, to be (partially) taken out at retirement time.
HAS INDIVIDUAL RETIREMENT ACCOUNTS
1. Yes
5. No

IF AS065_HasIndRetAcc = a1

IF MN005_ModeQues <> a1
Txt_FL_AS020

AS020_IndRetAcc
Who has individual retirement accounts? You [, your/ , your/ , your/ , your] [husband/ wife/ partner/ partner] [or/ or/ or/ or] [both/ both/ both/ both]?
WHO HAS INDIVIDUAL RETIREMENT ACCOUNTS
1. Respondent only
2. ^FL_AS020_5 only
3. Both

ENDIF
IF (MN005_ModeQues = a1) OR ((AS020_IndRetAcc = a1) OR (AS020_IndRetAcc = a3))
Txt_FL_AS021

AS021_AmIndRet
How much do you currently have in individual retirement accounts?

IWER:
Enter an amount in [FLDefault[9]]; code amount for respondent only

AMOUNT INDIVIDUAL RETIREMENT ACCOUNTS
-100000000000000000..1000000000000000000
CHECK NOT ((AS021_AmIndRet <= 0) AND (AS021_AmIndRet = RESPONSE)) L1 " [Amount is expected to be higher than zero]"
CHECK AS021_AmIndRet <> EMPTY L1 " [Please enter a value]"

IF AS021_AmIndRet = NONRESPONSE
UB SEQUENCE UB_AS921

ENDIF

AS023_IndRetStockBo
Are these individual retirement accounts mostly in stocks or mostly in bonds?
INDIVIDUAL RETIREMENT ACCOUNTS MOSTLY IN STOCKS OR BONDS
1. Mostly stocks
2. Half stocks and half bonds
3. Mostly bonds

ENDIF
IF (AS020_IndRetAcc = a2) OR (AS020_IndRetAcc = a3)
Txt_FL_AS024

AS024_PAmIndRet
How much does [your/ your/ your/ your] [husband/ wife/ partner/ partner] currently have in individual retirement accounts?

IWER:
Amount in [FLDefault[9]]
Code amount for partner only
PARTNER AMOUNT INDIVIDUAL RETIREMENT ACCOUNTS
-100000000000000000..1000000000000000000
CHECK NOT ((AS024_PAmIndRet <= 0) AND (AS024_PAmIndRet = RESPONSE)) L1 " [Amount is expected to be higher than zero]"
CHECK AS024_PAmIndRet <> EMPTY L1 " [Please enter a value]"

IF AS024_PAmIndRet = NONRESPONSE
UB SEQUENCE UB_AS924

ENDIF

AS026_PIndRetStockBo
Are these individual retirement accounts mostly in stocks or mostly in bonds?
PARTNER INDIVIDUAL RETIREMENT ACCOUNTS MOSTLY IN STOCKS OR BONDS
1. Mostly stocks
2. Half stocks and half bonds
3. Mostly bonds

ENDIF

*ENDIF*Txt_FL_AS066

AS066_HasContSav
Do you [or/ or/ or/ or] [your/ your/ your/ your] [husband/ wife/ partner/ partner] currently have any money in contractual saving for housing?

IWER:
Contractual savings for housing: an account at a financial institution that accumulates cash to be used towards the purchase of a house.
HAS CONTRACTUAL SAVING
1. Yes
5. No

IF AS066_HasContSav = a1

Txt_FL_AS027

AS027_AmContSav

About how much do you [and/ and/ and/ and] [your/ your/ your/ your] [husband/ wife/ partner/ partner] currently have in contractual saving for housing?

IWER:

Enter an amount in [FLDefault[9]]; code total amount for both partners

AMOUNT CONTRACTUAL SAVING

-1000000000000000000..1000000000000000000

CHECK NOT ((AS027_AmContSav <= 0) AND (AS027_AmContSav = RESPONSE)) L1 " [Amount is expected to be higher than zero]"

CHECK AS027_AmContSav <> EMPTY L1 " [Please enter a value]"

IF AS027_AmContSav = NONRESPONSE

UB SEQUENCE UB_AS927

ENDIF

ENDIFTxt_FL_AS067

AS067_HasLifeIns

Do you [or/ or/ or/ or] [your/ your/ your/ your] [husband/ wife/ partner/ partner] currently own any life insurance policies?

HAS LIFE INSURANCE

1. Yes
5. No

IF AS067_HasLifeIns = a1

Txt_FL_AS029

AS029_LifeInsPol

Are your life insurance policies term policies, whole life policies, or both of these?

IWER:

Term life insurance provides coverage for a fixed period of time and pays a predetermined amount only if the policyholder dies within this period. On the other hand, whole life insurance has a savings component that increases in value over time and can be paid back in many installments over time or all at once.

LIFE INSURANCE POLICIES TERM OR WHOLE LIFE

1. Term policies
2. Whole life policies
3. Both
97. Other

IF (AS029_LifeInsPol = a2) OR (AS029_LifeInsPol = a3)

Txt_FL_AS030

AS030_ValLifePol

What is the face value of the whole life policies owned by you [and/ and/ and/ and] [your/ your/ your/ your] [husband/ wife/ partner/ partner]?

IWER:

Amount in [FLDefault[9]] ; code total amount for both partners

FACE VALUE LIFE POLICIES

-1000000000000000000..1000000000000000000

CHECK NOT ((AS030_ValLifePol <= 0) AND (AS030_ValLifePol = RESPONSE)) L1 " [Amount is expected to be higher than zero]"

IF AS030_ValLifePol = NONRESPONSE

UB SEQUENCE UB_AS930

ENDIF

ENDIF

*ENDIF*Txt_FL_AS064

AS064_HasMutFunds
Do you [or/ or/ or/ or] [your/ your/ your/ your] [husband/ wife/ partner/ partner] currently have any money in mutual funds or managed investment accounts?

IWER:
A mutual fund is a form of investment which is set up by a financial institution that collects money from many investors and gives it to a manager to invest it in stocks, bonds, and other financial products
HAS MUTUAL FUNDS
1. Yes
5. No

IF AS064_HasMutFunds = a1
Txt_FL_AS017

AS017_AmMutFunds
About how much do you [and/ and/ and/ and] [your/ your/ your/ your] [husband/ wife/ partner/ partner] currently have in mutual funds or managed investment accounts?

IWER:
Amount in [FLDefault[9]]; code total amount for both partners
AMOUNT IN MUTUAL FUNDS
-1000000000000000000..1000000000000000000
CHECK NOT ((AS017_AmMutFunds <= 0) AND (AS017_AmMutFunds = RESPONSE)) L1 " [Amount is expected to be higher than zero]"
CHECK AS017_AmMutFunds <> EMPTY L1 " [Please enter a value]"

IF AS017_AmMutFunds = NONRESPONSE
UB SEQUENCE UB_AS917

ENDIF

AS019_MuFuStockBo
Are these mutual funds and managed investment accounts mostly stocks or mostly bonds?
MUTUAL FUNDS MOSTLY STOCKS OR BONDS
1. Mostly stocks
2. Half stocks and half bonds
3. Mostly bonds

*ENDIF*Txt_FL_AS063

AS063_HasStocks
Do you [or/ or/ or/ or] [your/ your/ your/ your] [husband/ wife/ partner/ partner] currently have any money in stocks or shares that are listed or unlisted on stockmarket?

IWER:
Stocks are a form of investment that allows a person to own a part of a corporation and gives him/her the right to receive dividends from it.
HAS STOCKS
1. Yes
5. No

IF AS063_HasStocks = a1
Txt_FL_AS011

AS011_AmStocks
About how much do you [and/ and/ and/ and] [your/ your/ your/ your] [husband/ wife/ partner/ partner] currently have in stocks or shares that are listed or unlisted on stock market?

IWER:
amount in [FLDefault[9]]; Code total amount for both partners
AMOUNT IN STOCKS
-1000000000000000000..1000000000000000000
CHECK NOT ((AS011_AmStocks <= 0) AND (AS011_AmStocks = RESPONSE)) L1 " [Amount is expected to be higher than zero]"
CHECK AS011_AmStocks <> EMPTY L1 " [Please enter a value]"

IF AS011_AmStocks = NONRESPONSE
UB SEQUENCE UB_AS911

ENDIF

*ENDIF*Txt_FL_AS062

AS062_HasBonds
Do you [or/ or/ or/ or] [your/ your/ your/ your] [husband/ wife/ partner/ partner] currently have any money in government or corporate bonds?

IWER:
Bonds are a debt instrument issued by the government or a corporation in order to generate capital by borrowing.
HAS BONDS
1. Yes
5. No

IF AS062_HasBonds = a1
Txt_FL_AS007

AS007_AmBonds
About how much do you currently [and/ and/ and/ and] [your/ your/ your/ your] [husband/ wife/ partner/ partner] have in government or corporate bonds?

IWER:
Enter an amount in [FLDefault[9]]; code total amount for both partners
AMOUNT IN BONDS
-1000000000000000000..1000000000000000000
CHECK NOT ((AS007_AmBonds <= 0) AND (AS007_AmBonds = RESPONSE)) L1 " [Amount is expected to be higher than zero]"
CHECK NOT (AS007_AmBonds = EMPTY AND AS007_AmBonds <> NONRESPONSE) L1 " [Please enter a value]"

IF AS007_AmBonds = NONRESPONSE
UB SEQUENCE UB_AS907

ENDIF

*ENDIF*Txt_FL_AS060

AS060_HasBankAcc
Do you [or/ or/ or/ or] [your/ your/ your/ your] [husband/ wife/ partner/ partner] currently have a bank account, or transaction account, or saving account or postal account?
HAS BANK ACCOUNT
1. Yes
5. No

IF AS060_HasBankAcc = a1
Txt_FL_AS003

AS003_AmBankAcc
About how much do you [and/ and/ and/ and] [your/ your/ your/ your] [husband/ wife/ partner/ partner] currently have in bank accounts, transaction accounts, saving accounts or postal accounts?

IWER:
amount in [FLDefault[9]]; Code total amount for both partners
AMOUNT BANK ACCOUNT
-100000000000000000..1000000000000000000
CHECK AS003_AmBankAcc <> EMPTY L1 " [Please enter a value]"

IF AS003_AmBankAcc = NONRESPONSE
UB SEQUENCE UB_AS903

ENDIF

ENDIF
IF (((AS060_HasBankAcc = a1) OR (AS062_HasBonds = a1)) OR (AS063_HasStocks = a1)) OR (AS064_HasMutFunds = a1)
Txt_FL_AS070

AS070_IntIncome
Overall, about how much interest or dividend income did you [and/ and/ and/ and] [your/ your/ your/ your] [husband/ wife/ partner/ partner] receive from your savings in bank accounts, bonds, stocks or mutual funds in [STR (Year - 1)]? Please give me the amount after taxes.

IWER:
Enter an amount in [FLDefault[9]]
INTEREST OR DIVIDEND
-100000000000000000..1000000000000000000
CHECK AS070_IntIncome <> EMPTY L1 " [Please enter a value]"

IF AS070_IntIncome = NONRESPONSE
UB SEQUENCE UB_AS970

ENDIF

*ENDIF*Txt_FL_AS641

AS641_OwnFirm
Do you [or/ or/ or/ or] [your/ your/ your/ your] [husband/ wife/ partner/ partner] currently own a firm, company, or business either entirely or as a partial ownership?
OWN FIRM COMPANY BUSINESS
1. Yes
5. No

IF AS641_OwnFirm = a1
Txt_FL_AS044

AS044_ShareFirm
What percentage or share of this firm, company or business is owned by you [or/ or/ or/ or] [your/ your/ your/ your] [husband/ wife/ partner/ partner]?

IWER:
Enter percent. If less then 1 percent, type 1.
PERCENTAGE SHARE FIRM OWNED
1..100
CHECK AS044_ShareFirm <= 100 L1 " [Percentage should be less or equal to 100]"

IF AS044_ShareFirm = NONRESPONSE
UB SEQUENCE UB_AS944

*ENDIF*Txt_FL_AS642

AS642_AmSellFirm
If the firm, company or business was sold and then paid off any debts on it, how much money would be left for you [or/ or/ or/ or] [your/ your/ your/ your] [husband/ wife/ partner/ partner]?

IWER:
Amount in [FLDefault[9]] ; code total amount for both partners
AMOUNT SELLING FIRM
-100000000000000000..1000000000000000000
CHECK NOT ((AS642_AmSellFirm = 0) AND (AS642_AmSellFirm = RESPONSE)) L1 " [Amount is expected to be lower or higher than zero]"
CHECK AS642_AmSellFirm <> EMPTY L1 " [Please enter a value]"

IF AS642_AmSellFirm = NONRESPONSE
UB SEQUENCE UB_AS942

ENDIF

*ENDIF*Txt_FL_AS649

AS649_NumCars
How many cars do you [or/ or/ or/ or] [your/ your/ your/ your] [husband/ wife/ partner/ partner] own? Please exclude company cars and leased cars.
NUMBER OF CARS
0..10

IF AS649_NumCars > 0
Txt_FL_AS051

AS051_AmSellingCars
If you sold [this/ these] [car/ cars] about how much would you get?

IWER:
amount in [FLDefault[9]]; Code total amount for both partners
AMOUNT SELLING CARS
-100000000000000000..1000000000000000000
CHECK NOT ((AS051_AmSellingCars = 0) AND (AS051_AmSellingCars = RESPONSE)) L1 " [Amount is expected to be lower or higher than zero]"
CHECK AS051_AmSellingCars <> EMPTY L1 " [Please enter a value]"

IF AS051_AmSellingCars = NONRESPONSE
UB SEQUENCE UB_AS951

ENDIF

*ENDIF*Txt_FL_AS054

AS054_OweMonAny
The next question refers to money that you may owe, excluding mortgages or money owed on land, property or firms (if any). Looking at card 36, which of these types of debts do you [or/ or/ or/ or] [your/ your/ your/ your] [husband/ wife/ partner/ partner] currently have, if any?

IWER:
{CodeAll}
OWE MONEY
1. Debt on cars and other vehicles (vans/motorcycles/boats, etc.)
2. Debt on credit cards / store cards
3. Loans (from bank, building society or other financial institution)
4. Debts to relatives or friends
5. Student loans
6. Overdue bills (phone, electricity, heating, rent)
96. None of these
97. Other

CHECK NOT ((AS054_OweMonAny.CARDINAL > 1) AND (96 IN AS054_OweMonAny)) L1 " [You cannot select ""None of the above"" together with any other answer. Please change your answer]"

IF NOT (96 IN AS054_OweMonAny)

Txt_FL_AS055

AS055_AmOweMon

Not including mortgages or money owed on land, property or firms, how much do you [and/ and/ and/ and] [your/ your/ your/ your] [husband/ wife/ partner/ partner] owe in total?

IWER:

amount in [FLDefault[9]]; Code total amount for both partners

AMOUNT OWING MONEY IN TOTAL

-1000000000000000000..1000000000000000000

CHECK NOT ((AS055_AmOweMon = 0) AND (AS055_AmOweMon = RESPONSE)) L1 " [Amount is expected to be lower or higher than zero]"

CHECK AS055_AmOweMon <> EMPTY L1 " [Please enter a value]"

IF AS055_AmOweMon = NONRESPONSE

UB SEQUENCE UB_AS955

ENDIF

ENDIF

AS057_IntCheck

IWER:

CHECK:

Who answered the questions in this section?

WHO ANSWERED THE QUESTIONS IN AS

1. Respondent only
2. Respondent and proxy
3. Proxy only

System preset value AS902_TimeStampEnd

IF AS902_TimeStampEnd = EMPTY AND AS057_IntCheck <> EMPTY

ENDIF

ENDIF

ENDBLOCK

ENDIF

IF (AC IN Test) OR (ALL IN Test)

BLOCK

AC011_Intro

We are also interested in how people think about their lives in general.

IWER:

Start of a @BNon-proxy section@B. No proxy allowed. If the respondent is not capable of answering any of these questions on her/his own, please select 5.

INTRODUCTION WELL-BEING

1. Continue
5. Proxy-interview

System preset value AC901_TimeStampStart

System preset value AC902_TimeStampEnd

IF AC901_TimeStampStart = EMPTY AND AC011_Intro <> EMPTY

ENDIF

IF (AC011_Intro = RESPONSE) AND NOT (AC011_Intro = a5)

Txt_FL_AC012

AC012_HowSat

On a scale from 0 to 10 where 0 means completely dissatisfied and 10 means completely satisfied, how satisfied are you with your life?

HOW SATISFIED WITH LIFE

0..10

Txt_FL_AC013

AC013_Intro

Please look at card 37. I will now read a list of statements that people have used to describe their lives or how they feel. We would like to know how often, if at all, you experienced the following feelings and thoughts: often, sometimes, rarely, or never.

INTRODUCTION CASP ITEMS

1. Continue

Txt_FL_AC014

AC014_AgePrev

How often do you think your age prevents you from doing the things you would like to do?

IWER:

Card 37. {ReadOut}

AGE PREVENTS FROM DOING THINGS

1. Often
2. Sometimes
3. Rarely
4. Never

Txt_FL_AC015

AC015_OutofContr

How often do you feel that what happens to you is out of your control?

IWER:

Card 37. {ReadOutNeed}

OUT OF CONTROL

1. Often
2. Sometimes
3. Rarely
4. Never

Txt_FL_AC016

AC016_LeftOut

How often do you feel left out of things?

IWER:

Card 37. {ReadOutNeed}

FEEL LEFT OUT OF THINGS

1. Often
2. Sometimes
3. Rarely
4. Never

Txt_FL_AC017

AC017_DoWant

How often do you think that you can do the things that you want to do?

IWER:

Card 37. {ReadOutNeed}

DO THE THINGS YOU WANT TO DO
1. Often
2. Sometimes
3. Rarely
4. Never

Txt_FL_AC018

AC018_FamRespPrev
How often do you think that family responsibilities prevent you from doing what you want to do?

IWER:
Card 37. {ReadOutNeed}
FAMILY RESPONSIBILITIES PREVENT
1. Often
2. Sometimes
3. Rarely
4. Never

Txt_FL_AC019

AC019_ShortMon
How often do you think that shortage of money stops you from doing the things you want to do?

IWER:
Card 37. {ReadOutNeed}
SHORTAGE OF MONEY STOPS
1. Often
2. Sometimes
3. Rarely
4. Never

Txt_FL_AC020

AC020_EachDay
How often do you look forward to each day?

IWER:
Card 37. {ReadOutNeed}
LOOK FORWARD TO EACH DAY
1. Often
2. Sometimes
3. Rarely
4. Never

Txt_FL_AC021

AC021_LifeMean
How often do you feel that your life has meaning?

IWER:
Card 37. {ReadOutNeed}
LIFE HAS MEANING
1. Often
2. Sometimes
3. Rarely
4. Never

Txt_FL_AC022

AC022_BackHapp
How often, on balance, do you look back on your life with a sense of happiness?

IWER:

Card 37. {ReadOutNeed}
LOOK BACK ON LIFE WITH HAPPINESS
1. Often
2. Sometimes
3. Rarely
4. Never

Txt_FL_AC023

AC023_FullEnerg
How often do you feel full of energy these days?

IWER:
Card 37. {ReadOutNeed}
FEEL FULL OF ENERGY
1. Often
2. Sometimes
3. Rarely
4. Never

Txt_FL_AC024

AC024_FullOpport
How often do you feel that life is full of opportunities?

IWER:
Card 37. {ReadOutNeed}
FULL OF OPPORTUNITIES
1. Often
2. Sometimes
3. Rarely
4. Never

Txt_FL_AC025

AC025_FutuGood
How often do you feel that the future looks good for you?

IWER:
Card 37. {ReadOutNeed}
FUTURE LOOKS GOOD
1. Often
2. Sometimes
3. Rarely
4. Never

IF MN024_NursingHome = a1

Txt_FL_AC001

AC001_Intro
Now I have a few questions about activities you may do.
INTRODUCTION AC ACTIVITIES
1. Continue

Txt_FL_AC035

AC035_ActPastTwelveMonths
Please look at card 38: which of the activities listed on this card - if any - have you done in the last twelve months?

IWER:
{CodeAll}
ACTIVITIES IN LAST YEAR

1. Done voluntary or charity work
4. Attended an educational or training course
5. Gone to a sport, social or other kind of club
7. Taken part in a political or community-related organization
8. Read books, magazines or newspapers
9. Did word or number games such as crossword puzzles or Sudoku
10. Played cards or games such as chess.
96. None of these

CHECK NOT ((AC035_ActPastTwelveMonths.CARDINAL > 1) AND (a96 IN AC035_ActPastTwelveMonths)) L1 " [You cannot select ""None of the above"" together with any other answer. Please change your answer]"

IF a96 IN AC035_ActPastTwelveMonths

Txt_FL_AC038

AC038_HowSatisfiedNoAct
You indicated that you do not engage in any of the activities on Card 38. On a scale from 0 to 10 where 0 means completely dissatisfied and 10 means completely satisfied, how satisfied are you with this?
SATISFIED WITH NO ACTIVITIES
0..10

ELSE

LOOP cnt1:= 1 TO 10

IF cnt1 IN AC035_ActPastTwelveMonths

BLOCK

Txt_FL_AC036

AC036_HowOftAct
How often in the past twelve months [did/ have/ did/ have/ did/ did/ did] [you/ you/ you/ you/ you/ you/ you] [do voluntary or charity work/ attended an educational or training course/ go to a sport, social or other kind of club/ taken part in a political or community-related organization/ read books, magazines or newspapers/ do word or number games such as crossword puzzles or Sudoku/ played cards or games such as chess.]?

IWER:
{ReadOut}.
HOW OFTEN ACTIVITY IN THE LAST TWELVE MONTHS
1. Almost daily
2. Almost every week
3. Almost every month
4. Less often

ENDBLOCK

ENDIF

*ENDLOOP*Txt_FL_AC037

AC037_HowSatisfied
On a scale from 0 to 10 where 0 means completely dissatisfied and 10 means completely satisfied, how satisfied are you with the activities that you mentioned?
SATISFIED WITH ACTIVITIES
0..10

ENDIF
IF AC902_TimeStampEnd = EMPTY AND (AC037_HowSatisfied <> EMPTY OR AC038_HowSatisfiedNoAct <> EMPTY)
ENDIF

ELSE

IF AC902_TimeStampEnd = EMPTY AND AC025_FutuGood <> EMPTY
ENDIF

ENDIF

ENDIF

ENDBLOCK

ENDIF
IF (EX IN Test) OR (ALL IN Test)

BLOCK

System preset value EX901_TimeStampStart
IF EX901_TimeStampStart = EMPTY
ENDIF

EX601_NonProxy

IWER:
Start of a @BNon-proxy section@B. No proxy allowed. If the respondent is not present or not capable to give consent to participation on her/his own, please select '5'.
INTRO EX_PROXY
1. Continue
5. Proxy-interview

IF (EX601_NonProxy = RESPONSE) AND NOT (EX601_NonProxy = a5)

IF MN101_Longitudinal = 0
Txt_FL_EX029

EX029_FreqPrayer
Now, I have a question about praying. These days, how often do you pray?

IWER:
{ReadOut}
PRAYING
1. More than once a day
2. Once daily
3. A couple of times a week
4. Once a week
5. Less than once a week
6. Never

ENDIFTxt_FL_EX001

EX001_Introtxt
Now, I have questions about how likely you think various events might be. When I ask a question I'd like for you to give me a number from 0 to 100.

Let's try an example together and start with the weather. Looking at card 39, what do you think the chances are that it will be sunny tomorrow? For example, '90' would mean a 90 per cent chance of sunny weather. You can say any number from 0 to 100.
INTRODUCTION AND EXAMPLE
0..100

IF MN101_Longitudinal = 0

IF Sec_EP.EP005_CurrentJobSit = a2

Txt_FL_EX007

EX007_GovRedPens
(Please look at card 39.)
What are the chances that before you retire the government will reduce the pension which you are entitled to?
GOVERNMENT REDUCES PENSION
0..100

IF MN808_AgeRespondent < 61
Txt_FL_EX025

EX025_ChWrkA65
(Please look at card 39.)
Thinking about your work generally and not just your present job, what are the chances that you will be working full-time after you reach age 63?
CHANCE TO WORK AFTER AGE OF 63
0..100

*ENDIF*Txt_FL_EX008

EX008_GovRaisAge
(Please look at card 39.)
What are the chances that before you retire the government will raise your retirement age?
GOVERNMENT RAISES RETIREMENT AGE
0..100

ENDIF

ENDIF
IF MN808_AgeRespondent < 101
Txt_FL_EX009

EX009_LivTenYrs
(Please look at card 39.)
What are the chances that you will live to be age [{Current age rounded up to 5 fold}] or more?
LIVING IN TEN YEARS
0..100

ENDIF
IF MN101_Longitudinal = 0
Txt_FL_EX026

EX026_Trust
Now I would like to ask a question about how you view other people. Generally speaking, would you say that most people
can be trusted or that you can't be too careful in dealing with people? Not looking at card 39 anymore, please tell me on a scale from 0 to 10, where 0 means you can't be too careful and 10 means that most people can be trusted.
TRUST IN OTHER PEOPLE
0..10
Txt_FL_EX110

EX110_RiskAv
Please look at card 40. When people invest their savings they can choose between assets that give low return with little risk to lose money, for instance a bank account or a safe bond, or assets with a high return but also a higher risk of losing money, for instance stocks and shares. Which of the statements on the card comes closest to the amount of financial risk that you are willing to take when you save or make investments?

IWER:
Read answers only if necessary. If more than one response is given use the first category that applies.
RISK AVERSION
1. Take substantial financial risks expecting to earn substantial returns

2. Take above average financial risks expecting to earn above average returns
3. Take average financial risks expecting to earn average returns
4. Not willing to take any financial risks

Txt_FL_EX111

EX111_XYZ_Planning_Horizon
In planning your saving and spending, which of the following time periods is most important to you?

IWER:
{ReadOut}
The option 'next few months' includes also 'next few days' and 'next few weeks'
PLANNING HORIZON
1. Next few months
2. Next year
3. Next few years
4. Next 5-10 years
5. Longer than 10 years

Txt_FL_EX028

EX028_LeftRight
In politics people sometimes talk of left and right. On a scale from 0 to 10, where 0 means the left and 10 means the right, where would you place yourself?
LEFT OR RIGHT IN POLITICS
0..10

ENDIF

ENDIF
IF MN101_Longitudinal = 0

IF MN005_ModeQues = a2

EX600_PartInterv

IWER:
Is the respondent's partner available and willing to be interviewed in this session or are you doing a proxy interview for the partner in this session?
If the partner or proxy refuses to conduct an interview, please set a refusal code for the partner or proxy in the SMS after finishing the CAPI.
PARTNER AVAILABLE AND WILLING TO PARTICIPATE
1. Yes, partner is available and willing to be (proxy) interviewed in this session
2. No, partner is unavailable to be (proxy) interviewed in this session
3. No, partner is unwilling to be (proxy) interviewed in this session

IF (EX600_PartInterv = a2) OR (EX600_PartInterv = a3)
Txt_FL_EX101

EX101_IntroPartInfo
Before we finish, could you please also give me some information on [your/ your] [husband/ wife/ partner/ partner], who is not doing the interview today?
INTRODUCTION PARTNER INFORMATION
1. Continue

Txt_FL_EX602

EX602_PartYrsEduc
How many years has [your/ your/ your/ your] [husband/ wife/ partner/ partner] been in school all together?

IWER:
"in school" means in "full-time education", that;

* includes: receiving tuition, engaging in practical work or supervised study or taking examinations
* excludes: full-time working, home schooling, distance learning, special on-the-job training, evening classes, part-time private vocational training, flexible or part-time higher education studies, etc
PARTNER YEARS OF EDUCATION
0..21
Txt_FL_EX603

EX603_PartJobSit
Please look at card 7.
In general, how would you describe the current employment situation of [your/ your] [husband/ wife/ partner/ partner]?

IWER:
{ReadOut}
PARTNER CURRENT JOB SITUATION
1. Retired
2. Employed or self-employed (including working for family business)
3. Unemployed
4. Permanently sick or disabled
5. Homemaker
97. Other

IF EX603_PartJobSit = a2
Txt_FL_EX603

EX603_LastJobPartner
What is the most recent job [your/ your] [husband/ wife/ partner/ partner] {FL_EX603_3} had?
NAME OR TITLE OF JOB PARTNER
STRING

IF EX603_LastJobPartner = RESPONSE

BLOCK

JobCode

STRING

ENDBLOCK

ENDIF

ENDIF
IF (EX603_PartJobSit <> a1) AND (EX603_PartJobSit <> a2)
Txt_FL_EX104

EX104_PartEvWork
Has [your/ your/ your/ your] [husband/ wife/ partner/ partner] ever done any paid work?
PARTNER EVER DONE PAID WORK
1. Yes
5. No

ENDIF
IF ((EX603_PartJobSit = a1) OR (EX603_PartJobSit = a2)) OR (EX104_PartEvWork = a1)
Txt_FL_EX105

EX105_PartEmp
In [his/ her] [last/ current/ last] job, [was/ is/ was] [your/ your/ your/ your] [husband/ wife/ partner/ partner] a private sector employee, a public sector employee or a self-employed?
PARTNER EMPLOYEE OR A SELF-EMPLOYED
1. Private sector employee

2. Public sector employee
3. Self-employed

ENDIF

ENDIF

ENDIF

ENDIF
IF (MN001_Country = a1) OR (((MN001_Country = a7) OR (MN001_Country = a3)) AND (MN101_Longitudinal = 0))

EX123_Consent
It has not been decided yet but we are thinking about continuing this research project in one or two years with another, much shorter interview. For this reason, we hope that it is ok with you that we keep your name and address in our files, so that we can contact you again. Is this ok?

IWER:
Let respondent sign consent statement if necessary. If the respondent asks or hesitates, say that he/she can still say no at the time when recontacting
CONSENT TO RECONTACT
1. Consent to recontact
5. No consent to recontact

*ENDIF*Txt_FL_EX024

EX024_Outro2
Thank you. This was the last question. We would like to thank you very much again for participating in our research project. We know it has been a long and difficult questionnaire, but your help was really important. With your participation you have helped researchers to understand how the ageing of populations in Europe affects our future.
THANK YOU FOR PARTICIPATION
1. Continue

IF (((MN028_bio = 1) OR (MN028_bio = 2)) OR (MN028_bio = 3)) OR (MN028_bio = 4)

EX121_Temp

IWER:
Enter the approximate outside temperature in degree Celsius.
OUTSIDE TEMPERATURE
-30..60

EX122_EndDBSInstruction

IWER:
Take 'DBS Interviewer Short Instructions' and follow instructions 16 to the end.
END DBS INSTRUCTION
1. Continue

ENDIF

EX106_HandOutA

IWER:
Take a drop-off questionnaire and fill in first name and respondent id @B {RespondentID}@B on the drop-off

cover.
Enter drop-off serial number from drop-off questionnaire to CAPI below.
Hand out drop-off questionnaire to respondent.
HAND OUT DROP-OFF QUESTIONNAIRE
STRING

System preset value EX902_TimeStampEnd
IF EX902_TimeStampEnd = EMPTY AND EX106_HandOutA <> EMPTY
ENDIF

ENDBLOCK

ENDIF
IF (IV IN Test) OR (ALL IN Test)

BLOCK

IV001_Intro

IWER:
THIS SECTION IS ABOUT YOUR OBSERVATIONS DURING THE INTERVIEW AND SHOULD BE FILLED OUT AFTER EACH COMPLETED INDIVIDUAL INTERVIEW.
INTRODUCTION TO IV
1. Continue

System preset value IV901_TimeStampStart
IF IV901_TimeStampStart = EMPTY AND IV001_Intro <> EMPTY
ENDIF
IF ((((((((((((((((((((Sec_DN1.DN038_IntCheck = a2) OR (Sec_DN1.DN038_IntCheck = a3)) OR (Sec_PH.PH054_IntCheck = a2)) OR (Sec_PH.PH054_IntCheck = a3)) OR (Sec_BR.BR017_IntCheck = a2)) OR (Sec_BR.BR017_IntCheck = a3)) OR (Sec_EP.EP210_IntCheck = a2)) OR (Sec_EP.EP210_IntCheck = a3)) OR (Sec_CH.CH023_IntCheck = a2)) OR (Sec_CH.CH023_IntCheck = a3)) OR (Sec_SP.SP022_IntCheck = a2)) OR (Sec_SP.SP022_IntCheck = a3)) OR (Sec_FT.FT021_IntCheck = a2)) OR (Sec_FT.FT021_IntCheck = a3)) OR (Sec_HO.HO041_IntCheck = a2)) OR (Sec_HO.HO041_IntCheck = a3)) OR (Sec_HH.HH014_IntCheck = a2)) OR (Sec_HH.HH014_IntCheck = a3)) OR (Sec_CO.CO009_IntCheck = a2)) OR (Sec_CO.CO009_IntCheck = a3)) OR (Sec_AS.AS057_IntCheck = a2)) OR (Sec_AS.AS057_IntCheck = a3)

IV020_RelProxy
A proxy respondent has answered some or all of the questions we had for {FLRespondentName}. How is the proxy respondent related to {FLRespondentName}?
RELATIONSHIP PROXY
1. Spouse/Partner
2. Child/child-in-law
3. Parent/ Parent-in-law
4. Sibling
5. Grand-child
6. Other relative
7. Nursing home staff
8. Home helper
9. Friend/acquaintance
10. Other

ENDIF

IV002_PersPresent
Were any third persons, except proxy respondent, present during (parts of) the interview with {FLRespondentName}?

IWER:
{CodeAll}
THIRD PERSONS PRESENT

1. Nobody
2. Spouse or partner
3. Parent or parents
4. Child or children
5. Other relatives
6. Other persons present

CHECK NOT ((IV002_PersPresent.CARDINAL > 1) AND (1 IN IV002_PersPresent)) L1 " [You cannot select ""Nobody"" together with any other answer. Please change your answer.]"

IF NOT ((a1 IN IV002_PersPresent) AND (IV002_PersPresent.CARDINAL = 1))

IV003_PersIntervened
Have these persons intervened in the interview?
INTERVENED IN INTERVIEW
1. Yes, often
2. Yes, occasionally
3. No

ENDIF

IV004_WillingAnswer
How would you describe the willingness of {FLRespondentName} to answer?
WILLINGNESS TO ANSWER
1. Very good
2. Good
3. Fair
4. Bad
5. Good in the beginning, got worse during the interview
6. Bad in the beginning, got better during the interview

IF IV004_WillingAnswer = a5

IV005_WillingnessWorse
Why did the respondent's willingness to answer get worse during the interview?

IWER:
{CodeAll}
WHY WILLINGNESS WORSE
1. The respondent was losing interest
2. The respondent was losing concentration or was getting tired
3. Other, please specify

IF a3 IN IV005_WillingnessWorse

IV006_OthReason
Which other reason?
WHICH OTHER REASON
STRING

ENDIF

ENDIF

IV007_AskClarification
Did {FLRespondentName} ask for clarification on any questions?
RESP. ASK FOR CLARIFICATION
1. Never
2. Almost never
3. Now and then
4. Often

5. Very often
6. Always

IV008_RespUnderstoodQst
Overall, did you feel that {FLRespondentName} understood the questions?
RESPONDENT UNDERSTOOD QUESTIONS
1. Never
2. Almost never
3. Now and then
4. Often
5. Very often
6. Always

IV018_HelpShowcards
Did the respondent need any help reading the showcards during the interview?
HELP NEEDED READING SHOWCARDS
1. Yes, due to sight problems
2. Yes, due to literacy problems
3. No

IF MN008_NumHHR = 1

IF (Sec_HO.HO001_Place = a1) OR (MN024_NursingHome = a2)

IV009_AreaLocationBldg
In which type of area is the building located?
WHICH AREA BUILDING LOCATED
1. A big city
2. The suburbs or outskirts of a big city
3. A large town
4. A small town
5. A rural area or village

IV610_TypeBuilding
Which type of building does the household live in?

IWER:
A nursing home provides all of the following services for its residents: dispensing of medication, available, 24-hour personal assistance and supervision (not necessarily a nurse), and room & meals
TYPE OF BUILDING
1. A farm house
2. A free standing one or two family house
3. A one or two family house as row or double house
4. A building with 3 to 8 flats
5. A building with 9 or more flats but no more than 8 floors
6. A high-rise with 9 or more floors
7. A housing complex with services for older people (residential home or sheltered housing, but not a nursing home)
8. A nursing home

IV012_StepstoEntrance
How many steps had to be climbed (up or down) to get to the main entrance of the household's flat?

IWER:
Do not include steps that are avoided, because the block has an elevator
NUMBER OF STEPS TO ENTRANCE
1. Up to 5
2. 6 to 15
3. 16 to 25

4. More than 25

ENDIF

ENDIF

IV019_InterviewerID
Your interviewer ID:
INTERVIEWER ID
STRING
CHECK NOT ((IV019_InterviewerID = '') OR NOT (IV019_InterviewerID = RESPONSE)) L1 " [Please enter a value]"

IV017_Outro
Thank you very much for completing this section.
OUTRA IV
1. Continue

System preset value IV902_TimeStampEnd
IF IV902_TimeStampEnd = EMPTY AND IV017_Outro <> EMPTY
ENDIF

ENDBLOCK

ENDIF
IF XT_active = 1

BLOCK

IF ((((MN001_Country = a11) OR (MN001_Country = a14)) OR (MN001_Country = a19)) OR (MN001_Country = a21)) OR (MN001_Country = a23)

XT601_Language

CHOOSE LANGUAGE
English (Generic)
MEDIA

ENDIF

XT104_SexDec

IWER:
note sex of decendent (ask if unsure)
SEX OF DECENDENT
1. Male
2. Female

Txt_FL_XT001

XT001_Intro
[{Name of the deceased}] has participated in the SHARE study before [his/ her] death. [His/ Her] contribution was very valuable. We would find it extremely helpful to have some information about the final year of [{Name of the deceased}]'s life. All the information collected is strictly confidential, and will be held anonymously.
INTRODUCTION TO EXIT INTERVIEW
1. Continue

System preset value XT901_TimeStampStart
IF XT901_TimeStampStart = EMPTY AND XT001_Intro <> EMPTY

ENDIF

XT006_ProxSex

IWER:
Code proxy respondent's sex.
PROXY RESPONDENT'S SEX
1. Male
2. Female

Txt_FL_XT002

XT002_Relation
Before we start asking questions about the last year of life of [{Name of the deceased}], would you please tell me what was your relationship to the deceased?

IWER:
If unclear, specify: "So you were [his/ her]..."
RELATIONSHIP TO THE DECEASED
1. Husband or wife or partner
2. Son or Daughter
3. Son- or Daughter-in-law
4. Son or Daughter of husband, wife or partner
5. Grandchild
6. Sibling
7. Other relative (specify)
8. Other non-relative (specify)

IF XT002_Relation = a7

XT003_OthRel

IWER:
Specify other relative
OTHER RELATIVE
STRING

ENDIF
IF XT002_Relation = a8

XT004_OthNonRel

IWER:
Specify other non-relative
OTHER NO-RELATIVE
STRING

*ENDIF*Txt_FL_XT005

XT005_HowOftCont
During the last twelve months of [his/ her] life, how often did you have contact with [{Name of the deceased}], either in person, by phone, mail, email, or any other electronic means?
HOW OFTEN CONTACT LAST TWELVE MONTHS
1. Daily
2. Several times a week
3. About once a week
4. About every two weeks
5. About once a month
6. Less than once a month
7. Never

IF XT002_Relation <> a1

Txt_FL_XT007

XT007_YearBirth

Can you tell me your year of birth?

YEAR OF BIRTH PROXY

1900..1999

*ENDIF*Txt_FL_XT101

XT101_ConfDecYrBirth

Let us now talk about the deceased. Just to make sure that we have the correct information about [{Name of the deceased}], can I just confirm that [he/ she] was born in [{Month and Year birth of deceased}]?

CONFIRMATION DECEASED YEAR OF BIRTH

1. Yes
5. No

IF XT101_ConfDecYrBirth = a5

Txt_FL_XT102

XT102_DecMonthBirth

In which month and year was [{Name of the deceased}] born?

@bMONTH@b:

YEAR:

IWER:

Month

DECEASED MONTH OF BIRTH

1. January
2. February
3. March
4. April
5. May
6. June
7. July
8. August
9. September
10. October
11. November
12. December

Txt_FL_XT103

XT103_DecYearBirth

In which month and year was [he/ she] born?

MONTH: {XT102_DecMonthBirth}

@bYEAR@b:

IWER:

Year

DECEASED YEAR OF BIRTH

1900..2008

*ENDIF*Txt_FL_XT008

XT008_MonthDied

We would like to know more about the circumstances of [{Name of the deceased}] 's death. In what @bmonth@b and year did [he/ she] pass away?

@bMONTH@b:
YEAR:

IWER:
Month
MONTH OF DECEASE
1. January
2. February
3. March
4. April
5. May
6. June
7. July
8. August
9. September
10. October
11. November
12. December

Txt_FL_XT009

XT009_YearDied
In what month and @bYEAR@b did [he/ she] pass away?

MONTH: {XT008_MonthDied}

@bYEAR@b:

IWER:
Year
YEAR OF DECEASE
1. 2006
2. 2007
3. 2008
4. 2009
5. 2010
6. 2011
7. 2012
8. 2013
9. 2014
10. 2015

Txt_FL_XT010

XT010_AgeDied
How old was [{Name of the deceased}] when [he/ she] passed away?

IWER:
Age in years
AGE AT THE MOMENT OF DECEASE
20..120
Txt_FL_XT109

XT109_DecMarried
Was [{Name of the deceased}] married at the time of [his/ her] death?

IWER:
Deceased married at time of death
DECEASED MARRIED AT TIME OF DEATH

1. Yes
5. No

Txt_FL_XT039

XT039_NumChild
How many children did [{Name of the deceased}] have that were still alive at the time of [his/ her] death?
Please count all natural children, fostered, adopted and stepchildren
NUMBER OF CHILDREN THE DECEASED HAD AT THE END
-1000000000000000000..1000000000000000000
Txt_FL_XT011

XT011_CauseDeath
What was the main cause of [his/ her] death?

IWER:
Read out if necessary
THE MAIN CAUSE OF DEATH
1. Cancer
2. A heart attack
3. A stroke
4. Other cardiovascular related illness such as heart failure, arrhythmia
5. Respiratory disease
6. Disease of the digestive system such as gastrointestinal ulcer, inflammatory bowel disease
7. Severe infectious disease such as pneumonia, septicemia or flu
8. Accident
97. Other (Please specify)

IF XT011_CauseDeath = a97

XT012_OthCauseDeath

IWER:
Specify other cause of death
OTHER CAUSE OF DEATH
STRING

ENDIF
IF XT011_CauseDeath <> a8
Txt_FL_XT013

XT013_HowLongIll
How long had [{Name of the deceased}] been ill before [he/ she] died?

IWER:
{ReadOut}
HOW LONG BEEN ILL BEFORE DECEASE
1. Less than one month
2. One month or more but less than 6 months
3. 6 months or more but less than a year
4. One year or more

Txt_FL_XT014

XT014_WhereDied
Did [he/ she] die ...

IWER:
{ReadOut}
PLACE OF DYING
1. at^FL_XT014_2 own home
2. at another person's home

3. in a hospital
4. in a nursing home
5. in a residential home or sheltered housing
6. in a hospice
97. at some other place

Txt_FL_XT615

XT615_TimesInHosp
In the last year before [he/ she] died, on how many different occasions did [{Name of the deceased}] stay in a hospital, hospice or nursing home?
TIMES IN HOSPITAL LAST YEAR BEFORE DYING
-1000000000000000000..1000000000000000000

IF XT615_TimesInHosp > 1
Txt_FL_XT016

XT016_TotalTimeHosp
During the last year of [his/ her] life, for how long altogether did [{Name of the deceased}] stay at hospitals, hospices or nursing homes?

IWER:
Do not read out
TOTAL TIME IN HOSPITAL LAST YEAR BEFORE DYING
1. Less than one week
2. One week or more but less than one month
3. One month or more but less than 3 months
4. 3 months or more but less than 6 months
5. 6 months or more but less than a year
6. A full year

ENDIF

*ENDIF*Txt_FL_XT017

XT017_IntroMedCare
We would now like to ask you some questions about any expenses which [{Name of the deceased}] incurred as a result of the medical care [he/ she] received in the last 12 months before [he/ she] died.
For each of the types of care I will now list, please indicate whether [{Name of the deceased}] received the care and, if so, give your best estimate of the costs incurred from that care.
@bPlease include only costs not paid or reimbursed by the health insurance or the employer.@b
INTRODUCTION EXPENSES MEDICAL CARE
1. Continue

LOOP cnt:= 1 TO 9

IF ((cnt < 3) OR (cnt > 5)) OR (XT615_TimesInHosp > 1)

BLOCK
Txt_FL_XT018

XT018_TypeMedCare
Did [{Name of the deceased}] have any [care from a general practitioner/ care from specialist physicians/ hospital stays/ care in a nursing home/ hospice stays/ medication/ aids and appliances/ help with personal care due to disability/ help with domestic tasks due to disability] (in the last 12 months of [his/ her] life)?
HAD TYPE OF MEDICAL CARE IN THE LAST TWELVE MONTHS
1. Yes
5. No

IF XT018_TypeMedCare = a1
Txt_FL_XT119

XT119_CostsMedCare
About how much did [he/ she] pay out of pocket for [care from a general practitioner/ care from specialist physicians/ hospital stays/ care in a nursing home/ hospice stays/ medication/ aids and appliances/ help with personal care due to disability/ help with domestic tasks due to disability] (in the last 12 months of [his/ her] life)? [By out of pocket we mean that the costs were not covered or reimbursed by the health insurance/national health system/third party.]

IWER:
Fill in '0' if all the expenses were covered or reimbursed. Otherwise fill in the amount in [FLDefault[9]]
COSTS OF TYPE OF MEDICAL CARE IN THE LAST TWELVE MONTHS
-1000000000000000000..10000000000000000000

IF XT119_CostsMedCare = NONRESPONSE
UB SEQUENCE XT619b_Random

ENDIF

ENDIF

ENDBLOCK

ENDIF

*ENDLOOP*Txt_FL_XT105

XT105_DiffWhere
We would like to know more about the difficulties people have in their last year of life because of a physical, mental, emotional or memory problems. During the last year of [his/ her] life, did [{Name of the deceased}] have any difficulty remembering @bwhere@b [he/ she] was? Please name only difficulties that lasted at least three months?
DIFFICULTIES REMEMBERING WHERE
1. Yes
5. No

Txt_FL_XT106

XT106_DiffYear
During the last year of [his/ her] life, did [{Name of the deceased}] have any difficulty remembering @bwhat year@b it was? Please name only difficulties that lasted at least three months?
DIFFICULTIES REMEMBERING THE YEAR
1. Yes
5. No

Txt_FL_XT107

XT107_DiffRecogn
During the last year of [his/ her] life, did [{Name of the deceased}] have any difficulty @brecognizing@b family members or good friends? Please name only difficulties that lasted at least three months?
DIFFICULTIES RECOGNIZING
1. Yes
5. No

Txt_FL_XT020

XT020_IntroDiffADL
Because of a physical, mental, emotional or memory problem, did [{Name of the deceased}] have difficulty doing any of the following activities during the last twelve months of [his/ her] life? Please name only difficulties that lasted at least three months.

IWER:
{ReadOut}.
{CodeAll}
INTRODUCTION DIFFICULTIES DOING ACTIVITIES

1. Dressing, including putting on shoes and socks
2. Walking across a room
3. Bathing or showering
4. Eating, such as cutting up your food
5. Getting in or out of bed
6. Using the toilet, including getting up or down
96. None of these

CHECK NOT ((XT020_IntroDiffADL.CARDINAL > 1) AND (96 IN XT020_IntroDiffADL)) L1 " [You cannot select ""None of the above"" together with any other answer. Please change your answer]"

IF (XT020_IntroDiffADL.CARDINAL > 0) AND NOT (a96 IN XT020_IntroDiffADL)

Txt_FL_XT022

XT022_HelpADL

Thinking about the activities that [{Name of the deceased}] had problems with during the last twelve months of [his/ her] life, has anyone helped regularly with these activities?

ANYONE HELPED WITH ADL

1. Yes
5. No

IF XT022_HelpADL = a1

Txt_FL_XT023

XT023_WhoHelpedADL

Who, including yourself, has mainly helped with these activities? Please name up to three persons.

IWER:
do not read out
at most three answers!

code relationship to deceased!

WHO HAS HELPED WITH ADL

1. Yourself (proxy respondent)
2. Husband or wife or partner of the deceased
3. Mother or father of the deceased
4. Son of the deceased
5. Son-in-law of the deceased
6. Daughter of the deceased
7. Daughter-in-law of the deceased
8. Grandson of the deceased
9. Granddaughter of the deceased
10. Sister of the deceased
11. Brother of the deceased
12. Other relative
13. Unpaid volunteer
14. Professional helper (e.g. nurse)
15. Friend or neighbor of the deceased
16. Other person

CHECK NOT (XT023_WhoHelpedADL.CARDINAL > 3) L1 " [At most three answers]"

Txt_FL_XT024

XT024_TimeRecHelp

Overall, during the last twelve months of [his/ her] life, for how long did [{Name of the deceased}] receive help?

IWER:
{ReadOut}

TIME THE DECEASED RECEIVED HELP

1. Less than one month
2. One month or more but less than 3 months
3. 3 months or more but less than 6 months

4. 6 months or more but less than a year
5. A full year

Txt_FL_XT025

XT025_HrsNecDay
And about how many hours of help did [{Name of the deceased}] receive during a typical day?
HOURS OF HELP NECESSARY DURING TYPICAL DAY
0..24

ENDIF

*ENDIF*Txt_FL_XT620

XT620_IntroDiffADLII
Because of a physical, mental, emotional or memory problem, did {FL_XT620_1} have difficulty doing any of the following activities during the last twelve months of [his] life?
Please name only difficulties that lasted at least three months.

IWER:
{ReadOut}.
{CodeAll}
INTRODUCTION DIFFICULTIES
1. Preparing a hot meal
2. Shopping for groceries
3. Making telephone calls
4. Taking medication
5. Using a map to figure out how to get around in a strange place
6. Doing work around the house or garden
7. Managing money, such as paying bills and keeping track of expenses
8. Leaving the house independently and accessing transportation services
9. Doing personal laundry
10. Continence over urination or defecation
96. None of these

CHECK NOT ((XT620_IntroDiffADLII.CARDINAL > 1) AND (96 IN XT620_IntroDiffADLII)) L1 " [You cannot select ""None of these"" together with any other answer. Please change your answer.]"

IF (XT620_IntroDiffADLII.CARDINAL > 0) AND NOT (a96 IN XT620_IntroDiffADLII)

Txt_FL_XT622

XT622_HelpADLII
Thinking about the activities that [{Name of the deceased}] had problems with during the last twelve months of [his/ her] life, has anyone helped regularly with these activities?
ANYONE HELPED WITH ADLII
1. Yes
5. No

IF XT622_HelpADLII = a1

Txt_FL_XT623

XT623_WhoHelpedADLII
Who, including yourself, has mainly helped with these activities? Please name up to three persons.

IWER:
do not read out
at most three answers!

code relationship to deceased!
WHO HAS HELPED WITH ADLII
1. Yourself (proxy respondent)
2. Husband or wife or partner of the deceased
3. Mother or father of the deceased

4. Son of the deceased
5. Son-in-law of the deceased
6. Daughter of the deceased
7. Daughter-in-law of the deceased
8. Grandson of the deceased
9. Granddaughter of the deceased
10. Sister of the deceased
11. Brother of the deceased
12. Other relative
13. Unpaid volunteer
14. Professional helper (e.g. nurse)
15. Friend or neighbor of the deceased
16. Other person

CHECK NOT (XT023_WhoHelpedADL.CARDINAL > 3) L1 " [At most three answers]"
Txt_FL_XT624

XT624_TimeRecHelp
Overall, during the last twelve months of [his/ her] life, for how long did [{Name of the deceased}] receive help?

IWER:
{ReadOut}
TIME THE DECEASED RECEIVED HELP
1. Less than one month
2. One month or more but less than 3 months
3. 3 months or more but less than 6 months
4. 6 months or more but less than a year
5. A full year

Txt_FL_XT625

XT625_HrsNecDay
And about how many hours of help did [{Name of the deceased}] receive during a typical day?
HOURS OF HELP NECESSARY DURING TYPICAL DAY
0..24

ENDIF

*ENDIF*Txt_FL_XT026a

XT026a_Intro
The next questions are about the assets and life insurance policies [{Name of the deceased}] may have owned and what happened to those assets after [he/ she] died. We would find it very helpful to have some information about the financial issues surrounding the time when people die. Before I continue, though, I'd like to assure you again that everything you have already told me and anything else you tell me will be kept completely confidential.
INTRODUCTION TO ASSETS
1. Continue

Txt_FL_XT026b

XT026b_HadWill
Some people make a will to determine who receives what parts of the estate.
Did [{Name of the deceased}] have a will?
THE DECEASED HAD A WILL
1. Yes
5. No

Txt_FL_XT027

XT027_Benefic
Who were the beneficiaries of the estate, including yourself?

IWER:
{ReadOut}
{CodeAll}
THE BENEFICIARIES OF THE ESTATE
1. Yourself (proxy)
2. Husband or wife or partner of the deceased
3. Children of the deceased
4. Grandchildren of the deceased
5. Siblings of the deceased
6. Other relatives (specify) of the deceased
7. Other non-relatives (specify)
8. Church, foundation or charitable organization
9. Deceased did not leave anything at all (SPONTANEOUS)

CHECK NOT ((XT027_Benefic.CARDINAL > 1) AND (9 IN XT027_Benefic)) L1 " [You cannot select ""Did not leave anything"" together with any other answer. Please change your answer.]"
Txt_FL_XT030

XT030_OwnHome
Did [{Name of the deceased}] own [his/ her] home or apartment - either in total or a share of it?
THE DECEASED OWNED HOME
1. Yes
5. No

IF XT030_OwnHome = a1
Txt_FL_XT031

XT031_ValHome
After any outstanding mortgages, what was the value of the home or apartment or the share of it owned by [{Name of the deceased}]?

IWER:
Enter an amount in [FLDefault[9]]
If deceased left debt, code negative amount.
VALUE HOME AFTER MORTGAGES
-50000000..50000000

IF XT031_ValHome = NONRESPONSE
UB SEQUENCE XT031b_Random

*ENDIF*Txt_FL_XT032

XT032_InhHome
Who inherited the home or apartment of [{Name of the deceased}], including yourself?

IWER:
Code relationship to deceased.
{CodeAll}
If the home or apartment is already sold, code all persons who got a share of the money.
WHO INHERITED THE HOME OF THE DECEASED
1. Yourself (proxy respondent)
2. Husband or wife or partner
3. Sons or daughters (ASK FOR FIRST NAMES)
4. Grandchildren
5. Siblings
6. Other relatives
7. Other non-relatives

IF a3 IN XT032_InhHome

XT053_FrstNme

IWER:
First names of children who inherited home
FIRST NAMES CHILDREN
STRING

ENDIF

*ENDIF*Txt_FL_XT033

XT033_OwnLifeInsPol
Did [{Name of the deceased}] own any life insurance policies?
THE DECEASED OWNED ANY LIFE INSURANCE POLICIES
1. Yes
5. No

IF XT033_OwnLifeInsPol = a1
Txt_FL_XT034

XT034_ValLifeInsPol
Approximately what was the total value of all life insurance policies owned by [{Name of the deceased}]?

IWER:
Enter an amount in [FLDefault[9]]
VALUE OF ALL LIFE INSURANCE POLICIES
-1000000000000000000..1000000000000000000
Txt_FL_XT035

XT035_BenLifeInsPol
Who were the beneficiaries of the life insurance polices, including yourself.

IWER:
Code relationship to deceased
{CodeAll}
BENEFICIARIES OF THE LIFE INSURANCE POLICIES
1. Yourself (proxy respondent)
2. Husband or wife or partner
3. Sons or daughters (ASK FOR FIRST NAMES)
4. Grandchildren
5. Siblings
6. Other relatives (specify)
7. Other non-relatives (specify)

IF a6 IN XT035_BenLifeInsPol

XT054_OthRel

IWER:
Specify other relative
OTHER RELATIVE
STRING

ENDIF
IF a7 IN XT035_BenLifeInsPol

XT055_OthNonRel

IWER:
Specify other non-relative
OTHER NO-RELATIVE

STRING

ENDIF
IF a3 IN XT035_BenLifeInsPol

XT056_FrstNme

IWER:
First names of children who were beneficiaries
FIRST NAMES CHILDREN
STRING

ENDIF

*ENDIF*Txt_FL_XT036

XT036_IntroAssets
I will now read out a few types of assets people may have. For each item, please tell me whether [{Name of the deceased}] owned them at the time of [his/ her] death and, if so, please give your best estimate of their value after any outstanding debts.
INTRODUCTION TYPES OF ASSETS
1. Continue

LOOP cnt:= 1 TO 5

BLOCK
Txt_FL_XT637

XT637_OwnAss
Did [he/ she] own any [businesses, including land or premises/ other real estate/ cars, except leased cars/ financial assets, e.g. cash, bonds or stocks/ jewelry or antiquities]?
THE DECEASED OWNED TYPE OF ASSETS
1. Yes
5. No

IF XT637_OwnAss = a1
Txt_FL_XT638

XT638_ValAss
About what was the value of the [businesses, including land or premises/ other real estate/ cars, except leased cars/ financial assets, e.g. cash, bonds or stocks/ jewelry or antiquities] owned by [{Name of the deceased}] at the time of [his/ her] death?

IWER:
Enter an amount in [FLDefault[9]]
If deceased left debt, code negative amount.
VALUE TYPE OF ASSETS
-50000000..50000000

IF XT638_ValAss = NONRESPONSE
UB SEQUENCE XT638b_Random

ENDIF

ENDIF

ENDBLOCK

ENDLOOP
IF (XT039_NumChild > 1) AND NOT (a9 IN XT027_Benefic)
Txt_FL_XT040a

XT040a_EstateDiv
How would you say that the total estate was divided among the children of [{Name of the deceased}]?

IWER:
{ReadOut}
TOTAL ESTATE DIVIDED AMONG THE CHILDREN
1. Some children received more than others
2. The estate was divided about equally among all children
3. The estate was distributed exactly among the children
4. The children have not received anything
5. Estate @bnot@b divided yet (SPONTANEOUS ONLY)

IF XT040a_EstateDiv = a1
Txt_FL_XT040b

XT040b_MoreForCare
Would you say that some children received more than others to make up for previous gifts?
SOME CHILDREN RECEIVED MORE FOR CARING
1. Yes
5. No

Txt_FL_XT040c

XT040c_MoreFinSupp
Would you say that some children received more than others to give them financial support?
SOME CHILDREN RECEIVED MORE TO GIVE THEM FINANCIAL SUPPORT
1. Yes
5. No

Txt_FL_XT040d

XT040d_MoreForCare
Would you say that some children received more than others because they helped or cared for [{Name of the deceased}] towards the end of [his/ her] life?
SOME CHILDREN RECEIVED MORE FOR CARING
1. Yes
5. No

Txt_FL_XT040e

XT040e_MoreOthReas
Would you say that some children received more than others because of other reasons?
SOME CHILDREN RECEIVED MORE FOR OTHER REASONS
1. Yes
5. No

ENDIF

*ENDIF*Txt_FL_XT041

XT041_Funeral
Finally, we would like to know about the funeral of [{Name of the deceased}]. Was the funeral accompanied by a religious ceremony?
THE FUNERAL WAS ACCOMPANIED BY A RELIGIOUS CEREMONY
1. Yes
5. No

Txt_FL_XT108

XT108_AnyElse
We have asked you many questions about numerous aspects of [{Name of the deceased}]'s health and

finances, and we want to thank you very much for your assistance with them. Is there anything else you would like to add about the life circumstances of [{Name of the deceased}] in [his/ her] last year of life?

IWER:
If nothing to say, type none and press enter
ANYTHING ELSE TO SAY ABOUT THE DECEASED
STRING

Txt_FL_XT042

XT042_Outro
This is the end of the interview. Thank you once again for all the information you have given us. It will prove extremely useful in helping us to understand how people fare at the end of their lives
THANKS FOR THE INFORMATION
1. Continue

XT043_IntMode

IWER:
Please state mode of interview
INTERVIEW MODE
1. Face-to-face
2. Telephone

XT044_IntID

IWER:
Your interviewer id.
INTERVIEWER ID
STRING

System preset value XT902_TimeStampEnd
IF XT902_TimeStampEnd = EMPTY AND XT042_Outro <> EMPTY
ENDIF

ENDBLOCK

ENDIF
